# Patient-reported outcome measures for atrial fibrillation: a systematic review and evaluation of psychometric properties of symptom severity measures

**DOI:** 10.1093/europace/euag252

**Published:** 2026-09-08

**Authors:** Jael-Joy Ogendo, Angeliki Darma, Miroslav Mihajlovic, Elena Arbelo, Deirdre A Lane

**Affiliations:** Department of Cardiovascular and Metabolic Medicine, Institute of Life Course and Medical Sciences,, University of Liverpool, William Henry Duncan Building, 6 West Derby Street, Liverpool L7 8TX, UK; Clinic for Electrophysiology, Herz- und Diabeteszentrum NRW, Ruhr-Universität Bochum, Bad Oeynhausen, Germany; Cardiology Clinic, University Clinical Centre of Serbia, Belgrade, Serbia; Arrhythmia Section, Cardiology Department, Hospital Clínic, Universitat de Barcelona, Barcelona, Spain; Department of Cardiovascular and Metabolic Medicine, Institute of Life Course and Medical Sciences,, University of Liverpool, William Henry Duncan Building, 6 West Derby Street, Liverpool L7 8TX, UK; Liverpool Centre for Cardiovascular Sciences, University of Liverpool, Liverpool L7 8TX, UK

**Keywords:** Atrial fibrillation, Patient-reported outcomes, Patient-reported outcome measures, Psychometric properties, Systematic review

## Abstract

**Aims:**

Collection of patient-reported outcomes (PROs) is advocated for benchmarking atrial fibrillation (AF) care; however, concerns over the robustness of patient-reported outcome measures (PROMs) limit their use in clinical practice. This systematic review identified PROMs used to evaluate health-related quality of life, physical, emotional, and cognitive function, symptom severity, exercise tolerance, and ability to work and appraised the measurement properties of symptom severity PROMs.

**Methods and results:**

Six bibliographic databases were searched from inception until 11 January 2025. Full-text publications in all languages investigating ≥1 measurement property of PROMs in AF patients aged ≥18 years were eligible. The Consensus-based Standards for the selection of health Measurement Instruments (COSMIN) recommendations appraised and synthesized measurement properties and risk of bias of symptom severity PROMs (PROSPERO ID: CRD42024523340). A total of 709 eligible publications identified 101 PROMs; six symptom severity PROMs (AFSymp, AFS/B, AFSS, ASTA, AF6, and SCL) underwent psychometric property evaluation. ASTA, AFSymp, and SCL indicated evidence of sufficient content validity and at least low-quality evidence for sufficient internal consistency. ASTA was the most validated PROM and exhibited the greatest level of certainty in evidence quality across assessed psychometric properties. COSMIN assessments revealed critical gaps in responsiveness and cross-cultural validity.

**Conclusion:**

ASTA emerged as the most robust symptom severity PROM. Further validation of PROMs identified for other PROs by COSMIN guidelines is recommended prior to their routine integration in AF care, and future studies should address responsiveness, interpretability, feasibility, and cross-cultural validity to ensure PROMs reliably reflect patients’ experiences and detect meaningful clinical changes across diverse populations.

## Graphical Abstract

**Graphical Abstract:**
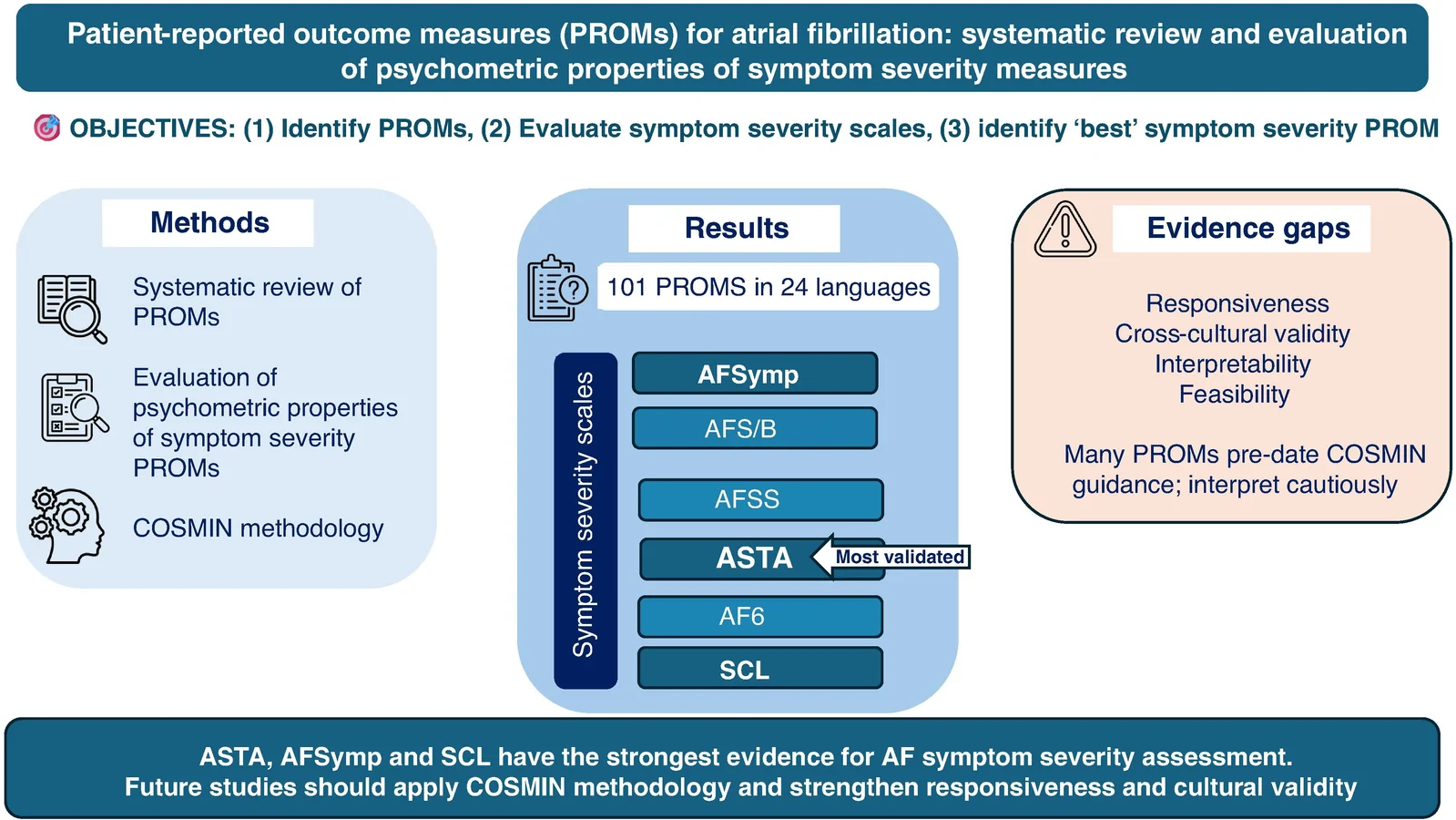

## Introduction

Atrial fibrillation (AF) is the most common cardiac arrhythmia in clinical practice, with AF and atrial flutter collectively affecting ∼52.6 million individuals globally.^1–3^ AF is associated with significant morbidity and mortality and reduced quality of life (QoL).^4,5^ Clinical guidelines advocate a holistic approach to AF management, and patient-reported outcomes (PROs) are increasingly recognized as important measures of the quality of care.^3,6^ The International Consortium for Health Outcomes Measurement AF working group identified the following PROs as important: health-related quality of life (HRQoL), physical, emotional, and cognitive function, symptom severity, exercise tolerance, and ability to work.^7^ The 2021 EHRA consensus paper on quality indicators of AF management recommended the measurement of the same PROs, with the exception of ‘ability to work’.^8,9^

PROs are optimally collected using validated patient-reported outcome measures (PROMs);^10,11^ however, PROMs are not routinely used in clinical practice.^2^ This may be related to doubt over their robustness regarding their psychometric properties (i.e. validity, reliability, and responsiveness), the diversity of measures available, difficulties with incorporating routine collection into clinical management, and lack of knowledge of how to interpret the findings and integrate the information into the care plan.^6,8^ Therefore, further research is required to rationalize their routine integration in AF care.^8^

A systematic review assessing psychometric properties of AF-specific QoL PROMs concluded that only one of the five evaluated PROMs demonstrated strong positive evidence, but only for two out of nine psychometric properties assessed.^12^ An updated review concluded that further validation research was required for the six AF-specific QoL PROMs examined, as sufficient evidence for key psychometric properties, including cross-cultural validity, measurement error, and responsiveness, was lacking.^13^

Therefore, this systematic review aimed to (i) identify psychometrically robust instruments to measure HRQoL, physical, emotional, and cognitive function, symptom severity, exercise tolerance, and ability to work; (ii) assess the psychometric measurement properties and methodological quality of the PROMs evaluating symptom severity; and (iii) offer recommendations on the ‘best’ symptom severity PROM for use in routine clinical practice to inform value-based healthcare and improve health outcomes.

## Methods

This systematic review was reported in line with recommendations outlined by the Consensus-based Standards for the selection of health Measurement Instruments (COSMIN) expansion of the Preferred Reporting Items for Systematic Reviews and Meta-analyses (PRISMA) and Synthesis Without Meta-analysis in systematic reviews guidelines.^14–17^ A completed PRISMA-COSMIN checklist is detailed in Supplementary material online, *Appendix B*. The protocol was registered in the International Prospective Register of Systematic Reviews (PROSPERO): ID CRD42024523340.

### Eligibility criteria

Studies fulfilling the following inclusion criteria were eligible: (i) studies in humans aged ≥18 years with any AF type; (ii) those utilizing a validated PROM (AF-specific, arrhythmia-specific, or generic, such as checklists, questionnaires, and scales) to assess one or more of the PROs of interest (see Supplementary material online, *Table S1*) in an AF population; (iii) those evaluating ≥1 psychometric measurement property (validity, reliability, or responsiveness) of a validated PROM to assess ≥1 PROs of interest in individuals with AF; (iv) randomized controlled trials (RCTs), pseudo-RCTs, methodological studies, observational designs (cohort, case-control, and cross-sectional studies), study protocols, systematic reviews, and mixed-methods studies; and (v) where the full-text publication was available. No language or publication date restrictions were applied. Studies that assessed ≥1 PRO of interest in an AF cohort but did not specify the PROM used, used a physician-reported measure, used a PROM specific to other diseases, or used a study-specific measure that lacked validation were excluded.

### Search strategy

Search strings included keywords related to the population of interest (AF), combined with validated sensitive search-term filters to identify PROMs^18^ and psychometric measurement properties,^19^ which were adapted for each database using the Polyglot Search Translator tool.^20^ Complete search strategies and database interfaces used are provided in Supplementary material online, *Appendix A*.

The electronic bibliographic databases PubMed, MEDLINE, Cumulative Index to Nursing and Allied Health Literature, APA PsycINFO, Cochrane Library, and Web of Science (for grey literature through Conference Proceedings Citation Index–Science) were searched from their respective dates of inception until 2 February 2024, with searches updated on 11 January 2025. Bibliographies of topical systematic reviews and methodological studies were hand-searched to identify additional studies not identified by the searches. Study authors were contacted for further information, including pre-print reports or full-text publications, where conference proceedings were identified, as required.

### Study selection

Studies from database searches were imported into EndNote for deduplication and then exported into Rayyan for screening.^21^ Titles and abstracts were first evaluated to determine eligibility according to the study criteria. Full-text publications, following title and abstract screening, were then sought and evaluated for relevance. For both stages, screening was conducted independently by J.J.O. for all identified reports and independently in duplicate (D.L., A.D., M.M. E.A.), who screened a quarter each. Native speakers were consulted for the translation of potentially eligible non-English publications to determine their eligibility. All disagreements during study screening, study quality evaluation, data extraction, and data synthesis were settled via discussion to reach consensus, with a third reviewer being consulted if necessary.

### Data extraction

Where available, the following data were extracted: study characteristics: authors, publication year, nations, study design, total participants (*n*), and demographics (age, gender proportions, ethnicity/race, and AF type), and PRO being assessed; PROM characteristics (language, domains and/or items, response options, feasibility and interpretability factors, and psychometric properties assessed).

### Quality assessment

For each included methodological study evaluating measurement properties (see Supplementary material online, *Table S2*) of symptom severity PROMs, the COSMIN Risk-of-Bias (RoB) checklist was used, and judgements were recorded using the COSMIN scoring form.^14,17,22,23^ J.J.O. conducted independent assessments of all eligible PROMs, while D.L., A.D., and M.M. performed duplicate independent assessments. Measurement properties were evaluated as either very good, adequate, doubtful, or inadequate, with the lowest score determining the global rating for each measurement property.^14,17,22,23^ Each methodological study was then rated against the COSMIN criteria for good measurement properties (see Supplementary material online, *Table S2*) and COSMIN’s 10 content validity criteria as sufficient (+), insufficient (−), indeterminate (?), or inconsistent (±).^14,23^

### Data synthesis

Meta-analysis was not feasible due to heterogeneity among included studies. Data from individual studies evaluating the same PROM were qualitatively summarized based on each measurement property and, for applicable content validity studies, by relevance, comprehensiveness, and comprehensibility.^14,17,23^ Measurement properties were combined into a global rating for each PROM. Consistent ratings of the included individual methodological studies for each PROM led to global ratings of sufficient (+), insufficient (−), or indeterminate (?).^14,17,23^ If there was evidence of both sufficient and insufficient measurement properties in individual methodological studies, the PROM’s global rating was determined by the majority rating of each measurement property.^14,17,23^ When adequate content validity data were lacking, reviewers’ ratings determined global PROM content validity evaluations.^23^

The certainty of evidence for each study was subsequently evaluated with the modified Grading of Recommendations, Assessment, Development and Evaluation (GRADE) framework as either high, moderate, low, or very low, based on overall RoB, measurement properties and the imprecision, indirectness, or inconsistency of study results.^14,17,24^ Evidence synthesis was conducted independently by two reviewers (JJO plus one other author).

Recommendations were then made regarding the ‘best’ PROM for use to assess symptom severity in individuals with AF. PROMs that demonstrated sufficient content validity, along with at least low-quality evidence supporting sufficient internal consistency, were recommended for use.^14,17^

## Results

From 18 839 records identified from database searches, 1176 full-text articles were evaluated for inclusion, and 709 reports (covering 600 studies) were eligible for qualitative synthesis (*Figure 1*). All excluded studies, with labelled reasons for exclusion, are available for review on Rayyan upon request.

**Figure 1:**
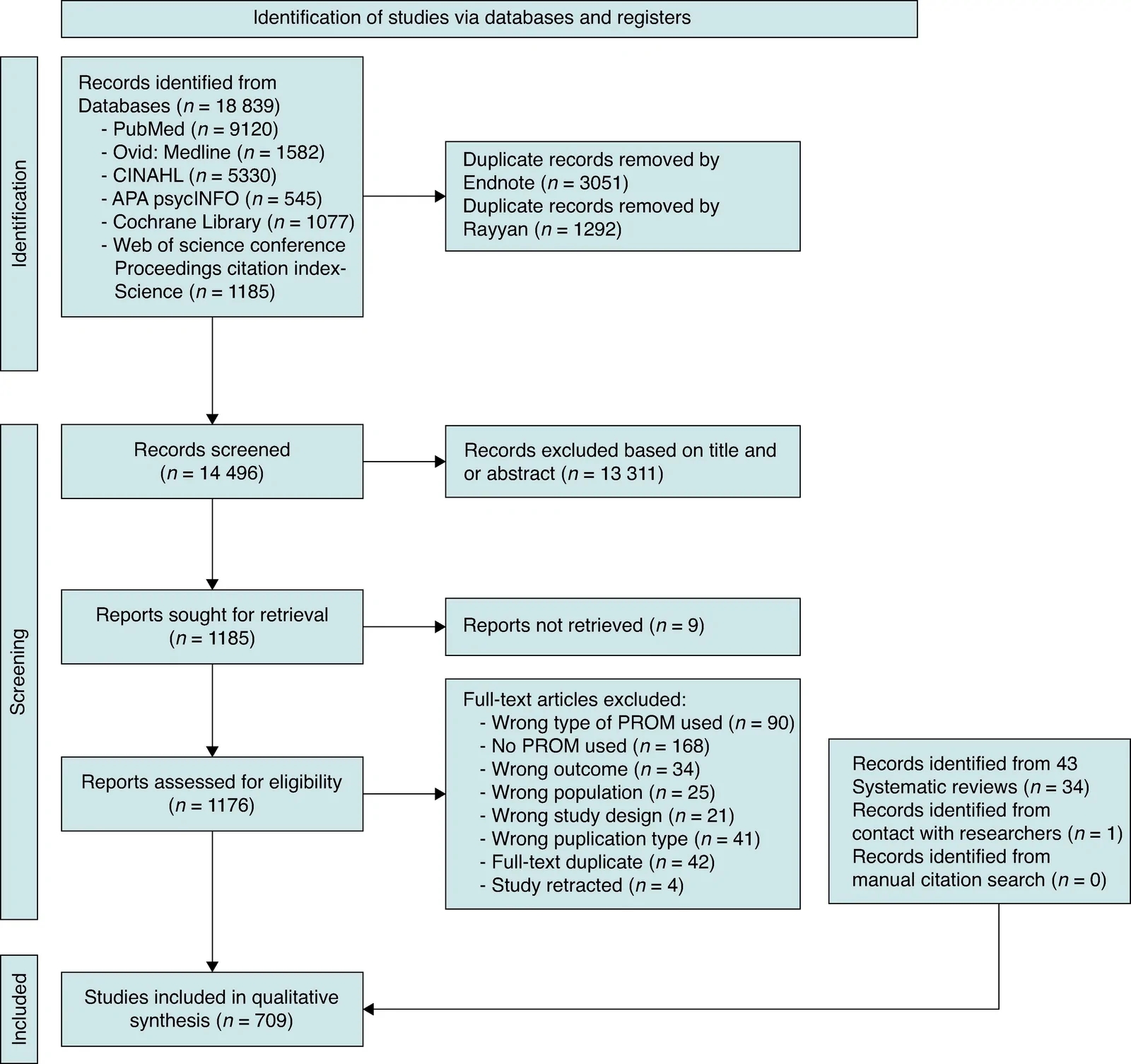
PRISMA flow diagram of the study selection process.

Thirteen were case-control, 332 cohort, 58 cross-sectional, 38 methodological, nine mixed-methods, 253 RCTs, four survey studies, one pseudo-RCT, and one analysis study conducted across 60 countries (see Supplementary material online, *Table S3*). Sample sizes ranged from 6^25^ to 66 692^26^ participants, with age ranging from 34.4 (±12.3)^27^ to 85.2 (±5.16) years.^28^ Of 642 articles reporting gender, females constituted <30% in 199 studies, 30–49% in 366 studies, 50–59% in 51 studies, 60–69% in 21 studies, and ≥70% in five studies. In 91 of the 107 studies reporting race and/or ethnicity, over 80% of included participants were White/Caucasian. Patients with all types of AF were included.

### Patient-reported outcomes identified

Included studies often assessed and reported on more than one PRO. HRQoL was the most frequently evaluated PRO, assessed in 571 (81%) studies. Symptom severity was assessed in 156 (22%) studies, emotional function in 148 (21%), cognitive function in 100 (14%), physical function in 36 (5%), exercise tolerance in 18 (3%), and ability to work in four (1%) studies. Overall, 101 PROMs in 24 languages were identified (*Figure 2*). Thirty-eight PROMs (38%) assessed HRQoL, 31 (31%) assessed emotional function, nine (9%) assessed physical function, eight (8%) assessed exercise tolerance, seven (7%) assessed symptom severity, six (6%) assessed cognitive function, and two (2%) assessed the ability to work. Eighty-one PROMs were generic, 12 were AF-specific, five were arrhythmia-specific, two were cardiovascular condition-specific, and one was for multiple chronic conditions.

**Figure 2:**
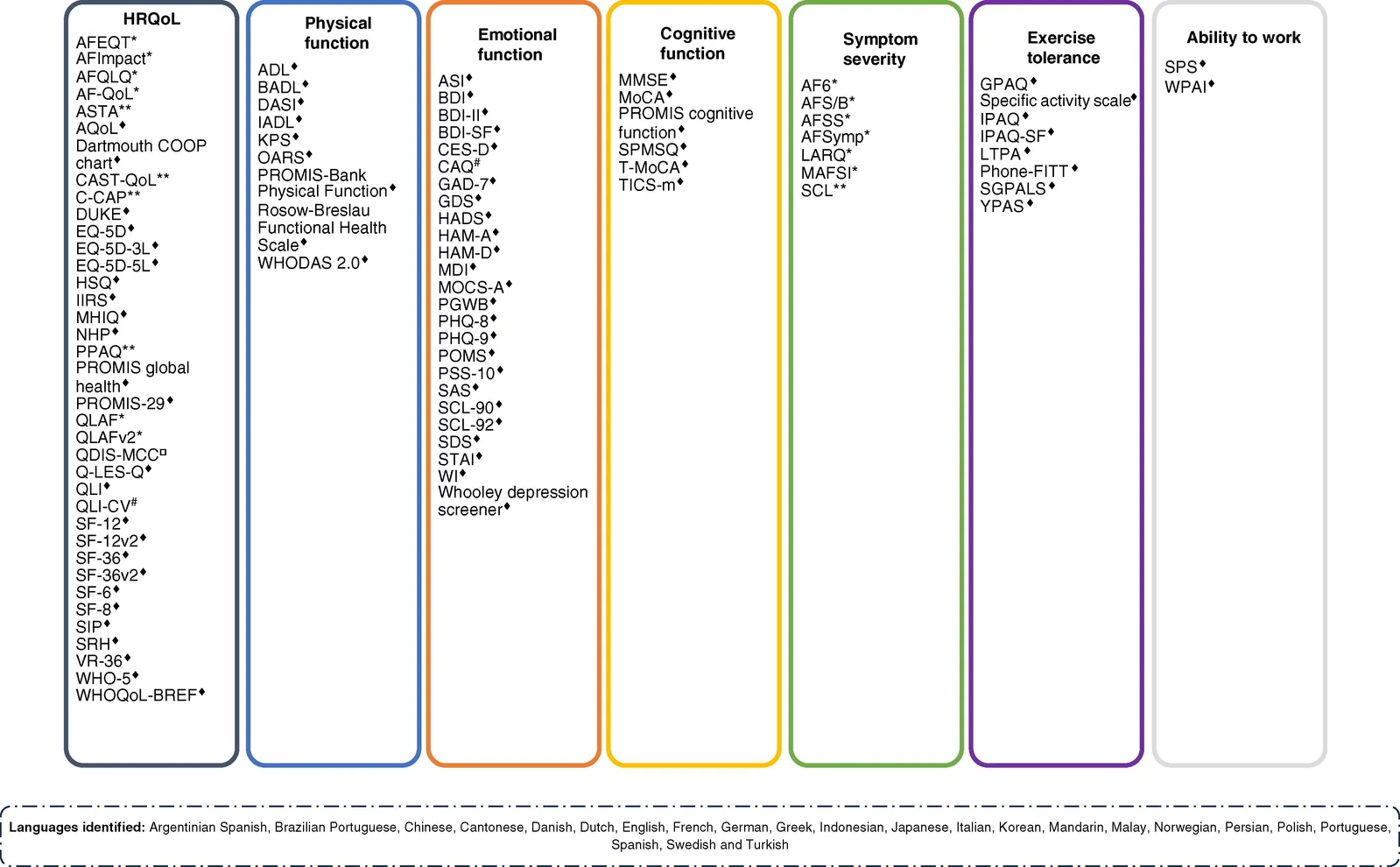
Patient-reported outcome measures, with languages of tools specified by studies, identified for measuring HRQoL, physical function, emotional function (defined as stress, depression, or anxiety), cognitive function, symptom severity, exercise tolerance, and ability to work in individuals with atrial fibrillation. ADL, Katz index of independence in Activities of Daily Living; AF6, Atrial Fibrillation six-item questionnaire; AFEQT, Atrial Fibrillation Effect on QualiTy-of-life; AFImpact, Atrial Fibrillation Impact; AFQLQ, Atrial fibrillation Quality of Life Questionnaire; AF-QoL, Atrial Fibrillation-Quality of Life; AFS/B, Atrial Fibrillation Symptom and Burden; AFSS, Atrial Fibrillation Symptom Severity; AFSymp, Atrial Fibrillation Symptom questionnaire; AQoL, Australian Quality of Life; ASI, Anxiety Sensitivity Index; ASTA, Arrhythmia-Specific questionnaire in Tachycardia and Arrhythmia; BADL, Barthel Activities of Daily Living; BDI, Beck Depression Inventory; BDI-II, Beck Depression Inventory version two; BDI-SF, Beck Depression Inventory Short Form; CAQ, Cardiac Anxiety Questionnaire; CAST-QoL, Cardiac Arrhythmia Suppression Trial Quality of Life questionnaire; C-CAP, Cardiff Cardiac Ablation Patient-reported outcome measure; CES-D, Centre for Epidemiologic Studies Depression scale; Dartmouth COOP chart, Dartmouth Care Cooperative Information Project chart system; DASI, Duke Activity Status Index; DUKE, Duke Health Profile; EQ-5D, EuroQol five dimensions questionnaire; EQ-5D-3L, EuroQol five dimensions questionnaire (three level); EQ-5D-5L, EuroQol five dimensions questionnaire (five level); EQ-VAS, EuroQol Visual Analogue Scale; GAD-7, General Anxiety Disorder seven-item questionnaire; GDS, Geriatric Depression Scale; GDS-5, Geriatric Depression Scale five item; GDS-5, Geriatric Depression Scale 15 item; GPAQ, Global Physical Activity Questionnaire; HADS, Hospital Anxiety and Depression Scale; HAM-A, Hamilton Anxiety Scale; HAM-D, Hamilton Depression Scale; HRQoL, health-related quality of life; HSQ, Health Status Questionnaire; IADL, Lawton Instrumental Activities of Daily Living; IIRS, Illness Intrusiveness Ratings Scale; IPAQ, International Physical Activity Questionnaire; IPAQ-SF, International Physical Activity Questionnaire-Short Form; KPS, Karnofsky Performance Scale; LARQ, Leuven Arrhythmia Questionnaire; LTPA, Minnesota Leisure Time Physical Activity questionnaire; MAFSI, Mayo Atrial Fibrillation-Specific Symptom Inventory; MDI, Major Depression Inventory; MHIQ, McMaster Health index Questionnaire; MMSE, Mini-Mental State Examination; MoCA, Montreal Cognitive Assessment; MOCS-A, Measure of Current Status-Anxiety; NHP, Nottingham Health Profile; OARS, Older Americans’ Resources and Services Multidimensional Functional Assessment Questionnaire; PGWB, Psychological General Well-Being index; Phone-FITT, Physical Activity Frequency Intensity Time and Type questionnaire; PHQ-2, Patient Health Questionnaire two item; PHQ-8, Patient Health Questionnaire eight item; PHQ-9, Patient Health Questionnaire nine item; POMS, Profile of Mood States questionnaire; PPAQ, Patient Perspective of Arrhythmia questionnaire; PROMIS, Patient-Reported Outcomes Measurement Information System; PROMIS-29, Patient-Reported Outcomes Measurement Information System (29 items); PROMIS-Bank Depression, Patient-Reported Outcomes Measurement Information System Bank Depression; PSWQ, Penn State Worry Questionnaire; PSS-4, Perceived Stress Scale (four items); PSS-10, Perceived Stress Scale (10 items); QDIS-MCC, Quality of Life Disease Impact Scale–Multiple Chronic Conditions; QLAF, Quality of Life in AF patients questionnaire; QLAFv2, Quality of Life in AF patients questionnaire version two; Q-LES-Q, Quality of Life Enjoyment and Satisfaction Questionnaire; QLI, Quality of Life Index; QLI-CV, Quality of Life Index–Cardiac Version; VR-36, Veterans RAND (Research and Development) 36 Item Health Survey; SAS, Zung Self-Rating Anxiety Scale; SCL, Symptom Checklist: Frequency and Severity Scale; SCL-90, Symptom Checklist (90 items); SCL-92, Symptom Checklist (92 items); SDS, Zung Self-Rating Depression Scale; SF-12, 12-item Short Form Survey; SF-12v2, 12-item Short Form Survey version two; SF-36, 36-item Short Form Survey; SF-36v2, 36-item Short Form Survey version two; SF-6, 6-item Short Form Survey; SF-8, 8-item Short Form Survey; SGPALS, Saltin-Grimby Physical Activity Level Scale; SIP, Sickness Impact Profile; SPMSQ, Short Portable Mental Status Questionnaire; SPS, Stanford Presenteeism Scale; SRH, Self-Rated Health measure; STAI, Spielberger State-Trait Anxiety Inventory; T-MoCA, Telephone-based Montreal Cognitive Assessment; TICS-m, Telephone Interview for Cognitive Status-modified; WHO-5, World Health Organization- Five Well-Being Index; WHODAS 2.0, World Health Organization Disability Assessment Schedule version two; WHOQoL-BREF, World Health Organization Quality of Life-Brief Version questionnaire; WI, Whitely Index; WPAI, Work Productivity and Activity Impairment Instrument; YPAS, Yale Physical Activity Survey. * = Patient-reported outcome measure specific to individuals with atrial fibrillation. ** = Patient-reported outcome measure specific to individuals with arrhythmias. # = Patient-reported outcome measure specific to individuals with cardiovascular conditions. ^□^ = Patient-reported outcome measure specific to those with chronic conditions. ♦ = Generic patient-reported outcome measure. Not all studies specified the languages used for each patient-reported outcome measure included in the study.

The 36-item Short Form Health Survey (SF-36)^29^ was the most frequently reported generic PROM and was used in 37% (*n* = 264) of the included studies. The Atrial Fibrillation Effect on QualiTy-of-life (AFEQT)^30^ was the most reported AF-specific QoL PROM, appearing in 25% (*n* = 174) of included studies.

### Symptom severity measures

Seven symptom severity PROMs were identified: the Atrial Fibrillation Symptoms questionnaire (AFSymp),^31^ Atrial Fibrillation Symptom and Burden classification system (AFS/B),^32^ Atrial Fibrillation Severity Scale (AFSS),^25,33,34^ Atrial Fibrillation six-item questionnaire (AF6),^35,36^ Symptom Checklist: Frequency and Severity Scale (SCL),^37,38,39^ Leuven Arrhythmia Questionnaire (LARQ),^40^ and the Mayo AF-Specific Symptom Inventory (MAFSI).^41^ Although the Arrhythmia-Specific questionnaire in Tachycardia and Arrhythmia (ASTA)^42–47^ is deemed a HRQoL PROM, it includes a separate symptom severity scale and was therefore included in COSMIN assessments.

LARQ and MAFSI were excluded from COSMIN assessment due to unsuccessful retrieval of methodological papers from corresponding study authors. Similarly, full-text development papers for AFSS^48^ (published as a conference proceeding) and SCL were unavailable; only validation papers for these PROMs were included in COSMIN assessments.

*Table 1* outlines the main characteristics of the 16 included methodological studies for PROMs assessing symptom severity, and *Table 2* provides a summary of the features of these PROMs. Development studies were available for the AFSymp,^31^ AFS/B,^32^ ASTA,^42,46^ and AF6.^36^ Further validation studies were not identified for the AFSymp^29^ and AFS/B.^30^ The eligible PROMs had between 6^35,36^ and 22^42–47^ items, all using Likert scale response options. AFSymp,^31^ AFS/B,^32^ and ASTA^42–47^ methodological studies provided evidence indicating that these PROMs were available in both electronic and paper format.

**Table 1:** Characteristics of identified development and validation studies for patient-reported outcome measures used to assess symptom severity in atrial fibrillation patients

PROM	Study characteristics				
	Country	Sample size (*n*)	Age in years [mean ± SD or median (IQR)]	Females	AF type or arrhythmias identified
**AFSymp** ^31^	USA, UK, France, Germany, and Japan	91 (for concept elicitation) and 313 (for psychometric evaluation)	65 ± 11.3	40%	Paroxysmal (32.9%), persistent (32%), and permanent (35.1%) AF
**AFS/B** ^32^	USA	224	63.7 ± 11	26%	Paroxysmal (55%), long-standing persistent (13%), and persistent (33%) AF
**AFSS** ^25,33,45^	Denmark,^25^ Turkey,^33^ and Indonesia^34^	6,^25^ 130,^33^ and 60^34^	75.5 (72.8–78.3),^25^ 63.1 ± 10.9,^33^ and 58.8^34^	50%,^25^ 41.5%,^33^ and 46.7%^34^	Paroxysmal (66.7%),^25^ (30%),^33^ (18.3%)^34^; persistent (16.7%),^25^ (6.2%),^33^ (15%)^34^; long-standing persistent (3.3%)^32^; permanent (0%),^25^ (63.8%),^33^ (63.3%)^34^; and unclassified (16.7%)^25^ AF
**ASTA** ^40–45^	Sweden,^40,44^ Poland,^41^ Brazil,^42^ Australia,^43^ and Indonesia^45^	147,^40^ 244,^41^ 140,^42^ 270,^44^ 202,^43^ and 60^45^	58.9 ± 12,^40^ 70.7 ± 10.7,^41^ 57.2 ± 13.1,^42^ 66.9 ± 12.2,^43^ 59.3 ± 12.9,^44^ and 58.8^45^	33%,^40^ 56.6%,^41^ 62.1%,^42^ 34%,^44^ 30.2%,^43^ and 53.3%^45^	AFL, AF, AVNRT, ventricular extrasystoles, WPW, atrial macro re-entry, ventricular arrhythmia, AT, and VT
**AF6** ^35,36^	Sweden^35,36^	111^35,36^	67 ± 12^35,36^	20%^33,34^	Persistent AF (100%)^35,36^
**SCL** ^37–39^	Germany,^37^ USA,^38^ and Sweden^39^	60,^37^ 159,^38^ and 646^39^	58 ± 11,^37^ 49 ± 15.8,^38^ and 61.4 ± 1.5^39^	35%,^37^ 55.97%,^38^ and 45.36%^39^	WPW, AVNRT, AF, AT, VT, and AFL

AF, atrial fibrillation; AF6, Atrial Fibrillation six-item questionnaire; AFL, atrial flutter; AFS/B, Atrial Fibrillation Symptom and Burden measure; AFSS, Atrial Fibrillation Symptom Severity Scale; AFSymp, Atrial Fibrillation Symptoms questionnaire; ASTA, Arrhythmia-Specific questionnaire in Tachycardia and Arrhythmia; AT, atrial tachycardia; AVNRT, Atrioventricular-Nodal Re-entry Tachycardia; AVRT, Atrioventricular Re-entrant Tachycardia; IQR, interquartile range; NR, not reported; PROM, patient-reported outcome measure; SCL, Symptom Checklist: Frequency and Severity Scale; SD, standard deviation; UK, United Kingdom; USA, United States of America; VT, ventricular tachycardia; WPW, Wolff–Parkinson–White syndrome.

**Table 2:** Characteristics of identified patient-reported outcome measures used to assess symptom severity in atrial fibrillation

PROM	PROM characteristics					
	Years		Items	Response options (all used Likert scales)	Scoring	Feasibility and interpretability (PROM format, missing item scores, and completion times)
	Development	Further validation				
**AFSymp** ^a^	2014^31^	NI	11 items; 3 domains: heart symptoms, tiredness, and chest discomfort	1; no symptoms to 7; symptoms experienced all the time	Sum of items from heart symptoms and tiredness subscales, divided by 7 Higher score = higher symptom burden	Paper and electronic versions of questionnaire available
						0%
						<5 min
**AFS/B** ^a^	2014^32^	NI	15 items; 2 domains: AF symptom severity and burden	**AFS**: 0; not at all to 5; severely **AFB**: 0; never in the last 3 months to 5; daily 1; 1–60 min to 4; all day Severity: 1; not bad to 4; severe Past 5 years event frequency: 0 to >3 times	Sum of items results in AF classifications: **AFS**: 0 to >20; asymptomatic (class I) to >30; severe (class IV) **AFB**: 0; none (class A) to 7–9 points; severe (class D) Higher score = higher AF burden.	Paper and electronic versions of questionnaire available
						**AFB**: 3% and **AFS**: 0%
						78% of responders took <5 min, 21% took 5–10 min, and 1% took >10 min
**AFSS** ^a^	1998^48^	Danish—2025^25^ Turkish—2014^33^ Indonesian—2023^34^	19 items; 3 domains: total AF burden, healthcare utilization, and symptom severity	5-point Likert scale.	Sum of items from 3 domains (0–35) Higher score = higher symptom burden	NR
						2.4–7%^33^
						8–8.8 min^34^ <5 min^33^
**ASTA** ^b^	Symptom severity scale—2012^44^ HRQoL scale—2015^40^	Polish—2019^41^ Brazilian—2020^42^ English—2022^43^ Indonesian version—2023^45^	22 items; 2 domains: symptom severity and HRQoL	0; no to 3; yes, a lot.	Sum of scores is transformed into a 0–100 scale (lowest score divided by score range × 100) Higher score = higher symptom and arrhythmia burden	Paper and electronic versions of questionnaire available
						3 in symptom scale, 2 in HRQoL scale^43^ 1 to 10 items, across 40 respondents^44^
						‘a few minutes’^40^
**AF6** ^a^	2009^36^	Responsiveness—2010^35^	6 items; 6 domains: breathing difficulties (during rest and exertion), AF-related daily activity limitations, AF-induced discomfort, tiredness, and worry or anxiety	0; absence of symptoms/no AF impact to 10; severe symptoms/high AF impact	Sum of scores from 6 domains (0 to 60) Higher score = higher symptom and AF impact burden	NR
						1 out of 666^36^
						‘easy to understand and to complete within an ordinary 20 minute patient visit’^36^
**SCL** ^b^	1996	Trial appraising measurement properties (SCL vs. SF-36 RFA cohort)—1996^38^ Trial appraising measurement properties (SCL vs. SF-36 PVI cohort) —2003^37^ Swedish—2020^39^	16 items; 2 domains: symptom severity and symptom frequency.	1; mild to 3; extreme (severity scale) and 1; never to 5; always (frequency scale)	Sum of items from the 2 domains scored separately: severity (0–48) and frequency (0–64) Higher score = higher symptom burden	NR
						Severity scale: 6% and frequency scale: 16%^37^
						NR

AF, atrial fibrillation; AF6, Atrial Fibrillation six-item questionnaire; AFS/B, Atrial Fibrillation Symptom and Burden measure; AFS, Atrial Fibrillation Symptom component of the Atrial Fibrillation Symptom and Burden questionnaire; AFS/B, Atrial Fibrillation Symptom and Burden questionnaire; AFSS, Atrial Fibrillation Symptom Severity Scale; AFSymp, Atrial Fibrillation Symptoms questionnaire; ASTA, Arrhythmia-Specific questionnaire in Tachycardia and Arrhythmia; HRQoL, health-related quality of life; NI, not identified (i.e. published validation studies were not identified); NR, not reported (i.e. data were not presented in the study); PROM, patient-reported outcome measure; PVI, pulmonary vein isolation; RFA, radiofrequency ablation; SCL, Symptom Checklist: Frequency and Severity Scale; SF-36, 36-item Short Form Survey; SF-12, 12-item Short Form Survey.

^a^Patient-reported outcome measure specific to individuals with atrial fibrillation.

^b^Patient-reported outcome measure specific to individuals with arrhythmias.

### Study quality and measurement properties

*Figure 3* illustrates the RoB study quality ratings and the measurement properties assigned to the included symptom severity PROMs, according to COSMIN’s definitions of good measurement properties.^14,17,22,23^ ASTA was the most validated PROM,^42–47^ while the SCL^37–39^ had the least evidence of validation across all measurement properties. Internal consistency and construct validity were the most evaluated measurement properties, with cross-cultural validity being assessed in just one PROM, the AFSS.^25^ Responsiveness was assessed only in the AF6.^35^

**Figure 3:**
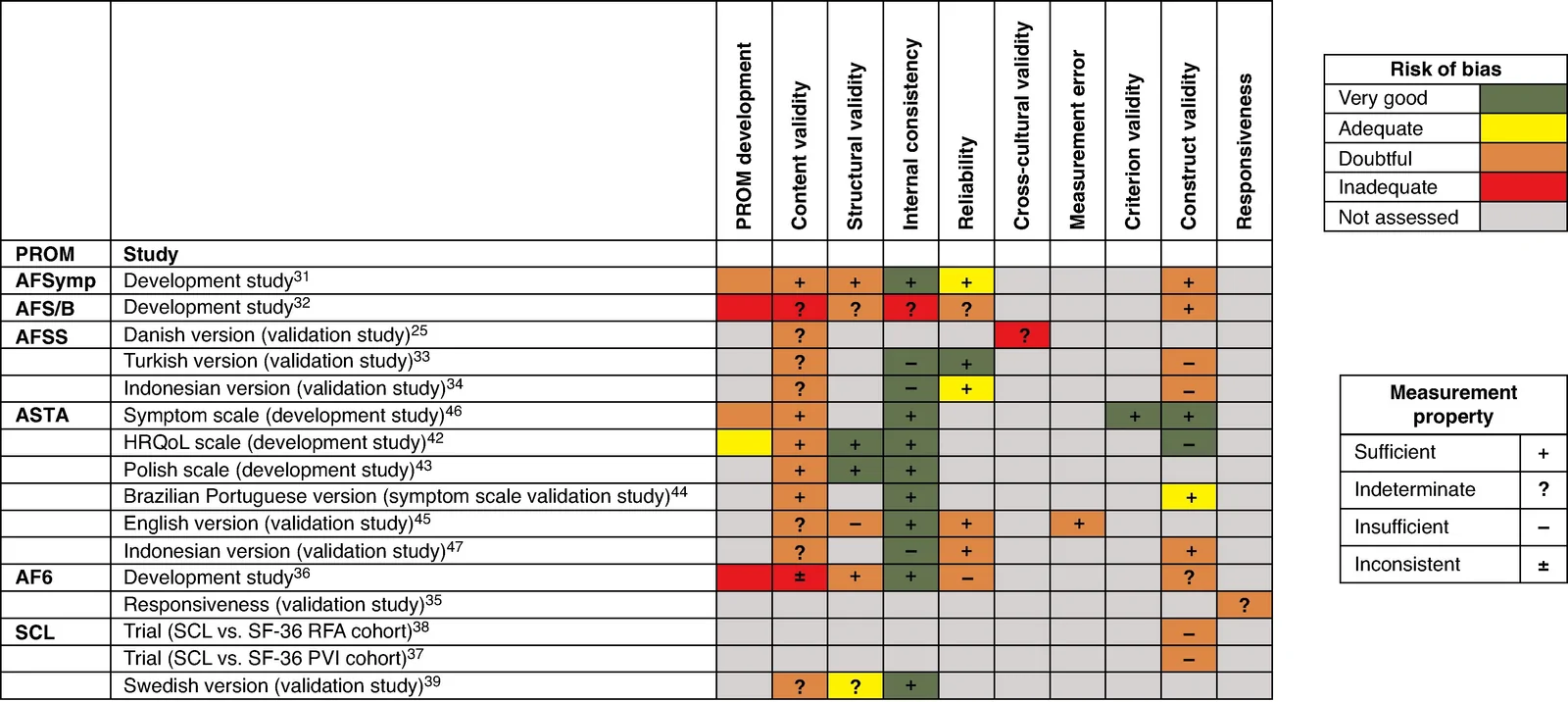
Risk of bias ratings and assessment of measurement properties of included symptom severity PROMs. AF6, Atrial Fibrillation six-item questionnaire; AFS/B, Atrial Fibrillation Symptom and Burden questionnaire; AFSS, Atrial Fibrillation Symptom Severity Scale; AFSymp, Atrial Fibrillation Symptoms questionnaire; ASTA, Arrhythmia-Specific questionnaire in Tachycardia; PROM, patient-reported outcome measure; PVI, pulmonary vein isolation; RFA, radiofrequency catheter ablation; SCL, Symptom Checklist: Frequency and Severity Scale; SF-36, 36-item Short Form Survey.

Doubtful RoB ratings were common due to methodological flaws, principally the ambiguity in details pertaining to the evaluation of a measurement property or PROM development study. Insufficient ratings (−) were evident for AF development studies (AF6^36^ and AFS/B^32^) as qualitative approaches were not employed during concept elicitation. Construct validity ratings were often classified as doubtful, as comparator PROMs typically did not contain symptom severity domains. Supplementary material online, *Table S4* summarizes justifications for each of the ratings.

### Synthesis of results

*Figure 4* illustrates the synthesized evaluation of the six symptom severity PROMs using the COSMIN modified GRADE approach.^14,17,22,23^ Due to the limited number of methodological studies for each PROM, many measurement properties were often rated as indeterminate. This, combined with inconsistencies in RoB and measurement property ratings across the pooled individual methodological studies, resulted in downgrading of evidence and generally low confidence in the quality of these studies. Of all validation studies, AFSS^33,34^ was the only PROM to demonstrate insufficient measurement properties for more than one criterion (internal consistency and construct validity). Reviewers’ ratings determined content validity for AFS/B,^32^ AFSS,^25,33,34^ and SCL^37–39^ due to methodological studies lacking sufficient data for assessment^22^ (see Supplementary material online, *Table S5*).

**Figure 4:**
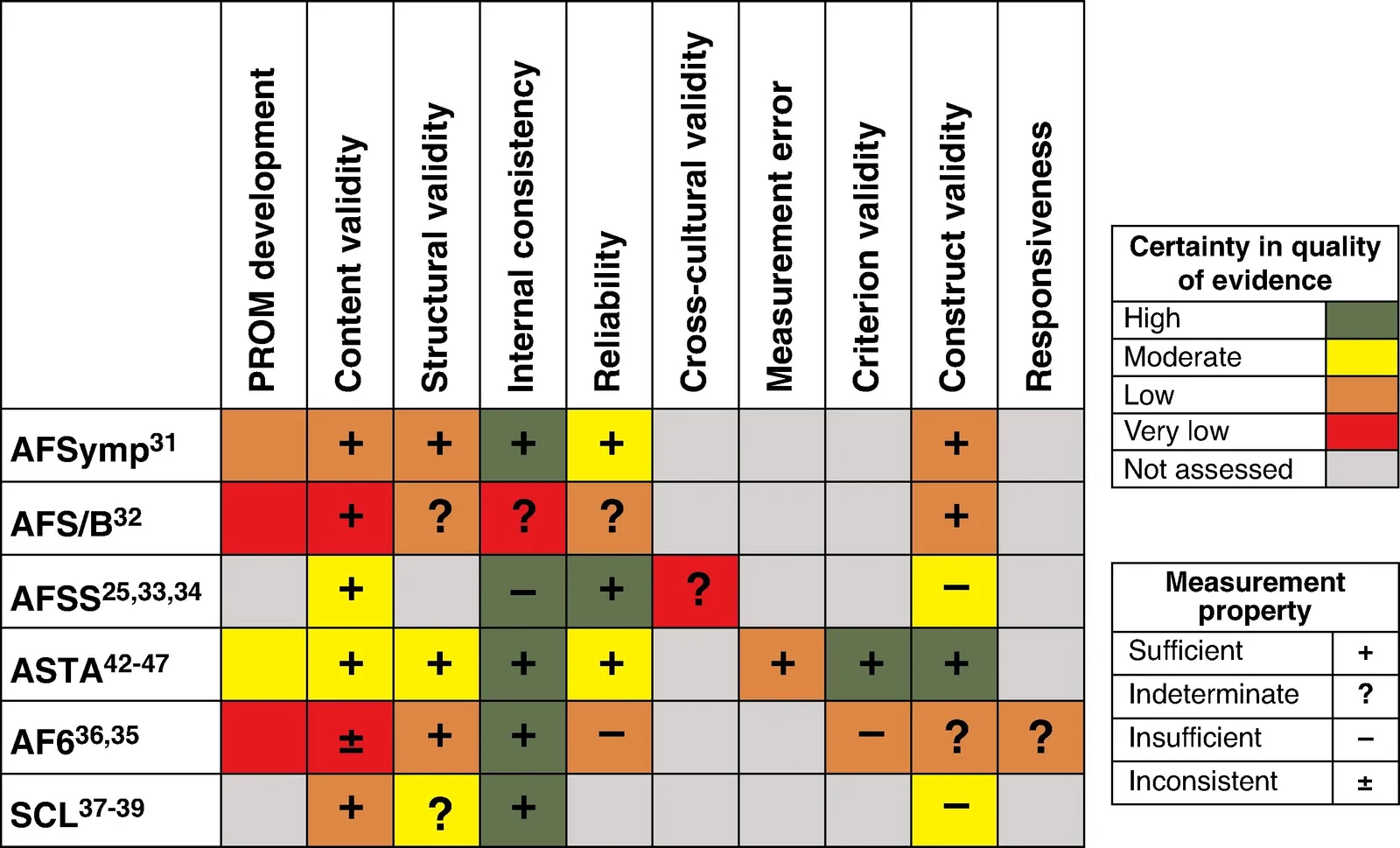
Modified Grading of Recommendations, Assessment Development and Evaluation (GRADE) ratings of the six included symptom severity patient-reported outcome measures. AF6, Atrial Fibrillation six-item questionnaire; AFS/B, Atrial Fibrillation Symptom and Burden questionnaire; AFSS, Atrial Fibrillation Symptom Severity Scale; AFSymp, Atrial Fibrillation Symptoms questionnaire; ASTA, Arrhythmia-Specific questionnaire in Tachycardia; PROM, patient-reported outcome measure; SCL, Symptom Checklist: Frequency and Severity Scale.

Based on COSMIN guidelines, the AFSymp,^31^ ASTA,^42–47^ and SCL^37–39^ had proof of sufficient content validity and at least low-quality evidence for sufficient internal consistency and can be proposed for use.^14,17^ Overall, AFSymp^31^ and ASTA^42–47^ were found to have the most robust measurement properties, with promising feasibility and interpretability factors, demonstrated by low missing item scores, short completion times, and availability in electronic and paper formats (*Table 2*). ASTA^42–47^ was assessed as having the highest certainty in evidence quality across assessed psychometric properties.

## Discussion

One hundred and one validated PROMs assessing the seven most important PROs for patients with AF^7^ were identified. HRQoL was the most frequently assessed PRO and most often evaluated using the SF-36.^29^

Seven PROMs assessing symptom severity in patients with AF were identified, with six (AFSymp,^31^ AFS/B,^32^ AFSS,^25,33,34^ ASTA,^42–47^ AF6,^35,36^ and SCL^37–39^) assessed for their measurement properties and appropriateness for clinical use. ASTA^42–47^ was assessed as having the most robust measurement properties, with the most certainty in the quality of evidence. AFSymp^31^ showed sufficient measurement properties for internal consistency, reliability, and responsiveness, but received an indeterminate rating for structural validity. Although Bentler’s Comparative Fit Index (CFI) was 1.0 for chest discomfort and 0.994 for the tiredness domains, warranting a rating of sufficient; the CFI for heart symptoms was 0.942, just below the threshold of >0.95 per COSMIN standards.^14,31^ Therefore, the utility of the AFSymp is currently constrained by reliance on a single development study^31^ and the lack of further validation studies.

Consistent with other reviews focusing on AF-specific QoL PROMs using COSMIN methodology,^12,13^ assessments of measurement error, responsiveness, and cross-cultural validity in symptom severity PROMs were lacking. The AF6^35^ and AFSS^25^ were the only PROMs to assess responsiveness and cultural validity, respectively. Measurement error was assessed only by ASTA.^45^ Developers of the AFSymp^31^ acknowledged the need for further validation of its responsiveness. The AFS/B^32^ study reported plans to evaluate its responsiveness in a catheter ablation cohort; however, this study was not identified in the database search. All PROMs should undergo validation using interventions prior to their implementation in AF care to evaluate their sensitivity in detecting meaningful clinical changes.

### Strengths and limitations

The main strength of this review is its methodological rigour. By employing six databases (including grey literature sources) and 98% sensitive search filters, the identification of eligible articles was maximized to comprehensively identify all relevant PROMs. No studies were excluded based on language, enhancing the cultural validity of this review and allowing identification of PROMs used across diverse countries and languages. Additionally, study screening, psychometric property evaluation, and quality of evidence assessments were conducted independently and in duplicate, with oversight by a third reviewer to reach consensus, therefore mitigating judgement bias and the potential for inaccuracy, which enhanced the reliability of findings.

This systematic review is novel, as, to our knowledge, no other review has comprehensively identified PROMs used to assess PROs deemed the most important to adults with AF.^7^ A database analysis found that in over 1700 trials involving AF patients, only 14% investigated PROs,^49^ demonstrating the additive value of this review in narrowing a critical research gap.

Individuals with AF often seek treatment due to the impact of symptoms on their daily lives, which may lead to hospitalization.^50^ A retrospective analysis found that 34% of patients with AF or atrial flutter were hospitalized within 12 months of diagnosis.^51^ Routine assessment of symptom severity using a validated PROM may help to capture problems such as psychosocial factors including anxiety, depression, and QoL impacts, which may influence symptom perception in AF, and enable early intervention to address psychosocial burden, which must be considered in holistic patient-centred AF care, which may ultimately avert hospitalization.^2,10,11,52^ Monitoring symptoms through PROMs during visit-to-visit consultations following the initiation of drugs to control heart rate or rhythm may guide adjustments to ascertain the patient’s optimal dosage regimen,^53^ or result in better adherence (through alleviation of symptoms)^10,11^ and lower healthcare resource utilization (due to less AF symptomatic burden). Psychosocial factors could also be considered within AF care through the incorporation of PROMs as tools to support the holistic management of AF.^3,52^

Adherence to the rigorous standards of the COSMIN methodology for evaluating measurement properties enhances the internal validity of the results of this review.^14^ However, a notable limitation is that COSMIN requires full-text methodological studies covering all measurement properties for a PROM to be deemed robust with sufficient psychometric properties, which were not available for two of the identified symptom severity PROMs. The full-text methodological studies for the MAFSI and LARQ PROMs were not published. Therefore, results should be interpreted with caution, as PROMs with ratings below ‘very good’ may have stronger psychometric properties, pending the publication of additional studies. Additionally, many of the PROMs identified were developed and validated before the development of the COSMIN methodology and therefore did not include robust assessment of all measurement properties in their studies.^35,36,54^ Due to the volume of PROMs identified for each PRO, it was not possible to evaluate the measurement properties of all PROs of interest as originally planned; this is a limitation of our study; however, subsequent studies could address this.

### Recommendations for further research

To enable routine integration of PROMs into routine AF management, further validation is necessary; none of the six symptom severity PROMs evaluated demonstrated validation across all measurement properties. Of note, responsiveness, interpretability, and feasibility aspects must be considered in future validation studies to support their use in clinical practice. Floor effects, which occur when respondents score at the lower end of a measure, may indicate the PROM’s inability to detect higher symptom severity or distinguish between varying degrees of symptom severity, which is important as the symptomatic experiences of AF individuals are variable.^2,14,55^

While measurement properties are important, future PROM validations should also address factors of interpretability, especially as clinicians’ concerns about interpreting PROM scores may be a key reason why PROMs are not yet routinely used in AF care. For example, a clinician may favour a PROM with a simple scoring algorithm, such as summing responses, due to the time constraints of medical consultations, making complicated scoring methods inappropriate. Training on the use and interpretation of PROMs for healthcare professionals may be required to facilitate appropriate implementation.

Other feasibility factors are also of importance. For example, electronic completion of the PROM in the AFSymp^31^ study resulted in no missing data because the questionnaire could not be submitted until all items were complete. However, electronic administration may not be possible in all settings and may limit participation of those who are not digitally literate or do not have access to a digital device. Hussein *et al.* found that there was a significant reduction in hospitalizations and emergency visits for people with AF whose routine care was augmented with automated PRO reporting, compared to those receiving standard care.^56^ Clinicians could improve efficiency by having AF patients complete PROMs prior to consultations, allowing for more focused in-person consultations.^57^ As healthcare systems evolve, future validations should consider the adaptability of PROM formats and the development of automated platforms. However, this approach requires careful consideration, particularly regarding the need for training of both healthcare providers and respondents. Women were under-represented in the included studies, with 88% of studies comprising ≤49% females, and most participants were of White ethnicity.

Future methodological studies should ensure equality and diversity in their cohorts to ensure generalizable results with high external validity.

A pragmatic decision was taken to focus on symptom severity PROMs, but PROMs identified for measuring the other PROs could also be appraised. Additionally, this review identified further AFEQT validation studies,^58,59^ and a novel QoL measure, the Atrial Fibrillation Impact questionnaire (AFImpact),^60,61^ which were not covered in previous reviews of AF QoL measures and require evaluation.^12,13^

### Implications for clinical practice

The EHRA and international sister societies advocate for the routine inclusion of PROMs in AF care to inform clinical management, but historically, implementation has been limited due to the lack of evidence on the robustness of these measures. However, the results of this review, identifying all available validated PROMs for the seven PROs considered important for patients with AF, together with evidence on the psychometric properties of the symptom severity scales and suggestions for which PROM to use, may support routine collection of symptom severity and other PROs in patients with AF.

## Conclusion

One hundred and one validated PROMs available to assess PROs recognized as important to individuals with AF were identified. Of the six PROMs evaluating symptom severity assessed for psychometric properties, the ASTA demonstrated the most robust performance. Future PROM development and validation should adhere to COSMIN methodology to ensure rigorous and reliable measurement properties.

## Supplementary Material

euag252_Supplementary_Data

## Data Availability

All the data included in this manuscript are from published studies and available in the original publications.
