# Supplementary material for: Patient-reported outcome measures for atrial fibrillation: a systematic review and evaluation of psychometric properties of symptom severity measures
Source: Europace. 2026 Sep 8;28(10):euag252. doi: 10.1093/europace/euag252 (PMC13678206; doi:10.1093/europace/euag252)
Supplement: Supplementary file 1 [file euag252_supplementary_data.zip › Supplementary Materials_Sept2026.docx]

**Supplementary Appendix A** **- Supplementary methods (search strategies)**

**PubMed database search strategy**

("atrial fibrillation"[MeSH]) OR ("atrial fibrillation*"[tiab] OR "atrium fibrillation*"[tiab] OR "auricular fibrillation*"[tiab])

*AND*

(HR-PRO[tiab] OR HRPRO[tiab] OR HRQL[tiab] OR HRQoL[tiab] OR QL[tiab] OR QoL[tiab] OR "quality of life"[tw] OR "life quality"[tw] OR "health index*"[tiab] OR "health indices"[tiab] OR "health profile*"[tiab] OR "health status"[tw] OR ((patient[tiab] OR self[tiab] OR child[tiab] OR parent[tiab] OR carer[tiab] OR proxy[tiab]) AND ((report[tiab] OR reported[tiab] OR reporting[tiab]) OR (rated[tiab] OR rating[tiab] OR ratings[tiab]) OR based[tiab] OR (assessed[tiab] OR assessment[tiab] OR assessments[tiab]))) OR ((disability[tiab] OR function[tiab] OR functional[tiab] OR functions[tiab] OR subjective[tiab] OR utility[tiab] OR utilities[tiab] OR wellbeing[tiab] OR "well being"[tiab]) AND (index[tiab] OR indices[tiab] OR instrument[tiab] OR instruments[tiab] OR measure[tiab] OR measures[tiab] OR questionnaire[tiab] OR questionnaires[tiab] OR profile[tiab] OR profiles[tiab] OR scale[tiab] OR scales[tiab] OR score[tiab] OR scores[tiab] OR status[tiab] OR survey[tiab] OR surveys[tiab])))

*AND*

((instrumentation[sh] OR methods[sh] OR "validation study"[pt] OR "Comparative Study"[pt] OR "psychometrics"[MeSH] OR psychometr*[tiab] OR clinimetr*[tw] OR clinometr*[tw] OR "outcome assessment, health care"[MeSH] OR "outcome assessment"[tiab] OR "outcome measure*"[tw] OR "observer variation"[MeSH] OR "observer variation"[tiab] OR "Health Status Indicators"[Mesh] OR "reproducibility of results"[MeSH] OR reproducib*[tiab] OR "discriminant analysis"[MeSH] OR reliab*[tiab] OR unreliab*[tiab] OR valid*[tiab] OR "coefficient of variation"[tiab] OR coefficient[tiab] OR homogeneity[tiab] OR homogeneous[tiab] OR "internal consistency"[tiab] OR (cronbach*[tiab] AND (alpha[tiab] OR alphas[tiab])) OR (item[tiab] AND (correlation*[tiab] OR selection*[tiab] OR reduction*[tiab])) OR agreement[tw] OR precision[tw] OR imprecision[tw] OR "precise values"[tw] OR test-retest[tiab] OR (test[tiab] AND retest[tiab]) OR (reliab*[tiab] AND (test[tiab] OR retest[tiab])) OR stability[tiab] OR interrater[tiab] OR inter-rater[tiab] OR intrarater[tiab] OR intra-rater[tiab] OR intertester[tiab] OR inter-tester[tiab] OR intratester[tiab] OR intra-tester[tiab] OR interobserver[tiab] OR inter-observer[tiab] OR intraobserver[tiab] OR intra-observer[tiab] OR intertechnician[tiab] OR inter-technician[tiab] OR intratechnician[tiab] OR intra-technician[tiab] OR interexaminer[tiab] OR inter-examiner[tiab] OR intraexaminer[tiab] OR intra-examiner[tiab] OR interassay[tiab] OR inter-assay[tiab] OR intraassay[tiab] OR intra-assay[tiab] OR interindividual[tiab] OR inter-individual[tiab] OR intraindividual[tiab] OR intra-individual[tiab] OR interparticipant[tiab] OR inter-participant[tiab] OR intraparticipant[tiab] OR intra-participant[tiab] OR kappa[tiab] OR kappa’s[tiab] OR kappas[tiab] OR repeatab*[tw] OR ((replicab*[tw] OR repeated[tw]) AND (measure[tw] OR measures[tw] OR findings[tw] OR result[tw] OR results[tw] OR test[tw] OR tests[tw])) OR generaliza*[tiab] OR generalisa*[tiab] OR concordance[tiab] OR (intraclass[tiab] AND correlation*[tiab]) OR discriminative[tiab] OR "known group"[tiab] OR "factor analysis"[tiab] OR "factor analyses"[tiab] OR "factor structure"[tiab] OR "factor structures"[tiab] OR dimension*[tiab] OR subscale*[tiab] OR (multitrait[tiab] AND scaling[tiab] AND (analysis[tiab] OR analyses[tiab])) OR "item discriminant"[tiab] OR "interscale correlation*"[tiab] OR error[tiab] OR errors[tiab] OR "individual variability"[tiab] OR "interval variability"[tiab] OR "rate variability"[tiab] OR (variability[tiab] AND (analysis[tiab] OR values[tiab])) OR (uncertainty[tiab] AND (measurement[tiab] OR measuring[tiab])) OR "standard error of measurement"[tiab] OR sensitiv*[tiab] OR responsive*[tiab] OR (limit[tiab] AND detection[tiab]) OR "minimal detectable concentration"[tiab] OR interpretab*[tiab] OR ((minimal[tiab] OR minimally[tiab] OR clinical[tiab] OR clinically[tiab]) AND (important[tiab] OR significant[tiab] OR detectable[tiab]) AND (change[tiab] OR difference[tiab])) OR (small*[tiab] AND (real[tiab] OR detectable[tiab]) AND (change[tiab] OR difference[tiab])) OR "meaningful change"[tiab] OR "ceiling effect"[tiab] OR "floor effect"[tiab] OR "Item response model"[tiab] OR IRT[tiab] OR Rasch[tiab] OR "Differential item functioning"[tiab] OR DIF[tiab] OR "computer adaptive testing"[tiab] OR "item bank"[tiab] OR "cross-cultural equivalence"[tiab]))

*NOT*

("address"[pt] OR "biography"[pt] OR "case reports"[pt] OR "comment"[pt] OR "directory"[pt] OR "editorial"[pt] OR "festschrift"[pt] OR "interview"[pt] OR "lecture"[pt] OR "legal case"[pt] OR "legislation"[pt] OR "letter"[pt] OR "news"[pt] OR "newspaper article"[pt] OR "patient education handout"[pt] OR "popular work"[pt] OR "congress"[pt] OR "consensus development conference"[pt] OR "consensus development conference, nih"[pt] OR "practice guideline"[pt]) OR ("animals"[MeSH] NOT "humans"[MeSH])

**MEDLINE (via OVID interface) database search strategy**

Atrial Fibrillation/ or (atrial fibrillation* or atrium fibrillation* or auricular fibrillation*).tw.

*AND*

(HR-PRO or HRPRO or HRQL or HRQoL or QL or QoL).tw. or quality of life.mp. or life quality.mp. or (health index* or health indices or health profile*).tw. or health status.mp. or ((patient or self or child or parent or carer or proxy) adj (appraisal* or appraised or report or reported or reporting or rated or rating* or based or assessed or assessment*)).tw. or ((disability or function or functional or functions or subjective or utility or utilities or wellbeing or well being) adj2 (index or indices or instrument or instruments or measure or measures or questionnaire or questionnaires or profile or profiles or scale or scales or score or scores or status or survey or surveys)).tw.

*AND*

instrumentation/ or methods/ or Outcome Assessment, Health Care/ or outcome assessment.tw. or outcome measure*.mp. or Health Status Indicators/ or Validation Studies.pt. or Comparative Study.pt. or psychometrics/ or psychometr*.tw. or clinimetr*.mp. or clinometr*.mp. or observer variation/ or observer variation.tw. or reproducibility of results/ or reproducib*.tw. or discriminant analysis/ or reliab*.tw. or unreliab*.tw. or valid*.tw. or coefficient of variation.tw. or coefficient.tw. or homogeneity.tw. or homogeneous.tw. or internal consistency.tw. or (cronbach* adj3 (alpha or alphas)).tw. or (item adj3 (correlation* or selection* or reduction*)).tw. or agreement.mp. or precision.mp. or imprecision.mp. or precise values.mp. or test-retest.tw. or (test adj3 retest).tw. or (reliab* adj3 (test or retest)).tw. or stability.tw. or interrater.tw. or inter-rater.tw. or intrarater.tw. or intra-rater.tw. or intertester.tw. or inter-tester.tw. or intratester.tw. or intra-tester.tw. or interobserver.tw. or inter-observer.tw. or intraobserver.tw. or intra-observer.tw. or intertechnician.tw. or inter-technician.tw. or intratechnician.tw. or intra-technician.tw. or interexaminer.tw. or inter-examiner.tw. or intraexaminer.tw. or intra-examiner.tw. or interassay.tw. or inter-assay.tw. or intraassay.tw. or intra-assay.tw. or interindividual.tw. or inter-individual.tw. or intraindividual.tw. or intra-individual.tw. or interparticipant.tw. or inter-participant.tw. or intraparticipant.tw. or intra-participant.tw. or kappa.tw. or kappa's.tw. or kappas.tw. or repeatab*.mp. or ((replicab* or repeated) adj3 (measure or measures or findings or result or results or test or tests)).mp. or generaliza*.tw. or generalisa*.tw. or concordance.tw. or (intraclass adj3 correlation*).tw. or discriminative.tw. or known group.tw. or factor analysis.tw. or factor analyses.tw. or factor structure.tw. or factor structures.tw. or dimension*.tw. or subscale*.tw. or (multitrait adj3 scaling adj3 (analysis or analyses)).tw. or item discriminant.tw. or interscale correlation*.tw. or error.tw. or errors.tw. or individual variability.tw. or interval variability.tw. or rate variability.tw. or (variability adj3 (analysis or values)).tw. or (uncertainty adj3 (measurement or measuring)).tw. or standard error of measurement.tw. or sensitiv*.tw. or responsive*.tw. or (limit adj3 detection).tw. or minimal detectable concentration.tw. or interpretab*.tw. or ((minimal or minimally or clinical or clinically) adj3 (important or significant or detectable) adj3 (change or difference)).tw. or (small* adj3 (real or detectable) adj3 (change or difference)).tw. or meaningful change.tw. or ceiling effect.tw. or floor effect.tw. or Item response model.tw. or IRT.tw. or Rasch.tw. or Differential item functioning.tw. or DIF.tw. or computer adaptive testing.tw. or item bank.tw. or cross-cultural equivalence.tw.

*NOT*

(address or biography or case reports or comment or directory or editorial or festschrift or interview or lecture or legal case or legislation or letter or news or newspaper article or patient education handout or popular work or congress or consensus development conference or consensus development conference nih or practice guideline).pt.

**Cumulative Index to Nursing and Allied Health Literature (CINAHL; via EBSCOhost interface) database search strategy**

(MH "Atrial Fibrillation") OR (TI "atrial fibrillation*" OR AB "atrial fibrillation*") OR (TI "atrium fibrillation*" OR AB "atrium fibrillation*") OR (TI "auricular fibrillation*" OR AB "auricular fibrillation*")

*AND*

TI ( (hrql OR hrqol OR ql OR qol OR "quality of life" OR "life quality" OR "health index*" OR "health indices" OR "health profile*" OR "health status" or ((patient OR carer OR proxy) AND (report OR reported OR reporting OR rated OR rating OR ratings OR based OR assessed OR assessment OR assessments)) OR ((disability OR function OR functional OR functions OR subjective OR utility OR utilities OR wellbeing OR "well being") AND (outcome OR outcomes OR index OR indices OR instrument OR instruments OR measure OR measures OR questionnaire OR questionnaires OR profile OR profiles OR scale OR scales OR score OR scores OR status OR surveys))) ) OR AB ( (hrql OR hrqol OR ql OR qol OR "quality of life" OR "life quality" OR "health index*" OR "health indices" OR "health profile*" OR "health status" or ((patient OR carer OR proxy) AND (report OR reported OR reporting OR rated OR rating OR ratings OR based OR assessed OR assessment OR assessments)) OR ((disability OR function OR functional OR functions OR subjective OR utility OR utilities OR wellbeing OR "well being") AND (outcome OR outcomes OR index OR indices OR instrument OR instruments OR measure OR measures OR questionnaire OR questionnaires OR profile OR profiles OR scale OR scales OR score OR scores OR status OR surveys))) )

*AND*

(("Instrumentation" OR "Methods" OR (PT "validation study") OR (PT "Comparative Study") OR (MH "Psychometrics") OR (TI psychometr* OR AB psychometr*) OR clinimetr* OR clinometr* OR (MH Outcome assessment") OR (TI "outcome assessment" OR AB "outcome assessment") OR "outcome measure*" OR ("observer variation") OR (MH "Health Status Indicators") OR (MH "Reproducibility of Results") OR (TI reproducib* OR AB reproducib*) OR (MH "Discriminant Analysis") OR (TI reliab* OR AB reliab*) OR (TI unreliab* OR AB unreliab*) OR (TI valid* OR AB valid*) OR (TI "coefficient of variation" OR AB "coefficient of variation") OR (TI coefficient OR AB coefficient) OR (TI homogeneity OR AB homogeneity) OR (TI homogeneous OR AB homogeneous) OR (TI "internal consistency" OR AB "internal consistency") OR ((TI cronbach* OR AB cronbach*) AND ((TI alpha OR AB alpha) OR (TI alphas OR AB alphas))) OR ((TI item OR AB item) AND ((TI correlation* OR AB correlation*) OR (TI selection* OR AB selection*) OR (TI reduction* OR AB reduction*))) OR agreement OR precision OR imprecision OR "precise values" OR (TI test-retest OR AB test-retest) OR ((TI test OR AB test) AND (TI retest OR AB retest)) OR ((TI reliab* OR AB reliab*) AND ((TI test OR AB test) OR (TI retest OR AB retest))) OR (TI stability OR AB stability) OR (TI interrater OR AB interrater) OR (TI inter-rater OR AB inter-rater) OR (TI intrarater OR AB intrarater) OR (TI intra-rater OR AB intra-rater) OR (TI intertester OR AB intertester) OR (TI inter-tester OR AB inter-tester) OR (TI intratester OR AB intratester) OR (TI intra-tester OR AB intra-tester) OR (TI interobserver OR AB interobserver) OR (TI inter-observer OR AB inter-observer) OR (TI intraobserver OR AB intraobserver) OR (TI intra-observer OR AB intra-observer) OR (TI intertechnician OR AB intertechnician) OR (TI inter-technician OR AB inter-technician) OR (TI intratechnician OR AB intratechnician) OR (TI intra-technician OR AB intra-technician) OR (TI interexaminer OR AB interexaminer) OR (TI inter-examiner OR AB inter-examiner) OR (TI intraexaminer OR AB intraexaminer) OR (TI intra-examiner OR AB intra-examiner) OR (TI interassay OR AB interassay) OR (TI inter-assay OR AB inter-assay) OR (TI intraassay OR AB intraassay) OR (TI intra-assay OR AB intra-assay) OR (TI interindividual OR AB interindividual) OR (TI inter-individual OR AB inter-individual) OR (TI intraindividual OR AB intraindividual) OR (TI intra-individual OR AB intra-individual) OR (TI interparticipant OR AB interparticipant) OR (TI inter-participant OR AB inter-participant) OR (TI intraparticipant OR AB intraparticipant) OR (TI intra-participant OR AB intra-participant) OR (TI kappa OR AB kappa) OR (TI kappa’s OR AB kappa’s) OR (TI kappas OR AB kappas) OR repeatab* OR ((replicab* OR repeated) AND (measure OR measures OR findings OR result OR results OR test OR tests)) OR (TI generaliza* OR AB generaliza*) OR (TI generalisa* OR AB generalisa*) OR (TI concordance OR AB concordance) OR ((TI intraclass OR AB intraclass) AND (TI correlation* OR AB correlation*)) OR (TI discriminative OR AB discriminative) OR (TI "known group" OR AB "known group") OR (TI "factor analysis" OR AB "factor analysis") OR (TI "factor analyses" OR AB "factor analyses") OR (TI "factor structure" OR AB "factor structure") OR (TI "factor structures" OR AB "factor structures") OR (TI dimension* OR AB dimension*) OR (TI subscale* OR AB subscale*) OR ((TI multitrait OR AB multitrait) AND (TI scaling OR AB scaling) AND ((TI analysis OR AB analysis) OR (TI analyses OR AB analyses))) OR (TI "item discriminant" OR AB "item discriminant") OR (TI "interscale correlation*" OR AB "interscale correlation*") OR (TI error OR AB error) OR (TI errors OR AB errors) OR (TI "individual variability" OR AB "individual variability") OR (TI "interval variability" OR AB "interval variability") OR (TI "rate variability" OR AB "rate variability") OR ((TI variability OR AB variability) AND ((TI analysis OR AB analysis) OR (TI values OR AB values))) OR ((TI uncertainty OR AB uncertainty) AND ((TI measurement OR AB measurement) OR (TI measuring OR AB measuring))) OR (TI "standard error of measurement" OR AB "standard error of measurement") OR (TI sensitiv* OR AB sensitiv*) OR (TI responsive* OR AB responsive*) OR ((TI limit OR AB limit) AND (TI detection OR AB detection)) OR (TI "minimal detectable concentration" OR AB "minimal detectable concentration") OR (TI interpretab* OR AB interpretab*) OR (((TI minimal OR AB minimal) OR (TI minimally OR AB minimally) OR (TI clinical OR AB clinical) OR (TI clinically OR AB clinically)) AND ((TI important OR AB important) OR (TI significant OR AB significant) OR (TI detectable OR AB detectable)) AND ((TI change OR AB change) OR (TI difference OR AB difference))) OR ((TI small* OR AB small*) AND ((TI real OR AB real) OR (TI detectable OR AB detectable)) AND ((TI change OR AB change) OR (TI difference OR AB difference))) OR (TI "meaningful change" OR AB "meaningful change") OR (TI "ceiling effect" OR AB "ceiling effect") OR (TI "floor effect" OR AB "floor effect") OR (TI "Item response model" OR AB "Item response model") OR (TI IRT OR AB IRT) OR (TI Rasch OR AB Rasch) OR (TI "Differential item functioning" OR AB "Differential item functioning") OR (TI DIF OR AB DIF) OR (TI "computer adaptive testing" OR AB "computer adaptive testing") OR (TI "item bank" OR AB "item bank") OR (TI "cross-cultural equivalence" OR AB "cross-cultural equivalence")))

*NOT*

((PT address) OR (PT biography) OR (PT "case reports") OR (PT comment) OR (PT directory) OR (PT editorial) OR (PT festschrift) OR (PT interview) OR (PT lecture) OR (PT "legal case") OR (PT legislation) OR (PT letter) OR (PT news) OR (PT "newspaper article") OR (PT "patient education handout") OR (PT "popular work") OR (PT congress) OR (PT "consensus development conference") OR (PT "consensus development conference, nih") OR (PT "practice guideline")) OR (MH "Animals" NOT MH "Human")

**APA PsycINFO (via EBSCOhost interface) database search strategy**

(DE "Heart Fibrillation") OR (TI "atrial fibrillation*" OR AB "atrial fibrillation*") OR (TI "atrium fibrillation*" OR AB "atrium fibrillation*") OR (TI "auricular fibrillation*" OR AB "auricular fibrillation*")

*AND*

((TI HR-PRO OR AB HR-PRO) OR (TI HRPRO OR AB HRPRO) OR (TI HRQL OR AB HRQL) OR (TI HRQoL OR AB HRQoL) OR (TI QL OR AB QL) OR (TI QoL OR AB QoL) OR "quality of life" OR "life quality" OR (TI "health index*" OR AB "health index*") OR (TI "health indices" OR AB "health indices") OR (TI "health profile*" OR AB "health profile*") OR "health status" OR (((TI patient OR AB patient) OR (TI self OR AB self) OR (TI child OR AB child) OR (TI parent OR AB parent) OR (TI carer OR AB carer) OR (TI proxy OR AB proxy)) AND (((TI report OR AB report) OR (TI reported OR AB reported) OR (TI reporting OR AB reporting)) OR ((TI rated OR AB rated) OR (TI rating OR AB rating) OR (TI ratings OR AB ratings)) OR (TI based OR AB based) OR ((TI assessed OR AB assessed) OR (TI assessment OR AB assessment) OR (TI assessments OR AB assessments)))) OR (((TI disability OR AB disability) OR (TI function OR AB function) OR (TI functional OR AB functional) OR (TI functions OR AB functions) OR (TI subjective OR AB subjective) OR (TI utility OR AB utility) OR (TI utilities OR AB utilities) OR (TI wellbeing OR AB wellbeing) OR (TI "well being" OR AB "well being")) AND ((TI index OR AB index) OR (TI indices OR AB indices) OR (TI instrument OR AB instrument) OR (TI instruments OR AB instruments) OR (TI measure OR AB measure) OR (TI measures OR AB measures) OR (TI questionnaire OR AB questionnaire) OR (TI questionnaires OR AB questionnaires) OR (TI profile OR AB profile) OR (TI profiles OR AB profiles) OR (TI scale OR AB scale) OR (TI scales OR AB scales) OR (TI score OR AB score) OR (TI scores OR AB scores) OR (TI status OR AB status) OR (TI survey OR AB survey) OR (TI surveys OR AB surveys))))

*AND*

(("Instrumentation" OR "Methods" OR (PT "validation study") OR (PT "Comparative Study") OR (DE "Psychometrics") OR (TI psychometr* OR AB psychometr*) OR clinimetr* OR clinometr* OR ("outcome assessment, health care") OR (TI "outcome assessment" OR AB "outcome assessment") OR "outcome measure*" OR ("observer variation") OR ("Health Status Indicators") OR ("reproducibility of results") OR (TI reproducib* OR AB reproducib*) OR ("discriminant analysis") OR (TI reliab* OR AB reliab*) OR (TI unreliab* OR AB unreliab*) OR (TI valid* OR AB valid*) OR (TI "coefficient of variation" OR AB "coefficient of variation") OR (TI coefficient OR AB coefficient) OR (TI homogeneity OR AB homogeneity) OR (TI homogeneous OR AB homogeneous) OR (TI "internal consistency" OR AB "internal consistency") OR ((TI cronbach* OR AB cronbach*) AND ((TI alpha OR AB alpha) OR (TI alphas OR AB alphas))) OR ((TI item OR AB item) AND ((TI correlation* OR AB correlation*) OR (TI selection* OR AB selection*) OR (TI reduction* OR AB reduction*))) OR agreement OR precision OR imprecision OR "precise values" OR (TI test-retest OR AB test-retest) OR ((TI test OR AB test) AND (TI retest OR AB retest)) OR ((TI reliab* OR AB reliab*) AND ((TI test OR AB test) OR (TI retest OR AB retest))) OR (TI stability OR AB stability) OR (TI interrater OR AB interrater) OR (TI inter-rater OR AB inter-rater) OR (TI intrarater OR AB intrarater) OR (TI intra-rater OR AB intra-rater) OR (TI intertester OR AB intertester) OR (TI inter-tester OR AB inter-tester) OR (TI intratester OR AB intratester) OR (TI intra-tester OR AB intra-tester) OR (TI interobserver OR AB interobserver) OR (TI inter-observer OR AB inter-observer) OR (TI intraobserver OR AB intraobserver) OR (TI intra-observer OR AB intra-observer) OR (TI intertechnician OR AB intertechnician) OR (TI inter-technician OR AB inter-technician) OR (TI intratechnician OR AB intratechnician) OR (TI intra-technician OR AB intra-technician) OR (TI interexaminer OR AB interexaminer) OR (TI inter-examiner OR AB inter-examiner) OR (TI intraexaminer OR AB intraexaminer) OR (TI intra-examiner OR AB intra-examiner) OR (TI interassay OR AB interassay) OR (TI inter-assay OR AB inter-assay) OR (TI intraassay OR AB intraassay) OR (TI intra-assay OR AB intra-assay) OR (TI interindividual OR AB interindividual) OR (TI inter-individual OR AB inter-individual) OR (TI intraindividual OR AB intraindividual) OR (TI intra-individual OR AB intra-individual) OR (TI interparticipant OR AB interparticipant) OR (TI inter-participant OR AB inter-participant) OR (TI intraparticipant OR AB intraparticipant) OR (TI intra-participant OR AB intra-participant) OR (TI kappa OR AB kappa) OR (TI kappa’s OR AB kappa’s) OR (TI kappas OR AB kappas) OR repeatab* OR ((replicab* OR repeated) AND (measure OR measures OR findings OR result OR results OR test OR tests)) OR (TI generaliza* OR AB generaliza*) OR (TI generalisa* OR AB generalisa*) OR (TI concordance OR AB concordance) OR ((TI intraclass OR AB intraclass) AND (TI correlation* OR AB correlation*)) OR (TI discriminative OR AB discriminative) OR (TI "known group" OR AB "known group") OR (TI "factor analysis" OR AB "factor analysis") OR (TI "factor analyses" OR AB "factor analyses") OR (TI "factor structure" OR AB "factor structure") OR (TI "factor structures" OR AB "factor structures") OR (TI dimension* OR AB dimension*) OR (TI subscale* OR AB subscale*) OR ((TI multitrait OR AB multitrait) AND (TI scaling OR AB scaling) AND ((TI analysis OR AB analysis) OR (TI analyses OR AB analyses))) OR (TI "item discriminant" OR AB "item discriminant") OR (TI "interscale correlation*" OR AB "interscale correlation*") OR (TI error OR AB error) OR (TI errors OR AB errors) OR (TI "individual variability" OR AB "individual variability") OR (TI "interval variability" OR AB "interval variability") OR (TI "rate variability" OR AB "rate variability") OR ((TI variability OR AB variability) AND ((TI analysis OR AB analysis) OR (TI values OR AB values))) OR ((TI uncertainty OR AB uncertainty) AND ((TI measurement OR AB measurement) OR (TI measuring OR AB measuring))) OR (TI "standard error of measurement" OR AB "standard error of measurement") OR (TI sensitiv* OR AB sensitiv*) OR (TI responsive* OR AB responsive*) OR ((TI limit OR AB limit) AND (TI detection OR AB detection)) OR (TI "minimal detectable concentration" OR AB "minimal detectable concentration") OR (TI interpretab* OR AB interpretab*) OR (((TI minimal OR AB minimal) OR (TI minimally OR AB minimally) OR (TI clinical OR AB clinical) OR (TI clinically OR AB clinically)) AND ((TI important OR AB important) OR (TI significant OR AB significant) OR (TI detectable OR AB detectable)) AND ((TI change OR AB change) OR (TI difference OR AB difference))) OR ((TI small* OR AB small*) AND ((TI real OR AB real) OR (TI detectable OR AB detectable)) AND ((TI change OR AB change) OR (TI difference OR AB difference))) OR (TI "meaningful change" OR AB "meaningful change") OR (TI "ceiling effect" OR AB "ceiling effect") OR (TI "floor effect" OR AB "floor effect") OR (TI "Item response model" OR AB "Item response model") OR (TI IRT OR AB IRT) OR (TI Rasch OR AB Rasch) OR (TI "Differential item functioning" OR AB "Differential item functioning") OR (TI DIF OR AB DIF) OR (TI "computer adaptive testing" OR AB "computer adaptive testing") OR (TI "item bank" OR AB "item bank") OR (TI "cross-cultural equivalence" OR AB "cross-cultural equivalence")))

*NOT*

((PT address) OR (PT biography) OR (PT "case reports") OR (PT comment) OR (PT directory) OR (PT editorial) OR (PT festschrift) OR (PT interview) OR (PT lecture) OR (PT "legal case") OR (PT legislation) OR (PT letter) OR (PT news) OR (PT "newspaper article") OR (PT "patient education handout") OR (PT "popular work") OR (PT congress) OR (PT "consensus development conference") OR (PT "consensus development conference, nih") OR (PT "practice guideline")) OR (DE “Animals” NOT humans)

**Cochrane Library database search strategy**

MeSH descriptor: [Atrial Fibrillation] explode all trees or (atrial NEAR/2 fibrillation*):ti,ab OR (atrium NEAR/2 fibrillation*):ti,ab OR (auricular NEAR/2 fibrillation*):ti,ab

*AND*

HR-PRO:ti,ab OR HRPRO:ti,ab OR HRQL:ti,ab OR HRQoL:ti,ab OR QL:ti,ab OR QoL:ti,ab OR "quality of life":ti,ab,kw OR "life quality":ti,ab,kw OR (health NEAR/2 index*):ti,ab OR "health indices":ti,ab OR (health NEAR/2 profile*):ti,ab OR "health status":ti,ab,kw OR ((patient:ti,ab OR self:ti,ab OR child:ti,ab OR parent:ti,ab OR carer:ti,ab OR proxy:ti,ab) AND ((report:ti,ab OR reported:ti,ab OR reporting:ti,ab) OR (rated:ti,ab OR rating:ti,ab OR ratings:ti,ab) OR based:ti,ab OR (assessed:ti,ab OR assessment:ti,ab OR assessments:ti,ab))) OR ((disability:ti,ab OR function:ti,ab OR functional:ti,ab OR functions:ti,ab OR subjective:ti,ab OR utility:ti,ab OR utilities:ti,ab OR wellbeing:ti,ab OR "well being":ti,ab) AND (outcome:ti,ab OR outcomes:ti,ab OR index:ti,ab OR indices:ti,ab OR instrument:ti,ab OR instruments:ti,ab OR measure:ti,ab OR measures:ti,ab OR questionnaire:ti,ab OR questionnaires:ti,ab OR profile:ti,ab OR profiles:ti,ab OR scale:ti,ab OR scales:ti,ab OR score:ti,ab OR scores:ti,ab OR status:ti,ab OR survey:ti,ab OR surveys:ti,ab))

*AND*

((instrumentation:kw OR methods:kw OR "validation study":pt OR "Comparative Study":pt OR psychometrics:kw OR psychometr*:ti,ab OR clinimetr*:ti,ab,kw OR clinometr*:ti,ab,kw OR "outcome assessment, health care":ti,ab,kw OR "outcome assessment":ti,ab OR (outcome NEXT/2 measure*):ti,ab,kw OR "observer variation":ti,ab,kw OR "Health Status Indicators":ti,ab,kw OR "reproducibility of results":ti,ab,kw OR reproducib*:ti,ab OR "discriminant analysis":ti,ab,kw OR reliab*:ti,ab OR unreliab*:ti,ab OR valid*:ti,ab OR "coefficient of variation":ti,ab OR coefficient:ti,ab OR homogeneity:ti,ab OR homogeneous:ti,ab OR "internal consistency":ti,ab OR (cronbach*:ti,ab AND (alpha:ti,ab OR alphas:ti,ab)) OR (item:ti,ab AND (correlation*:ti,ab OR selection*:ti,ab OR reduction*:ti,ab)) OR agreement:ti,ab,kw OR precision:ti,ab,kw OR imprecision:ti,ab,kw OR "precise values":ti,ab,kw OR test-retest:ti,ab OR (test:ti,ab AND retest:ti,ab) OR (reliab*:ti,ab AND (test:ti,ab OR retest:ti,ab)) OR stability:ti,ab OR interrater:ti,ab OR inter-rater:ti,ab OR intrarater:ti,ab OR intra-rater:ti,ab OR intertester:ti,ab OR inter-tester:ti,ab OR intratester:ti,ab OR intra-tester:ti,ab OR interobserver:ti,ab OR inter-observer:ti,ab OR intraobserver:ti,ab OR intra-observer:ti,ab OR intertechnician:ti,ab OR inter-technician:ti,ab OR intratechnician:ti,ab OR intra-technician:ti,ab OR interexaminer:ti,ab OR inter-examiner:ti,ab OR intraexaminer:ti,ab OR intra-examiner:ti,ab OR interassay:ti,ab OR inter-assay:ti,ab OR intraassay:ti,ab OR intra-assay:ti,ab OR interindividual:ti,ab OR inter-individual:ti,ab OR intraindividual:ti,ab OR intra-individual:ti,ab OR interparticipant:ti,ab OR inter-participant:ti,ab OR intraparticipant:ti,ab OR intra-participant:ti,ab OR kappa:ti,ab OR kappa’s:ti,ab OR kappas:ti,ab OR repeatab*:ti,ab,kw OR ((replicab*:ti,ab,kw OR repeated:ti,ab,kw) AND (measure:ti,ab,kw OR measures:ti,ab,kw OR findings:ti,ab,kw OR result:ti,ab,kw OR results:ti,ab,kw OR test:ti,ab,kw OR tests:ti,ab,kw)) OR generaliza*:ti,ab OR generalisa*:ti,ab OR concordance:ti,ab OR (intraclass:ti,ab AND correlation*:ti,ab) OR discriminative:ti,ab OR "known group":ti,ab OR "factor analysis":ti,ab OR "factor analyses":ti,ab OR "factor structure":ti,ab OR "factor structures":ti,ab OR dimension*:ti,ab OR subscale*:ti,ab OR (multitrait:ti,ab AND scaling:ti,ab AND (analysis:ti,ab OR analyses:ti,ab)) OR "item discriminant":ti,ab OR ("interscale" NEXT correlation*):ti,ab OR error:ti,ab OR errors:ti,ab OR "individual variability":ti,ab OR "interval variability":ti,ab OR "rate variability":ti,ab OR (variability:ti,ab AND (analysis:ti,ab OR values:ti,ab)) OR (uncertainty:ti,ab AND (measurement:ti,ab OR measuring:ti,ab)) OR "standard error of measurement":ti,ab OR sensitiv*:ti,ab OR responsive*:ti,ab OR (limit:ti,ab AND detection:ti,ab) OR "minimal detectable concentration":ti,ab OR interpretab*:ti,ab OR ((minimal:ti,ab OR minimally:ti,ab OR clinical:ti,ab OR clinically:ti,ab) AND (important:ti,ab OR significant:ti,ab OR detectable:ti,ab) AND (change:ti,ab OR difference:ti,ab)) OR (small*:ti,ab AND (real:ti,ab OR detectable:ti,ab) AND (change:ti,ab OR difference:ti,ab)) OR "meaningful change":ti,ab OR "ceiling effect":ti,ab OR "floor effect":ti,ab OR "Item response model":ti,ab OR IRT:ti,ab OR Rasch:ti,ab OR "Differential item functioning":ti,ab OR DIF:ti,ab OR "computer adaptive testing":ti,ab OR "item bank":ti,ab OR "cross-cultural equivalence":ti,ab))

*NOT*

(address:pt OR biography:pt OR "case reports":pt OR comment:pt OR directory:pt OR editorial:pt OR festschrift:pt OR interview:pt OR lecture:pt OR "legal case":pt OR legislation:pt OR letter:pt OR news:pt OR "newspaper article":pt OR "patient education handout":pt OR "popular work":pt OR congress:pt OR "consensus development conference":pt OR "consensus development conference, nih":pt OR "practice guideline":pt) OR (animals:kw NOT humans:kw)

**Web of Science (Conference Proceedings Citation Index - Science) via Clarivate interface database search strategy**

(TI=("atrial fibrillation*" OR "atrium fibrillation*" or "auricular fibrillation*")) OR AB=("atrial fibrillation*" OR "atrium fibrillation*" or "auricular fibrillation*")

*AND*

((TI=HR-PRO OR AB=HR-PRO) OR (TI=HRPRO OR AB=HRPRO) OR (TI=HRQL OR AB=HRQL) OR (TI=HRQoL OR AB=HRQoL) OR (TI=QL OR AB=QL) OR (TI=QoL OR AB=QoL) OR (TS="quality of life") OR (TS="life quality") OR (TI="health index*" OR AB="health index*") OR (TI="health indices" OR AB="health indices") OR (TI="health profile*" OR AB="health profile*") OR (TS="health status") OR (((TI=patient OR AB=patient) OR (TI=self OR AB=self) OR (TI=child OR AB=child) OR (TI=parent OR AB=parent) OR (TI=carer OR AB=carer) OR (TI=proxy OR AB=proxy)) AND (((TI=report OR AB=report) OR (TI=reported OR AB=reported) OR (TI=reporting OR AB=reporting)) OR ((TI=rated OR AB=rated) OR (TI=rating OR AB=rating) OR (TI=ratings OR AB=ratings)) OR (TI=based OR AB=based) OR ((TI=assessed OR AB=assessed) OR (TI=assessment OR AB=assessment) OR (TI=assessments OR AB=assessments)))) OR (((TI=disability OR AB=disability) OR (TI=function OR AB=function) OR (TI=functional OR AB=functional) OR (TI=functions OR AB=functions) OR (TI=subjective OR AB=subjective) OR (TI=utility OR AB=utility) OR (TI=utilities OR AB=utilities) OR (TI=wellbeing OR AB=wellbeing) OR (TI="well being" OR AB="well being")) AND ((TI=index OR AB=index) OR (TI=indices OR AB=indices) OR (TI=instrument OR AB=instrument) OR (TI=instruments OR AB=instruments) OR (TI=measure OR AB=measure) OR (TI=measures OR AB=measures) OR (TI=questionnaire OR AB=questionnaire) OR (TI=questionnaires OR AB=questionnaires) OR (TI=profile OR AB=profile) OR (TI=profiles OR AB=profiles) OR (TI=scale OR AB=scale) OR (TI=scales OR AB=scales) OR (TI=score OR AB=score) OR (TI=scores OR AB=scores) OR (TI=status OR AB=status) OR (TI=survey OR AB=survey) OR (TI=surveys OR AB=surveys))))

*AND*

(((TS=Instrumentation) OR (TS=methods) OR (TS="Validation study") OR (TS="Comparative study") OR (TS=psychometr*) OR (TS=clinimetr*) OR (TS=clinometr*) OR (TS="outcome assessment, health care") OR (TI="outcome assessment" OR AB="outcome assessment") OR (TS="outcome measure*") OR (TS="observer variation") OR (TS="Health Status Indicators") OR (TS="reproducibility of results") OR (TI=reproducib* OR AB=reproducib*) OR (TS="discriminant analysis") OR (TI=reliab* OR AB=reliab*) OR (TI=unreliab* OR AB=unreliab*) OR (TI=valid* OR AB=valid*) OR (TI="coefficient of variation" OR AB="coefficient of variation") OR (TI=coefficient OR AB=coefficient) OR (TI=homogeneity OR AB=homogeneity) OR (TI=homogeneous OR AB=homogeneous) OR (TI="internal consistency" OR AB="internal consistency") OR ((TI=cronbach* OR AB=cronbach*) AND ((TI=alpha OR AB=alpha) OR (TI=alphas OR AB=alphas))) OR ((TI=item OR AB=item) AND ((TI=correlation* OR AB=correlation*) OR (TI=selection* OR AB=selection*) OR (TI=reduction* OR AB=reduction*))) OR (TS=agreement) OR (TS=precision) OR (TS=imprecision) OR (TS="precise values") OR (TI=test-retest OR AB=test-retest) OR ((TI=test OR AB=test) AND (TI=retest OR AB=retest)) OR ((TI=reliab* OR AB=reliab*) AND ((TI=test OR AB=test) OR (TI=retest OR AB=retest))) OR (TI=stability OR AB=stability) OR (TI=interrater OR AB=interrater) OR (TI=inter-rater OR AB=inter-rater) OR (TI=intrarater OR AB=intrarater) OR (TI=intra-rater OR AB=intra-rater) OR (TI=intertester OR AB=intertester) OR (TI=inter-tester OR AB=inter-tester) OR (TI=intratester OR AB=intratester) OR (TI=intra-tester OR AB=intra-tester) OR (TI=interobserver OR AB=interobserver) OR (TI=inter-observer OR AB=inter-observer) OR (TI=intraobserver OR AB=intraobserver) OR (TI=intra-observer OR AB=intra-observer) OR (TI=intertechnician OR AB=intertechnician) OR (TI=inter-technician OR AB=inter-technician) OR (TI=intratechnician OR AB=intratechnician) OR (TI=intra-technician OR AB=intra-technician) OR (TI=interexaminer OR AB=interexaminer) OR (TI=inter-examiner OR AB=inter-examiner) OR (TI=intraexaminer OR AB=intraexaminer) OR (TI=intra-examiner OR AB=intra-examiner) OR (TI=interassay OR AB=interassay) OR (TI=inter-assay OR AB=inter-assay) OR (TI=intraassay OR AB=intraassay) OR (TI=intra-assay OR AB=intra-assay) OR (TI=interindividual OR AB=interindividual) OR (TI=inter-individual OR AB=inter-individual) OR (TI=intraindividual OR AB=intraindividual) OR (TI=intra-individual OR AB=intra-individual) OR (TI=interparticipant OR AB=interparticipant) OR (TI=inter-participant OR AB=inter-participant) OR (TI=intraparticipant OR AB=intraparticipant) OR (TI=intra-participant OR AB=intra-participant) OR (TI=kappa OR AB=kappa) OR (TI=kappa’s OR AB=kappa’s) OR (TI=kappas OR AB=kappas) OR (TS=repeatab*) OR ((TS=replicab* OR TS=repeated) AND (TS=measure OR TS=measures OR TS=findings OR TS=result OR TS=results OR TS=test OR TS=tests)) OR (TI=generaliza* OR AB=generaliza*) OR (TI=generalisa* OR AB=generalisa*) OR (TI=concordance OR AB=concordance) OR ((TI=intraclass OR AB=intraclass) AND (TI=correlation* OR AB=correlation*)) OR (TI=discriminative OR AB=discriminative) OR (TI="known group" OR AB="known group") OR (TI="factor analysis" OR AB="factor analysis") OR (TI="factor analyses" OR AB="factor analyses") OR (TI="factor structure" OR AB="factor structure") OR (TI="factor structures" OR AB="factor structures") OR (TI=dimension* OR AB=dimension*) OR (TI=subscale* OR AB=subscale*) OR ((TI=multitrait OR AB=multitrait) AND (TI=scaling OR AB=scaling) AND ((TI=analysis OR AB=analysis) OR (TI=analyses OR AB=analyses))) OR (TI="item discriminant" OR AB="item discriminant") OR (TI="interscale correlation*" OR AB="interscale correlation*") OR (TI=error OR AB=error) OR (TI=errors OR AB=errors) OR (TI="individual variability" OR AB="individual variability") OR (TI="interval variability" OR AB="interval variability") OR (TI="rate variability" OR AB="rate variability") OR ((TI=variability OR AB=variability) AND ((TI=analysis OR AB=analysis) OR (TI=values OR AB=values))) OR ((TI=uncertainty OR AB=uncertainty) AND ((TI=measurement OR AB=measurement) OR (TI=measuring OR AB=measuring))) OR (TI="standard error of measurement" OR AB="standard error of measurement") OR (TI=sensitiv* OR AB=sensitiv*) OR (TI=responsive* OR AB=responsive*) OR ((TI=limit OR AB=limit) AND (TI=detection OR AB=detection)) OR (TI="minimal detectable concentration" OR AB="minimal detectable concentration") OR (TI=interpretab* OR AB=interpretab*) OR (((TI=minimal OR AB=minimal) OR (TI=minimally OR AB=minimally) OR (TI=clinical OR AB=clinical) OR (TI=clinically OR AB=clinically)) AND ((TI=important OR AB=important) OR (TI=significant OR AB=significant) OR (TI=detectable OR AB=detectable)) AND ((TI=change OR AB=change) OR (TI=difference OR AB=difference))) OR ((TI=small* OR AB=small*) AND ((TI=real OR AB=real) OR (TI=detectable OR AB=detectable)) AND ((TI=change OR AB=change) OR (TI=difference OR AB=difference))) OR (TI="meaningful change" OR AB="meaningful change") OR (TI="ceiling effect" OR AB="ceiling effect") OR (TI="floor effect" OR AB="floor effect") OR (TI="Item response model" OR AB="Item response model") OR (TI=IRT OR AB=IRT) OR (TI=Rasch OR AB=Rasch) OR (TI="Differential item functioning" OR AB="Differential item functioning") OR (TI=DIF OR AB=DIF) OR (TI="computer adaptive testing" OR AB="computer adaptive testing") OR (TI="item bank" OR AB="item bank") OR (TI="cross-cultural equivalence" OR AB="cross-cultural equivalence")))

*NOT*

(TS=address OR TS=biography OR TS="case reports" OR TS=comment OR TS=directory OR TS=editorial OR TS=festschrift OR TS=interview OR TS=lecture OR TS="legal case" OR TS=legislation OR TS=letter OR TS=news OR TS="newspaper article" OR TS="patient education handout" OR TS="popular work" OR TS=congress OR TS="consensus development conference" OR TS="consensus development conference, nih" OR TS="practice guideline") OR (TS=animals NOT TS=humans)

**Supplementary Table S1.** Patient-reported outcomes for assessing atrial fibrillation patients, identified by the International Consortium for Health Outcomes Measurement (ICHOM) AF working group, along with their definitions

| **PRO** | **Definition of PRO** |
| --- | --- |
| HRQoL | The influence of one’s health on different parameters of their lives |
| Physical function | An individual’s functional capacity when carrying out routine activities |
| Emotional function | The presence and/or degree of stress, depression or anxiety in an individual |
| Cognitive function | Indications of cognitive impairment |
| Symptom severity | The intensity and/or extent of an individual’s symptoms |
| Exercise tolerance | Assessment of energy expenditure, time and/or intensity when performing physical tasks, using metrics such as metabolic equivalents or calories |
| Ability to work | Presenteeism, absenteeism or employment-related productivity |

HRQoL, health-related quality of life; PRO, patient-reported outcome

**Supplementary Table S2.** Definitions and standards for good measurement properties according to COSMIN, adapted from^1, 2^

| **Measurement property** | | **Definition** | **Criteria for a positive (+) rating** |
| --- | --- | --- | --- |
| **Validity** | Structural validity | The extent to which the PROM comprehensively captures the dimensionality of the construct it measures | *CTT*: CFI, TLI or comparable measure >0.95 *OR* RMSEA <0.06 OR SRMS <0.08  *IRT/Rasch*:  CFI, TFLI or comparable measure >0.95 *OR* RMSEA <0.06 *OR* SRMS <0.08 ***AND*** residual correlations among items <0.20 *OR* R Q3’s <0.37 ***AND*** item scalability >0.30 *OR* adequate looking graphs ***AND*** for IRT: χ^2^ >0.01, for Rasch: infit/outfit values ≥0.5 and ≤1.5 *OR* Z-standardised values >-2 and <2 |
|  | Hypothesis testing for construct validity | The extent to which the PROM scores are consistent or differ with similar domains of other PROMs (convergent validity) or between known groups of individuals (discriminative validity) | Result is ≥75% in agreement with the hypotheses |
|  | Cross-cultural validity | The extent to which items from a translated or culturally adapted PROM correspond accurately to the performance of the original PROM version | No significant variations observed between group factors (e.g. age, gender or language) or no meaningful DIF for group factors (McFadden’s R^2^ <0.02) |
|  | Criterion validity | The extent to which the PROM scores correspond to those of a gold standard | Correlation with gold standard ≥0.70 or AUC ≥0.70 |
| **Reliability** | Internal consistency | The extent of inter-correlation between PROM items | At least low evidence (according to quality assessment of the evidence) for sufficient structural validity and Cronbach’s alpha ≥0.70 for each scale or subscale |
|  | Reliability | The proportion of the overall variance in the measurements attributable to actual differences over time (test-retest) or variability among different individuals administering the PROM on the same occasion (inter-rater) or across different occasions (intra-rater) | ICC or weighted Kappa ≥0.70 |
|  | Measurement error | The extent to which the PROM can accurately evaluate genuine changes in the construct it measures | SDC or LoA < MIC |
| **Responsiveness** |  | The capacity of a PROM to identify changes in the construct being assessed over time | Result is ≥75% in agreement with the hypothesis or the AUC ≥0.70 |

*AUC, Area Under the Curve; CFA, Confirmatory Factor Analysis; CFI, Comparative Fit Index; CTT, Classical Test Theory; DIF, Differential Item Functioning; ICC, Intraclass Correlation Coefficient; IRT, Item Response Theory; LoA, Limits of Agreement; MIC, Minimal Important Change; PROM, Patient-Reported Outcome Measure; RMSEA, Root Mean Square Error of Approximation; SEM, Standard Error of Measurement; SDC, Smallest Detectable Change; SRMS, Standardised Root Mean Residuals; TLI, Tucker-Lewis Index*

Supplementary Table 3 has been uploaded as a separate file due to the size of the file.

**Supplementary Table S4**. Risk of bias and measurement properties ratings for symptom severity patient-reported outcome measures for individuals with atrial fibrillation

| **PROM** | | **Risk of Bias (quality assessment) and COSMIN good measurement properties ratings** | | | | | | | | |
| --- | --- | --- | --- | --- | --- | --- | --- | --- | --- | --- |
|  | | **PROM development** | **Content validity** | **Structural validity** | **Internal consistency** | **Reliability** | **Cross-cultural validity/ Measurement error** | **Criterion validity** | **Hypothesis testing for construct validity** | **Responsiveness** |
| **AFSymp** | | A **doubtful** rating was assigned because it is unclear whether at least two researchers coded qualitative data during PROM development independently. | A **doubtful** rating was assigned because it is unclear which approach was used to analyse feedback obtained from professionals and patients.  **(+)** assigned as feedback on the relevance and clinical importance of AFSymp’s items was obtained from 21 international expert clinicians.  30 patients evaluated the relevance and comprehensibility of PROM items during its development. Nine items were rephrased, and two items were deleted based on this qualitative patient feedback to improve AFSymp’s understandability. | A **doubtful** rating was assigned due to a minor methodological flaw (absence of a description of the factor analysis rotation method).  **(+)** assigned as 7-item CFI (basis of global AFSymp score) = 0.998. | A **very good** rating was assigned as an internal consistency statistic was calculated for each of the PROM’s 11 items.  **(+)** assigned as  Cronbach’s α values for AFSymp domains: tiredness = 0.91, heart symptoms = 0.82, chest discomfort = 0.79 and single global score = 0.87. | An **adequate** rating was assigned because:  AFSymp’s test-retest reliability was evaluated on two occasions in patients considered stable, as indicated by their ‘about the same’ response on the OTE questionnaire on their second visit. However, limited data on the OTE questionnaire (e.g. questions used for the OTE questionnaire and answers obtained) were not reported. It is assumable that test conditions were consistent across both visits, and across all sites where psychometric evaluations were conducted, but no evidence of this was presented in the paper.  **(+)** assigned due to ICC values for AFSymp domains: tiredness = 0.77, heart symptoms = 0.74, chest discomfort = 0.76 and single global score = 0.78. | Δ | Δ | A **doubtful** rating was assigned due to insufficient descriptions of the subgroup (symptom levels of very mild vs mild vs moderate vs severe) characteristics which were used to assess known-groups validity.  (+) assigned for convergent validity as positive correlations were observed between AFSymp and the comparator PROM which measures a similar construct used in the study (the AFSS) ranging from r = 0.55 to 0.67, in accordance with H1. | Δ |
| **AFS/B** | | An **inadequate** rating was assigned as no evidence of qualitative data being coded during PROM development was reported. | Although senior electrophysiologists were consulted to generate AFS/B’s questions, an **inadequate** rating was assigned as professionals were not asked to feedback on comprehensiveness or relevance of PROM items overall.  Professionals were only asked to give an assessment of symptom severity and burden for the included patients, not to assess the PROM itself.  The study appendix includes a questionnaire suggesting that patients were consulted for feedback on the PROM, however it is unclear if this applied to each AFS/B item. Usability, comprehensiveness and relevance feedback from the questionnaire were not reported, limiting the assessment of content validity due to insufficient information, a **(?)** was given. | A **doubtful** rating was assigned as although factor analysis was conducted, the type (CFA or EFA) or rotation method were not reported.  **(?)** rating assigned, statistics for factor analysis were not reported. | An **inadequate** rating was assigned as weighted kappa values and Cronbach’s α was only reported for the AFS scale (α = 0.82), not AFB scale.  This warranted a **(?)** rating as although the reported Cronbach’s α meets the COSMIN criteria for a (+) rating, the overall judgement of internal consistency for the PROM cannot be determined due to missing information on the burden construct. | A **doubtful** rating was assigned as no information was reported pertaining to administration conditions during PROM development and validation.  **(?)** rating assigned as ICC = 0.93 only for AFS, but nothing was reported for ASB so a rating for the whole PROM cannot be determined. | Δ | Δ | **A doubtful** rating was assigned due to insufficient descriptions of the subgroup (participants with different symptom classifications) characteristics which were used to assess known-groups validity. Additionally, a methodological flaw was identified for convergent validity, as the SF-12, used as the only comparator PROM, lacks symptom severity and AF burden constructs.  **(+)** rating was assigned. AFS’s physical score and SF-12’s physical health score domains correlated in accordance with H1 (r = -0.63), AFS’s anxiety score and SF-12’s mental health score domains correlated with H2 (r = -48) and AFB’s score and SF-12’s physical component summary score correlated in accordance with H3 (r = -0.27). | Δ |
| **AFSS** | Danish language validation study | Δ | While appropriate modifications such as reordering items 11 and 12 were implemented during translation to improve usability, and patients found the AFSS Danish clear and readable aside from minor comprehension issues with two items, it is unclear whether patient testing included assessment of the relevance or comprehensiveness of each PROM item in relation to AF and the context of its use.  As a result, a doubtful **(?)** rating was given. | Δ | Δ | Δ | Despite the recognised MAPI guidelines being utilised to ascertain cross-language equivalence and cultural relevance for AFSS Danish during translation, the limited sample size (n = 6) was not adequate to allow for a robust cultural validity evaluation, warranting a rating of **inadequate.**  **(?)** assigned as no factor analyses were conducted. | Δ | Δ | Δ |
|  | Turkish language validation study | Δ | Although the study states the PROM was ‘well perceived by Turkish patients with no need for any changes’, a **doubtful (?)** rating was assigned as details regarding the 130 included patients or any professionals being consulted about the relevance, comprehensiveness or comprehensibility of the PROM were not reported, therefore content validity cannot be determined. | Δ | A **very good** rating was assigned as  no critical methodological flaws were identified.  **(-)** assigned as although Cronbach’s α value for the PROM’s symptom severity (α = 0.88), AF severity (α = 0.77) and AF burden (α = 0.70) domains warrant a sufficient rating, its healthcare utilisation domain value was insufficient (α = 0.62).  Evidence for structural validity was also not reported. | A **very good** rating was assigned as  no critical methodological flaws were identified.  **(+)** assigned as ICC >0.80 for all items (test-retest). | Δ | Δ | A **doubtful** rating was assigned due to methodological flaws as the SF-36 comparator PROM used for convergent validity lacks symptom severity and health care utilisation domains.  Additionally, the other measures (EHRA and CCS-SAF) differ from the AFSS as they are physician reported measures, not PROMs.  For known groups validity, a methodological flaw was observed as descriptions of the subgroup characteristics were inadequate.  A **(-)** rating was assigned as  the analysis of correlations between two of the Turkish AFSS domains (total AF burden alongside the AF frequency domains) and all of SF-36’s domains, six out of eight SF-36 domains demonstrated correlations > r = 0.30, despite these constructs being unrelated to these Turkish AFSS domains, which is not in accordance with H3. | Δ |
|  | Indonesian language validation study | Δ | A **doubtful** rating was assigned due to ambiguity in details related to AFSS Indonesian’s relevance, comprehensiveness and comprehensibility.  Although the study provides evidence that aspects of content validity were assessed (‘the questionnaire was first pretested on 30 participants, then edited based on patient feedback’) further methodological details were not provided. Therefore, content validity could not be evaluated and a **(?)** rating was assigned. | Δ | A **very good** rating was assigned as no critical methodological flaws were identified.  **(-)** assigned due to the insufficient internal consistency of the total AF burden domain (Cronbach’s α = 0.651), despite the symptom severity (α = 0.778) and healthcare utilisation (α = 0.713) meeting COSMIN’s criteria for sufficiency. Additionally, no evidence for structural validity was reported. | Although the study exclusion criteria implies that the condition of included participants was stable during the test-retest period (exclusion of participants exhibiting signs of physical or psychological ailments, recent hospitalisation or cardiac surgery within the preceding month) and an appropriate test-retest interval of 7-14 days was employed, there is not enough data on consistency of test-retest conditions. Therefore, an **adequate** rating was assigned.  **(+)** assigned as test-retest ICC >0.80 for all items (ranging from r = 0.803-0.975). | Δ | Δ | Although the Indonesian AFSS was assessed against the SF-36 (QoL measure) and EHRA score (classification system based on symptom severity) for convergent validity, these comparator instruments do not have healthcare utilisation domains and EHRA is a physician reported measure, not a PROM. Due to these methodological flaws, a **doubtful** rating was assigned.  The total score of the Indonesian AFSS demonstrated strong correlations (r >0.60) with SF-36, which is not in accordance with H2. Given that aspects of the Indonesian AFSS and SF-36 PROMs measure related yet dissimilar constructs, COSMIN recommendations state that correlations should be within the range of r = 0.30 – 0.50.  Consequently, a **(-)** rating was assigned. | Δ |
| **ASTA** | Symptom severity scale development study | A **doubtful** rating was assigned.  Aspects related to concept elicitation and cognitive interviews were unclear, which elevate risk of bias according to COSMIN standards. For example, it is unclear whether meetings with patients were recorded and independently coded, or whether a suitable data analysis approach was used during PROM development. | A **doubtful** rating was assigned due to ambiguity in details related to ASTA symptom scale’s relevance, comprehensiveness and comprehensibility according to COSMIN guidelines.  **(+)** rating assigned as the ASTA symptom scale was developed in consultation with an expert panel who conducted multiple evaluations to ensure the PROM items were clinically relevant.  Over 300 patients evaluated the ASTA symptom scale during its development. Modifications were made following patient feedback to enhance its relevance to the user (e.g. inclusion of further symptoms, weakness and infirmity, as scale items) and item wording was revised to improve clarity and comprehension of the scale (e.g. supplementary explanations for the cold sweat symptom). | Δ | A **very good** rating assigned as no critical methodological flaws were identified.  **(+)** rating assigned as Cronbach’s α = 0.795 for the nine-item symptom scale and α = 0.822 for the 10-item symptom scale. | Δ | Δ | A **very good** rating was assigned for ASTA symptom scale for criterion validity, as it was tested against the gold-standard  SCL. Additionally, no critical methodological flaws were identified.  **(+)** rating assigned as mean Spearman’s correlation value between the ASTA symptom scale and SCL ≥0.70. | A **very good** rating was assigned due to the use of appropriate comparator PROMs (SF-36 for HRQoL and SCL for symptom severity).  **(+)** rating assigned for convergent validity as ASTA symptom scale correlations with SCL (r >0.60) are in line with H1. | Δ |
|  | HRQoL scale development study | A **doubtful** rating was assigned as it is not clear whether cognitive interviews with patients were recorded and transcribed verbatim or if data was independently coded during PROM development. | A **doubtful** rating assigned as despite appropriate content validity guide being used, some aspects of content validity e.g. unclear if patients were asked about relevance, comprehensiveness or comprehensibility for each individual item on the ASTA HRQoL scale or whether an appropriate interview guide was used.  **(+)** rating was given as an expert panel of 2 cardiac nurses with knowledge of psychometrics and 5 cardiologists assessed the relevance of the PROM and high CVI values (I-CVI = 0.86 to 1.0 and S-CVI = 0.99) were generated.  240 patients tested ASTA HRQoL, and the PROM was adjusted to enhance usability and understandability.  Based on patient suggestions, a single item of the PROM related to time spent with friends, relatives and acquaintances was separated into two distinct items - one to address interactions with friends and relatives, the other focusing on interactions with acquaintances. Its response scale was adapted based on patient feedback. | A **very good** rating was assigned as  CFA was performed and described, and no critical methodological flaws were identified.  **(+)** rating assigned as CFI values (Table 5): For the 1-factor model the initial CFI = 0.953 & respecified CFI = 0.988 and for the 2-factor model initial CFI = 0.964 and respecified CFI = 0.992. | A **very good** rating was assigned as no critical methodological flaws were identified.  **(+)** rating assigned as total Cronbach’s α = 0.91, α = 0.89 for the ASTA physical subscale and α = 0.79 for the ASTA mental subscale. | Δ | Δ | Δ | Appropriate constructs of the SF-36 (physical and mental subscales) were tested against the ASTA physical and mental component summary domains. A **very good** rating was assigned as no critical methodological flaws were identified.  **(-)** rating was assigned as although correlations in alignment with H1 were observed e.g. between the ASTA physical subscale and the SF-36 physical component summary domain (r = -0.61), stronger correlations were observed in the PROMs own subscales (between ASTA’s physical and mental scale, r = 0.68) which is not in alignment with H2 as these scales evaluate dissimilar constructs. | Δ |
|  | Polish language validation study | Δ | A **doubtful** rating was assigned as although the Polish ASTA was piloted in 15 patients, it is unclear whether an interview guide was used during piloting to ask patients to evaluate each item for its relevance or comprehensibility.  **(+)** rating assigned as patients reported that the Polish version of the ASTA was both user-friendly and effectively captured and represented their clinical symptomatic experiences.  After its initial translation by 2 native Polish-speaking clinicians, the Polish version of the ASTA was reviewed by a professional translation agency who provided suggestions to refine the phrasing of the PROM. Native Polish-speaking electrophysiologists, nurses and cardiologists then assessed the translated PROM to further ascertain its clarity, understandability and clinical relevance to patients with arrhythmias. | A **very good** rating assigned as no critical methodological flaws were identified.  **(+)** rating assigned as CFI for the 1-factor model CFI = 0.947 and CFI = 0.988 for the 2-factor model. | **Very good** rating assigned as no critical methodological flaws identified.  **(+)** rating assigned as Cronbach’s α = 0.718 for ASTA symptom scale and α = 0.855 for ASTA HRQoL scale. | Δ | Δ | Δ | Δ | Δ |
|  | Brazilian Portuguese language validation study (symptom scale) | Δ | A **doubtful** rating was given. Despite appropriate aspects of content validity being considered during translation (e.g. semantics and translation of colloquialisms to enhance cultural validity) and the content validity index being employed for data analysis by an expert panel, it remains unclear whether patient feedback obtained during the pre-test with 32 participants was recorded or transcribed verbatim.  **(+)** given as 93.8% of patients stated that the Brazilian Portuguese ASTA symptom scale was easy to understand, suggesting only minor phrasing modifications following feedback using the usability evaluation of instruments tool.  An expert panel comprised of eight professionals – a Brazilian cardiology nurse, an arrythmia specialist physician, four translators fluent in Brazilian and Swedish (original language of the ASTA PROM), a Portuguese linguist and a nurse practitioner with expertise in on translation theoretical frameworks - evaluated the content validity of PROM. The panel deemed the translated PROM clear and comprehensible and made no recommendations for any adjustments. | Δ | A **very good** rating was assigned as no critical methodological flaws were identified.  **(+)** rating assigned as Cronbach’s α = 0.79 | Δ | Δ | Δ | The Brazilian ASTA symptom scale was evaluated against the QLFA-v2 (or AFQLQ -v2), an appropriate comparator assessing the same constructs. However, an **adequate** rating was assigned as the study did not present the distribution or mean of Spearman’s r for convergent validity.  **(+)** rating assigned as  correlations between the Brazilian Portuguese ASTA scores and QVFA-v2 (r = 0.89) align with H1 and the study hypothesis for convergent validity. | Δ |
|  | English language validation study | Δ | The English ASTA underwent an extensive translation process involving a translation agency and a panel of four healthcare professionals, with three being native Swedish-speaking physicians who specialised in arrhythmias, who only recommended a minor revision for one item on the HRQoL scale.  202 patients completed the English ASTA and when asked to evaluate the phrasing of the PROM, no uncertainties were reported. Some response options  However, a **doubtful (?)** rating was assigned as it is unclear whether the patients or professionals evaluated the relevance or comprehensiveness of each PROM item. | A **doubtful** rating assigned as Rasch analysis rotation model not described (minor methodological flaw).  **(-)** rating was assigned as although unidimensionality was supported, there were issues with local dependency (e.g. residual correlations were 0.41 for items 7 and 8 of the English ASTA symptom scale and 0.59 for items 2 and 3 of the English ASTA HRQoL scale) and the model fit was not adequate as standardised fit residual values exceeded -2 and 2 for seven items in the PROM (e.g. item  6 was -2.27 for the English ASTA symptom scale, and item 9 was 2.90 for the English ASTA HRQoL scale). | A **very good** rating was assigned as no critical methodological flaws were identified.  **(+)** rating assigned as Cronbach’s α = 0.81 for ASTA symptom scale and α = 0.88 for ASTA HRQoL scale. | A **doubtful** rating was assigned due to insufficient data pertaining aspects of reliability in the study (e.g. unclear whether patients were stable when ASTA English’s test-retest reliability was being assessed).  Participants completed ASTA English before an intervention, but further details regarding their stability were not provided, indicating a potential bias.  **(+)** rating assigned as person separation ICC = 0.75 for the ASTA symptom scale and ICC = 0.77 for the ASTA HRQoL scale. | A **doubtful** rating was assigned as it is unclear how many subjects were included for each group in the DIF analyses.  **(+)** rating assigned as DIF was analysed based on age (<65 and 65+) and gender, and no important differences were observed. | Δ | Δ | Δ |
|  | Indonesian language validation study | Δ | Although the Indonesian version of ASTA was pretested in 30 participants and modified based on patient feedback, it is unclear whether professionals/patients were asked about the relevance, comprehensiveness and comprehensibility of each item, therefore a **doubtful** **(?)** rating was assigned. | Δ | A **very good** rating assigned as no critical methodological flaws were identified.  **(-)** rating assigned as although  Cronbach’s α = 0.789 for medication utilisation domain and α = 0.766 for the HRQoL domain, α = 0.629 for the arrhythmia specific symptoms domain. | A **doubtful** rating was assigned due to insufficient data pertaining aspects of reliability in the study (e.g. unclear whether patients were stable when ASTA Indonesian’s test-retest reliability was being assessed, or consistency of its administration conditions).  (+) assigned as test-retest ICC >0.80 for all items (ranging from r: 0.856-0.983; p < 0.01). | Δ | Δ | A **doubtful** rating was assigned as the SF-36 comparator PROM used for convergent validity lacks symptom severity and health care utilisation domains.  (+) rating assigned, a significant negative correlation between the total score of ASTA Indonesian and the SF-36 of r = -0.331 (p < 0.01) was observed, which is in line with H2. | Δ |
| **AF6** | Developmental study | **An inadequate** rating was assigned as rationale for PROM construct was not described in sufficient detail and inappropriate methods for item identification were used, as items were based solely on patient interviews with no qualitative approach described. | An **inadequate** and  (**±**) rating was assigned as  although the context of use for the PROM was sufficiently described (designed to assess the symptomatic experience of AF patients prior and following cardioversion), AF6 items were formatted by just one AF clinic nurse without any input from patients or other relevant professionals, limiting certainty of its relevance and comprehensiveness for its intended use.  Further, no evidence was reported regarding opinions on its comprehensibility following feedback from the 111 included participants, compromising content validity. | A **doubtful** rating assigned due to a minor methodological flaw (no description of the factor analysis rotation method).  **(+)** assigned based on the following criteria: mean square statistic of 0.99, Rash analysis confirmed AF6 unidimensionality after removal of three items, appropriate appearance of the Rasch analysis graph (Figure 3) and infit plus outfit mean values (0.99 and 1.01, respectively) meet COSMIN criteria for a sufficient rating. | **Very good** rating assigned as no critical methodological flaws were identified.  **(+)** assigned based as Cronbach’s α = 0.81 and evidence was reported for sufficient structural validity. | A **doubtful** rating was assigned due to insufficient data pertaining aspects of reliability in the study (e.g. unclear whether patients were stable when test-retest reliability was being assessed, or consistency of its administration conditions). Also, ICC evaluation was conducted in only nine participants which is not sufficient.  **(-)** rating given as although AF6 ICC values for  dyspnoea at rest (ICC = 0.82), dyspnoea on exertion (ICC = 0.88) and anxiety due to AF (ICC = 0.93) meet the criteria for a sufficient rating, limitations in daily life due to AF (ICC = 0.19), discomfort due to AF (ICC = 0.04) and fatigue due to AF (ICC = 0.39) items meet the criteria for an insufficient rating. | Δ | **A doubtful** rating was assigned due to methodological flaws. The AF6 was able to distinguish between different patients based on severity and frequencies of symptoms, as measured by the three levels of the SCL. However, criterion validity is compromised as at the time of study publication, the SCL was yet to be validated in Swedish (original language of the AF6).  **(?)** rating assigned as exact Pearson’s r values for correlations per item between the AF6 and SCL (gold-standard) were not reported. | A **doubtful** rating was assigned as the AF6 was compared to SF-36 to assess convergent validity, yet SF-36 does not have AF symptom domains.  Although some correlations were in accordance with H2, e.g. SF-36’s emotional health and AF6’s anxiety due to AF domains (r = -0.42472, p < 0.0001), the correlation coefficient values used to assess construct validity for the other comparator PROM with relevant arrhythmia symptom related constructs, the SCL, were not reported in the study. Therefore, a hypothesis testing for construct validity rating cannot be determined due to insufficient results, and a **(?)** rating was assigned. | Δ |
|  | Responsiveness validation study | Δ | Δ | Δ | Δ | Δ | Δ | Δ | Δ | AF6 responsiveness was assessed against itself, the AFSS and SF-36. However, effect sizes for the comparator PROMs were not reported separately, so a **doubtful** rating was assigned for this methodological flaw.  The omission of this explicit data also means a responsiveness rating cannot be determined. Additionally, no explicit hypothesis was stated for the expected effect size or standardised response mean values of the AF6, therefore a **(?)** rating for this measurement property was assigned. |
| **SCL** | Trial appraising measurement properties (SCL vs SF-36 in a radiofrequency catheter ablation cohort) | Δ | Δ | Δ | Δ | Δ | Δ | Δ | A **doubtful** rating was assigned as the SCL was compared to SF-36 to assess convergent validity, yet SF-36 does not have symptom severity or frequency domains (minor methodological flaw).  **(-)** given as strong inverse correlations were observed between unrelated constructs e.g. SCL items and physical and social subscales (r = -0.44 to -0.65, p < 0.0005) which does not align with H3. | Δ |
|  | Trial appraising measurement properties (SCL vs SF-36 in a pulmonary vein isolation cohort) | Δ | Δ | Δ | Δ | Δ | Δ | Δ | **Doubtful** rating assigned as SCL was compared to SF-36 to assess convergent validity, yet SF-36 does not have symptom severity or frequency domains (minor methodological flaw).  Significant inverse correlations were observed between every SF-36 subscale and the SCL (r = -0.31 to -0.50, p < 0.001) which is not in alignment with H3, thus a **(-)** rating was assigned. | Δ |
|  | Swedish language validation study | Δ | A **doubtful (?) rating** was assigned as some aspects of content validity e.g. unclear if patients were asked about relevance, comprehensiveness or comprehensibility for *each* i*ndividual* item or whether any quantitative approach was used for analyses. | An **adequate** rating was assigned as EFA was conducted.  **(?)** rating assigned as structural validity cannot be determined as values for CFA were not assessed or reported. | A **very good** rating was assigned as no critical methodological flaws were identified.  **(+)** rating assigned as Cronbach’s α = 0.81 for both SCL’s symptom scale and severity scale. | Δ | Δ | Δ | Δ | Δ |

*AF6, Atrial Fibrillation six-item questionnaire; AFB, Atrial Fibrillation Burden component of the Atrial Fibrillation Symptom and Burden questionnaire; AFQLQ-v2 or QLFA-v2, Quality of life questionnaire for patients with Atrial Fibrillation-version two; AFS, Atrial Fibrillation Symptom component of the Atrial Fibrillation Symptom and Burden questionnaire; AFS/B, Atrial Fibrillation Symptom and Burden questionnaire; AFSS, Atrial Fibrillation Symptom Severity Scale; AFSymp, Atrial Fibrillation Symptoms questionnaire; ASTA, Arrhythmia Specific questionnaire in Tachycardia; CCS-SAF, Canadian Cardiovascular Society Severity of Atrial Fibrillation scale; CFA, confirmatory factor analysis; CFI, Comparative fit index; COSMIN, Consensus based Standards for the selection of health Measurement Instruments; DIF, Differential Item Functioning; EFA, Exploratory Factor Analysis; EHRA score, European Hearth Rhythm Association classification system; FA, Factor Analysis; GRADE, Grading of Recommendations Assessment Development and Evaluation; ICC, Intra-class Correlation Coefficient; I-CVI, Item Content Validity Index; OTE, Overall Treatment Effect; PCA, Principal Component Analysis; QoL, Quality of Life; QVFA-v2, Quality of life questionnaire for patients with Atrial Fibrillation-version two; S-CVI, Scale Content Validity Index; SCL = Symptom Checklist: Frequency and Severity Scale; SF-12, 12-item Short Form Survey; SF-36, 36-item Short Form Survey*

Δ = insufficient evidence to the corresponding measurement property in the study for assessment using the Consensus based Standards for the selection of health Measurement Instruments tools
+ = measurement property is sufficient according to the criteria of good measurement properties recommended by the Consensus based Standards for the selection of health Measurement Instruments
- = measurement property is insufficient according to the criteria of good measurement properties recommended by the Consensus based Standards for the selection of health Measurement Instruments
? = measurement property can not be determined according to the criteria of good measurement properties recommended by the Consensus based Standards for the selection of health Measurement Instruments

H1 = Hypothesis 1 – Correlations with patient-reported outcome measures assessing similar constructs should be ≥0.50^1^
H2 = Hypothesis 2 – Correlations with patient-reported outcome measures assessing related yet dissimilar constructs should be 0.30-0.501^1^
H3 = Hypothesis 3 – Correlations with patient-reported outcome measures assessing unrelated constructs should be <0.30^1^

Studies were downgraded based on the criteria set by COSMIN for their Modified Grading of Recommendations, Assessment Development and Evaluation (GRADE) based on their Risk of Bias (RoB), imprecision, indirectness and inconsistency using the following methods:

- **RoB**
  - no downgrade if >1 adequate quality studies or only 1 very good study available
  - downgrade by 1 if >1 doubtful quality studies or only 1 adequate study available (serious RoB)
  - downgraded by 2 if >1 inadequate quality studies or only 1 doubtful quality study available (very serious RoB)
  - downgraded by 3 if >1 inadequate quality studies or only 1 inadequate quality study available (extremely serious RoB)
- **Inconsistency**
  - downgrade by 1 if measurement properties (sufficient 🡪 +, insufficient🡪 - or indeterminate 🡪 ?) or if RoB ratings (e.g. two studies deemed as very good and one deemed as doubtful quality) were variable across studies
- **Imprecision**
  - downgrade by 1 if pooled sample size <100
  - downgraded by 2 if pooled sample size <50
- **Indirectness**
  - downgrade by 1 if study population are variable regarding condition of interest (in the context of the purpose of the PROM), or if comparator PROMs consist of domains that are different from or not part of the symptom severity PROM.

The individual methodological studies were pooled, and quality of evidence (i.e. methodological studies) first began as being ‘high’, and if applicable, downgrading was applied according to COSMIN’s modified GRADE criteria to either moderate, low or very low, based on the lowest score determining the global rating. Supplementary Table S5 provides justifications for the GRADE ratings assigned to the symptom severity PROMs included in this systematic review.

**Supplementary Table S5.** Justifications for Modified Grading of Recommendations, Assessment Development and Evaluation (GRADE) ratings for the symptom severity patient-reported outcome measures used in individuals with atrial fibrillation

| **PROM** | **Risk of Bias** | | **Imprecision** | | **Indirectness** | | **Inconsistency** | **Overall modified GRADE rating** (based on the lowest score) |
| --- | --- | --- | --- | --- | --- | --- | --- | --- |
| **AFSymp** | Development study – one study of doubtful quality available | Downgraded by 2 to LOW certainty in quality of evidence | One study with a sample size of 91 for the concept elicitation study and 313 for psychometric testing of the PROM | Not downgraded | AFSymp is an AF-specific PROM, and participants in this study all had AF | Not downgraded | Downgraded by 1 due to the inconsistency in RoB ratings to MODERATE certainty in quality evidence. | *LOW certainty in quality of evidence* |
|  | Content validity – one study of doubtful quality available | Downgraded by 2 to LOW quality of evidence |  |  |  |  |  |  |
|  | Structural validity - one study of doubtful quality available | Downgraded by 2 to LOW certainty in quality of evidence |  |  |  |  |  |  |
|  | Internal consistency – one study of very good quality | Not downgraded |  |  |  |  |  |  |
|  | Reliability – one study of adequate quality available | Downgraded by 1 to MODERATE certainty in quality of evidence |  |  |  |  |  |  |
|  | Construct validity – one study of doubtful quality available | Downgraded by 2 to LOW certainty in quality of evidence |  |  |  |  |  |  |
| **AFS/B** | Development study – one study of inadequate quality available | Downgraded by 3 to VERY LOW certainty in quality of evidence | One study with a sample size of 224 | Not downgraded | AFS/B is an AF-specific PROM, and participants in this study all had AF | Not downgraded | Downgraded by 1 due to the inconsistency in RoB ratings to MODERATE certainty in quality evidence. | *VERY LOW certainty in quality of evidence* |
|  | Content validity– one study of inadequate quality available | Downgraded by 3 to VERY LOW certainty in quality of evidence |  |  |  |  |  |  |
|  | Structural validity - one study of doubtful quality available | Downgraded by 2 to LOW certainty in quality of evidence |  |  |  |  |  |  |
|  | Internal consistency – one study of inadequate quality available | Downgraded by 3 to VERY LOW certainty in quality of evidence |  |  |  |  |  |  |
|  | Reliability – one study of doubtful quality available | Downgraded by 2 to LOW certainty in quality of evidence |  |  |  |  |  |  |
|  | Construct validity - one study of doubtful quality available | Downgraded by 2 to LOW certainty in quality of evidence |  |  |  |  |  |  |
| **AFSS** | Content validity - three studies of doubtful quality available | Downgraded by 1 to MODERATE certainty in quality of evidence | Pooled study sample size of 196 | Not downgraded | AFSS is an AF-specific PROM, and participants in all three studies had AF | Not downgraded | Downgraded by 1 due to the inconsistency in RoB ratings to MODERATE certainty in quality evidence | *MODERATE certainty in quality of evidence* |
|  | Internal consistency – two studies of very good quality available.  Both studies rated as having insufficient measurement properties | Not downgraded |  |  |  |  |  |  |
|  | Reliability – two very good quality studies available | Not downgraded |  |  |  |  |  |  |
|  | Cross-cultural validity – one study of inadequate quality available | Downgraded by 2 to LOW certainty in quality of evidence |  |  |  |  |  |  |
|  | Construct validity – two studies of doubtful quality available | Downgraded by 1 to MODERATE certainty in quality of evidence |  |  |  |  |  |  |
| **ASTA** | Development studies – two studies of doubtful quality available | Downgraded by 1 to MODERATE certainty in quality of evidence | Pooled study sample size of 1,063 | Not downgraded | ASTA is a PROM designed for an arrhythmia population, and participants in all six studies had different types of arrhythmias | Not downgraded | Downgraded by 1 due to the inconsistency in RoB ratings to MODERATE certainty in quality evidence | *MODERATE certainty in quality of evidence* |
|  | Content validity – six studies of doubtful quality available | Downgraded by 1 to MODERATE certainty in quality of evidence |  |  |  |  |  |  |
|  | Structural validity – two studies of very good quality and one of doubtful quality available.  Two studies rated as having sufficient measurement property, one study with insufficient measurement property | Downgraded by 1 to MODERATE certainty in quality of evidence |  |  |  |  |  |  |
|  | Internal consistency – six studies of very good quality available.  All six studies rated as having sufficient measurement properties. | Not downgraded |  |  |  |  |  |  |
|  | Reliability – two studies of doubtful quality available | Downgraded by 1 to MODERATE certainty in quality of evidence |  |  |  |  |  |  |
|  | Measurement error – one study of doubtful quality available | Downgraded by 2 to LOW certainty in quality of evidence |  |  |  |  |  |  |
|  | Criterion validity – one study of very good quality available | Not downgraded |  |  |  |  |  |  |
|  | Construct validity – two studies of very good quality, one study of adequate quality and one study of doubtful quality available  All six studies rated as having sufficient measurement properties. | Not downgraded |  |  |  |  |  |  |
| AF6 | Development study - one study of inadequate quality available | Downgraded by 3 to VERY LOW certainty in quality of evidence | Pooled sample size of 111 | Not downgraded based on imprecision | AF6 is an AF-specific PROM designed for AF individuals undergoing catheter ablation, and participants in all three studies had AF and underwent catheter ablation | Not downgraded | Downgraded by 1 due to the inconsistency in RoB ratings to MODERATE certainty in quality evidence | *VERY LOW certainty in quality of evidence* |
|  | Content validity - one study of inadequate quality available | Downgraded by 3 to VERY LOW certainty in quality of evidence |  |  |  |  |  |  |
|  | Structural validity - one study of doubtful quality available | Downgraded by 2 to LOW certainty in quality of evidence |  |  |  |  |  |  |
|  | Internal consistency - one study of very good quality available | Not downgraded |  |  |  |  |  |  |
|  | Reliability – one study of doubtful quality available | Downgraded by 2 to LOW certainty in quality of evidence |  |  |  |  |  |  |
|  | Criterion validity - one study of doubtful quality available | Downgraded by 2 to LOW certainty in quality of evidence |  |  |  |  |  |  |
|  | Construct validity – one study of doubtful quality available | Downgraded by 2 to LOW certainty in quality of evidence |  |  |  |  |  |  |
|  | Responsiveness - one study of doubtful quality available | Downgraded by 2 to LOW certainty in quality of evidence |  |  |  |  |  |  |
| **SCL** | Content validity – one study of doubtful quality available | Downgraded by 2 to LOW certainty in quality of evidence | Pooled study sample size of 865 | Not downgraded | SCL is an arrhythmia specific PROM, and all three studies were conducted in populations with arrhythmias | Not downgraded | Downgraded by 1 due to the inconsistency in RoB ratings to MODERATE certainty in quality evidence | *LOW certainty in quality of evidence* |
|  | Structural validity – one study of adequate quality available | Downgraded by 1 to MODERATE certainty in quality of evidence |  |  |  |  |  |  |
|  | Internal consistency - one study of very good quality available | Not downgraded |  |  |  |  |  |  |
|  | Construct validity – two studies of doubtful quality available | Downgraded by 1 to MODERATE certainty in quality of evidence |  |  |  |  |  |  |

*AF, Atrial Fibrillation; AF6, Atrial Fibrillation six-item questionnaire; AFS/B, Atrial Fibrillation Symptom and Burden measure; AFSS, Atrial Fibrillation Symptom Severity Scale; AFSymp, Atrial Fibrillation Symptoms questionnaire; ASTA, Arrhythmia Specific questionnaire in Tachycardia and Arrhythmia; GRADE, Grading of Recommendations, Assessment Development and Evaluation; PROM, Patient Reported Outcome Measure; RoB, Risk of Bias; SCL, Symptom Checklist: Frequency and Severity Scale*

**References**

1. Prinsen CAC, Mokkink LB, Bouter LM, Alonso J, Patrick DL, de Vet HCW, et al. COSMIN guideline for systematic reviews of patient-reported outcome measures. Qual Life Res 2018;27:1147-57.
2. Mokkink L, Prinsen C, DL. P, Alonso J, Bouter L, de Vet H, et al. COSMIN methodology for systematic reviews of Patient‐Reported Outcome Measures (PROMs) - User manual (version 1.0). 2018. <https://www.cosmin.nl/tools/guideline-conducting-systematic-review-outcome-measures/> (11 August 2024, date last accessed).

**Appendix B:** COSMIN expansion of the PRISMA checklist

| **Section and Topic** | **#** | **Checklist item^a^** | **Location** |
| --- | --- | --- | --- |
| TITLE | | | |
| Title | 1 | Identify the report as a systematic review and include as applicable the following (in any order): outcome domain of interest, population  of interest, name/type of OMIs of interest, and measurement properties of interest. | Page 1 |
| ABSTRACT | | | |
| OPEN SCIENCE | | | |
| Funding^b^ | 2.2 | Specify the primary source of funding for the review. | N/A |
| Registration | 2.3 | Provide the register name and registration number. | Page 2 |
| BACKGROUND | | | |
| Objectives | 2.4 | Provide an explicit statement of the main objective(s) or question(s) the review addresses. | Page 2 |
| METHODS | | | |
| Eligibility criteria | 2.5 | Specify the inclusion and exclusion criteria for the review. | Page 2 |
| Information sources | 2.6 | Specify the information sources (e.g., databases, registers) used to identify studies and the date when each was last searched. | Page 2 |
| Risk of bias | 2.7 | Specify the methods used to assess risk of bias in the included studies. | Page 2 |
| Measurement properties | 2.8 | Specify the methods used to rate the results of a measurement property. | Page 2 |
| Synthesis methods | 2.9 | Specify the methods used to present and synthesize results. | Page 2 |
| RESULTS | | | |
| Included studies | 2.10 | Give the total number of included OMIs and study reports. | Page 2 |
| Synthesis of results | 2.11 | Present the syntheses of results of OMIs, indicating the certainty of the evidence. | Page 2-3 |
| DISCUSSION | | | |
| Limitations of evidence | 2.12 | Provide a brief summary of the limitations of the evidence included in the review (e.g., study risk of bias, inconsistency, and imprecision). | Page 2-3 |
| Interpretation | 2.13 | Provide a general interpretation of the results and important implications. | Page 3 |
| PLAIN LANGUAGE SUMMARY | | | |
| Plain language summary | 3 | If allowed by the journal, provide a plain language summary with background information and key findings. | N/A |
| OPEN SCIENCE | | | |
| Registration and protocol | 4a | Provide registration information for the review, including register name and registration number, or state that the review was not registered. | Page 8 |
|  | 4b | Indicate where the review protocol can be accessed, or state that a protocol was not prepared. | Page 8 |
|  | 4c | Describe and explain any amendments to information provided at registration or in the protocol. | N/A |
| Support | 5 | Describe sources of financial or non-financial support for the review, and the role of the funders in the review. | N/A |
| Competing interests | 6 | Declare any competing interests of review authors. | Page 32 |
| Availability of data, code, and other materials | 7 | Report which of the following are publicly available and where they can be found: template data collection forms; data extracted from  included studies; data used for all analyses; analytic code; any other materials used in the review. | Pages 8, 11, 14 |
| INTRODUCTION | | | |
| Rationale | 8 | Describe the rationale for the review in the context of existing knowledge. | Pages 4-6 |
| Objectives | 9 | Provide an explicit statement of the objective(s) or question(s) the review addresses and include as applicable the following (in any  order): outcome domain of interest, population of interest, name/type of OMIs of interest, and measurement properties of interest. | Page7 |
| METHODS | | | |
| Followed guidelines | 10 | Specify, with references, the methodology and/or guidelines used to conduct the systematic review. | Page 8 |
| Eligibility criteria | 11 | Specify the inclusion and exclusion criteria for the review. | Pages 8-9 |
| Information sources | 12 | Specify all databases, registers, preprint servers, websites, organizations, reference lists and other sources searched or consulted to  identify studies. Specify the date when each source was last searched or consulted. | Page 10 |
| Search strategy | 13 | Present the full search strategies for all databases, registers, and websites, including any filters and limits used. | Supplementary Appendix B |
| Selection process | 14 | Specify the methods used to decide whether a study met the inclusion criteria of the review, e.g., including how many reviewers  screened each record and each report retrieved, whether they worked independently, and if applicable, details of automation tools/AI  used in the process. | Page 10-11 |
| Data collection process | 15 | Specify the methods used to collect data from reports, e.g., including how many reviewers collected data from each report, whether  they worked independently, any processes for obtaining or confirming data from study investigators, and if applicable, details of  automation tools/AI used in the process. | Page 11 |
| Data items | 16 | List and define which data were extracted (e.g., characteristics of study populations and OMIs, measurement properties’ results, and  aspects of feasibility and interpretability). Describe methods used to deal with any missing or unclear information. | Page 11 |
| Study risk of bias assessment | 17 | Specify the methods used to assess risk of bias in the included studies, e.g., including details of the tool(s) used, how many reviewers  assessed each study and whether they worked independently, and if applicable, details of automation tools/AI used in the process. | Page 11 |
| Measurement properties | 18 | Specify the methods used to rate the results of a measurement property for each individual study and for the summarized or pooled  results, e.g., including how many reviewers rated each study and whether they worked independently. | Pages 11-12 |
| Synthesis methods | 19a | Describe the processes used to decide which studies were eligible for each synthesis. | Page 12-13 |
|  | 19b | Describe any methods used to synthesize results. | Page 12 |
|  | 19c | If applicable, describe any methods used to explore possible causes of inconsistency among study results (e.g., subgroup analysis). | N/A |
|  | 19d | If applicable, describe any sensitivity analyses conducted to assess robustness of the synthesized results. | N/A |
| Certainty assessment | 20 | Describe any methods used to assess certainty (or confidence) in the body of evidence. | Page 12 |
| Formulating recommendations | 21 | If appropriate, describe any methods used to formulate recommendations regarding the suitability of OMIs for a particular use. | Page 13 |
| RESULTS | | | |
| Study selection | 22a | Describe the results of the search and selection process, from the number of records identified in the search to the number of study  reports included in the review, ideally using a flow diagram. If applicable, also report the final number of OMIs included and the number  of study reports relevant to each OMI. [T] | Page 14, 41 |
|  | 22b | Cite study reports that might appear to meet the inclusion criteria, but which were excluded, and explain why they were excluded. | Page 14 |
|  |  |  |  |
| OMI characteristics | 23a | Present characteristics of each included OMI, with appropriate references. [T] | Pages 15-16, 45-47 |
|  | 23b | If applicable, present interpretability aspects for each included OMI. [T] | Pages 45-47 |
|  | 23c | If applicable, present feasibility aspects for each included OMI. [T] | Pages 45-47 |
| Study characteristics | 24 | Cite each included study report evaluating one or more measurement properties and present its characteristics. [T] | Supplementary table S1, Page 14, 44 |
| Risk of bias in studies | 25 | Present assessments of risk of bias for each included study. [T] | Page 16, 48 |
| Results of individual studies | 26 | For all measurement properties, present for each study: (a) the reported result and (b) the rating against quality criteria, ideally using  structured tables or plots. [T] | Page 48, Supplementary table S2 |
| Results of syntheses | 27a | Present results of all syntheses conducted. For each measurement property of an OMI, present: (a) the summarized or pooled result and  (b) the overall rating against quality criteria. [T] | Page 49, Supplementary table S3 |
|  | 27b | If applicable, present results of all investigations of possible causes of inconsistency among study results. | N/A |
|  | 27c | If applicable, present results of all sensitivity analyses conducted to assess the robustness of the synthesized results. | N/A |
| Certainty of evidence | 28 | Present assessments of certainty (or confidence) in the body of evidence for each measurement property of an OMI assessed. [T] | Page 49, Supplementary table S3 |
| Recommendations | 29 | If appropriate, make recommendations for suitable OMIs for a particular use. | Page 17 |
| DISCUSSION | | | |
| Discussion | 30a | Provide a general interpretation of the results in the context of other evidence. | Pages 18-19 |
|  | 30b | Discuss any limitations of the evidence included in the review. | Pages 19-21 |
|  | 30c | Discuss any limitations of the review processes used. | Page 19-21 |
|  | 30d | Discuss implications of the results for practice, policy, and future research. | Pages 21-24 |

*N/A = Not Applicable*
