# Supplementary material for: Patient-reported outcome measures for atrial fibrillation: a systematic review and evaluation of psychometric properties of symptom severity measures
Source: Europace. 2026 Sep 8;28(10):euag252. doi: 10.1093/europace/euag252 (PMC13678206; doi:10.1093/europace/euag252)
Supplement: Supplementary file 1 [file euag252_supplementary_data.zip › Supplementary table - S3.docx]

**Supplementary Table S3.** Data extraction table detailing study characteristics of full-text articles included in a systematic review investigating patient-reported outcome measures, with a focus on symptom severity instruments, used in individuals with atrial fibrillation

|  | | | | **Patient-reported outcome measure used to assess outcome** | | | | | | |
| --- | --- | --- | --- | --- | --- | --- | --- | --- | --- | --- |
| **Author** | **Country** | **Study design** | **Population characteristics (sample size, age, %female, ethnicity or race & AF type)** | **HRQoL** | **Physical function** | **Emotional function** | **Cognitive function** | **Symptom severity** | **Exercise tolerance** | **Ability to work** |
| Abu et al, 2020^1^ | USA | Cohort study | 1,097 | AFEQT | IADL | GAD-7 & PHQ-9 | MoCA | / | / | / |
|  |  |  | 72.5±7 years |  |  |  |  |  |  |  |
|  |  |  | 48.3% |  |  |  |  |  |  |  |
|  |  |  | White (87.4%) & non-White (12.6%) |  |  |  |  |  |  |  |
|  |  |  | Paroxysmal (67.7%), persistent (14.4%) & permanent (17.9%) |  |  |  |  |  |  |  |
| Abu et al, 2020 [a] ^2^ | USA | Cohort study | 1,097 | QDIS-MCC | / | GAD-7 | MoCA | / | / | / |
|  |  |  | 75.5±7 years |  |  |  |  |  |  |  |
|  |  |  | 48.3% |  |  |  |  |  |  |  |
|  |  |  | White (87.4%) & non-White (12.6%) |  |  |  |  |  |  |  |
|  |  |  | Paroxysmal (66.4%), persistent (15.1%) & permanent (18.5%) |  |  |  |  |  |  |  |
| Abed et al, 2013^3^ | Australia | RCT | 150 | / | / | / | / | AFSS | / | / |
|  |  |  | Intervention group (n=75): 59.8±9.5 years & control group (n=75) = 60.3±10.3 years |  |  |  |  |  |  |  |
|  |  |  | Intervention group: 32% & control group: 50% |  |  |  |  |  |  |  |
|  |  |  | NR |  |  |  |  |  |  |  |
|  |  |  | Intervention group: paroxysmal (59%) & control group: paroxysmal (44%) *persistent |  |  |  |  |  |  |  |
| Accinni et al, 2024^4^ | Italy | Cohort study | 89 with AF (total n=110) | / | / | PHQ-9 [Italian] | / | / | / | / |
|  |  |  | 76.05±10.69 years |  |  |  |  |  |  |  |
|  |  |  | 36.4% |  |  |  |  |  |  |  |
|  |  |  | NR |  |  |  |  |  |  |  |
|  |  |  | Paroxysmal (47.3%), persistent (7.3%) & permanent (26.4%) & not specified |  |  |  |  |  |  |  |
| Ad et al, 2013^5^ | USA | Cohort study | 104 | SF-12 | / | / | / | / | / | / |
|  |  |  | 55.9±9 years |  |  |  |  |  |  |  |
|  |  |  | 9% |  |  |  |  |  |  |  |
|  |  |  | NR |  |  |  |  |  |  |  |
|  |  |  | Long-standing persistent (81%) & persistent (23%) |  |  |  |  |  |  |  |
| Ad et al, 2013 [a] ^6^ | USA | Cohort study | 534 | SF-12 | / | / | / | SCL | / | / |
|  |  |  | Sinus rhythm at discharge group (n=469): 63.2±10.9 years & non sinus rhythm at discharge group (n=65): 67.4±10.1 years |  |  |  |  |  |  |  |
|  |  |  | Sinus rhythm at discharge group: 32% & non sinus rhythm at discharge group: 34% |  |  |  |  |  |  |  |
|  |  |  | NR |  |  |  |  |  |  |  |
|  |  |  | Sinus rhythm at discharge group: long-standing persistent (52%) & non sinus rhythm at discharge group: long-standing persistent (71%) |  |  |  |  |  |  |  |
| Ad et al, 2011^7^ | USA | Cohort study | 124 | SF-12 | / | / | / | SCL | / | / |
|  |  |  | Reported as age>75 years (n=23) & age >75 years (n=101) |  |  |  |  |  |  |  |
|  |  |  | NR |  |  |  |  |  |  |  |
|  |  |  | NR |  |  |  |  |  |  |  |
|  |  |  | Long-standing persistent or permanent (42%), persistent (49%) & paroxysmal (6%) |  |  |  |  |  |  |  |
| Ad et al, 2011 [a] ^8^ | USA | Cohort study | 42 | SF-12 | / | / | / | SCL | / | / |
|  |  |  | 61.6±12.9 years |  |  |  |  |  |  |  |
|  |  |  | 26% |  |  |  |  |  |  |  |
|  |  |  | NR |  |  |  |  |  |  |  |
|  |  |  | Paroxysmal (7%), persistent (48%) & long-standing persistent (45%) |  |  |  |  |  |  |  |
| Ad et al, 2011 [b] ^9^ | USA | Cohort study | 40 | SF-12 | / | / | / | SCL | / | / |
|  |  |  | 55.8±10.1 years |  |  |  |  |  |  |  |
|  |  |  | NR |  |  |  |  |  |  |  |
|  |  |  | NR |  |  |  |  |  |  |  |
|  |  |  | Long-standing persistent (95%) & persistent (5%) |  |  |  |  |  |  |  |
| Ad et al, 2012^10^ | USA | Cohort study | 95 | SF-12 | / | / | / | / | / |  |
|  |  |  | 68.2±9 years |  |  |  |  |  |  |  |
|  |  |  | 21% |  |  |  |  |  |  |  |
|  |  |  | NR |  |  |  |  |  |  |  |
|  |  |  | NR |  |  |  |  |  |  |  |
| Ad et al, 2013 [b] ^11^ | USA | Cohort study | 44 | SF-12 | / | / | / | / | / | / |
|  |  |  | 79.5±3 years |  |  |  |  |  |  |  |
|  |  |  | 48% |  |  |  |  |  |  |  |
|  |  |  | NR |  |  |  |  |  |  |  |
|  |  |  | NR |  |  |  |  |  |  |  |
| Ahmed et al, 2010^12^ | Netherlands | RCT | 158 | SF-36 [Dutch] | / | / | / | AFSS | / | / |
|  |  |  | Episodic treatment group (n=75): 68±9 years & continuous treatment group (n=83): 65±9 years |  |  |  |  |  |  |  |
|  |  |  | Episodic treatment group: 43% & continuous treatment group: 33% |  |  |  |  |  |  |  |
|  |  |  | NR |  |  |  |  |  |  |  |
|  |  |  | Persistent (all participants) |  |  |  |  |  |  |  |
| Aguilera et al, 2022^13^ | NR | Cohort study | 838 | / | / | / | / | AFSS | / | / |
|  |  |  | Pulmonary vein connection group (n=684): 63±9 years & electrically silent pulmonary vein group (n=154): 65±8 years |  |  |  |  |  |  |  |
|  |  |  | 28% |  |  |  |  |  |  |  |
|  |  |  | NR |  |  |  |  |  |  |  |
|  |  |  | Pulmonary vein connection group: non-paroxysmal (45%) & not specified (55%) Electrically silent pulmonary vein group: non-paroxysmal (58%) & not specified (42%) |  |  |  |  |  |  |  |
| Ahn et al, 2021^14^ | South Korea | Cohort study | 66,692 | / | / | / | / | / | IPAQ [Korean] | / |
|  |  |  | 59.5±12.4 years |  |  |  |  |  |  |  |
|  |  |  | 36.4% |  |  |  |  |  |  |  |
|  |  |  | NR |  |  |  |  |  |  |  |
|  |  |  | Newly diagnosed (all participants) |  |  |  |  |  |  |  |
| Ahn et al, 2024^15^ | South Korea | Cohort study (registry) | 299 | EQ-5D-3L | / | / | / | / | / | / |
|  |  |  | 60±11 years |  |  |  |  |  |  |  |
|  |  |  | 24.7% |  |  |  |  |  |  |  |
|  |  |  | NR |  |  |  |  |  |  |  |
|  |  |  | Paroxysmal (50.2%) & persistent (49.8%) |  |  |  |  |  |  |  |
| Akintade et al, 2015^16^ | USA | Cross-sectional study | 150 | SF-36v2 | / | BDI-II & STAI | MMSE | SCL | / | / |
|  |  |  | 66.25±13.11 years |  |  |  |  |  |  |  |
|  |  |  | 40% |  |  |  |  |  |  |  |
|  |  |  | White (56.7%), African American (42.7%) & Other (0.7%) |  |  |  |  |  |  |  |
|  |  |  | Paroxysmal (17.3%), persistent (22.7%) & permanent (33.3%) |  |  |  |  |  |  |  |
| Al-Bashaireh et al, 2024^17^ | Jordan | Cohort study (registry) | 620 | AFEQT | / | / | / | / | / | / |
|  |  |  | Median age reported: 63.0 (IQR range 54.0-70.5) years |  |  |  |  |  |  |  |
|  |  |  | 50.3% |  |  |  |  |  |  |  |
|  |  |  | NR |  |  |  |  |  |  |  |
|  |  |  | NR |  |  |  |  |  |  |  |
| Al-Jazairi et al, 2019^18^ | Netherlands | Cohort study | 50 | SF-36 | / | / | / | AFSS | / | / |
|  |  |  | 57±9 years |  |  |  |  |  |  |  |
|  |  |  | 24% |  |  |  |  |  |  |  |
|  |  |  | NR |  |  |  |  |  |  |  |
|  |  |  | Paroxysmal (10%), persistent (68%) & long-standing persistent (22%) |  |  |  |  |  |  |  |
| Al-Kaisey et al, 2023^19^ | Australia | RCT | 96 | / | / | BDI-II & HADS | / | / | / | / |
|  |  |  | 59±12 years |  |  |  |  |  |  |  |
|  |  |  | 32% |  |  |  |  |  |  |  |
|  |  |  | NR |  |  |  |  |  |  |  |
|  |  |  | Ablation group (n=49): paroxysmal (47%) & persistent (53%)  Medical group (n=57): paroxysmal (62%) & persistent (38%) |  |  |  |  |  |  |  |
| Alli et al, 2013^20^ | USA & European nations | RCT | 547 | SF-12v2 | / | / | / | / | / | / |
|  |  |  | 72.1±9.0 years |  |  |  |  |  |  |  |
|  |  |  | 28.5% |  |  |  |  |  |  |  |
|  |  |  | Asian (0.4%), Black or African American (0.9%), Caucasian (92.7%), Hispanic or Latino (5.5%), Hawaiian or Pacific Islander (0.2%) & Other (0.4%) |  |  |  |  |  |  |  |
|  |  |  | Paroxysmal, persistent & permanent (n or % not specified) |  |  |  |  |  |  |  |
| Almeida et al, 2011^21^ | Brazil | Cross-sectional study | 72 | SF-36 [Brazilian Portuguese] | / | / | / | / | / | / |
|  |  |  | Age range reported: 22-87 years |  |  |  |  |  |  |  |
|  |  |  | 62.5% |  |  |  |  |  |  |  |
|  |  |  | Black (48.6%), White (20.8%), Mulatto (9.7%) & Other (20.8%) |  |  |  |  |  |  |  |
|  |  |  | NR |  |  |  |  |  |  |  |
| Althouse et al, 2023^22^ | USA | RCT (protocol) | 243 | AFEQT & PROMIS-29 | / | / | / | / | / | / |
|  |  |  | 70.7±9.8 years |  |  |  |  |  |  |  |
|  |  |  | 64.2% |  |  |  |  |  |  |  |
|  |  |  | Non-Hispanic White (66.3%) & Black (30.5%) |  |  |  |  |  |  |  |
|  |  |  | NR |  |  |  |  |  |  |  |
| Andrade et al, 2018^23^ | Canada | RCT (protocol) | 298 participants to be enrolled | AFEQT & EQ-5D | / | / | / | / | / | / |
|  |  |  | Participants aged ≥18 years to be enrolled |  |  |  |  |  |  |  |
|  |  |  | NR |  |  |  |  |  |  |  |
|  |  |  | NR |  |  |  |  |  |  |  |
|  |  |  | Paroxysmal or early persistent AF participants to be enrolled |  |  |  |  |  |  |  |
| Andrade et al, 2023^24^ | Canada | RCT | 346 | AFEQT & EQ-5D | / | / | / | / | / | / |
|  |  |  | No AF recurrence group (n=182): 57.2±9.8 years <2 min AF recurrence group (n=19): 64.7±11.5 years 2-60 minutes AF recurrence group (n=42): 61±8.4 years >1-6 hours AF recurrence group (n=50): 58.5±9.1 years >6-24 hours AF recurrence group (n=30): 59.5±10.9 years >24 hours AF recurrence group (n=23): 61.9±10.9 years |  |  |  |  |  |  |  |
|  |  |  | NR |  |  |  |  |  |  |  |
|  |  |  | NR |  |  |  |  |  |  |  |
|  |  |  | Paroxysmal (all participants) |  |  |  |  |  |  |  |
| Andrade et al, 2023 [a] ^25^ | Canada | RCT | 287 | AFEQT & EQ-5D | / | / | / | / | / | / |
|  |  |  | Antiarrhythmic group (n=141): 59.5±10.7 years & ablation group (n=146): 58.2±11.2 years |  |  |  |  |  |  |  |
|  |  |  | Antiarrhythmic group: 31.5% & ablation group: 27.3% |  |  |  |  |  |  |  |
|  |  |  | NR |  |  |  |  |  |  |  |
|  |  |  | Paroxysmal (all participants) |  |  |  |  |  |  |  |
| Andrade et al, 2021^26^ | Canada | RCT | 303 | AFEQT & EQ-5D | / | / | / | / | / | / |
|  |  |  | Antiarrhythmic group (n=149): 59.5±10.6 years & ablation group (n=154): 57.7±12.3 years |  |  |  |  |  |  |  |
|  |  |  | Antiarrhythmic group: 31.5% & ablation group: 27.3% |  |  |  |  |  |  |  |
|  |  |  | NR |  |  |  |  |  |  |  |
|  |  |  | Paroxysmal (all participants) |  |  |  |  |  |  |  |
| Antoun et al, 2024^27^ | Syria | Methodological study | 432 | EQ-5D [Arabic] & SF-36 [Arabic] | / | / | / | / | / | / |
|  |  |  | 63±15 years |  |  |  |  |  |  |  |
|  |  |  | 44% |  |  |  |  |  |  |  |
|  |  |  | NR |  |  |  |  |  |  |  |
|  |  |  | NR |  |  |  |  |  |  |  |
| Arbelo et al, 2021^28^ | NR | Mixed methods study | NR | AFEQT & SF-12 | / | / | / | AFSS | / | / |
|  |  |  | NR |  |  |  |  |  |  |  |
|  |  |  | NR |  |  |  |  |  |  |  |
|  |  |  | NR |  |  |  |  |  |  |  |
|  |  |  | NR |  |  |  |  |  |  |  |
| Ariansen et al, 2010^29^ | Norway | Case-control study | 27 with AF (total n=117) | SF-36 [Norwegian] | / | / | / | SCL | / | / |
|  |  |  | 75 years |  |  |  |  |  |  |  |
|  |  |  | Yes – fulfils Guidelines’ criteria group (n=6): 33% & no – does not fulfil Guidelines’ criteria group (n=21): 24% |  |  |  |  |  |  |  |
|  |  |  | NR |  |  |  |  |  |  |  |
|  |  |  | Permanent (all participants) |  |  |  |  |  |  |  |
| Ariansen et al, 2011^30^ | Norway | Case-control study | 27 with AF (total n=117) | SF-36 | / | HADS | / | / | / | / |
|  |  |  | 75 years |  |  |  |  |  |  |  |
|  |  |  | 26% |  |  |  |  |  |  |  |
|  |  |  | NR |  |  |  |  |  |  |  |
|  |  |  | Permanent (all participants) |  |  |  |  |  |  |  |
| Armentaro et al, 2025^31^ | Italy | Cohort study | 983 | / | / | / | MMSE | / | / | / |
|  |  |  | 76±6 years |  |  |  |  |  |  |  |
|  |  |  | 51% |  |  |  |  |  |  |  |
|  |  |  | NR |  |  |  |  |  |  |  |
|  |  |  | Paroxysmal (23%), persistent (13%) & permanent (64%) |  |  |  |  |  |  |  |
| Arribas et al, 2010^32^ | Spain | Methodological study | 341 with AF (total n=417) | AF-QoL & SF-36 | / | / | / | / | / | / |
|  |  |  | 60.7±12.4 years |  |  |  |  |  |  |  |
|  |  |  | 34.6% |  |  |  |  |  |  |  |
|  |  |  | NR |  |  |  |  |  |  |  |
|  |  |  | Paroxysmal, persistent & permanent (n or % not specified) |  |  |  |  |  |  |  |
| Arvanitis et al, 2020^33^ | Sweden | Cohort study | 43 | / | / | / | MMSE | / | / | / |
|  |  |  | 55±9.6 years |  |  |  |  |  |  |  |
|  |  |  | 16.3% |  |  |  |  |  |  |  |
|  |  |  | NR |  |  |  |  |  |  |  |
|  |  |  | First onset (39.5%), paroxysmal (39.5%) & persistent (20%) |  |  |  |  |  |  |  |
| Athreya et al, 2024^34^ | USA | Cohort study | 972 | / | PHQ-9 | / | MoCA | / | / | / |
|  |  |  | 75±6.8 years |  |  |  |  |  |  |  |
|  |  |  | Rate control group (n=408): 51.2% & rhythm control group (n=564): 48.2% |  |  |  |  |  |  |  |
|  |  |  | Rate control group: non-Hispanic White (86.2%) & not specified (13.8%) Rhythm control group: non-Hispanic White (87.8%) & not specified (12.2%) |  |  |  |  |  |  |  |
|  |  |  | Rate control group: paroxysmal (63%) & persistent (37%)  Rhythm control group: paroxysmal (69.9%) & persistent (30.1%) |  |  |  |  |  |  |  |
| Badia et al, 2007^35^ | Spain | Methodological study | 112 | AF-QoL | / | / | / | / | / | / |
|  |  |  | 60.52±13.43 years |  |  |  |  |  |  |  |
|  |  |  | 35.7% |  |  |  |  |  |  |  |
|  |  |  | NR |  |  |  |  |  |  |  |
|  |  |  | Permanent (47.3%) & paroxysmal (52.7%) |  |  |  |  |  |  |  |
| Bagge et al, 2009^36^ | NR | Cohort study | 43 | SF-36 | / | / | / | / | / | / |
|  |  |  | Median age reported: 58 (IQR range 36-71) years |  |  |  |  |  |  |  |
|  |  |  | 33% |  |  |  |  |  |  |  |
|  |  |  | NR |  |  |  |  |  |  |  |
|  |  |  | Paroxysmal (65%), persistent (14%) & permanent (21%) |  |  |  |  |  |  |  |
| Bagge et al, 2018^37^ | Sweden | RCT | 65 | SF-36 | / | / | / | / | / | / |
|  |  |  | Cryoablation and mitral valve surgery group (n=30): 69.5±7.9 years & mitral valve surgery group (n=35): 65.6±8.8 years |  |  |  |  |  |  |  |
|  |  |  | Cryoablation and mitral valve surgery group: 16.7% & mitral valve surgery group: 25.7% |  |  |  |  |  |  |  |
|  |  |  | NR |  |  |  |  |  |  |  |
|  |  |  | Permanent (all participants) |  |  |  |  |  |  |  |
| Bai et al, 2015^38^ | China | Cohort study | 222 | AFEQT [Chinese] | / | / | / | / | / | / |
|  |  |  | Radiofrequency ablation group (n=74): 61.82±8.90 years & non-radiofrequency ablation group (n=148): 62.42±10.52 years |  |  |  |  |  |  |  |
|  |  |  | Radiofrequency ablation group: 36.49% & non-radiofrequency ablation group: 37.16% |  |  |  |  |  |  |  |
|  |  |  | NR |  |  |  |  |  |  |  |
|  |  |  | Radiofrequency ablation group: paroxysmal (70.27%) & non-paroxysmal (29.73%)  Non-radiofrequency ablation group: paroxysmal (54.73%) & non-paroxysmal (45.27%) |  |  |  |  |  |  |  |
| Bakker et al, 2013^39^ | Netherlands | Cohort study | 169 | SF-36 [Dutch] | / | / | / | / | / | / |
|  |  |  | 63.7±11.0 years |  |  |  |  |  |  |  |
|  |  |  | 43.8% |  |  |  |  |  |  |  |
|  |  |  | NR |  |  |  |  |  |  |  |
|  |  |  | Paroxysmal (35.5%) & long-standing persistent (64.5%) |  |  |  |  |  |  |  |
| Baldus et al, 2023^40^ | Germany | Cohort study (protocol) | 720 participants to be enrolled | EQ-5D-5L | / | / | / | / | / | / |
|  |  |  | Participants aged ≥18 years to be enrolled |  |  |  |  |  |  |  |
|  |  |  | NR |  |  |  |  |  |  |  |
|  |  |  | NR |  |  |  |  |  |  |  |
|  |  |  | Non-valvular AF participants to be enrolled |  |  |  |  |  |  |  |
| Ball et al, 2013^41^ | Australia | RCT | 260 | / | / | / | MoCA | / | / | / |
|  |  |  | 72±11 years |  |  |  |  |  |  |  |
|  |  |  | 47% |  |  |  |  |  |  |  |
|  |  |  | NR |  |  |  |  |  |  |  |
|  |  |  | Paroxysmal (4%), persistent (86%) & permanent (10%) |  |  |  |  |  |  |  |
| Ballard et al, 2015^42^ | USA | Cohort study | 652 | AFEQT | / | / | / | / | / | / |
|  |  |  | Grouped ages reported: 18-48 years (11.2% of participants), 50-59 years (18.9% of participants), 60-69 years (28.7% of participants), 70-79 years (25.9% of participants) & ≥80 years (15.3% of participants) |  |  |  |  |  |  |  |
|  |  |  | 47.9% |  |  |  |  |  |  |  |
|  |  |  | NR |  |  |  |  |  |  |  |
|  |  |  | Paroxysmal (83.9%), chronic (2.8%) & unclear (13.3%) |  |  |  |  |  |  |  |
| Bang et al, 2023^43^ | South Korea | Descriptive survey study | 175 | SF-36v2 [Korean] | / | CES-D [Korean] & STAI [Korean] | / | SCL | / | / |
|  |  |  | 61.55±11.50 years |  |  |  |  |  |  |  |
|  |  |  | 25.1% |  |  |  |  |  |  |  |
|  |  |  | NR |  |  |  |  |  |  |  |
|  |  |  | Paroxysmal (62.3%) & persistent (37.7%) |  |  |  |  |  |  |  |
| Bamgbade et al, 2020^44^ | USA | Cohort study | 1,244 | AFEQT | / | GAD-7 & PHQ-9 | MoCA | / | / | / |
|  |  |  | 75.5±7.1 years |  |  |  |  |  |  |  |
|  |  |  | 49% |  |  |  |  |  |  |  |
|  |  |  | Non-Hispanic White (86%) & not specified (14%) |  |  |  |  |  |  |  |
|  |  |  | NR |  |  |  |  |  |  |  |
| Barber et al, 2004^45^ | NR | Cohort study | 258 | / | OARS | GDS-5 | TICS-m | / | / | / |
|  |  |  | Median age reported: 72 (IQR range 66-78) years |  |  |  |  |  |  |  |
|  |  |  | 54% |  |  |  |  |  |  |  |
|  |  |  | NR |  |  |  |  |  |  |  |
|  |  |  | Paroxysmal (21%) & not specified (79%) |  |  |  |  |  |  |  |
| Barbagallo et al, 2025^46^ | Switzerland | Cohort study | 2,413 | / | / | / | MoCA | / | / | / |
|  |  |  | 73.25±8.42 years |  |  |  |  |  |  |  |
|  |  |  | 27.4% |  |  |  |  |  |  |  |
|  |  |  | NR |  |  |  |  |  |  |  |
|  |  |  | NR |  |  |  |  |  |  |  |
| Barmano et al, 2019^47^ | Sweden | Cohort study | 192 | ASTA HRQoL scale & SF-36 | / | / | / | ASTA symptom scale | / | / |
|  |  |  | 60.5±10.2 years |  |  |  |  |  |  |  |
|  |  |  | 29% |  |  |  |  |  |  |  |
|  |  |  | NR |  |  |  |  |  |  |  |
|  |  |  | Paroxysmal (37%) & persistent (63%) |  |  |  |  |  |  |  |
| Barmano et al, 2016^48^ | Sweden | Cohort study | 322 | ASTA HRQoL scale, EQ-5D, EQ-VAS & SF-36 [Swedish] | / | HADS | / | ASTA symptom scale | / | / |
|  |  |  | Intervention group (n=176): 66±10 years & control group (n=146): 68±11years |  |  |  |  |  |  |  |
|  |  |  | Intervention group: 36% & control group: 42% |  |  |  |  |  |  |  |
|  |  |  | NR |  |  |  |  |  |  |  |
|  |  |  | Intervention group: first episode (24%), paroxysmal (35%), persistent (32%) & permanent (9%)  Control group: first episode (35%), paroxysmal (34%), persistent (26%) & permanent (5%) |  |  |  |  |  |  |  |
| Barrios et al, 2019^49^ | Spain | Cohort study | 965 | AF-QoL | / | / | / | / | / | / |
|  |  |  | 73.3±9.4 years |  |  |  |  |  |  |  |
|  |  |  | 43% |  |  |  |  |  |  |  |
|  |  |  | NR |  |  |  |  |  |  |  |
|  |  |  | Paroxysmal (35.1%), persistent (28.6%) & permanent (36.4%) |  |  |  |  |  |  |  |
| Bazemore et al, 2019^50^ | USA | Cohort study | 191 | AFEQT | DASI | / | / | / | / | / |
|  |  |  | 63.7±9.5 years |  |  |  |  |  |  |  |
|  |  |  | 27.2% |  |  |  |  |  |  |  |
|  |  |  | NR |  |  |  |  |  |  |  |
|  |  |  | Paroxysmal (43%), persistent (50.8%) & long-standing persistent (6.3%) |  |  |  |  |  |  |  |
| Beaver et al, 2016^51^ | NR | Pilot RCT | 23 | SF-36 | / | / | / | / | / | / |
|  |  |  | Thoracoscopic pulmonary vein isolation and atrial appendage ligation group (n=12): 67± 9.4years & medically managed group (n=11): 68± 8.4 years |  |  |  |  |  |  |  |
|  |  |  | 39.13% |  |  |  |  |  |  |  |
|  |  |  | NR |  |  |  |  |  |  |  |
|  |  |  | Thoracoscopic pulmonary vein isolation and atrial appendage ligation group: paroxysmal (42%), persistent or long-standing persistent (58%)  Medically managed group: paroxysmal (55%), persistent or long-standing persistent (45%) |  |  |  |  |  |  |  |
| Benzimra et al, 2018^52^ | France | Cross-sectional study | 200 | EQ-5D-3L [French] | / | / | / | / | / | / |
|  |  |  | 74.3±10.1 years |  |  |  |  |  |  |  |
|  |  |  | 41% |  |  |  |  |  |  |  |
|  |  |  | NR |  |  |  |  |  |  |  |
|  |  |  | Non-valvular (all participants) |  |  |  |  |  |  |  |
| Berg et al, 2010^53^ | 35 European countries (not specified) | Survey study | 5,050 | EQ-5D | / | / | / | / | / | / |
|  |  |  | 66.4±12.8 years |  |  |  |  |  |  |  |
|  |  |  | 41.9% |  |  |  |  |  |  |  |
|  |  |  | NR |  |  |  |  |  |  |  |
|  |  |  | First detected (19.1%), paroxysmal (29.1%), persistent (22.7%) & permanent (29.1%) |  |  |  |  |  |  |  |
| Berger et al, 2016^54^ | NR | Cohort study | 99 | SF-36 | / | / | / | / | / | / |
|  |  |  | 55.4±8.9 years |  |  |  |  |  |  |  |
|  |  |  | 25% |  |  |  |  |  |  |  |
|  |  |  | NR |  |  |  |  |  |  |  |
|  |  |  | Paroxysmal (91.5%) & persistent (8.5%) |  |  |  |  |  |  |  |
| Berkowitsch et al, 2003^55^ | NR | Cohort study | 60 | SF-36 | / | / | / | SCL | / | / |
|  |  |  | 58±11 years |  |  |  |  |  |  |  |
|  |  |  | 35% |  |  |  |  |  |  |  |
|  |  |  | NR |  |  |  |  |  |  |  |
|  |  |  | Paroxysmal (all participants) |  |  |  |  |  |  |  |
| Berry et al, 2001^56^ | UK | Cohort study | 111 | EQ-VAS | / | / | / | / | / | / |
|  |  |  | 66.8±11 years |  |  |  |  |  |  |  |
|  |  |  | 33% |  |  |  |  |  |  |  |
|  |  |  | NR |  |  |  |  |  |  |  |
|  |  |  | NR |  |  |  |  |  |  |  |
| Bittman et al, 2022^57^ | Canada | RCT | 72 | SF-36 | / | GAD-7 & PHQ-8 | / | AFSS | / | / |
|  |  |  | 62±9.2 years |  |  |  |  |  |  |  |
|  |  |  | 39% |  |  |  |  |  |  |  |
|  |  |  | European (62.5%), East Asian (16.7%), South Asian (6.9%), Southeast Asian (4.2%), Latin American (2.8%) & did not provide race or ethnicity (6.9%) |  |  |  |  |  |  |  |
|  |  |  | Control group (n=38): paroxysmal (92.1%) & persistent (7.9%)  Intervention group (n=34): paroxysmal (85.3%) & persistent (14.7%) |  |  |  |  |  |  |  |
| Biviano et al, 2017^58^ | USA | Cohort study (registry) | 736 | AFEQT | / | / | / | / | / | / |
|  |  |  | Older patients group (n=381): 71.1±4.9 years & younger patients group (n=355): 53.6±9.1 years |  |  |  |  |  |  |  |
|  |  |  | 38.6% |  |  |  |  |  |  |  |
|  |  |  | NR |  |  |  |  |  |  |  |
|  |  |  | Paroxysmal (all participants) |  |  |  |  |  |  |  |
| Björkenheim et al, 2018^59^ | Scandinavian country (not specified) | Cohort study | 57 | SF-36 | / | / | / | AF6 | / | / |
|  |  |  | 57±9 years |  |  |  |  |  |  |  |
|  |  |  | 40% |  |  |  |  |  |  |  |
|  |  |  | NR |  |  |  |  |  |  |  |
|  |  |  | Paroxysmal (88%) & persistent (12%) |  |  |  |  |  |  |  |
| Björkenheim et al, 2017^60^ | Scandinavian country (not specified) | Cohort study | 57 | / | / | / | / | AF6 | / | / |
|  |  |  | 57±9 years |  |  |  |  |  |  |  |
|  |  |  | 40% |  |  |  |  |  |  |  |
|  |  |  | NR |  |  |  |  |  |  |  |
|  |  |  | Paroxysmal (88%) & persistent (12%) |  |  |  |  |  |  |  |
| Blandino et al, 2013^61^ | NR | Cohort study | 412 | SF-36 | / | / | / | / | / | / |
|  |  |  | Group A (n=153): 75±5 years & group B (n=259): 76±5 years |  |  |  |  |  |  |  |
|  |  |  | Group A: 29% & group B: 28% |  |  |  |  |  |  |  |
|  |  |  | NR |  |  |  |  |  |  |  |
|  |  |  | Persistent (all participants) |  |  |  |  |  |  |  |
| Blomström-Lundqvist et al, 2019^62^ | Finland & Sweden | RCT | 155 | SF-36 | / | / | / | / | / | / |
|  |  |  | 56.1 years |  |  |  |  |  |  |  |
|  |  |  | 22.6% |  |  |  |  |  |  |  |
|  |  |  | NR |  |  |  |  |  |  |  |
|  |  |  | Ablation group (n=79): paroxysmal (70.9%) & persistent (29.1%) Medication group (n=76): paroxysmal (75%) & persistent (25%) |  |  |  |  |  |  |  |
| Bo et al, 2016^63^ | Italy | Cohort study | 980 | / | ADL & IADL | / | SPMSQ | / | / | / |
|  |  |  | 83.4±6.7 years |  |  |  |  |  |  |  |
|  |  |  | 60.5% |  |  |  |  |  |  |  |
|  |  |  | NR |  |  |  |  |  |  |  |
|  |  |  | Permanent (73.5%) & not specified (26.5%) |  |  |  |  |  |  |  |
| Boehmer et al, 2024^64^ | Austria & Germany | RCT (protocol) | 196 participants to be enrolled | AFEQT | / | / | / | / | / | / |
|  |  |  | Participants aged ≥75 years to be enrolled |  |  |  |  |  |  |  |
|  |  |  | NR |  |  |  |  |  |  |  |
|  |  |  | NR |  |  |  |  |  |  |  |
|  |  |  | Persistent AF participants to be enrolled |  |  |  |  |  |  |  |
| Boersma et al, 2020^65^ | France, Germany, Greece, Hungary, Italy, Netherlands, Poland, Portugal, Spain, Switzerland, UK, Georgia, Israel & South Korea | Cohort study (registry) | 1,054 | AFEQT | / | / | / | / | / | / |
|  |  |  | 32.4% |  |  |  |  |  |  |  |
|  |  |  | 60.6±10.9 years |  |  |  |  |  |  |  |
|  |  |  | NR |  |  |  |  |  |  |  |
|  |  |  | Paroxysmal (70.2%), persistent (26.5%) & long-standing persistent (3.3%) |  |  |  |  |  |  |  |
| Borland et al, 2022^66^ | NR | RCT | 38 | SF-36 [Swedish] | / | / | / | / | IPAQ-SF [Swedish] & SGPALS | / |
|  |  |  | 74±4 years |  |  |  |  |  |  |  |
|  |  |  | 26.3% |  |  |  |  |  |  |  |
|  |  |  | NR |  |  |  |  |  |  |  |
|  |  |  | Permanent (all participants) |  |  |  |  |  |  |  |
| Bostrom et al, 2017^67^ | USA | Cohort study | 218 | AFEQT | / | GAD-7 & PHQ-9 | MoCA | / | / | / |
|  |  |  | 63.5±10.2 years |  |  |  |  |  |  |  |
|  |  |  | 64.9% |  |  |  |  |  |  |  |
|  |  |  | White (93.9%) & not specified (6.1%) |  |  |  |  |  |  |  |
|  |  |  | Paroxysmal (81%) & not specified (19%) |  |  |  |  |  |  |  |
| Boveda et al, 2018^68^ | France, Germany & Greece | RCT | 101 | SF-36v2 | / | / | / | / | / | / |
|  |  |  | 61.8±10.5 years |  |  |  |  |  |  |  |
|  |  |  | 25.7% |  |  |  |  |  |  |  |
|  |  |  | NR |  |  |  |  |  |  |  |
|  |  |  | Persistent (all participants) |  |  |  |  |  |  |  |
| Bowyer et al, 2017^69^ | NR | RCT | 41 | SF-36 | / | / | / | SCL | / | / |
|  |  |  | 62.1±10.6 years |  |  |  |  |  |  |  |
|  |  |  | 34.1% |  |  |  |  |  |  |  |
|  |  |  | NR |  |  |  |  |  |  |  |
|  |  |  | Paroxysmal (69%) & non-paroxysmal (31%) |  |  |  |  |  |  |  |
| Boyalla et al, 2024^70^ | UK | RCT | 104 | AFEQT & EQ-5D-5L | / | / | / | / | / | / |
|  |  |  | 63±10 years |  |  |  |  |  |  |  |
|  |  |  | Catheter ablation group (n=57): 26% & surgical ablation group (n=47): 28% |  |  |  |  |  |  |  |
|  |  |  | Catheter ablation group: White (94.7%), Asian (3.5%), Black (0%) & Middle Eastern (1.8%) Surgical ablation group: White (93.6%), Asian (0%), Black (4.3%) & Middle Eastern (2.1%) |  |  |  |  |  |  |  |
|  |  |  | Persistent (all participants) |  |  |  |  |  |  |  |
| Braganca et al, 2010^71^ | Brazil | Methodological study | 63 | SF-36 [Brazilian Portuguese] & QLAF | / | / | / | / | / | / |
|  |  |  | 62.8±12.2 years |  |  |  |  |  |  |  |
|  |  |  | 42.9% |  |  |  |  |  |  |  |
|  |  |  | NR |  |  |  |  |  |  |  |
|  |  |  | Paroxysmal (24%), persistent (20%) & permanent (19%) |  |  |  |  |  |  |  |
| Bubien et al, 1996^72^ | USA | Cohort study | 22 with AF (total n=159) | SF-36 | ADL | / | / | SCL | / | / |
|  |  |  | 61.43±11.77 years |  |  |  |  |  |  |  |
|  |  |  | 72.7% |  |  |  |  |  |  |  |
|  |  |  | NR |  |  |  |  |  |  |  |
|  |  |  | NR |  |  |  |  |  |  |  |
| Buist et al, 2020^73^ | Netherlands | RCT | 52 | SF-36 | / | / | / | / | / | / |
|  |  |  | Median age reported: catheter ablation group (n=25): 59 (IQR range 55-66) years & minimally invasive thoracoscopic pulmonary vein isolation (n=25): 55 (IQR range 47-62) years |  |  |  |  |  |  |  |
|  |  |  | 22% |  |  |  |  |  |  |  |
|  |  |  | NR |  |  |  |  |  |  |  |
|  |  |  | Paroxysmal (74%) & not specified (26%) |  |  |  |  |  |  |  |
| Bulková et al, 2014^74^ | NR | Cohort study | 387 | EQ-5D & EQ-VAS | / | / | / | / | / | / |
|  |  |  | Persistent AF group (n=261): 57±10 years & long-standing persistent AF group (n=126): 59±9 years |  |  |  |  |  |  |  |
|  |  |  | Persistent AF group: 33% & long-standing persistent AF group: 21% |  |  |  |  |  |  |  |
|  |  |  | NR |  |  |  |  |  |  |  |
|  |  |  | Persistent (67.4%) & long-standing persistent (32.6%) |  |  |  |  |  |  |  |
| Bunch et al, 2022^75^ | USA | RCT | 101 | / | / | / | MMSE | / | / | / |
|  |  |  | 73.7±6.0 years |  |  |  |  |  |  |  |
|  |  |  | 46.5% |  |  |  |  |  |  |  |
|  |  |  | Dabigatran group (n=50): White (100%) & warfarin group (n=51): White (98%) & not specified (2%) |  |  |  |  |  |  |  |
|  |  |  | Non-valvular (all participants) |  |  |  |  |  |  |  |
| Burns et al, 2004^76^ | USA | Cross-sectional study | 96 | SF-36 | / | CES-D & STAI | / | SCL | / | / |
|  |  |  | 62±11 years |  |  |  |  |  |  |  |
|  |  |  | 28% |  |  |  |  |  |  |  |
|  |  |  | NR |  |  |  |  |  |  |  |
|  |  |  | NR |  |  |  |  |  |  |  |
| Caramelli et al, 2022^77^ | Brazil | RCT | 149 | / | / | / | MMSE & MoCA | / | / | / |
|  |  |  | Median age reported: dabigatran group (n=83): 74 (IQR range 71-77) years & warfarin group (n=66): 76 (IQR range 72-77) years |  |  |  |  |  |  |  |
|  |  |  | Dabigatran group: 38.6% & warfarin group: 40.9% |  |  |  |  |  |  |  |
|  |  |  | NR |  |  |  |  |  |  |  |
|  |  |  | NR |  |  |  |  |  |  |  |
| Carnlöf et al, 2020^78^ | Sweden | Methodological study | 338 with AF (total n=646) | / | / | / | / | SCL [Swedish] | / | / |
|  |  |  | 61.4±1.5 years |  |  |  |  |  |  |  |
|  |  |  | 25.1% (with AF) |  |  |  |  |  |  |  |
|  |  |  | NR |  |  |  |  |  |  |  |
|  |  |  | NR |  |  |  |  |  |  |  |
| Caceres et al, 2020^79^ | USA | RCT | 238 | AFEQT, EQ-5D & SF-36 | / | / | / | AFSS | / | / |
|  |  |  | Intervention group (n=115): 61.4±11.9 years & control group (n=123): 61.2±11.8 years |  |  |  |  |  |  |  |
|  |  |  | 23% |  |  |  |  |  |  |  |
|  |  |  | Intervention group: White (77%), Black (3%), Asian (1%) & Unknown (20%)  Control group: White (76%), Black (7%), Asian (4%) & Unknown (14%) |  |  |  |  |  |  |  |
|  |  |  | NR |  |  |  |  |  |  |  |
| Calò et al, 2004^80^ | Italy | Cohort study | 74 | SF-36 [Italian] | / | / | / | SCL | / | / |
|  |  |  | 57±7 years |  |  |  |  |  |  |  |
|  |  |  | 28.4% |  |  |  |  |  |  |  |
|  |  |  | NR |  |  |  |  |  |  |  |
|  |  |  | Paroxysmal (66.2%) & permanent (33.8%) |  |  |  |  |  |  |  |
| Cannavan et al, 2020^81^ | Brazil | Methodological study | 71 with AF (total n=140) | ASTA HRQoL scale [Brazilian Portuguese] & QLAFv2 | / | / | SPMSQ | ASTA symptom scale [Brazilian Portuguese] | / | / |
|  |  |  | 57.2±13.1 years |  |  |  |  |  |  |  |
|  |  |  | 55% |  |  |  |  |  |  |  |
|  |  |  | NR |  |  |  |  |  |  |  |
|  |  |  | NR |  |  |  |  |  |  |  |
| Carlsson et al, 2003^82^ | Germany | Pilot RCT | 200 | SF-36 [German] | / | / | / | / | / | / |
|  |  |  | Rhythm control group (n=100): 65.3±9.4 years & rate control group (n=100): 66.2±7.6 years |  |  |  |  |  |  |  |
|  |  |  | Rhythm control group: 41% & rate control group: 32% |  |  |  |  |  |  |  |
|  |  |  | NR |  |  |  |  |  |  |  |
|  |  |  | Persistent (all participants) |  |  |  |  |  |  |  |
| Carnlöf et al, 2010^83^ | Sweden | Cohort study | 40 | SF-36 [Swedish] | / | / | / | / | / | / |
|  |  |  | 53±9 years |  |  |  |  |  |  |  |
|  |  |  | 25% |  |  |  |  |  |  |  |
|  |  |  | NR |  |  |  |  |  |  |  |
|  |  |  | Paroxysmal & persistent (n or % not specified) |  |  |  |  |  |  |  |
| Carnlöf et al, 2022^84^ | Swedish | Cohort study | 242 | SF-36 [Swedish] & SIP [Swedish] | / | / | / | SCL [Swedish] | / | / |
|  |  |  | 62±9 years |  |  |  |  |  |  |  |
|  |  |  | 50% |  |  |  |  |  |  |  |
|  |  |  | NR |  |  |  |  |  |  |  |
|  |  |  | Paroxysmal (74%) & persistent (26%) |  |  |  |  |  |  |  |
| Castro-Urda et al, 2023^85^ | NR | RCT | 100 | EQ-5D-5L & EQ-VAS | / | / | / | / | / | / |
|  |  |  | Median age reported: 64 (IQR range 56.25–69) years |  |  |  |  |  |  |  |
|  |  |  | 31% |  |  |  |  |  |  |  |
|  |  |  | NR |  |  |  |  |  |  |  |
|  |  |  | Paroxysmal (57%) & persistent (43%) |  |  |  |  |  |  |  |
| Cha et al, 2008^86^ | USA | Cohort study | 523 | SF-36 | / | / | / | / | / | / |
|  |  |  | 54±10 years |  |  |  |  |  |  |  |
|  |  |  | 16% |  |  |  |  |  |  |  |
|  |  |  | NR |  |  |  |  |  |  |  |
|  |  |  | Paroxysmal (58%) & persistent or permanent (42%) |  |  |  |  |  |  |  |
| Chandan et al, 2024^87^ | UK | RCT (protocol) | 106 participants to be enrolled | AFEQT & SF-36 | / | / | / | / | / | / |
|  |  |  | Participants aged ≥18 years to be enrolled |  |  |  |  |  |  |  |
|  |  |  | NR |  |  |  |  |  |  |  |
|  |  |  | NR |  |  |  |  |  |  |  |
|  |  |  | Paroxysmal or persistent AF participants to be enrolled |  |  |  |  |  |  |  |
| Chapa et al, 2014^88^ | USA & Canada | RCT | 693 | SF-36 & QLI-CV | / | / | / | / | / | / |
|  |  |  | Women group (n=262): 72.02±7.46 years & men group (n=431): 68.49±8.34 years |  |  |  |  |  |  |  |
|  |  |  | 37.8% |  |  |  |  |  |  |  |
|  |  |  | NR |  |  |  |  |  |  |  |
|  |  |  | NR |  |  |  |  |  |  |  |
| Charitakis et al, 2015^89^ | Sweden | Mixed methods study (protocol) | 200 participants to be enrolled | ASTA HRQoL scale & SF-36 | / | HADS | / | ASTA symptom scale | / | / |
|  |  |  | Participants aged >17 years to be enrolled |  |  |  |  |  |  |  |
|  |  |  | NR |  |  |  |  |  |  |  |
|  |  |  | NR |  |  |  |  |  |  |  |
|  |  |  | Paroxysmal or persistent AF participants to be enrolled |  |  |  |  |  |  |  |
| Chen et al, 2023^90^ | NR | RCT | 100 | / | / | / | MoCA | / | / | / |
|  |  |  | Median age reported: High-power short-duration ablation group (n=50): 63 (IQR range 52-70) years & conventional group (n=50): 63 (IQR range 55-70) years |  |  |  |  |  |  |  |
|  |  |  | 40% |  |  |  |  |  |  |  |
|  |  |  | NR |  |  |  |  |  |  |  |
|  |  |  | High-power short-duration ablation group: paroxysmal (66%) & persistent (34%)  Conventional group: paroxysmal (52%) & persistent (48%) |  |  |  |  |  |  |  |
| Chen et al, 2021^91^ | USA | Cohort study | 339 | AFEQT | / | / | / | / | / | / |
|  |  |  | 72.3±10.1 years |  |  |  |  |  |  |  |
|  |  |  | 42.5% |  |  |  |  |  |  |  |
|  |  |  | White (94.4%) & not specified (5.6%) |  |  |  |  |  |  |  |
|  |  |  | Non-valvular (all participants) |  |  |  |  |  |  |  |
| Cherian et al, 2017^92^ | USA | Cohort study (registry) | 9,743 | AFEQT | / | / | / | / | / | / |
|  |  |  | Median age reported: no heart failure group (n=6,540): 74 (IQR range 66-81) years & heart failure group (n=3,203): 76 (IQR 68-83) years |  |  |  |  |  |  |  |
|  |  |  | No heart failure group: 44% & heart failure group: 40% |  |  |  |  |  |  |  |
|  |  |  | NR |  |  |  |  |  |  |  |
|  |  |  | No heart failure group: new onset (5%), paroxysmal (55%), persistent (15%) & permanent (24%)  Heart failure group: new onset (3%), paroxysmal (42%), persistent (20%) & permanent (36%) |  |  |  |  |  |  |  |
| Chernyavskiy et al, 2016^93^ | NR | RCT | 95 | SF-36 | / | / | / | / | / | / |
|  |  |  | Group 1 (n=31): 64±6.97 years, group 2 (n=30): 62.1±7.09 years & group 3: 63.5±7.98 years |  |  |  |  |  |  |  |
|  |  |  | Group 1: 22.6%, group 2: 16.7% & group 3: 26.5% |  |  |  |  |  |  |  |
|  |  |  | NR |  |  |  |  |  |  |  |
|  |  |  | Persistent (all participants) |  |  |  |  |  |  |  |
| Chieng et al, 2023^94^ | Australia | RCT | 31 | AFEQT | / | / | MMSE | / | / | / |
|  |  |  | 66±8 years |  |  |  |  |  |  |  |
|  |  |  | 51.6% |  |  |  |  |  |  |  |
|  |  |  | NR |  |  |  |  |  |  |  |
|  |  |  | Persistent (80.6%) & paroxysmal (19.4%) |  |  |  |  |  |  |  |
| Chung et al, 2005^95^ | Canada & USA | RCT | 4,060 | / | / | / | MMSE | / | / | / |
|  |  |  | 69.7±9.0 years |  |  |  |  |  |  |  |
|  |  |  | 39% |  |  |  |  |  |  |  |
|  |  |  | Ethnic minority group (11%) |  |  |  |  |  |  |  |
|  |  |  | NR |  |  |  |  |  |  |  |
| Clarkesmith et al, 2013^96^ | UK | RCT | 97 | AF-QoL | / | HADS | / | / | / | / |
|  |  |  | 72.9±8.2 years |  |  |  |  |  |  |  |
|  |  |  | 35.1% |  |  |  |  |  |  |  |
|  |  |  | White (99%) & not specified (1%) |  |  |  |  |  |  |  |
|  |  |  | Paroxysmal (30.9%), persistent (22.6%) & permanent (24.7%) |  |  |  |  |  |  |  |
| Conde et al, 2017^97^ | Argentina | Cohort study | 80 | EQ-5D [Argentinian Spanish] | / | / | / | / | / | / |
|  |  |  | Median age reported: 63 (IQR range 54–69.5) years |  |  |  |  |  |  |  |
|  |  |  | 32.5% |  |  |  |  |  |  |  |
|  |  |  | NR |  |  |  |  |  |  |  |
|  |  |  | NR |  |  |  |  |  |  |  |
| Conen et al, 2017^98^ | Switzerland | Cohort study (protocol) | 2,133 participants already enrolled | / | / | / | MoCA | / | / | / |
|  |  |  | Participants aged ≥65 years participants to be enrolled |  |  |  |  |  |  |  |
|  |  |  | NR |  |  |  |  |  |  |  |
|  |  |  | NR |  |  |  |  |  |  |  |
|  |  |  | Paroxysmal, persistent or permanent AF participants to be enrolled |  |  |  |  |  |  |  |
| Conen et al, 2019^99^ | Switzerland | Cohort study | 1,737 | / | / | / | MoCA | / | / | / |
|  |  |  | 73±8 years |  |  |  |  |  |  |  |
|  |  |  | 28% |  |  |  |  |  |  |  |
|  |  |  | NR |  |  |  |  |  |  |  |
|  |  |  | Paroxysmal (46%), persistent (30%) & permanent (24%) |  |  |  |  |  |  |  |
| Contreras et al, 2017^100^ | Spain | Cross-sectional study | 1,337 | EQ-5D-3L | / | / | / | / | / | / |
|  |  |  | 75.0±8.9 years |  |  |  |  |  |  |  |
|  |  |  | 44.2% |  |  |  |  |  |  |  |
|  |  |  | NR |  |  |  |  |  |  |  |
|  |  |  | Paroxysmal (26.5%), persistent (6.2%) & permanent (70.3%) |  |  |  |  |  |  |  |
| Cooper et al, 2004^101^ | USA & Canada | RCT | 680 | SF-36 & QLI | / | / | / | SCL | / | / |
|  |  |  | 70.5±8.8 years |  |  |  |  |  |  |  |
|  |  |  | 36% |  |  |  |  |  |  |  |
|  |  |  | White (90%) & not specified (10%) |  |  |  |  |  |  |  |
|  |  |  | First episode (30%) & not specified (70%) |  |  |  |  |  |  |  |
| Corino et al, 2015^102^ | Norway | RCT | 38 | / | / | / | / | SCL [Norwegian] | / | / |
|  |  |  | 71±9 years |  |  |  |  |  |  |  |
|  |  |  | 21.1% |  |  |  |  |  |  |  |
|  |  |  | NR |  |  |  |  |  |  |  |
|  |  |  | Permanent (all participants) |  |  |  |  |  |  |  |
| Cosansu et al, 2019^103^ | Turkey | Cross-sectional study | 100 | / | / | HADS | / | / | / | / |
|  |  |  | 70.2±8.7 years |  |  |  |  |  |  |  |
|  |  |  | 65% |  |  |  |  |  |  |  |
|  |  |  | NR |  |  |  |  |  |  |  |
|  |  |  | Non-valvular (all participants) |  |  |  |  |  |  |  |
| Coyne et al, 2017^104^ | Japan, centres in European countries (not specified) & USA | Methodological study | 91 | AFImpact & SF-36 | / | / | / | AFSS | / | / |
|  |  |  | Reported as: 73% participants aged ≥60 years |  |  |  |  |  |  |  |
|  |  |  | 34.1% |  |  |  |  |  |  |  |
|  |  |  | NR |  |  |  |  |  |  |  |
|  |  |  | Paroxysmal (33%), persistent (33%) & permanent (34%) |  |  |  |  |  |  |  |
| Crowley et al, 2024^105^ | Australia, Canada & UK | RCT | 333 | AFEQT | / | / | / | / | / | / |
|  |  |  | Median age reported: AF recurrence group (n=154): 67.3 (IQR range 60.4-72.7) years & no AF recurrence group (n=179): 64.3 (IQR range 57.6-70.2) years |  |  |  |  |  |  |  |
|  |  |  | AF recurrence group: 27.9% & no AF recurrence group: 18.4% |  |  |  |  |  |  |  |
|  |  |  | AF recurrence group: Caucasian (97.4%) & not specified (2.6%) & no AF recurrence group: Caucasian (97.8%) & not specified (2.2%) |  |  |  |  |  |  |  |
|  |  |  | Persistent (all participants) |  |  |  |  |  |  |  |
| Dąbrowski et al, 2010^106^ | Poland | Cohort study | 150 | NHP [Polish] | / | BDI [Polish] | / | / | / | / |
|  |  |  | Women group (n=55): 67.8±10.5 years & men group (n=95): 68.4±9.4 years |  |  |  |  |  |  |  |
|  |  |  | 36.7% |  |  |  |  |  |  |  |
|  |  |  | NR |  |  |  |  |  |  |  |
|  |  |  | Paroxysmal (40.7%), persistent (30.7%) & permanent (28.6%) |  |  |  |  |  |  |  |
| Dagres et al, 2007^107^ | 35 European countries (not specified) | Cohort study | 5,333 | EQ-5D & EQ-VAS | / | / | / | / | / | / |
|  |  |  | Women group (n=2,249): 70±12 years & men group (n=3,084): 64±13 years |  |  |  |  |  |  |  |
|  |  |  | 42% |  |  |  |  |  |  |  |
|  |  |  | NR |  |  |  |  |  |  |  |
|  |  |  | Women group: first detected (19%), paroxysmal (30%), persistent (21%) & permanent (31%)  Men group: first detected (19%), paroxysmal (29%), persistent (24%) & permanent (29%) |  |  |  |  |  |  |  |
| Das et al, 2017^108^ | UK | RCT | 80 | AFEQT | / | / | / | / | / | / |
|  |  |  | Median age reported: 61.5 (IQR range 55.8–68.0) years |  |  |  |  |  |  |  |
|  |  |  | 47% |  |  |  |  |  |  |  |
|  |  |  | NR |  |  |  |  |  |  |  |
|  |  |  | Paroxysmal (all participants) |  |  |  |  |  |  |  |
| De Caterina et al, 2018^109^ | Seven European countries (not specified) | Cohort study (registry) | 2,315 | EQ-5D-5L | / | / | / | / | / | / |
|  |  |  | 72±9 years |  |  |  |  |  |  |  |
|  |  |  | Patients on stable treatment with vitamin K antagonist group (n=2,102): 39% & patients switched from vitamin k antagonist to non-vitamin K antagonist oral anticoagulant group (n=213): 35% |  |  |  |  |  |  |  |
|  |  |  | NR |  |  |  |  |  |  |  |
|  |  |  | Paroxysmal (30%), persistent (24%), long-standing persistent (7%) & permanent (39%) |  |  |  |  |  |  |  |
| De Potter et al, 2018^110^ | 24 European countries (not specified), Australia & Canada | Cohort study (registry) | 261 | AFEQT | / | / | / | / | / | / |
|  |  |  | 58.8±11.3 years |  |  |  |  |  |  |  |
|  |  |  | 29.3% |  |  |  |  |  |  |  |
|  |  |  | Caucasian (91.7%) & not specified (8.3%) |  |  |  |  |  |  |  |
|  |  |  | Paroxysmal (all participants) |  |  |  |  |  |  |  |
| De With et al, 2019^111^ | NR | RCT | 230 | SF-36 | / | / | / | AFSS | / | / |
|  |  |  | 65±9 years |  |  |  |  |  |  |  |
|  |  |  | 22% |  |  |  |  |  |  |  |
|  |  |  | NR |  |  |  |  |  |  |  |
|  |  |  | Persistent (all participants) |  |  |  |  |  |  |  |
| Deftereos et al, 2014^112^ | NR | RCT | 206 | WHOQoL-BREF | / | / | / | / | / | / |
|  |  |  | 62.2±5.8 years |  |  |  |  |  |  |  |
|  |  |  | 40% |  |  |  |  |  |  |  |
|  |  |  | NR |  |  |  |  |  |  |  |
|  |  |  | Paroxysmal (all participants) |  |  |  |  |  |  |  |
| Delesie et al, 2021^113^ | Belgium | RCT (protocol) | 1,038 participants to be enrolled | AFEQT & EQ-5D-3L | / | / | / | AFSS & LARQ | / | / |
|  |  |  | NR |  |  |  |  |  |  |  |
|  |  |  | NR |  |  |  |  |  |  |  |
|  |  |  | NR |  |  |  |  |  |  |  |
|  |  |  | NR |  |  |  |  |  |  |  |
| Demolder et al, 2023^114^ | Austria, Belgium, Denmark, Spain & Switzerland | RCT | 200 | SF-36v2 | / | / | / | SCL | / | / |
|  |  |  | 65±9 years |  |  |  |  |  |  |  |
|  |  |  | 40% |  |  |  |  |  |  |  |
|  |  |  | NR |  |  |  |  |  |  |  |
|  |  |  | Persistent (all participants) |  |  |  |  |  |  |  |
| Desteghe et al, 2019^115^ | Belgium | RCT | 67 | SF-12 | / | / | / | LARQ | / | / |
|  |  |  | 72.1±8.6 years |  |  |  |  |  |  |  |
|  |  |  | 37.3% |  |  |  |  |  |  |  |
|  |  |  | NR |  |  |  |  |  |  |  |
|  |  |  | First episode (11.9%), paroxysmal (35.8%), persistent or long-standing persistent (26.9%), permanent (11.9%) & predominant atrial flutter (13.4%) |  |  |  |  |  |  |  |
| Dhruva et al, 2020^116^ | USA | Cohort study | 59 | C-CAP & PROMIS | / | / | / | / | / | / |
|  |  |  | 55.2±13.5 years |  |  |  |  |  |  |  |
|  |  |  | 58.3% |  |  |  |  |  |  |  |
|  |  |  | NR |  |  |  |  |  |  |  |
|  |  |  | NR |  |  |  |  |  |  |  |
| Dhruva et al, 2021^117^ | USA | RCT (protocol) | 150 participants to be enrolled | AFEQT | / | / | / | / | / | / |
|  |  |  | Participants aged >22 years to be enrolled |  |  |  |  |  |  |  |
|  |  |  | NR |  |  |  |  |  |  |  |
|  |  |  | NR |  |  |  |  |  |  |  |
|  |  |  | NR |  |  |  |  |  |  |  |
| Dierkes et al, 2003^118^ | Germany | Cohort study | 33 | SF-36 | / | / | / | / | / |  |
|  |  |  | 56.1±9.9 years |  |  |  |  |  |  |  |
|  |  |  | 22.2% |  |  |  |  |  |  |  |
|  |  |  | NR |  |  |  |  |  |  |  |
|  |  |  | Paroxysmal (all participants) |  |  |  |  |  |  |  |
| Ding et al, 2018^119^ | Sweden | Cohort study (registry) | 243 with AF (total n=2,685) | / | / | / | MMSE | / | / | / |
|  |  |  | 80.9±9.4 years |  |  |  |  |  |  |  |
|  |  |  | 56% |  |  |  |  |  |  |  |
|  |  |  | NR |  |  |  |  |  |  |  |
|  |  |  | NR |  |  |  |  |  |  |  |
| Dohi et al, 2019^120^ | Japan | RCT (protocol) | Participants aged >20 years to be enrolled | SF-36 | / | / | / | / | / | / |
|  |  |  | 512 participants to be enrolled |  |  |  |  |  |  |  |
|  |  |  | NR |  |  |  |  |  |  |  |
|  |  |  | NR |  |  |  |  |  |  |  |
|  |  |  | Persistent AF participants to be enrolled |  |  |  |  |  |  |  |
| Dorian et al, 2013^121^ | Canada & USA | Methodological study | 210 | AFEQT | / | / | / | AFSS | / | / |
|  |  |  | 62.1±11.9 years |  |  |  |  |  |  |  |
|  |  |  | 42.5% |  |  |  |  |  |  |  |
|  |  |  | NR |  |  |  |  |  |  |  |
|  |  |  | Paroxysmal (65.9%), persistent (23.8%), long-standing persistent (5.1%) & permanent (5.1%) |  |  |  |  |  |  |  |
| Dorian et al, 2009^122^ | Canada | Methodological study | 484 | SF-36 | / | / | / | / | / | / |
|  |  |  | 62.2±12.5 years |  |  |  |  |  |  |  |
|  |  |  | 33% |  |  |  |  |  |  |  |
|  |  |  | NR |  |  |  |  |  |  |  |
|  |  |  | Paroxysmal (62%) & persistent or permanent (38%) |  |  |  |  |  |  |  |
| Dorian et al, 2000^123^ | USA, Canada, Germany & UK | Cohort study | 152 | SF-36 | / | / | / | AFSS | / | / |
|  |  |  | 58±12 years |  |  |  |  |  |  |  |
|  |  |  | 27% |  |  |  |  |  |  |  |
|  |  |  | NR |  |  |  |  |  |  |  |
|  |  |  | Paroxysmal (60.5%) & persistent (39.5%) |  |  |  |  |  |  |  |
| Dorian et al, 2002^124^ | Canada | RCT | 264 | SF-36 | DASI | / | / | AFSS & SCL | / | / |
|  |  |  | 65±10 years |  |  |  |  |  |  |  |
|  |  |  | 41% |  |  |  |  |  |  |  |
|  |  |  | NR |  |  |  |  |  |  |  |
|  |  |  | Paroxysmal (42%) & persistent (58%) |  |  |  |  |  |  |  |
| Doshi et al, 2005^125^ | Canada & USA | RCT | 184 | SF-36 | / | / | / | / | / | / |
|  |  |  | Patients randomised to right ventricular pacing group (n=81): 67±10 years & patients randomised to biventricular pacing group (n=103): 70±10 years |  |  |  |  |  |  |  |
|  |  |  | Patients randomised to right ventricular pacing group: 36% & patients randomised to biventricular pacing group: 37% |  |  |  |  |  |  |  |
|  |  |  | NR |  |  |  |  |  |  |  |
|  |  |  | Chronic (all participants) |  |  |  |  |  |  |  |
| Dossett et al, 2021^126^ | USA | Pilot RCT | 18 | AFEQT | / | GAD-7, MOCS-A, PHQ-8, PSS-10, PSWQ | / | AFS/B | / | / |
|  |  |  | 66±5.5 years |  |  |  |  |  |  |  |
|  |  |  | 78% |  |  |  |  |  |  |  |
|  |  |  | White (100%) |  |  |  |  |  |  |  |
|  |  |  | Paroxysmal (all participants) |  |  |  |  |  |  |  |
| Doucet et al, 2008^127^ | France | Cohort study | 209 | / | ADL | / | MMSE | / | / | / |
|  |  |  | 84.7±7 years |  |  |  |  |  |  |  |
|  |  |  | 60.8% |  |  |  |  |  |  |  |
|  |  |  | NR |  |  |  |  |  |  |  |
|  |  |  | Chronic (all participants) |  |  |  |  |  |  |  |
| Driessen et al, 2018^128^ | Netherlands | RCT | 201 | SF-36 [Dutch] | / | / | / | / | / | / |
|  |  |  | 59.6±7.8 years |  |  |  |  |  |  |  |
|  |  |  | 28% |  |  |  |  |  |  |  |
|  |  |  | NR |  |  |  |  |  |  |  |
|  |  |  | Paroxysmal (43%) & persistent (57%) |  |  |  |  |  |  |  |
| Du et al, 2023^129^ | China | Cohort study | 428 | / | / | HADS | / | / | / | / |
|  |  |  | Median age reported: 67 (IQR range 61–73) years |  |  |  |  |  |  |  |
|  |  |  | 40.2% |  |  |  |  |  |  |  |
|  |  |  | NR |  |  |  |  |  |  |  |
|  |  |  | Paroxysmal (60.7%) & persistent (39.3%) |  |  |  |  |  |  |  |
| Du et al, 2017^130^ | China | Cohort study | 264 after matching | AFEQT & SF-36 | / | / | / | / | / | / |
|  |  |  | Age categories reported - paroxysmal AF ablation group (n=75): ≥75 years (n=4), paroxysmal AF drug group (n=75): ≥75 years (n=4), persistent AF ablation group (n=57): ≥75 years (n=20), persistent AF drug group (n=57): ≥75 years (n=20) |  |  |  |  |  |  |  |
|  |  |  | Paroxysmal AF ablation group: 42.67%, paroxysmal AF drug group: 37.33%, persistent AF ablation group: 31.58%, persistent AF drug group: 42.11% |  |  |  |  |  |  |  |
|  |  |  | NR |  |  |  |  |  |  |  |
|  |  |  | Paroxysmal (60.7%) & persistent (39.3%) |  |  |  |  |  |  |  |
| Duan et al, 2023^131^ | China | Cohort study | 274 | SF-36 | / | HAM-A & HAM-D | / | / | / | / |
|  |  |  | Study group (n=136): 57.88±2.17 years & control group (n=138): 58.17±1.92 years |  |  |  |  |  |  |  |
|  |  |  | 44.9% |  |  |  |  |  |  |  |
|  |  |  | NR |  |  |  |  |  |  |  |
|  |  |  | Paroxysmal (30.3%), persistent (26.6%), long-standing persistent (26.6%) & permanent (16.5%) |  |  |  |  |  |  |  |
| Dublin et al, 2011^132^ | USA | Cohort study | 132 with AF (total n=3,045) | / | / | CES-D | / | / | / | / |
|  |  |  | Median age reported: 76.5 (71.6-82.6) years |  |  |  |  |  |  |  |
|  |  |  | 53.8% |  |  |  |  |  |  |  |
|  |  |  | White (95.5%) & not specified (4.5%) |  |  |  |  |  |  |  |
|  |  |  | NR |  |  |  |  |  |  |  |
| Dudink et al, 2017^133^ | Netherlands | RCT (protocol) | 437 participants to be enrolled | AFEQT & SF-36 | / | / | / | / | / | / |
|  |  |  | Participants aged >18 years to be enrolled |  |  |  |  |  |  |  |
|  |  |  | NR |  |  |  |  |  |  |  |
|  |  |  | NR |  |  |  |  |  |  |  |
|  |  |  | New-onset AF participants to be enrolled |  |  |  |  |  |  |  |
| Duff et al, 2003^134^ | Canada | RCT | 33 | SF-6 | / | / | / | / | / | / |
|  |  |  | Rate responsive mode off group (n=17): 67±4 years & rate responsive mode on group (n=16): 63±3 years |  |  |  |  |  |  |  |
|  |  |  | Rate responsive mode off group: 43% & rate responsive mode on group: 29% |  |  |  |  |  |  |  |
|  |  |  | NR |  |  |  |  |  |  |  |
|  |  |  | Rate responsive mode off group: persistent (71%) & rate responsive mode on group (86%) |  |  |  |  |  |  |  |
| Dulai et al, 2023^135^ | UK | RCT (protocol) | 105 already randomised, 140 participants in total to be enrolled | AFEQT & SF-36 | / | / | / | MAFSI | / | / |
|  |  |  | 66.4±8.7 years |  |  |  |  |  |  |  |
|  |  |  | 27.6% |  |  |  |  |  |  |  |
|  |  |  | NR |  |  |  |  |  |  |  |
|  |  |  | Paroxysmal (20%) & persistent (80%) |  |  |  |  |  |  |  |
| Dulai et al, 2024^136^ | UK | RCT | 126 | AFEQT & SF-36 | / | / | / | MAFSI | / | / |
|  |  |  | Ablation group (n=64): 66.1±8.9 years & sham group (n=62): 67.5±8.3 years |  |  |  |  |  |  |  |
|  |  |  | Ablation group: 26.6% & sham: 32.3% |  |  |  |  |  |  |  |
|  |  |  | NR |  |  |  |  |  |  |  |
|  |  |  | Ablation group: paroxysmal (20.3%) & persistent (79.7%)  Sham group: paroxysmal (21%) & persistent (79%) |  |  |  |  |  |  |  |
| Duytschaever et al, 2020^137^ | Belgium | Cohort study | 105 | SF-36 | / | / | / | SCL | / | / |
|  |  |  | 62±8 years |  |  |  |  |  |  |  |
|  |  |  | 38% |  |  |  |  |  |  |  |
|  |  |  | NR |  |  |  |  |  |  |  |
|  |  |  | Paroxysmal (all participants) |  |  |  |  |  |  |  |
| Duytschaever et al, 2018^138^ | Belgium | RCT | 153 | SF-36v2 | / | / | / | SCL | / | / |
|  |  |  | Median age reported: antiarrhythmic drug therapy off group (n=76): 62 (IQR range 54-70) years & antiarrhythmic drug therapy on group (n=77): 63 (IQR range 56-63) years |  |  |  |  |  |  |  |
|  |  |  | Antiarrhythmic drug therapy off group: 28% & antiarrhythmic drug therapy on group: 26% |  |  |  |  |  |  |  |
|  |  |  | NR |  |  |  |  |  |  |  |
|  |  |  | Paroxysmal (all participants) |  |  |  |  |  |  |  |
| Echouffo-Tcheugui et al, 2017^139^ | USA | Cohort study (registry) | 9,749 | AFEQT | / | / | / | / | / | / |
|  |  |  | Median age reported: 75 (IQR range 67-82) years |  |  |  |  |  |  |  |
|  |  |  | 42.6% |  |  |  |  |  |  |  |
|  |  |  | White (89.6%), Black (4.9%), Hispanic (4.1%) & Other (1.4%) |  |  |  |  |  |  |  |
|  |  |  | New onset (4.5%), paroxysmal (50.7%), persistent (16.8%) & permanent (28.1%) |  |  |  |  |  |  |  |
| Edwards et al, 2004^140^ | UK | RCT | 147 with AF (total n=747) | SF-12 | / | STAI | / | / | / | / |
|  |  |  | 65 years |  |  |  |  |  |  |  |
|  |  |  | 59% |  |  |  |  |  |  |  |
|  |  |  | NR |  |  |  |  |  |  |  |
|  |  |  | Non-valvular (all participants) |  |  |  |  |  |  |  |
| Efremidis et al, 2014^141^ | NR | Cohort study | 57 | SF-36 | / | BDI [Greek] & STAI [Greek] | / | / | / | / |
|  |  |  | 56.9±12.2 years |  |  |  |  |  |  |  |
|  |  |  | 40.4% |  |  |  |  |  |  |  |
|  |  |  | NR |  |  |  |  |  |  |  |
|  |  |  | Paroxysmal (all participants) |  |  |  |  |  |  |  |
| Elliott et al, 2021^142^ | Australia | Cohort study | 203 | / | / | / | / | AFSS | / | / |
|  |  |  | 66±11 years |  |  |  |  |  |  |  |
|  |  |  | 40.4% |  |  |  |  |  |  |  |
|  |  |  | NR |  |  |  |  |  |  |  |
|  |  |  | Persistent & paroxysmal (n or % not specified) |  |  |  |  |  |  |  |
| Elliott et al, 2023^143^ | Australia | RCT | 120 | / | / | / | / | AFSS | / | / |
|  |  |  | Exercise group (n=60): 65±10 years & control group (n=60): 65±11 years |  |  |  |  |  |  |  |
|  |  |  | Exercise group: 42% & control group: 43% |  |  |  |  |  |  |  |
|  |  |  | NR |  |  |  |  |  |  |  |
|  |  |  | Exercise group: persistent (37%) & control group: persistent (40%) |  |  |  |  |  |  |  |
| Elliott et al, 2008^144^ | USA | RCT | 118 | SR-36 | DASI | / | / | / | / | / |
|  |  |  | Enoxaparin group (n=55): 66.2±13.3 years & unfractionated heparin group (n=63): 63.9±15.5 years |  |  |  |  |  |  |  |
|  |  |  | Enoxaparin group: 24% & unfractionated heparin group: 29% |  |  |  |  |  |  |  |
|  |  |  | NR |  |  |  |  |  |  |  |
|  |  |  | NR |  |  |  |  |  |  |  |
| Erdogan et al, 2003^145^ | Germany | Cohort study | 30 | SF-36 | / | / | / | / | / | / |
|  |  |  | 54.1±9.5 years |  |  |  |  |  |  |  |
|  |  |  | 30% |  |  |  |  |  |  |  |
|  |  |  | NR |  |  |  |  |  |  |  |
|  |  |  | Paroxysmal (all participants) |  |  |  |  |  |  |  |
| Essebag et al, 2020^146^ | Canada | Cohort study | 230 | AFEQT & SF-12 | / | / | / | / | / | / |
|  |  |  | 57.30±10.82 years |  |  |  |  |  |  |  |
|  |  |  | 29.6% |  |  |  |  |  |  |  |
|  |  |  | NR |  |  |  |  |  |  |  |
|  |  |  | Paroxysmal (all participants) |  |  |  |  |  |  |  |
| Evans et al, 2019^147^ | UK | Cohort study | 370 | C-CAP & EQ-5D-5L | / | / | / | / | / | / |
|  |  |  | 62.04±11.83 years |  |  |  |  |  |  |  |
|  |  |  | 43.6% |  |  |  |  |  |  |  |
|  |  |  | NR |  |  |  |  |  |  |  |
|  |  |  | NR |  |  |  |  |  |  |  |
| Farina et al, 1997^148^ | Italy | Case-control study | 37 | / | / | HAM-D | MMSE | / | / | / |
|  |  |  | Chronic AF group (n=16): 65.3±6.6 years & paroxysmal AF group (n=21): 58.3+9.5 years |  |  |  |  |  |  |  |
|  |  |  | Chronic AF group: 18.1% & paroxysmal AF group: 23.8% |  |  |  |  |  |  |  |
|  |  |  | NR |  |  |  |  |  |  |  |
|  |  |  | Chronic (43.2%) & paroxysmal (56.8%) |  |  |  |  |  |  |  |
| Fareau et al, 2015^149^ | France | Cohort study | 89 | / | ADL & OARS | / | / | / | / | / |
|  |  |  | 79.6±8.1 years |  |  |  |  |  |  |  |
|  |  |  | 60.7% |  |  |  |  |  |  |  |
|  |  |  | NR |  |  |  |  |  |  |  |
|  |  |  | Non-valvular (all participants) |  |  |  |  |  |  |  |
| Farwati et al, 2023^150^ | USA | Cohort study (registry) | 848 | / | / | / | / | AFSS | / | / |
|  |  |  | 63.8±9 years |  |  |  |  |  |  |  |
|  |  |  | 28.3% |  |  |  |  |  |  |  |
|  |  |  | NR |  |  |  |  |  |  |  |
|  |  |  | Paroxysmal (49%) & persistent (51%) |  |  |  |  |  |  |  |
| Farwati et al, 2021^151^ | USA | Cohort study (registry) | 956 | / | / | / | / | AFSS | / | / |
|  |  |  | 63.9±9 years |  |  |  |  |  |  |  |
|  |  |  | 25% |  |  |  |  |  |  |  |
|  |  |  | NR |  |  |  |  |  |  |  |
|  |  |  | Paroxysmal (56.1%) & persistent (43.9%) |  |  |  |  |  |  |  |
| Feinberg et al, 2021^152^ | Denmark | RCT (protocol) | 350 participants to be enrolled | AFEQT & SF-36 | / | / | / | / | / | / |
|  |  |  | Participants aged ≥18 to be enrolled |  |  |  |  |  |  |  |
|  |  |  | NR |  |  |  |  |  |  |  |
|  |  |  | NR |  |  |  |  |  |  |  |
|  |  |  | Persistent or paroxysmal AF participants to be enrolled |  |  |  |  |  |  |  |
| Fengsrud et al, 2016^153^ | Sweden | RCT | 34 | SF-36 | / | / | / | / | / | / |
|  |  |  | Ablation group (n=15): 66.6±5.5 years & control group (n=19): 65.8±5.0 years |  |  |  |  |  |  |  |
|  |  |  | 23.5% |  |  |  |  |  |  |  |
|  |  |  | NR |  |  |  |  |  |  |  |
|  |  |  | Long-standing persistent (all participants) |  |  |  |  |  |  |  |
| Ferguson et al, 2024^154^ | Australia | RCT (protocol) | 100 participants to be enrolled | AFEQT | / | / | / | / | / | / |
|  |  |  | Participants aged ≥18 to be enrolled |  |  |  |  |  |  |  |
|  |  |  | NR |  |  |  |  |  |  |  |
|  |  |  | NR |  |  |  |  |  |  |  |
|  |  |  | NR |  |  |  |  |  |  |  |
| Ferreira et al, 2024^155^ | Portugal | Cohort study | 78 | AFEQT [Portuguese] | / | / | / | / | / | / |
|  |  |  | 59±10 years |  |  |  |  |  |  |  |
|  |  |  | 40% |  |  |  |  |  |  |  |
|  |  |  | NR |  |  |  |  |  |  |  |
|  |  |  | Paroxysmal (85.9%) & persistent (14.1%) |  |  |  |  |  |  |  |
| Feyz et al, 2019^156^ | NR | Pilot cohort study | 20 | AFEQT | / | / | / | / | / | / |
|  |  |  | 64±7 years |  |  |  |  |  |  |  |
|  |  |  | 55% |  |  |  |  |  |  |  |
|  |  |  | NR |  |  |  |  |  |  |  |
|  |  |  | Paroxysmal (90%) & persistent (10%) |  |  |  |  |  |  |  |
| Fiala et al, 2017^157^ | NR | Cohort study (registry) | 202 | EQ-5D | / | / | / | / | / | / |
|  |  |  | Patients with sinus rhythm at month 12 group (n=171): 59±9 years & patients with AF at month 12 group (n=31): 61±8 years |  |  |  |  |  |  |  |
|  |  |  | Patients with sinus rhythm at month 12 group: 20.5% & patients with AF at month 12 group: 32.3% |  |  |  |  |  |  |  |
|  |  |  | NR |  |  |  |  |  |  |  |
|  |  |  | Long-standing persistent (all participants) |  |  |  |  |  |  |  |
| Fiala et al, 2014^158^ | Czech Republic | Cohort study | 160 | EQ-5D | / | / | / | / | / | / |
|  |  |  | 59±9 years |  |  |  |  |  |  |  |
|  |  |  | 23% |  |  |  |  |  |  |  |
|  |  |  | NR |  |  |  |  |  |  |  |
|  |  |  | Long-standing persistent (all patients) |  |  |  |  |  |  |  |
| Fichtner et al, 2012^159^ | Germany | Cohort study | 133 | WHO-5 | / | MDI | / | AFSS | / | / |
|  |  |  | 57±10 years |  |  |  |  |  |  |  |
|  |  |  | 26% |  |  |  |  |  |  |  |
|  |  |  | NR |  |  |  |  |  |  |  |
|  |  |  | Paroxysmal (65.4%) & persistent (34.6%) |  |  |  |  |  |  |  |
| Flaker et al, 2010^160^ | 31 countries in Europe & North and South America (not specified) | RCT | 2,510 | / | / | / | MMSE | / | / | / |
|  |  |  | 70.9±9.5 years |  |  |  |  |  |  |  |
|  |  |  | 34.5% |  |  |  |  |  |  |  |
|  |  |  | White (81.9%), Black (1.2%), Asian (0.9%) & Other (16%) |  |  |  |  |  |  |  |
|  |  |  | NR |  |  |  |  |  |  |  |
| Fleischmann et al, 2009^161^ | USA | RCT | 2,010 | SF-36 | / | / | / | / | Specific activity scale | / |
|  |  |  | Rate modulated right ventricular pacing group (n=996): 73.1±11.0 years & dual chamber pacing group (n=1,014): 72.9±11.1 years |  |  |  |  |  |  |  |
|  |  |  | Rate modulated right ventricular pacing group: 48% & dual chamber pacing group: 47% |  |  |  |  |  |  |  |
|  |  |  | Rate modulated right ventricular pacing group: non-White (14%) & dual chamber pacing group: non-White (16%) |  |  |  |  |  |  |  |
|  |  |  | Rate modulated right ventricular pacing group: paroxysmal (44%) & dual chamber pacing group: paroxysmal (47%) |  |  |  |  |  |  |  |
| Forlani et al, 2006^162^ | Italy | Cohort study | 91 | SF-36 [Italian] | / | / | / | / | / | / |
|  |  |  | Good quality of life group (n=54): 67±6 years & poor quality of life group (n=37): 66±8 years |  |  |  |  |  |  |  |
|  |  |  | 71.4% |  |  |  |  |  |  |  |
|  |  |  | NR |  |  |  |  |  |  |  |
|  |  |  | Permanent (all participants) |  |  |  |  |  |  |  |
| Forleo et al, 2009^163^ | Italy | Pilot RCT | 70 | SF-36 | / | / | / | / | / | / |
|  |  |  | Ablation group (n=35): 63.2±8.6 years & antiarrhythmic drug treatment group (n=35): 64.8±6.5 years |  |  |  |  |  |  |  |
|  |  |  | Ablation group: 42.9% & antiarrhythmic drug treatment group: 34.3% |  |  |  |  |  |  |  |
|  |  |  | NR |  |  |  |  |  |  |  |
|  |  |  | Ablation group: paroxysmal (45.7%) & persistent (54.3%)  Antiarrhythmic drug treatment group: paroxysmal (37.1%) & persistent (62.9%) |  |  |  |  |  |  |  |
| Forleo et al, 2007^164^ | Italy | RCT | 221 | SF-36 | / | / | / | / | / | / |
|  |  |  | 58.2+10.5 years |  |  |  |  |  |  |  |
|  |  |  | 32.1% |  |  |  |  |  |  |  |
|  |  |  | NR |  |  |  |  |  |  |  |
|  |  |  | Paroxysmal (59.7%), persistent (36.7%) & permanent (3.6%) |  |  |  |  |  |  |  |
| Foster-Witassek et al, 2023^165^ | Switzerland | Cohort study | 2,412 | EQ-5D-3L | / | / | / | / | / | / |
|  |  |  | Median age reported: 73.6 (IQR 68.2-79.0) years |  |  |  |  |  |  |  |
|  |  |  | 27.4% |  |  |  |  |  |  |  |
|  |  |  | NR |  |  |  |  |  |  |  |
|  |  |  | Paroxysmal (44.7%), persistent (30.7%) & permanent (24.7%) |  |  |  |  |  |  |  |
| Frasure-Smith et al, 2009^166^ | NR | RCT | 974 | / | / | BDI-II [English French, Danish, Spanish & Portuguese] | / | / | / | / |
|  |  |  | 66+11 years |  |  |  |  |  |  |  |
|  |  |  | 17.7% |  |  |  |  |  |  |  |
|  |  |  | Not depressed group (n=662): non-White (9.7%) & mild-moderate depression group (n=312): non-White (17.9%) |  |  |  |  |  |  |  |
|  |  |  | NR |  |  |  |  |  |  |  |
| Frasure-Smith et al, 2012^167^ | NR | RCT | 933 | / | / | ASI [English French, Danish, Spanish & Portuguese] | / | / | / | / |
|  |  |  | 66+11.2 years |  |  |  |  |  |  |  |
|  |  |  | 17.7% |  |  |  |  |  |  |  |
|  |  |  | Lower anxiety score group (n=700): non-White (10.7%) & high anxiety group (n=233): non-White (18.5%) |  |  |  |  |  |  |  |
|  |  |  | Paroxysmal (74.9%) & not specified (25.1%) |  |  |  |  |  |  |  |
| Frausing et al, 2024^168^ | Netherlands | Cohort study | 417 | EQ-5D-5L | / | / | / | AFSS | / | / |
|  |  |  | Median age reported: 65 (IQR range 58-71) years |  |  |  |  |  |  |  |
|  |  |  | 43% |  |  |  |  |  |  |  |
|  |  |  | NR |  |  |  |  |  |  |  |
|  |  |  | Paroxysmal (all participants) |  |  |  |  |  |  |  |
| Fujisawa et al, 2021^169^ | Japan | Cohort study (registry) | 1,285 | AFEQT | / | / | / | / | / | / |
|  |  |  | Family history of AF group (n=204): 63.3±10.9 years & no family history of AF group (n=1081): 67.3±11.2 years |  |  |  |  |  |  |  |
|  |  |  | Family history of AF group: 27.8% & no family history of AF group: 28.6% |  |  |  |  |  |  |  |
|  |  |  | NR |  |  |  |  |  |  |  |
|  |  |  | Family history of AF group: paroxysmal (55.9%) & no family history of AF group: paroxysmal (51.2%) |  |  |  |  |  |  |  |
| Fumagalli et al, 2015^170^ | NR | Cohort study | 30 | EQ-5D-3L & EQ-VAS | / | HADS & PSS-10 | / | / | / | / |
|  |  |  | 81±9 years |  |  |  |  |  |  |  |
|  |  |  | Warfarin group (n=15): 26.7% & new oral anticoagulant group (n=15): 40% |  |  |  |  |  |  |  |
|  |  |  | NR |  |  |  |  |  |  |  |
|  |  |  | Permanent (13.3%) & not specified (86.7%) |  |  |  |  |  |  |  |
| Fumagalli et al, 2021^171^ | NR | Cohort study | 31 | / | / | GDS | MMSE | / | / | / |
|  |  |  | 79±6 years |  |  |  |  |  |  |  |
|  |  |  | 41.9% |  |  |  |  |  |  |  |
|  |  |  | NR |  |  |  |  |  |  |  |
|  |  |  | Persistent (all participants) |  |  |  |  |  |  |  |
| Fumagalli et al, 2020^172^ | NR | Cohort study | 134 | / | / | GDS | MMSE | / | / | / |
|  |  |  | 77±9 years |  |  |  |  |  |  |  |
|  |  |  | 36.6% |  |  |  |  |  |  |  |
|  |  |  | NR |  |  |  |  |  |  |  |
|  |  |  | Persistent (all participants) |  |  |  |  |  |  |  |
| Fumagalli et al, 2023^173^ | NR | Cohort study | 50 with AF (total n=72) | / | / | GDS | MMSE | / | / | / |
|  |  |  | 76±6 years |  |  |  |  |  |  |  |
|  |  |  | 32% |  |  |  |  |  |  |  |
|  |  |  | NR |  |  |  |  |  |  |  |
|  |  |  | Persistent (all participants) |  |  |  |  |  |  |  |
| Furlanello et al, 2008^174^ | Italy | Cohort study | 20 | SF-36 | / | / | / | / | / | / |
|  |  |  | 44.4±13.0 years |  |  |  |  |  |  |  |
|  |  |  | 0% |  |  |  |  |  |  |  |
|  |  |  | NR |  |  |  |  |  |  |  |
|  |  |  | Idiopathic (all participants) |  |  |  |  |  |  |  |
| Gabilondo et al, 2021^175^ | Spain | Cross-sectional study | 333 | EQ-5D-5L [Spanish] | / | / | SPMSQ | / | / | / |
|  |  |  | Median age reported: 75 (IQR range 68-82) years |  |  |  |  |  |  |  |
|  |  |  | 44.1% |  |  |  |  |  |  |  |
|  |  |  | NR |  |  |  |  |  |  |  |
|  |  |  | Non-valvular (all participants) |  |  |  |  |  |  |  |
| Gagné et al, 2019^176^ | Canada | RCT | 60 | AFEQT [French] | / | / | / | / | / | / |
|  |  |  | 56±13 years |  |  |  |  |  |  |  |
|  |  |  | Education only group (n=30): 40% & education and video group (n=30): 23% |  |  |  |  |  |  |  |
|  |  |  | NR |  |  |  |  |  |  |  |
|  |  |  | NR |  |  |  |  |  |  |  |
| Gaita et al, 2018^177^ | Italy | Cohort study | 255 | SF-36 | / | / | / | / | / | / |
|  |  |  | 57.4±10 years |  |  |  |  |  |  |  |
|  |  |  | 23% |  |  |  |  |  |  |  |
|  |  |  | NR |  |  |  |  |  |  |  |
|  |  |  | Paroxysmal (42.7%) & persistent (57.2%) |  |  |  |  |  |  |  |
| Gallagher et al, 2020^178^ | Australia | RCT | 72 | SF-12 | / | / | / | / | GPAQ | / |
|  |  |  | 65±11 years |  |  |  |  |  |  |  |
|  |  |  | 44% |  |  |  |  |  |  |  |
|  |  |  | NR |  |  |  |  |  |  |  |
|  |  |  | NR |  |  |  |  |  |  |  |
| Galli et al, 2018^179^ | Italy | Case-control study | 58 | / | / | HADS & PSS-10 | / | / | / | / |
|  |  |  | Unknown reasons group (n=39): 75.08±10.64 years & known reasons group (n=19): 74.78±11.1 years |  |  |  |  |  |  |  |
|  |  |  | Unknown reasons group: 51.3% & known reasons group: 57.9% |  |  |  |  |  |  |  |
|  |  |  | NR |  |  |  |  |  |  |  |
|  |  |  | NR |  |  |  |  |  |  |  |
| García-Izquierdo et al, 2020^180^ | Spain | Cohort study | 107 | / | / | STAI [Spanish] | / | / | / | / |
|  |  |  | 65.8±9.6 years |  |  |  |  |  |  |  |
|  |  |  | 26.2% |  |  |  |  |  |  |  |
|  |  |  | NR |  |  |  |  |  |  |  |
|  |  |  | Persistent (all participants) |  |  |  |  |  |  |  |
| Garimella et al, 2015^181^ | USA | Cohort study | 458 | AFEQT | / | HADS, PHQ-9 & WI | / | AFSS | / | / |
|  |  |  | 65.9 years |  |  |  |  |  |  |  |
|  |  |  | 33.2% |  |  |  |  |  |  |  |
|  |  |  | NR |  |  |  |  |  |  |  |
|  |  |  | Persistent (48.5%) |  |  |  |  |  |  |  |
| Garimella et al, 2016^182^ | USA | Cohort study | 332 | / | / | PHQ-9 | / | AFSS | / | / |
|  |  |  | 60.1±15.8 years |  |  |  |  |  |  |  |
|  |  |  | 28.6% |  |  |  |  |  |  |  |
|  |  |  | NR |  |  |  |  |  |  |  |
|  |  |  | NR |  |  |  |  |  |  |  |
| Gatti et al, 2023^183^ | Italy | Cohort study | 36 | EQ-5D [Italian] | / | / | / | / | / | / |
|  |  |  | Median age reported: permanent AF group (n=19): 80 (IQR range 76-84.5) years, persistent AF group (n=4): 67 (IQR range 65.8-71.3) years & paroxysmal group (n=13): 81 (IQR range 68-86) years |  |  |  |  |  |  |  |
|  |  |  | 50% |  |  |  |  |  |  |  |
|  |  |  | NR |  |  |  |  |  |  |  |
|  |  |  | Paroxysmal (36.1%), persistent (11.1%) & permanent (52.8%), |  |  |  |  |  |  |  |
| Gehi et al, 2012^184^ | USA | Cohort study | 300 | AFEQT & SF-12 | / | HADS, PHQ-9 & WI | / | AFSS | / | / |
|  |  |  | 61.7±13.5 years |  |  |  |  |  |  |  |
|  |  |  | 33.9% |  |  |  |  |  |  |  |
|  |  |  | Caucasian (88.3%), Black (9.7%) & Hispanic (0.8%) |  |  |  |  |  |  |  |
|  |  |  | Persistent (58%) & not specified (42%) |  |  |  |  |  |  |  |
| Genbrugge et al, 2021^185^ | Belgium | Cohort study | 60 with AF (total n=80) | SF-36 | / | / | MMSE | / | / | / |
|  |  |  | Median age reported: successful electrical cardioversion group (n=50): 67 (IQR range 60-73) years & unsuccessful electrical cardioversion group (n=10): 75 (IQR range 70-83) years |  |  |  |  |  |  |  |
|  |  |  | Successful electrical cardioversion group: 32% & unsuccessful electrical cardioversion group: 30% |  |  |  |  |  |  |  |
|  |  |  | NR |  |  |  |  |  |  |  |
|  |  |  | Paroxysmal (43.3%) & persistent (56.7%) |  |  |  |  |  |  |  |
| Gerstenfeld et al, 2001^186^ | USA | Cohort study | 71 | SF-36 | / | / | / | AFSS | / | / |
|  |  |  | 51±12 years |  |  |  |  |  |  |  |
|  |  |  | Recur group (n=32): 19% & success group (n=16): 25% |  |  |  |  |  |  |  |
|  |  |  | NR |  |  |  |  |  |  |  |
|  |  |  | Paroxysmal (87%) & persistent (13%) |  |  |  |  |  |  |  |
| Gilbert et al, 2015^187^ | NR | Cohort study | 29 | EQ-5D | / | / | / | / | / | / |
|  |  |  | Successful electrical cardioversion group:50±11 years & unsuccessful electrical cardioversion group: 65±7 years |  |  |  |  |  |  |  |
|  |  |  | 10.3% |  |  |  |  |  |  |  |
|  |  |  | NR |  |  |  |  |  |  |  |
|  |  |  | Persistent (all participants) |  |  |  |  |  |  |  |
| Gill et al, 2024^188^ | UK & USA | RCT | 153 | SF-36 | / | / | / | AFSS | / | / |
|  |  |  | Hybrid AF convergent group (n=102): 63.7±9.6 years & catheter ablation group (n=51): 65.1±6.7 years |  |  |  |  |  |  |  |
|  |  |  | Hybrid AF convergent group: 22% & catheter ablation group: 47% |  |  |  |  |  |  |  |
|  |  |  | NR |  |  |  |  |  |  |  |
|  |  |  | Persistent (58%) & long-standing persistent (42%) |  |  |  |  |  |  |  |
| Gisi et al, 2020^189^ | USA | Cohort study | 260 | AFEQT | / | PHQ-9 | / | / | / | / |
|  |  |  | 71.7±10.1 years |  |  |  |  |  |  |  |
|  |  |  | 44.6% |  |  |  |  |  |  |  |
|  |  |  | White (95%) & not specified (5%) |  |  |  |  |  |  |  |
|  |  |  | Non-valvular (all participants) |  |  |  |  |  |  |  |
| Gleason et al, 2019^190^ | USA | Cohort study | 953 | AFEQT & PROMIS-29 | / | / | / | / | / | / |
|  |  |  | 72±10 years |  |  |  |  |  |  |  |
|  |  |  | 35% |  |  |  |  |  |  |  |
|  |  |  | White (93%), Black (5%) & Other (1%) |  |  |  |  |  |  |  |
|  |  |  | NR |  |  |  |  |  |  |  |
| Goebel et al, 2021^191^ | Germany | Cohort study | 67 | SF-36 | / | / | / | / | / | / |
|  |  |  | 75.3±6.3 years |  |  |  |  |  |  |  |
|  |  |  | 43.3% |  |  |  |  |  |  |  |
|  |  |  | NR |  |  |  |  |  |  |  |
|  |  |  | Paroxysmal (16.4%) & non-paroxysmal (83.6%) |  |  |  |  |  |  |  |
| Goette et al, 2015^192^ | Germany | Cohort study | 342 | AF-QoL, EQ-5D, SF-12 | / | / | / | / | / | / |
|  |  |  | 66.3±9.7 years |  |  |  |  |  |  |  |
|  |  |  | 42.7% |  |  |  |  |  |  |  |
|  |  |  | NR |  |  |  |  |  |  |  |
|  |  |  | Paroxysmal (71.4%), persistent (26.6%) & not specified (2%) |  |  |  |  |  |  |  |
| Goette et al, 2022^193^ | NR | RCT | 2,785 | EQ-5D & SF-12 | / | / | MoCA | / | / | / |
|  |  |  | 70.0±8.3 years |  |  |  |  |  |  |  |
|  |  |  | 46% |  |  |  |  |  |  |  |
|  |  |  | NR |  |  |  |  |  |  |  |
|  |  |  | First episode (37.6%), paroxysmal (35.7%) & persistent (26.7%) |  |  |  |  |  |  |  |
| Goette et al, 2012^194^ | Germany | RCT | 425 | SF-12 | / | / | / | / | / | / |
|  |  |  | Placebo group (n=211): 61.9±10.2 years & Olmesartan group (n=214): 61.1±11.2 years |  |  |  |  |  |  |  |
|  |  |  | Placebo group: 45% & Olmesartan group: 37.9% |  |  |  |  |  |  |  |
|  |  |  | NR |  |  |  |  |  |  |  |
|  |  |  | Paroxysmal (all participants) |  |  |  |  |  |  |  |
| Goldberg et al, 2003^195^ | USA | Cohort study | 33 | SF-36 | / | / | / | / | / | / |
|  |  |  | 51±18 years |  |  |  |  |  |  |  |
|  |  |  | 30% |  |  |  |  |  |  |  |
|  |  |  | NR |  |  |  |  |  |  |  |
|  |  |  | Paroxysmal (all participants) |  |  |  |  |  |  |  |
| Goli et al, 2012^196^ | USA | Cohort study | 300 | / | / | HADS & PHQ-9 | / | AFSS | / | / |
|  |  |  | 61.7±13.5 years |  |  |  |  |  |  |  |
|  |  |  | 33.9% |  |  |  |  |  |  |  |
|  |  |  | Caucasian (88.1%) & Black or Hispanic (11.9%) |  |  |  |  |  |  |  |
|  |  |  | Persistent (58%) & paroxysmal (42%) |  |  |  |  |  |  |  |
| Golwala et al, 2016^197^ | USA | Cohort study (registry) | 9,524 | AFEQT | / | / | / | / | / | / |
|  |  |  | 74±11 years |  |  |  |  |  |  |  |
|  |  |  | 42.5% |  |  |  |  |  |  |  |
|  |  |  | White (91%), Black (5%) & Hispanic (4%) |  |  |  |  |  |  |  |
|  |  |  | First detected (4.5%), paroxysmal (50.6%) & persistent or permanent (44.9%) |  |  |  |  |  |  |  |
| Goren et al, 2013^198^ | USA | Survey study | 1,296 | SF-12v2 | / | Whooley depression screener | / | / | / | WPAI |
|  |  |  | 64.86±12.17 years |  |  |  |  |  |  |  |
|  |  |  | 34.9% |  |  |  |  |  |  |  |
|  |  |  | White (92.75%), Black (2.08%), Hispanic (2.39%) & Other (2.78%) |  |  |  |  |  |  |  |
|  |  |  | NR |  |  |  |  |  |  |  |
| Gorzelak-Pabiś et al, 2016^199^ | Poland | Cohort study | 154 | / | / | / | MMSE [Polish] | / | / | / |
|  |  |  | 76±10 years |  |  |  |  |  |  |  |
|  |  |  | 55.8% |  |  |  |  |  |  |  |
|  |  |  | NR |  |  |  |  |  |  |  |
|  |  |  | MMSE ≥27 group (n=62): paroxysmal (32%), persistent (22%) & permanent (8%) MMSE <27 group (n=42): paroxysmal (8%), persistent (24%) & permanent (10%) |  |  |  |  |  |  |  |
| Groenveld et al, 2009^200^ | Netherlands | RCT | 214 | SF-36 [Dutch] | / | / | / | / | / | / |
|  |  |  | Rate control heart rate <80 beats per minute group (n=75): 70±8 years & rate control heart rate ≥80 beats per minute group (n=139): 68±10 years |  |  |  |  |  |  |  |
|  |  |  | NR |  |  |  |  |  |  |  |
|  |  |  | NR |  |  |  |  |  |  |  |
|  |  |  | Permanent (all participants) |  |  |  |  |  |  |  |
| Groenveld et al, 2011^201^ | Netherlands | RCT | 437 | SF-36 [Dutch] | / | / | / | AFSS | / | / |
|  |  |  | 68±8 years |  |  |  |  |  |  |  |
|  |  |  | 33.6% |  |  |  |  |  |  |  |
|  |  |  | NR |  |  |  |  |  |  |  |
|  |  |  | Permanent (all participants) |  |  |  |  |  |  |  |
| Grönefeld et al, 2003^202^ | Germany | RCT | 252 | SF-36 | / | / | / | / | / | / |
|  |  |  | Rate control group (n=125): 61±9 years & rhythm control group (n=127): 60±10 years |  |  |  |  |  |  |  |
|  |  |  | Rate control group: 28% & rhythm control group: 28% |  |  |  |  |  |  |  |
|  |  |  | NR |  |  |  |  |  |  |  |
|  |  |  | Persistent (all participants) |  |  |  |  |  |  |  |
| Gu et al, 2013^203^ | NR | Cohort study | 550 | SF-36 | / | / | / | / | / | / |
|  |  |  | Patients with chronic obstructive pulmonary disease (n=54): 67.5±7.4 years & patients without chronic obstructive pulmonary disease (n=496): 63.8±8.8 years |  |  |  |  |  |  |  |
|  |  |  | Patients with chronic obstructive pulmonary disease: 27.8% & patients without chronic obstructive pulmonary disease: 42.1% |  |  |  |  |  |  |  |
|  |  |  | NR |  |  |  |  |  |  |  |
|  |  |  | Patients with chronic obstructive pulmonary disease: paroxysmal (37%), persistent (29.6%) & long-standing persistent (33.3%)  Patients without chronic obstructive pulmonary disease: paroxysmal (34.5%), persistent (34.9%) & long-standing persistent (30.6%) |  |  |  |  |  |  |  |
| Guedon-Moreau et al, 2010^204^ | Belgium, France & Italy | Cohort study | 229 | SF-36 [French, Dutch & Italian] | / | / | / | AFSS [French, Dutch & Italian] | / | / |
|  |  |  | 64.6±12.0 years |  |  |  |  |  |  |  |
|  |  |  | 38% |  |  |  |  |  |  |  |
|  |  |  | NR |  |  |  |  |  |  |  |
|  |  |  | Paroxysmal (all participants) |  |  |  |  |  |  |  |
| Guhl et al, 2019^205^ | USA | Cohort study | 295 | AFEQT & SF-12 | / | / | / | / | / | / |
|  |  |  | 71.3±9.9 years |  |  |  |  |  |  |  |
|  |  |  | 40% |  |  |  |  |  |  |  |
|  |  |  | White (95.3%) & not specified (4.7%) |  |  |  |  |  |  |  |
|  |  |  | Non-valvular (all participants) |  |  |  |  |  |  |  |
| Guhl et al, 2017^206^ | USA | Pilot RCT (protocol) | 180 participants to be enrolled. 31 participants enrolled in the preliminary study | AFEQT & PROMIS-29 | / | PHQ-9 | / | / | / | / |
|  |  |  | 68±11 years in preliminary study |  |  |  |  |  |  |  |
|  |  |  | 39% in preliminary study |  |  |  |  |  |  |  |
|  |  |  | NR |  |  |  |  |  |  |  |
|  |  |  | Chronic AF (all participants) |  |  |  |  |  |  |  |
| Gullón et al, 2019^207^ | Spain | Cohort study (registry) | 615 | / | BADL | / | SPMSQ | / | / | / |
|  |  |  | 85.23±5.16 years |  |  |  |  |  |  |  |
|  |  |  | 54.3% |  |  |  |  |  |  |  |
|  |  |  | NR |  |  |  |  |  |  |  |
|  |  |  | Paroxysmal (16.7%), persistent (6%) & permanent (77.4%) |  |  |  |  |  |  |  |
| Güneş et al, 2021^208^ | Turkey | Methodological study | 204 | AFEQT [Turkish] | / | / | / | AFSS [Turkish] | / | / |
|  |  |  | 71.33±10.34 years |  |  |  |  |  |  |  |
|  |  |  | 65.2% |  |  |  |  |  |  |  |
|  |  |  | NR |  |  |  |  |  |  |  |
|  |  |  | Paroxysmal (11.8%) & permanent (88.2%) |  |  |  |  |  |  |  |
| Guo et al, 2017^209^ | China | RCT | 209 | EQ-5D | / | / | / | / | / | / |
|  |  |  | App group (n=113): 67.4±10.6 years & usual care group (n=96): 70.9±17.4 years |  |  |  |  |  |  |  |
|  |  |  | App group: 42.5% & usual care group: 47.5% |  |  |  |  |  |  |  |
|  |  |  | NR |  |  |  |  |  |  |  |
|  |  |  | Non-valvular (all participants) |  |  |  |  |  |  |  |
| Gupta et al, 2007^210^ | UK | RCT | 57 | SF-36 | / | / | / | / | / | / |
|  |  |  | Cardioversion group (n=30): 60.2±10 years & ablation group (n=27): 54.7±9.5 years |  |  |  |  |  |  |  |
|  |  |  | 15.8% |  |  |  |  |  |  |  |
|  |  |  | NR |  |  |  |  |  |  |  |
|  |  |  | Lone AF (43.9%) & not specified (56.1%) |  |  |  |  |  |  |  |
| Gupta et al, 2021^211^ | 9 European countries (not specified) | Cohort study | 329 | AFEQT & EQ-5D-5L | / | / | / | / | / | / |
|  |  |  | 61.4±10.0 years |  |  |  |  |  |  |  |
|  |  |  | 39.2% |  |  |  |  |  |  |  |
|  |  |  | NR |  |  |  |  |  |  |  |
|  |  |  | Paroxysmal (all participants) |  |  |  |  |  |  |  |
| Ha et al, 2014^212^ | 21 countries in Europe, North and South America & Asia inclusive of Colombia, France, Germany, Mexico, Spain, Sweden and USA | Cohort study (registry) | 2,439 | / | / | / | / | AFSS | / | / |
|  |  |  | 67.6±11.8 years |  |  |  |  |  |  |  |
|  |  |  | 41.5% |  |  |  |  |  |  |  |
|  |  |  | NR |  |  |  |  |  |  |  |
|  |  |  | Paroxysmal (51.5%) & persistent (42.8%) |  |  |  |  |  |  |  |
| Ha et al, 2020^213^ | Canada | RCT | 148 | AFEQT [English] | / | / | / | AFSS [English & French] | / | / |
|  |  |  | 65.6±9.5 years |  |  |  |  |  |  |  |
|  |  |  | 29.1% |  |  |  |  |  |  |  |
|  |  |  | NR |  |  |  |  |  |  |  |
|  |  |  | Persistent (all participants) |  |  |  |  |  |  |  |
| Haeusler et al, 2022^214^ | 8 European countries (not specified) & USA | RCT | 626 | SF-12 | / | / | MoCA | / | / | / |
|  |  |  | Median age reported: no brain MRI group (n=293): 64 (IRQ range 57-69) years & brain MRI group (n=33): 64 (58-69) years |  |  |  |  |  |  |  |
|  |  |  | No brain MRI group: 34% & brain MRI group: 33% |  |  |  |  |  |  |  |
|  |  |  | NR |  |  |  |  |  |  |  |
|  |  |  | No brain MRI group: paroxysmal (54%), persistent (45%) & long-standing persistent (1%)  Brain MRI group: paroxysmal (62%), persistent (36%) & long-standing persistent (2%) |  |  |  |  |  |  |  |
| Hagens et al, 2005^215^ | Netherlands | RCT | 261 | SF-36 | / | / | / | / | / | / |
|  |  |  | Rate control group (n=130): 69±9 years& rhythm control (n=131): 69±8 years |  |  |  |  |  |  |  |
|  |  |  | Rate control group: 35% & rhythm control group: 35% |  |  |  |  |  |  |  |
|  |  |  | NR |  |  |  |  |  |  |  |
|  |  |  | Persistent (all participants) |  |  |  |  |  |  |  |
| Hagens et al, 2004^216^ | Netherlands | RCT | 352 | SF-36 [Dutch] | / | / | / | / | / | / |
|  |  |  | Rate control group (n=175): 68±9 years & rhythm control group (n=177): 68±8 years |  |  |  |  |  |  |  |
|  |  |  | Rate control group: 39% & rhythm control group: 33% |  |  |  |  |  |  |  |
|  |  |  | NR |  |  |  |  |  |  |  |
|  |  |  | Persistent (all participants) |  |  |  |  |  |  |  |
| Haldar et al, 2020^217^ | UK | RCT | 120 | AFEQT & EQ-5D-5L | / | / | / | / | / | / |
|  |  |  | 62.3±9.6 years |  |  |  |  |  |  |  |
|  |  |  | 25.8% |  |  |  |  |  |  |  |
|  |  |  | White (93%), Asian (1%), Black (3%), Middle Eastern (1%) & not stated (2%) |  |  |  |  |  |  |  |
|  |  |  | Long-standing persistent (all participants) |  |  |  |  |  |  |  |
| Hamer et al, 1994^218^ | USA | Cohort study | 26 with AF (total n=69) | / | / | STAI & SCL-90 | / | / | / | / |
|  |  |  | 60±14 years |  |  |  |  |  |  |  |
|  |  |  | 38.5% |  |  |  |  |  |  |  |
|  |  |  | NR |  |  |  |  |  |  |  |
|  |  |  | Paroxysmal (all participants) |  |  |  |  |  |  |  |
| Hämmerle et al, 2022^219^ | Switzerland | Cohort study | 710 with AF (total n=1,685) | / | / | / | MoCA | / | / | / |
|  |  |  | 75±8 years |  |  |  |  |  |  |  |
|  |  |  | 26% |  |  |  |  |  |  |  |
|  |  |  | NR |  |  |  |  |  |  |  |
|  |  |  | Paroxysmal (15%), persistent (30%) & permanent (55%) |  |  |  |  |  |  |  |
| Haman et al, 2012^220^ | Czech Republic | Cohort study | 303 | EQ-5D [Czech] | / | / | / | / | / | / |
|  |  |  | 57 years |  |  |  |  |  |  |  |
|  |  |  | 43.2% |  |  |  |  |  |  |  |
|  |  |  | NR |  |  |  |  |  |  |  |
|  |  |  | Paroxysmal (51.8%), persistent (31%) & long-standing persistent (17.2%) |  |  |  |  |  |  |  |
| Han et al, 2023^221^ | China | Cohort study | 316 | SF-36 | / | / | / | / | / | / |
|  |  |  | Control group (n=156): 59.03±9.48 years & observational group (n=160): 59.38±8.99 years |  |  |  |  |  |  |  |
|  |  |  | Control group: 40.38% & observational group: 41.25% |  |  |  |  |  |  |  |
|  |  |  | NR |  |  |  |  |  |  |  |
|  |  |  | Control group: primary (28.85%), paroxysmal (47.44%) & persistent (23.72%) Observational group: primary (31.88%), paroxysmal (41.25%) & persistent (26.88%) |  |  |  |  |  |  |  |
| Hanon et al, 2016^222^ | France | Cohort study | 405 | SF-36 | / | / | / | / | / | / |
|  |  |  | 74.8±9.0 years |  |  |  |  |  |  |  |
|  |  |  | 37% |  |  |  |  |  |  |  |
|  |  |  | NR |  |  |  |  |  |  |  |
|  |  |  | Paroxysmal (40%), persistent (11.6%) & permanent (43.2%) |  |  |  |  |  |  |  |
| Hanon et al, 2017^223^ | Austria, France, Germany, Italy, Spain, Switzerland & UK | Cohort study (registry) | 7,225 | EQ-5D | / | / | / | / | / | / |
|  |  |  | 71.5±7.9 years |  |  |  |  |  |  |  |
|  |  |  | 40% |  |  |  |  |  |  |  |
|  |  |  | NR |  |  |  |  |  |  |  |
|  |  |  | Paroxysmal (30%), persistent (24.1%), long-standing persistent (7.2%) & permanent (38.7%) |  |  |  |  |  |  |  |
| Harden et al, 2010^224^ | Sweden | Methodological study | 111 | SF-36 | / | / | / | AF6 & AFSS | / | / |
|  |  |  | 67±12 years |  |  |  |  |  |  |  |
|  |  |  | 20% |  |  |  |  |  |  |  |
|  |  |  | NR |  |  |  |  |  |  |  |
|  |  |  | Persistent (all participants) |  |  |  |  |  |  |  |
| Harden et al, 2009^225^ | Sweden | Methodological study | 111 | SF-36 | / | / | / | AF6 & SCL | / | / |
|  |  |  | 67±12 years |  |  |  |  |  |  |  |
|  |  |  | 20% |  |  |  |  |  |  |  |
|  |  |  | NR |  |  |  |  |  |  |  |
|  |  |  | Persistent (all participants) |  |  |  |  |  |  |  |
| Hasebe et al, 2020^226^ | Japan | Cohort study | 35 | / | / | STAI & SDS | / | / | / | / |
|  |  |  | 60.5±11.9 years |  |  |  |  |  |  |  |
|  |  |  | 31% |  |  |  |  |  |  |  |
|  |  |  | NR |  |  |  |  |  |  |  |
|  |  |  | Paroxysmal (all participants) |  |  |  |  |  |  |  |
| Hashiba et al, 2025^227^ | Denmark | Methodological study | 6 | / | / | / | / | AFSS [Danish] | / | / |
|  |  |  | Median age reported: 75.5 (IQR range 72.8-78.3) years |  |  |  |  |  |  |  |
|  |  |  | 50% |  |  |  |  |  |  |  |
|  |  |  | NR |  |  |  |  |  |  |  |
|  |  |  | Paroxysmal (66.7%), persistent (16.7%) & unclassified (16.7%) |  |  |  |  |  |  |  |
| Hashimoto et al, 2023^228^ | Japan | Cohort study | 84 | / | / | / | MMSE | / | / | / |
|  |  |  | 63.1±9.1 years |  |  |  |  |  |  |  |
|  |  |  | 26% |  |  |  |  |  |  |  |
|  |  |  | NR |  |  |  |  |  |  |  |
|  |  |  | Paroxysmal (59.5%) & non-paroxysmal (40.5%) |  |  |  |  |  |  |  |
| He et al, 2023^229^ | China | Pilot RCT | 80 | / | / | STAI | / | / | / | / |
|  |  |  | Intervention group (n=40): 58.1±10.4 years & control group (n=40): 60.0±11.2 years |  |  |  |  |  |  |  |
|  |  |  | Intervention group: 38% & control group: 40% |  |  |  |  |  |  |  |
|  |  |  | NR |  |  |  |  |  |  |  |
|  |  |  | Intervention group: paroxysmal (60%) & persistent (40%)  Control group: paroxysmal (50%) & persistent (50%) |  |  |  |  |  |  |  |
| Hedberg et al, 2018^230^ | Sweden | Cross-sectional study | 196 | SF-36 | / | HADS | / | / | / | / |
|  |  |  | 66.1±10.5 years |  |  |  |  |  |  |  |
|  |  |  | 34% |  |  |  |  |  |  |  |
|  |  |  | NR |  |  |  |  |  |  |  |
|  |  |  | First episode (28%), paroxysmal (32%), persistent (34%) & permanent (7%) |  |  |  |  |  |  |  |
| Hemels et al, 2006^231^ | Netherlands | Cohort study | 29 | SF-36 | / | / | / | / | / | / |
|  |  |  | 48±6 years |  |  |  |  |  |  |  |
|  |  |  | 6.9% |  |  |  |  |  |  |  |
|  |  |  | NR |  |  |  |  |  |  |  |
|  |  |  | Paroxysmal (62%) & persistent (38%) |  |  |  |  |  |  |  |
| Hemels et al, 2008^232^ | Netherlands | Pilot RCT | 38 | SF-36 [Dutch] | / | / | / | SCL | / | / |
|  |  |  | 62±9 years |  |  |  |  |  |  |  |
|  |  |  | 47% |  |  |  |  |  |  |  |
|  |  |  | NR |  |  |  |  |  |  |  |
|  |  |  | Paroxysmal (all participants) |  |  |  |  |  |  |  |
| Hegbom et al, 2006^233^ | NR | RCT | 28 | SF-36 | / | / | / | / | / | / |
|  |  |  | 64±7 years |  |  |  |  |  |  |  |
|  |  |  | 7.1% |  |  |  |  |  |  |  |
|  |  |  | NR |  |  |  |  |  |  |  |
|  |  |  | Chronic (all participants) |  |  |  |  |  |  |  |
| Hendriks et al, 2010^234^ | Netherlands | RCT (protocol) | 349 participants to be enrolled | SF-36 | / | HADS | / | / | / | / |
|  |  |  | Participants aged >18 years to be enrolled |  |  |  |  |  |  |  |
|  |  |  | NR |  |  |  |  |  |  |  |
|  |  |  | NR |  |  |  |  |  |  |  |
|  |  |  | Newly diagnosed AF participants to be enrolled |  |  |  |  |  |  |  |
| Hendriks et al, 2013^235^ | Netherlands | RCT | 645 (with QoL data available) | SF-36 | / | / | / | / | / | / |
|  |  |  | 67±12 years |  |  |  |  |  |  |  |
|  |  |  | 41.7% |  |  |  |  |  |  |  |
|  |  |  | NR |  |  |  |  |  |  |  |
|  |  |  | Paroxysmal (55%), persistent (16.1%) & permanent (22.3%) |  |  |  |  |  |  |  |
| Hendriks et al, 2014^236^ | Netherlands | RCT | 534 | SF-36 | / | HADS | / | / | / | / |
|  |  |  | 66.6±12.0 years |  |  |  |  |  |  |  |
|  |  |  | 41% |  |  |  |  |  |  |  |
|  |  |  | Caucasian (99.4%) |  |  |  |  |  |  |  |
|  |  |  | Paroxysmal (56.2%), persistent (16.7%) & permanent (21.2%) |  |  |  |  |  |  |  |
| Henry et al, 2013^237^ | USA | Cohort study | 540 | SF-12 | / | / | / | SCL | / | / |
|  |  |  | Women group (n=181): 65.0+12.3 years & men group (n=359): 62.0±11.3 years |  |  |  |  |  |  |  |
|  |  |  | 34% |  |  |  |  |  |  |  |
|  |  |  | NR |  |  |  |  |  |  |  |
|  |  |  | Women group: long-standing persistent (88%) & paroxysmal or other (12%)  Men group: long-standing persistent (93%) & paroxysmal or other (7%) |  |  |  |  |  |  |  |
| Herber et al, 2023^238^ | Switzerland | Cross-sectional study | 1,490 | / | / | / | MoCA | / | IPAQ | / |
|  |  |  | 72.3±8.5 years |  |  |  |  |  |  |  |
|  |  |  | 26.3% |  |  |  |  |  |  |  |
|  |  |  | NR |  |  |  |  |  |  |  |
|  |  |  | Paroxysmal (46.3%), persistent (30.9%) & permanent (22.8%) |  |  |  |  |  |  |  |
| Hickey et al, 2016^239^ | USA | RCT (protocol) | 300 participants to be enrolled | AFEQT, EQ-5D & SF-36 | / | PHQ-9 & STAI | / | / | / | / |
|  |  |  | Participants aged >18 years to be enrolled |  |  |  |  |  |  |  |
|  |  |  | NR |  |  |  |  |  |  |  |
|  |  |  | NR |  |  |  |  |  |  |  |
|  |  |  | Paroxysmal or persistent AF participants to be enrolled |  |  |  |  |  |  |  |
| Ho et al, 2012^240^ | China | Cohort study | 244 | EQ-5D-5L | / | / | / | / | / | / |
|  |  |  | 70.1 years |  |  |  |  |  |  |  |
|  |  |  | 45.9% |  |  |  |  |  |  |  |
|  |  |  | NR |  |  |  |  |  |  |  |
|  |  |  | Newly diagnosed (all participants) |  |  |  |  |  |  |  |
| Hoegh et al, 2016^241^ | Denmark | Cross-sectional study | 873 with AF (total n=42,598) | SF-36v2 [Danish] | / | / | / | / | / | / |
|  |  |  | Median age reported: 64.0 (IQR range 57.1-69.7) years |  |  |  |  |  |  |  |
|  |  |  | 31.5% |  |  |  |  |  |  |  |
|  |  |  | NR |  |  |  |  |  |  |  |
|  |  |  | NR |  |  |  |  |  |  |  |
| Hoglund et al, 2013^242^ | Sweden | Cohort study | 105 | SF-36 | / | / | / | / | / | / |
|  |  |  | 58±9 years |  |  |  |  |  |  |  |
|  |  |  | 26% |  |  |  |  |  |  |  |
|  |  |  | NR |  |  |  |  |  |  |  |
|  |  |  | Paroxysmal (50%), persistent (48%) & long-standing persistent (2%) |  |  |  |  |  |  |  |
| Holmes et al, 2019^243^ | USA | Methodological study | 1,347 | AFEQT | / | / | / | / | / | / |
|  |  |  | 74.3±9.8 years |  |  |  |  |  |  |  |
|  |  |  | 43% |  |  |  |  |  |  |  |
|  |  |  | White (90.7%), Black (5.1%), Hispanic (2.8%) & Other (1.4%) |  |  |  |  |  |  |  |
|  |  |  | First detected (8.2%), paroxysmal (48.8%), persistent (13.4%) & permanent (29.6%) |  |  |  |  |  |  |  |
| Holmlund et al, 2024^244^ | Sweden | Cross-sectional study | 167 | ASTA HRQoL scale, EQ-5D-3L & EQ-VAS | / | / | / | ASTA symptom scale | / | / |
|  |  |  | 68.7±10.4 years |  |  |  |  |  |  |  |
|  |  |  | 31.1% |  |  |  |  |  |  |  |
|  |  |  | NR |  |  |  |  |  |  |  |
|  |  |  | Paroxysmal (34.8%) & persistent (65.2%) |  |  |  |  |  |  |  |
| Holmlund et al, 2024 [a] ^245^ | Sweden | Survey study | 129 | ASTA HRQoL scale | / | HADS | / | / | / | / |
|  |  |  | 70.8±8.5 years |  |  |  |  |  |  |  |
|  |  |  | 33.3% |  |  |  |  |  |  |  |
|  |  |  | NR |  |  |  |  |  |  |  |
|  |  |  | Paroxysmal (36.2%) & persistent (63.8%) |  |  |  |  |  |  |  |
| Howes et al, 2001^246^ | USA | Case-control study | 52 with AF (total n =100) | SF-36 | / | / | / | / | YPAS | / |
|  |  |  | 77±7.2 years |  |  |  |  |  |  |  |
|  |  |  | 1% |  |  |  |  |  |  |  |
|  |  |  | NR |  |  |  |  |  |  |  |
|  |  |  | Persistent (all participants) |  |  |  |  |  |  |  |
| Hsieh et al, 2021^247^ | Taiwan | RCT | 231 | EQ-5D-3L [Chinese] | / | / | / | / | / | / |
|  |  |  | 73.08±11.71 years |  |  |  |  |  |  |  |
|  |  |  | 49.8% |  |  |  |  |  |  |  |
|  |  |  | NR |  |  |  |  |  |  |  |
|  |  |  | Paroxysmal (74%), persistent (9.1%) & permanent (16.9%) |  |  |  |  |  |  |  |
| Hsu et al, 2016^248^ | Taiwan | Cohort study | 100 with AF (total n=1,732) | SF-12v2 & SF-36 [Chinese] | / | HADS [Chinese] | / | / | / | / |
|  |  |  | 78.7±7.7 years |  |  |  |  |  |  |  |
|  |  |  | 35% |  |  |  |  |  |  |  |
|  |  |  | NR |  |  |  |  |  |  |  |
|  |  |  | Paroxysmal (29%) & not specified (71%) |  |  |  |  |  |  |  |
| Hsu et al, 2004^249^ | NR | Cohort study | 116 | SF-36 | / | / | / | SCL | / | / |
|  |  |  | 56±10 years |  |  |  |  |  |  |  |
|  |  |  | 12% |  |  |  |  |  |  |  |
|  |  |  | NR |  |  |  |  |  |  |  |
|  |  |  | Permanent (74%), persistent (17%) & paroxysmal (9%) |  |  |  |  |  |  |  |
| Humphries et al, 2021^250^ | Canada | RCT | 717 | EQ-5D-5L | / | / | / | / | / | / |
|  |  |  | Clinical decision support group (n=374): 72.5±9.4 years & control group (n=343): 72.1 ±9.4 years |  |  |  |  |  |  |  |
|  |  |  | Clinical decision support group: 39.8% & control group: 34.7% |  |  |  |  |  |  |  |
|  |  |  | Clinical decision support group: White (88%) & Other or not documented (12%)  Control group: White (89.8%) & Other or not documented (10.2%) |  |  |  |  |  |  |  |
|  |  |  | NR |  |  |  |  |  |  |  |
| Hu et al, 2006^251^ | China | RCT | 183 | SF-36 [Chinese] | / | / | / | / | / | / |
|  |  |  | 36.9±5.4 years |  |  |  |  |  |  |  |
|  |  |  | Rate control group (n=91): 64.8% & rhythm control group (n=92): 68.5% |  |  |  |  |  |  |  |
|  |  |  | NR |  |  |  |  |  |  |  |
|  |  |  | Chronic (all participants) |  |  |  |  |  |  |  |
| Huang H et al, 2024^252^ | China | RCT | 882 | SF-36 | / | / | / | / | / | / |
|  |  |  | Median age reported: Shensong Yangxin group (n=443): 62 (IQR range 54-68) years & placebo group (n=439): 63 (IQR range 55-68) years |  |  |  |  |  |  |  |
|  |  |  | Shensong Yangxin group: 22.8% & placebo group (n=439): 30.7% |  |  |  |  |  |  |  |
|  |  |  | NR |  |  |  |  |  |  |  |
|  |  |  | Persistent (all participants) |  |  |  |  |  |  |  |
| Huang T et al, 2024^253^ | China | Cohort study | 190 | SF-36 | / | / | / | / | / | / |
|  |  |  | High density mapping group (n=95): 60.36±9.44 years & routine group (n=105): 62.60±9.34 years |  |  |  |  |  |  |  |
|  |  |  | 33.7% |  |  |  |  |  |  |  |
|  |  |  | NR |  |  |  |  |  |  |  |
|  |  |  | Persistent (all participants) |  |  |  |  |  |  |  |
| Hussein et al, 2023^254^ | USA | Cohort study | 166 | AFEQT | / | / | / | / | / | / |
|  |  |  | 63.2±11.0 years |  |  |  |  |  |  |  |
|  |  |  | 39.2% |  |  |  |  |  |  |  |
|  |  |  | NR |  |  |  |  |  |  |  |
|  |  |  | Paroxysmal (all participants) |  |  |  |  |  |  |  |
| Hussein et al, 2019^255^ | USA | Cohort study | 2,530 | / | / | / | / | AFSS | / | / |
|  |  |  | 63.6±10.1 years |  |  |  |  |  |  |  |
|  |  |  | 28.3% |  |  |  |  |  |  |  |
|  |  |  | NR |  |  |  |  |  |  |  |
|  |  |  | Paroxysmal (50.5%) & non-paroxysmal (49.5%) |  |  |  |  |  |  |  |
| Hutchings et al, 2022^256^ | UK | Cross-sectional study (protocol) | 200 participants to be enrolled | AFEQT & EQ-5D-5L | / | / | / | / | / | / |
|  |  |  | Participants aged ≥18 to be enrolled |  |  |  |  |  |  |  |
|  |  |  | NR |  |  |  |  |  |  |  |
|  |  |  | NR |  |  |  |  |  |  |  |
|  |  |  | NR |  |  |  |  |  |  |  |
| Hutchings et al, 2023^257^ | UK | Cross-sectional study | Demographic data only available for 14 participants (total n=26) | AFEQT & EQ-5D-5L | / | / | / | / | / | / |
|  |  |  | NR |  |  |  |  |  |  |  |
|  |  |  | 78.6% |  |  |  |  |  |  |  |
|  |  |  | White (100%) |  |  |  |  |  |  |  |
|  |  |  | NR |  |  |  |  |  |  |  |
| Ibáñez et al, 2020^258^ | Spain | Cohort study | 11 | AF-QoL & SF-36 | / | / | / | / | / | / |
|  |  |  | Mean age reported: 63 (IQR range 55-65) years |  |  |  |  |  |  |  |
|  |  |  | 45.5% |  |  |  |  |  |  |  |
|  |  |  | NR |  |  |  |  |  |  |  |
|  |  |  | Refractory (all participants) |  |  |  |  |  |  |  |
| Ikemura et al, 2019^259^ | Japan | Cohort study (registry) | 539 | AFEQT [Japanese] | / | / | / | / | / | / |
|  |  |  | 57±10 years |  |  |  |  |  |  |  |
|  |  |  | 27.8% |  |  |  |  |  |  |  |
|  |  |  | NR |  |  |  |  |  |  |  |
|  |  |  | NR |  |  |  |  |  |  |  |
| Ikemura et al, 2019 [a] ^260^ | Japan | Cohort study (registry) | 2,464 | AFEQT [Japanese] | / | / | / | / | / | / |
|  |  |  | 67.1±11.7 years |  |  |  |  |  |  |  |
|  |  |  | 30.3% |  |  |  |  |  |  |  |
|  |  |  | NR |  |  |  |  |  |  |  |
|  |  |  | First detected (6%), paroxysmal (49.2%), persistent (27%) & permanent (15.2%) |  |  |  |  |  |  |  |
| Ikemura et al, 2024^261^ | Japan | Cohort study | 330 | AFEQT | / | / | / | / | / | / |
|  |  |  | 67.9±11.9 years |  |  |  |  |  |  |  |
|  |  |  | 27.9% |  |  |  |  |  |  |  |
|  |  |  | NR |  |  |  |  |  |  |  |
|  |  |  | Paroxysmal (49.4%) & not specified (50.6%) |  |  |  |  |  |  |  |
| Inagaki et al, 2020^262^ | Japan | Cohort study | 119 | AFQLQ | / | / | / | / | / | / |
|  |  |  | 66.3±9.5 years |  |  |  |  |  |  |  |
|  |  |  | 26.1% |  |  |  |  |  |  |  |
|  |  |  | NR |  |  |  |  |  |  |  |
|  |  |  | Paroxysmal (87.4%) & persistent (12.6%) |  |  |  |  |  |  |  |
| Iroegbu et al, 2020^263^ | China | Cohort study | 10,326 | EQ-5D & SF-36 | / | / | / | / | / | / |
|  |  |  | Mitral + tricuspid + CABG + Cox Maze IV group (n=1,012): 54.6±10.1 years Aortic + CABG + Cox Maze IV group (n=819): 58.4±12.7 years Mitral + tricuspid + aortic + CABG + Cox Maze IV group: (1,146): 70.9±8.1 years  Isolated CABG + Cox Maze IV group: (n=91): 57.5±13.3 years  Surgery as usual group (n=7,258): 60.3±14.9 years |  |  |  |  |  |  |  |
|  |  |  | Mitral + tricuspid + CABG + Cox Maze IV group: 82.7%  Aortic + CABG + Cox Maze IV group: 37.9%  Mitral + tricuspid + aortic + CABG + Cox Maze IV group: 76.1%  Isolated CABG + Cox Maze IV group: 35.2%  Surgery as usual group: 49% |  |  |  |  |  |  |  |
|  |  |  | NR |  |  |  |  |  |  |  |
|  |  |  | Mitral + tricuspid + CABG + Cox Maze group: paroxysmal (48.8%), persistent (28.9%) & long-standing persistent (22.3%) Aortic + CABG + Cox Maze IV group: paroxysmal (41%), persistent (41.6%) & long-standing persistent (17.3%)  Mitral + tricuspid + aortic + CABG + Cox Maze IV group: paroxysmal (47.7%), persistent (31.2%) & long-standing persistent (21.1%)  Isolated CABG + Cox Maze IV group: paroxysmal (67%), persistent (0%) & long-standing persistent (33%)  Surgery as usual group: paroxysmal (43.7%), persistent (27.5%) & long-standing persistent (36.7%) |  |  |  |  |  |  |  |
| Ishiguro et al, 2008^264^ | Japan | Cohort study | 136 | AFQLQ | / | / | / | / | / | / |
|  |  |  | 66.6±14.6 years |  |  |  |  |  |  |  |
|  |  |  | 45.6% |  |  |  |  |  |  |  |
|  |  |  | NR |  |  |  |  |  |  |  |
|  |  |  | Paroxysmal (all participants) |  |  |  |  |  |  |  |
| Jeong et al, 2014^265^ | South Korea | Cohort study | 216 | SF-12 | / | / | / | / | / | / |
|  |  |  | 60.4±8.8 years |  |  |  |  |  |  |  |
|  |  |  | High mental component score group (n=54): 13%  Low mental component score group (n=54): 39%  High physical component score group (n=41): 15%  Low physical component score group (n=67): 33% |  |  |  |  |  |  |  |
|  |  |  | NR |  |  |  |  |  |  |  |
|  |  |  | NR |  |  |  |  |  |  |  |
| Jackson et al, 2021^266^ | Sweden | Cohort study | 30 | SF-36 | / | / | / | AF6 | / | / |
|  |  |  | 69±6 years |  |  |  |  |  |  |  |
|  |  |  | 40% |  |  |  |  |  |  |  |
|  |  |  | NR |  |  |  |  |  |  |  |
|  |  |  | Permanent (all participants) |  |  |  |  |  |  |  |
| Jain et al, 2020^267^ | Canada & USA | RCT | 344 | SF-12 | / | / | / | / | / | / |
|  |  |  | 60.2±10.4 years |  |  |  |  |  |  |  |
|  |  |  | 33.7% |  |  |  |  |  |  |  |
|  |  |  | NR |  |  |  |  |  |  |  |
|  |  |  | Paroxysmal (all participants) |  |  |  |  |  |  |  |
| Jaïs et al, 2008^268^ | Countries in North America & Europe (not specified) | RCT | 112 | SF-36 | / | / | / | SCL | / | / |
|  |  |  | 51.1±11.1 years |  |  |  |  |  |  |  |
|  |  |  | 16.1% |  |  |  |  |  |  |  |
|  |  |  | NR |  |  |  |  |  |  |  |
|  |  |  | Paroxysmal (all participants) |  |  |  |  |  |  |  |
| Jankowska-Polańska et al, 2018^269^ | Poland | Cross-sectional study | 99 | ASTA HRQoL scale & WHOQoL-BREF [Polish] | / | / | / | ASTA symptom scale | / | / |
|  |  |  | 64.65±10.63 years |  |  |  |  |  |  |  |
|  |  |  | 55.6% |  |  |  |  |  |  |  |
|  |  |  | NR |  |  |  |  |  |  |  |
|  |  |  | NR |  |  |  |  |  |  |  |
| Jankowska-Polańska et al, 2016^270^ | Poland | Cohort study | 111 | / | / | / | MMSE | / | / | / |
|  |  |  | 73.5±8.3 years |  |  |  |  |  |  |  |
|  |  |  | 49.5% |  |  |  |  |  |  |  |
|  |  |  | NR |  |  |  |  |  |  |  |
|  |  |  | Low treatment adherence group (n=52): paroxysmal (42.3%), persistent (17.3%) & permanent (40.4%)  Medium treatment adherence group (n=22): paroxysmal (50.1%), persistent (18.2%) & permanent (31.8%)  High treatment adherence group (n=37): paroxysmal (48.6%), persistent (21.6%) & permanent (29.7%) |  |  |  |  |  |  |  |
| Jansson et al, 2021^271^ | Sweden | RCT | 150 | SF-36 | / | / | / | / | / | / |
|  |  |  | Median age reported: 58 (IQR range 50-64) years |  |  |  |  |  |  |  |
|  |  |  | 23% |  |  |  |  |  |  |  |
|  |  |  | NR |  |  |  |  |  |  |  |
|  |  |  | Paroxysmal (72%) & persistent (28%) |  |  |  |  |  |  |  |
| Jasper et al, 2005^272^ | NR | RCT | 1,074 | / | DASI | / | / | / | / | / |
|  |  |  | Transoesophageal echocardiography group (n=544): 64.9±12.8 years & conventional group (n=530): 64.8±13.1 years |  |  |  |  |  |  |  |
|  |  |  | Transoesophageal echocardiography group: 33.3% & conventional group: 31.7% |  |  |  |  |  |  |  |
|  |  |  | NR |  |  |  |  |  |  |  |
|  |  |  | NR |  |  |  |  |  |  |  |
| Jenkins et al, 2005^273^ | Canada & USA | RCT | 716 | SF-36 & QLI-CV | / | / | / | SCL | / | / |
|  |  |  | 70.0±9.0 years |  |  |  |  |  |  |  |
|  |  |  | 38% |  |  |  |  |  |  |  |
|  |  |  | White (93%) |  |  |  |  |  |  |  |
|  |  |  | NR |  |  |  |  |  |  |  |
| Jessurun et al, 2003^274^ | Netherlands | RCT | 35 | SF-36 | / | / | / | / | / | / |
|  |  |  | Maze surgery group (n=25): 64±12 years & no maze surgery group (n=10): 64±9 years |  |  |  |  |  |  |  |
|  |  |  | 45.7% |  |  |  |  |  |  |  |
|  |  |  | NR |  |  |  |  |  |  |  |
|  |  |  | Chronic (57.1%) & paroxysmal (42.9%) |  |  |  |  |  |  |  |
| Ji-Yeong et al, 2023^275^ | South Korea | Cross-sectional study | 158 | SF-36 [Korean] | / | / | / | / | / | / |
|  |  |  | 61.01±9.56 years |  |  |  |  |  |  |  |
|  |  |  | 33.5% |  |  |  |  |  |  |  |
|  |  |  | NR |  |  |  |  |  |  |  |
|  |  |  | Paroxysmal (27.8%), persistent (11.4%) & permanent (60.8%) |  |  |  |  |  |  |  |
| Jia et al, 2021^276^ | China | Cross-sectional study | 922 with AF (total n=47,588) | / | / | CES-D [Chinese] & SAS [Chinese] | / | / | / | / |
|  |  |  | 67.1±9.9 years |  |  |  |  |  |  |  |
|  |  |  | 54.8% |  |  |  |  |  |  |  |
|  |  |  | Han (96.9%) & Other (29%) |  |  |  |  |  |  |  |
|  |  |  | NR |  |  |  |  |  |  |  |
| Jin et al, 2019^277^ | South Korea | Case-control study | 308 | / | / | / | MoCA [Korean] | / | / | / |
|  |  |  | 60.6±9.1 years |  |  |  |  |  |  |  |
|  |  |  | 28.6% |  |  |  |  |  |  |  |
|  |  |  | NR |  |  |  |  |  |  |  |
|  |  |  | Persistent (34.1%) & not specified (65.9%) |  |  |  |  |  |  |  |
| Joensen et al, 2019^278^ | Denmark | RCT | 52 | AFEQT, AF-QoL & EQ-5D | / | GAD-7 & PHQ-9 | / | / | / | / |
|  |  |  | Intervention group (n=28): 62.2±10.0 years & control group (n=24): 60.2±8.9 years |  |  |  |  |  |  |  |
|  |  |  | Intervention group: 39% & control group: 29% |  |  |  |  |  |  |  |
|  |  |  | NR |  |  |  |  |  |  |  |
|  |  |  | Intervention group: paroxysmal (57%) & persistent (43%) & control group: paroxysmal (38%) & persistent (63%) |  |  |  |  |  |  |  |
| Johannson et al, 2008^279^ | NR | RCT | 78 | SF-36 | / | / | / | SCL | / | / |
|  |  |  | Ablation group (n=39): 73±5 years & control group (n=39): 72±6 years |  |  |  |  |  |  |  |
|  |  |  | 20.5% |  |  |  |  |  |  |  |
|  |  |  | NR |  |  |  |  |  |  |  |
|  |  |  | Paroxysmal or persistent (48.7%) & permanent (51.3%) |  |  |  |  |  |  |  |
| Johnson et al, 2023^280^ | USA | Cohort study | 241 | / | / | / | / | AFSS | / | / |
|  |  |  | 43.1±7.3 years |  |  |  |  |  |  |  |
|  |  |  | 17% |  |  |  |  |  |  |  |
|  |  |  | White (87.6%), Black (3.7%), Asian (0.8%), American Indian or Alaska Native (0.4%), multi-racial (6.2%) & declined to declare (1.2%) |  |  |  |  |  |  |  |
|  |  |  | Paroxysmal (59.3%), persistent (40.3%) & unknown (0.4%) |  |  |  |  |  |  |  |
| Jones et al, 2020^281^ | UK | RCT | 19 | AFEQT, EQ-5D-3L & SF-36 | / | / | / | / | / | / |
|  |  |  | 74±7 years |  |  |  |  |  |  |  |
|  |  |  | 42.1% |  |  |  |  |  |  |  |
|  |  |  | White (100%) |  |  |  |  |  |  |  |
|  |  |  | Permanent (all participants) |  |  |  |  |  |  |  |
| Jons et al, 2009^282^ | Countries in Germany and Scandinavia (not specified) | RCT (protocol) | 300 participants to be enrolled | SF-36 | / | / | / | / | / | / |
|  |  |  | Participants aged ≤70 years to be enrolled |  |  |  |  |  |  |  |
|  |  |  | NR |  |  |  |  |  |  |  |
|  |  |  | NR |  |  |  |  |  |  |  |
|  |  |  | Paroxysmal AF participants to be enrolled |  |  |  |  |  |  |  |
| Joosten et al, 2019^283^ | Netherlands | RCT (protocol) | 1,250 participants to be enrolled | EQ-5D-5L | / | / | / | / | / | / |
|  |  |  | Participants aged ≥75 years to be enrolled |  |  |  |  |  |  |  |
|  |  |  | NR |  |  |  |  |  |  |  |
|  |  |  | NR |  |  |  |  |  |  |  |
|  |  |  | Non-valvular AF participants to be enrolled |  |  |  |  |  |  |  |
| Joshibayev et al, 2016^284^ | Kazakhstan | Cohort study | 147 | SF-36 | / | / | / | / | / | / |
|  |  |  | Study group (n=54): 57±8 years & control group (n=93): 52±9 years |  |  |  |  |  |  |  |
|  |  |  | Study group: 42% & control group: 46% |  |  |  |  |  |  |  |
|  |  |  | NR |  |  |  |  |  |  |  |
|  |  |  | Study group: paroxysmal (0%), persistent (4%) & long-standing persistent (96%)  Control group: paroxysmal (3%), persistent (29%) & long-standing persistent (68%) |  |  |  |  |  |  |  |
| Ju Yuon, et al 2022^285^ | South Korea | Cohort study (registry) | 1,515 | AFEQT [Korean] | / | / | / | / | / | / |
|  |  |  | 65.7±10.5 years |  |  |  |  |  |  |  |
|  |  |  | 34.1% |  |  |  |  |  |  |  |
|  |  |  | NR |  |  |  |  |  |  |  |
|  |  |  | Paroxysmal (63.3%) & persistent (36.7%) |  |  |  |  |  |  |  |
| Kanthasamy et al, 2024^286^ | UK | RCT | 20 | EQ-5D-5L, SF-12 | / | / | / | / | / | / |
|  |  |  | Pulmonary vein isolation group (n=10): 69±6 years & DC cardioversion group (n=10): 72±7 years |  |  |  |  |  |  |  |
|  |  |  | 30% |  |  |  |  |  |  |  |
|  |  |  | White (80%) & Other (20%) |  |  |  |  |  |  |  |
|  |  |  | Persistent (all participants) |  |  |  |  |  |  |  |
| Kahya Eren et al, 2014^287^ | Turkey | Methodological study | 130 | SF-36 [Turkish] | / | / | / | AFSS [Turkish] | / | / |
|  |  |  | 63.1±10.9 years |  |  |  |  |  |  |  |
|  |  |  | 41.5% |  |  |  |  |  |  |  |
|  |  |  | NR |  |  |  |  |  |  |  |
|  |  |  | Paroxysmal (30%), persistent (6.2%) & permanent (63.8%) |  |  |  |  |  |  |  |
| Kale et al, 2002^288^ | UK | Cohort study | 28 | SF-36 | / | / | / | / | / | / |
|  |  |  | 58 years |  |  |  |  |  |  |  |
|  |  |  | 17.9% |  |  |  |  |  |  |  |
|  |  |  | NR |  |  |  |  |  |  |  |
|  |  |  | Paroxysmal (all participants) |  |  |  |  |  |  |  |
| Kamalvand et al, 1997^289^ | NR | RCT | 48 | / | / | / | / | / | Specific activity scale | / |
|  |  |  | 64±13 years |  |  |  |  |  |  |  |
|  |  |  | 42% |  |  |  |  |  |  |  |
|  |  |  | NR |  |  |  |  |  |  |  |
|  |  |  | Paroxysmal (all participants) |  |  |  |  |  |  |  |
| Kanagaratnam et al, 2022^290^ | UK | RCT | 321 | AFEQT & EQ-5D-5L | / | / | / | / | / | / |
|  |  |  | Protocol arm group (n=110): 60.1±9.00 years, anti-arrhythmic arm group (n=103): 60.5±10.34 years & conventional ablation arm group (n=108): 59.7±12.22 years |  |  |  |  |  |  |  |
|  |  |  | Protocol arm group: 42%, anti-arrhythmic arm group: 45% & conventional ablation arm group: 39% |  |  |  |  |  |  |  |
|  |  |  | NR |  |  |  |  |  |  |  |
|  |  |  | Paroxysmal (all participants) |  |  |  |  |  |  |  |
| Kang, 2005^291^ | USA | Cross-sectional study | 81 | SF-36 | / | / | / | SCL | / | / |
|  |  |  | 67.3 years |  |  |  |  |  |  |  |
|  |  |  | 49.4% |  |  |  |  |  |  |  |
|  |  |  | White (88.9%) & Black (11.1%) |  |  |  |  |  |  |  |
|  |  |  | NR |  |  |  |  |  |  |  |
| Kang, 2009^292^ | South Korea & USA | Cohort study | 129 | SF-36 | / | / | / | / | / | / |
|  |  |  | American group (n=81): 67.3±12.05 years & Korean group (n=38): 59.1±9.91 years |  |  |  |  |  |  |  |
|  |  |  | American group: 49.4% & Korean group: 50% |  |  |  |  |  |  |  |
|  |  |  | NR |  |  |  |  |  |  |  |
|  |  |  | NR |  |  |  |  |  |  |  |
| Kang, 2011^293^ | South Korea | Cross-sectional study | 109 | / | / | / | / | SCL | / | / |
|  |  |  | 60.2 years |  |  |  |  |  |  |  |
|  |  |  | 35.8% |  |  |  |  |  |  |  |
|  |  |  | NR |  |  |  |  |  |  |  |
|  |  |  | NR |  |  |  |  |  |  |  |
| Kang et al, 2004^294^ | USA | Cross-sectional study | 81 | SF-36 | / | / | / | SCL | / | / |
|  |  |  | 67.3 years |  |  |  |  |  |  |  |
|  |  |  | 49.4% |  |  |  |  |  |  |  |
|  |  |  | White (88.9%) & Black (11.1%) |  |  |  |  |  |  |  |
|  |  |  | Newly diagnosed (all participants) |  |  |  |  |  |  |  |
| Kato et al, 2019^295^ | Japan | Cohort study | 60 | SF-36 | / | / | / | / | / | / |
|  |  |  | 65.5±9.5 years |  |  |  |  |  |  |  |
|  |  |  | 23.3% |  |  |  |  |  |  |  |
|  |  |  | NR |  |  |  |  |  |  |  |
|  |  |  | Paroxysmal (63.3%) & persistent (36.7%) |  |  |  |  |  |  |  |
| Kato et al, 2021^296^ | Japan | Cohort study | 74 | / | / | / | MMSE | / | / | / |
|  |  |  | 68.3±10.0 years |  |  |  |  |  |  |  |
|  |  |  | 28.4% |  |  |  |  |  |  |  |
|  |  |  | NR |  |  |  |  |  |  |  |
|  |  |  | Paroxysmal (44.6%) & persistent (55.4%) |  |  |  |  |  |  |  |
| Katsumata et al, 2020^297^ | Japan | Cohort study (registry) | 1,493 | AFEQT | / | / | / | / | / | / |
|  |  |  | 67±11 years |  |  |  |  |  |  |  |
|  |  |  | 29% |  |  |  |  |  |  |  |
|  |  |  | NR |  |  |  |  |  |  |  |
|  |  |  | Paroxysmal (52%), persistent (28%) & permanent (14%) |  |  |  |  |  |  |  |
| Kay et al, 1988^298^ | USA | Cohort study | 12 | MHIQ | / | PGWB | / | / | / | / |
|  |  |  | 67 years |  |  |  |  |  |  |  |
|  |  |  | 33.3% |  |  |  |  |  |  |  |
|  |  |  | NR |  |  |  |  |  |  |  |
|  |  |  | Paroxysmal (all participants) |  |  |  |  |  |  |  |
| Kay et al, 1998^299^ | USA | RCT | 156 | HSQ & QLI-CV | / | / | / | SCL | / | / |
|  |  |  | 66.1±11.5 years |  |  |  |  |  |  |  |
|  |  |  | 42.3% |  |  |  |  |  |  |  |
|  |  |  | NR |  |  |  |  |  |  |  |
|  |  |  | Chronic (44.9%), recurrent (19.9%) & paroxysmal (35.2%) |  |  |  |  |  |  |  |
| Khalifehzadeh-Esfahani et al, 2018^300^ | Iran | RCT | 50 | SF-36 [Persian] | / | / | / | / | / | / |
|  |  |  | Study group (n=25): 65.70±11.30 years & control group (n=25): 66.40±16.70 years |  |  |  |  |  |  |  |
|  |  |  | 26% |  |  |  |  |  |  |  |
|  |  |  | NR |  |  |  |  |  |  |  |
|  |  |  | NR |  |  |  |  |  |  |  |
| Khan et al, 2018^301^ | UK | RCT (protocol) | 120 participants to be enrolled | AFEQT & EQ-5D-5L | / | / | / | / | / | / |
|  |  |  | Participants aged ≥18 years to be enrolled |  |  |  |  |  |  |  |
|  |  |  | NR |  |  |  |  |  |  |  |
|  |  |  | NR |  |  |  |  |  |  |  |
|  |  |  | Long-standing persistent AF participants to be enrolled |  |  |  |  |  |  |  |
| Khan et al, 2004^302^ | UK | RCT | 79 | EQ-5D & SF-36 | / | / | / | / | / | / |
|  |  |  | Median age reported: education and clinic care group (n=39): 75 (IQR range 65-67) years & weekly self-monitoring group (n=40): 71 (IQR range 65-91) |  |  |  |  |  |  |  |
|  |  |  | 43% |  |  |  |  |  |  |  |
|  |  |  | NR |  |  |  |  |  |  |  |
|  |  |  | NR |  |  |  |  |  |  |  |
| Kilander et al, 1998^303^ | Sweden | Cross-sectional study | 44 with AF (total n=952) | / | / | / | MMSE | / | / | / |
|  |  |  | 72.4±1.1 years |  |  |  |  |  |  |  |
|  |  |  | 0% |  |  |  |  |  |  |  |
|  |  |  | NR |  |  |  |  |  |  |  |
|  |  |  | NR |  |  |  |  |  |  |  |
| Kim et al, 2024^304^ | South Korea | RCT (protocol) | 840 participants to be enrolled | AFEQT | / | / | MoCA [Korean] |  |  |  |
|  |  |  | Participants aged 19-80 years to be enrolled |  |  |  |  |  |  |  |
|  |  |  | NR |  |  |  |  |  |  |  |
|  |  |  | NR |  |  |  |  |  |  |  |
|  |  |  | Non-valvular AF participants to be enrolled |  |  |  |  |  |  |  |
| Kim et al, 2023^305^ | South Korea | RCT | 74 | SF-36 [Korean] | / | / | / | / | / | / |
|  |  |  | 63±6 years |  |  |  |  |  |  |  |
|  |  |  | Medical treatment only group (n=31): 38.7%, detraining group (n=30): 26.7%) & continuous exercise training group (n=30): 23.3% |  |  |  |  |  |  |  |
|  |  |  | NR |  |  |  |  |  |  |  |
|  |  |  | Non-permanent (all participants) |  |  |  |  |  |  |  |
| Kimura et al, 2022^306^ | Japan | Cohort study (registry) | 352 | EQ-5D-3L | / | / | / | / | / | / |
|  |  |  | 65±10 years |  |  |  |  |  |  |  |
|  |  |  | 36% |  |  |  |  |  |  |  |
|  |  |  | NR |  |  |  |  |  |  |  |
|  |  |  | Paroxysmal (all participants) |  |  |  |  |  |  |  |
| Kirchhof et al, 2013^307^ | 11 European countries (not specified) | RCT (protocol) | 2,810 participants to be enrolled | EQ-5D & SF-12 | / | / | MoCA | / | / | / |
|  |  |  | Participants aged ≥18 years to be enrolled |  |  |  |  |  |  |  |
|  |  |  | NR |  |  |  |  |  |  |  |
|  |  |  | NR |  |  |  |  |  |  |  |
|  |  |  | Participants with recent-onset AF to be enrolled |  |  |  |  |  |  |  |
| Kirchhof et al, 2020^308^ | Belgium, Czech Republic, Denmark, France, Germany, Greece, Italy, Netherlands,  Poland, Spain & UK | RCT | 2,789 | EQ-5D & SF-12 | / | / | MoCA | / | / | / |
|  |  |  | Early rhythm control group (n=1,395): 70.2±8.4 years & usual care group (n=1,394): 70.4±8.2 years |  |  |  |  |  |  |  |
|  |  |  | Early rhythm control group: 46.2% & usual care group: 46.5% |  |  |  |  |  |  |  |
|  |  |  | NR |  |  |  |  |  |  |  |
|  |  |  | Early rhythm control group: first episode (38%), paroxysmal (36%) & persistent (26%)  Usual care group: first episode (37.3%), paroxysmal (35.4%) & persistent (27.3%) |  |  |  |  |  |  |  |
| Kirchhof et al, 2018^309^ | Belgium, Denmark, Italy, Netherlands, German, Austria, Spain, USA & UK | RCT | 633 | EQ-5D & SF-12 | KPS | / | MoCA | / | / | / |
|  |  |  | Median age reported: 64 (IQR range 58-70) years |  |  |  |  |  |  |  |
|  |  |  | 33% |  |  |  |  |  |  |  |
|  |  |  | NR |  |  |  |  |  |  |  |
|  |  |  | Paroxysmal (58%) & persistent or long-standing persistent (42%) |  |  |  |  |  |  |  |
| Kjolseth et al, 2025^310^ | Denmark & Norway | Cohort study | 408 with AF (total n=3,417) | SF-12 | / | / | / | / | / | / |
|  |  |  | 72±9 years |  |  |  |  |  |  |  |
|  |  |  | 19% |  |  |  |  |  |  |  |
|  |  |  | NR |  |  |  |  |  |  |  |
|  |  |  | NR |  |  |  |  |  |  |  |
| Kloosterman et al, 2020^311^ | Countries in Europe and North America (not specified) | RCT | 633 | EQ-5D & SF-12 | KPS | / | MoCA | / | / | / |
|  |  |  | Median age reported: 64 (IQR range 58-70) years |  |  |  |  |  |  |  |
|  |  |  | 33% |  |  |  |  |  |  |  |
|  |  |  | NR |  |  |  |  |  |  |  |
|  |  |  | Paroxysmal (58%) & persistent or long-standing persistent (42%) |  |  |  |  |  |  |  |
| Koca et al, 2020^312^ | Turkey | Cross-sectional study | 69 with AF (total n=123) | / | ADL & IADL | GDS | MMSE | / | / | / |
|  |  |  | 75.31±5.67 years |  |  |  |  |  |  |  |
|  |  |  | 54.7% |  |  |  |  |  |  |  |
|  |  |  | NR |  |  |  |  |  |  |  |
|  |  |  | Long-standing persistent (70.3%) |  |  |  |  |  |  |  |
| Kocheril et al, 2005^313^ | USA | Cohort study | 93 | SF-36 | / | / | / | AFSS | / | / |
|  |  |  | Men group (n=69): 56.8 years & women group (n=24): 62.2 years |  |  |  |  |  |  |  |
|  |  |  | 25.8% |  |  |  |  |  |  |  |
|  |  |  | NR |  |  |  |  |  |  |  |
|  |  |  | Paroxysmal (all participants) |  |  |  |  |  |  |  |
| Kochhäuser et al, 2016^314^ | Canada & USA | Cohort study | 410 with AF (total n=798) | SF-12 | / | / | / | SCL | / | / |
|  |  |  | AT or AF ≤ 30 minutes group (n=145): 72±10 years AT or AF 30 minutes-2 hours group (n=81): 70±10 years  AT or AF > 2 hours group (n=184): 70±11 years |  |  |  |  |  |  |  |
|  |  |  | AT or AF ≤ 30 minutes group: 50% AT or AF 30 minutes-2 hours group: 44%  AT or AF > 2 hours group: 36% |  |  |  |  |  |  |  |
|  |  |  | NR |  |  |  |  |  |  |  |
|  |  |  | NR |  |  |  |  |  |  |  |
| Koci et al, 2014^315^ | USA | Methodological study | 234 | SF-12v2 | / | / | / | AFS/B | / | / |
|  |  |  | 63.7±11.0 years |  |  |  |  |  |  |  |
|  |  |  | 26% |  |  |  |  |  |  |  |
|  |  |  | NR |  |  |  |  |  |  |  |
|  |  |  | Paroxysmal (55%), persistent (33%) & long-standing persistent (13%) |  |  |  |  |  |  |  |
| Kooistra et al, 2016^316^ | Netherlands | Cohort study | 807 | SF-36 [Dutch] | / | / | / | / | / | / |
|  |  |  | Grouped ages reported: New group (n=240): <56 years (n=19), 56-65 years (n=17), 66-75 years (n=39) & ≥76 years (n=36) Long-term group (n=567): <56 years (n=1), 56-65 years (n=10), 66-75 years (n=35) & ≥76 years (n=54) |  |  |  |  |  |  |  |
|  |  |  | New group: 43% & long-term group: 47% |  |  |  |  |  |  |  |
|  |  |  | NR |  |  |  |  |  |  |  |
|  |  |  | NR |  |  |  |  |  |  |  |
| Kosior et al, 2018^317^ | Poland | RCT | 205 | SF-36 [Polish] | / | / | / | / | / | / |
|  |  |  | 60.8±11.2 years |  |  |  |  |  |  |  |
|  |  |  | 65.4% |  |  |  |  |  |  |  |
|  |  |  | NR |  |  |  |  |  |  |  |
|  |  |  | Persistent (all participants) |  |  |  |  |  |  |  |
| Kosmalska et al, 2021^318^ | Poland | Cohort study | 269 | / | / | / | MMSE | / | / | / |
|  |  |  | With left atrial appendage thrombi group: 75.76±8.26 years & without left atrial appendage thrombi group: 73.01±10.19 years |  |  |  |  |  |  |  |
|  |  |  | With left atrial appendage thrombi group: 53.16% & without left atrial appendage thrombi group: 43.16% |  |  |  |  |  |  |  |
|  |  |  | NR |  |  |  |  |  |  |  |
|  |  |  | Persistent (all participants) |  |  |  |  |  |  |  |
| Kotalczyk et al, 2022^319^ | China | Cohort study (registry) | 3,938 | EQ-5D-5L | / | / | / | / | / | / |
|  |  |  | Multimorbidity group (n=2,610): 74.4±10.2 years & polypharmacy group (n=1,328): 74.6±10.1 years |  |  |  |  |  |  |  |
|  |  |  | Multimorbidity group: 42.8% & polypharmacy group: 43.3% |  |  |  |  |  |  |  |
|  |  |  | NR |  |  |  |  |  |  |  |
|  |  |  | NR |  |  |  |  |  |  |  |
| Kotecha et al, 2020^320^ | UK | RCT | 160 | AFEQT, EQ-5D-5L & SF-36 | / | / | / | / | / | / |
|  |  |  | 76±8 years |  |  |  |  |  |  |  |
|  |  |  | 46% |  |  |  |  |  |  |  |
|  |  |  | Asian or Asian British (5%), Black or Black British (1.9%) & White (93.1%) |  |  |  |  |  |  |  |
|  |  |  | Permanent (all participants) |  |  |  |  |  |  |  |
| Kotecha et al, 2017^321^ | UK | RCT (protocol) | 160 participants to be enrolled | AFEQT, EQ-5D-5L & SF-36 | / | / | / | / | / | / |
|  |  |  | Participants aged ≥60 years to be enrolled |  |  |  |  |  |  |  |
|  |  |  | NR |  |  |  |  |  |  |  |
|  |  |  | NR |  |  |  |  |  |  |  |
|  |  |  | Permanent AF participants to be enrolled |  |  |  |  |  |  |  |
| Koutoukidis et al, 2020^322^ | UK | Cross-sectional study | 882 | EQ-5D-5L | / | / | / | AFSS | / | / |
|  |  |  | 74.3±10.2 years |  |  |  |  |  |  |  |
|  |  |  | 35.8% |  |  |  |  |  |  |  |
|  |  |  | White (98.1%) |  |  |  |  |  |  |  |
|  |  |  | Paroxysmal, persistent & permanent (n or % not specified) |  |  |  |  |  |  |  |
| Kowalewski et al, 2022^323^ | Poland | Cohort study (protocol) | 200 participants to be enrolled per year | EQ-5D-5L | / | / | / | / | / | / |
|  |  |  | Participants aged ≥18 years to be enrolled |  |  |  |  |  |  |  |
|  |  |  | NR |  |  |  |  |  |  |  |
|  |  |  | NR |  |  |  |  |  |  |  |
|  |  |  | Non-valvular AF participants to be enrolled |  |  |  |  |  |  |  |
| Kristensen et al, 2016^324^ | Denmark | Methodological study | 462 responded to PROMs (total n=627) | SF-36 | / | HADS | / | / | / | / |
|  |  |  | 61.1±9.9 years |  |  |  |  |  |  |  |
|  |  |  | 26.8% |  |  |  |  |  |  |  |
|  |  |  | NR |  |  |  |  |  |  |  |
|  |  |  | Paroxysmal & persistent (n or % not specified) |  |  |  |  |  |  |  |
| Kuck et al, 2016^325^ | NR | RCT | 750 | EQ-5D-3L & SF-12 | / | / | / | / | / | / |
|  |  |  | NR |  |  |  |  |  |  |  |
|  |  |  | NR |  |  |  |  |  |  |  |
|  |  |  | NR |  |  |  |  |  |  |  |
|  |  |  | Paroxysmal (all participants) |  |  |  |  |  |  |  |
| Kupper et al, 2018^326^ | Netherlands | Cohort study | 159 | AF-QoL [Dutch] & SF-36 [Dutch] | / | HADS [Dutch] | / | AFSS | / | / |
|  |  |  | 61.6±0.8 years |  |  |  |  |  |  |  |
|  |  |  | 37% |  |  |  |  |  |  |  |
|  |  |  | NR |  |  |  |  |  |  |  |
|  |  |  | Paroxysmal (72%), persistent (25%) & permanent (3%) |  |  |  |  |  |  |  |
| Kupper et al, 2013^327^ | Netherlands | Cohort study | 118 | / | / | BDI, PSS-10 & STAI | / | AFSS | / | / |
|  |  |  | 68.0±9.4 years |  |  |  |  |  |  |  |
|  |  |  | 38.8% |  |  |  |  |  |  |  |
|  |  |  | NR |  |  |  |  |  |  |  |
|  |  |  | Persistent (all participants) |  |  |  |  |  |  |  |
| Kwon et al, 2021^328^ | Canada | Cohort study | 7,439 responded to PROM (total n=13,113) | AFEQT | / | / | / | / | / | / |
|  |  |  | Median age reported: 67 years (IQR range 16) |  |  |  |  |  |  |  |
|  |  |  | 38.9% |  |  |  |  |  |  |  |
|  |  |  | NR |  |  |  |  |  |  |  |
|  |  |  | NR |  |  |  |  |  |  |  |
| Lakkireddy et al, 2013^329^ | USA | Cohort study | 49 | SF-36 | / | SAS & SDS | / | / | / | / |
|  |  |  | 60.6±11.5 years |  |  |  |  |  |  |  |
|  |  |  | 53.1% |  |  |  |  |  |  |  |
|  |  |  | NR |  |  |  |  |  |  |  |
|  |  |  | Paroxysmal (all participants) |  |  |  |  |  |  |  |
| Lai et al, 2022^330^ | China | Methodological study | 101 | / | / | / | MMSE [Chinese] & T-MoCA [Chinese] | / | / | / |
|  |  |  | Clinical dementia rating 0.5 group (n=35): 62.8±7.5 years & clinical dementia rating 0 group (n=66): 61.3±8.7 years |  |  |  |  |  |  |  |
|  |  |  | Clinical dementia rating 0.5 group: 42.9% & clinical dementia rating 0 group: 39.4% |  |  |  |  |  |  |  |
|  |  |  | NR |  |  |  |  |  |  |  |
|  |  |  | Clinical dementia rating 0.5 group: paroxysmal (71.4%) & clinical dementia rating 0 group: paroxysmal (59.1%) |  |  |  |  |  |  |  |
| LaMori et al, 2012^331^ | USA | Cohort study | 1,297 | SF-12 | / | / | / | / | / | WPAI |
|  |  |  | AF patients with dyspepsia group (n=499): 62.9±12.9 years & AF patients without dyspepsia group (n=848): 66.0±11.7 years |  |  |  |  |  |  |  |
|  |  |  | AF patients with dyspepsia group: 43% & AF patients without dyspepsia group: 31% |  |  |  |  |  |  |  |
|  |  |  | NR |  |  |  |  |  |  |  |
|  |  |  | NR |  |  |  |  |  |  |  |
| Lane et al, 2009^332^ | UK | Cohort study | 70 | SF-36 | / | BDI-SF, PSS & STAI | / | / | / | / |
|  |  |  | 71.4±9.1 years |  |  |  |  |  |  |  |
|  |  |  | 36% |  |  |  |  |  |  |  |
|  |  |  | Caucasian (100%) |  |  |  |  |  |  |  |
|  |  |  | Persistent (54%) & permanent (46%) |  |  |  |  |  |  |  |
| Lau et al, 2001^333^ | NR | RCT | 22 | SF-36 | / | / | / | SCL | / | / |
|  |  |  | 63±10 years |  |  |  |  |  |  |  |
|  |  |  | 63.6% |  |  |  |  |  |  |  |
|  |  |  | NR |  |  |  |  |  |  |  |
|  |  |  | Paroxysmal (all participants) |  |  |  |  |  |  |  |
| Lawin et al, 2024^334^ | 37 countries worldwide (not specified) | Cohort study (registry) | 1,674 | EQ-5D-3L | / | / | / | / | / | / |
|  |  |  | Octogenarian group (n=101): 82±2 years & control group (n=1,573): 62±10 years |  |  |  |  |  |  |  |
|  |  |  | Octogenarian group: 51.5% & control group: 35.7% |  |  |  |  |  |  |  |
|  |  |  | NR |  |  |  |  |  |  |  |
|  |  |  | Octogenarian group: paroxysmal (70.3%), persistent (24.8%) & long-standing persistent (5%) control group: paroxysmal (74.4%), persistent (19.6%) & long-standing persistent (6%) |  |  |  |  |  |  |  |
| Lazaridis et al, 2022^335^ | Greece | Cohort study | 80 | AFEQT [Greek] & EQ-5D-5L [Greek] | / | / | MoCA | / | / | / |
|  |  |  | 58.1±9.1 years |  |  |  |  |  |  |  |
|  |  |  | 34% |  |  |  |  |  |  |  |
|  |  |  | NR |  |  |  |  |  |  |  |
|  |  |  | Paroxysmal (all participants) |  |  |  |  |  |  |  |
| Lee et al, 2023^336^ | South Korea | RCT (protocol) | 716 to be enrolled | EQ-5D & SF-12 | / | / | / | / | / | / |
|  |  |  | Participants aged between 18-80 years to be enrolled |  |  |  |  |  |  |  |
|  |  |  | NR |  |  |  |  |  |  |  |
|  |  |  | NR |  |  |  |  |  |  |  |
|  |  |  | NR |  |  |  |  |  |  |  |
| Leiner et al, 2023^337^ | Germany | RCT (protocol) | 352 participants to be enrolled | AFEQT | / | PHQ-2 | / | / | / | / |
|  |  |  | Participants aged ≥18 years to be enrolled |  |  |  |  |  |  |  |
|  |  |  | NR |  |  |  |  |  |  |  |
|  |  |  | NR |  |  |  |  |  |  |  |
|  |  |  | NR |  |  |  |  |  |  |  |
| Lelakowski et al, 2008^338^ | Poland | Cohort study | 30 | SF-36 | DASI | / | / | / | / | / |
|  |  |  | 69.8±11.3 years |  |  |  |  |  |  |  |
|  |  |  | 40% |  |  |  |  |  |  |  |
|  |  |  | NR |  |  |  |  |  |  |  |
|  |  |  | Drug refractory (all participants) |  |  |  |  |  |  |  |
| Levy et al, 2001^339^ | UK | RCT | 36 | NHP | / | / | / | / | / | / |
|  |  |  | 69±7 years |  |  |  |  |  |  |  |
|  |  |  | 38.9% |  |  |  |  |  |  |  |
|  |  |  | NR |  |  |  |  |  |  |  |
|  |  |  | Permanent (all participants) |  |  |  |  |  |  |  |
| Li et al, 2019^340^ | China | Mixed methods study (protocol) | 392 participants to be enrolled | AFEQT [Chinese] | / | HADS [Cantonese] | / | / | / | / |
|  |  |  | Participants aged ≥65 years to be enrolled |  |  |  |  |  |  |  |
|  |  |  | NR |  |  |  |  |  |  |  |
|  |  |  | NR |  |  |  |  |  |  |  |
|  |  |  | NR |  |  |  |  |  |  |  |
| Li et al, 2021^341^ | China | Methodological study | 200 | AFEQT [Chinese] & EQ-5D-5L [Chinese] | / | / | / | / | / | / |
|  |  |  | 69.8±5.2 years |  |  |  |  |  |  |  |
|  |  |  | 48.5% |  |  |  |  |  |  |  |
|  |  |  | NR |  |  |  |  |  |  |  |
|  |  |  | Paroxysmal (44.5%), persistent (2%), permanent (1%) & undifferentiated (52.5%) |  |  |  |  |  |  |  |
| Li et al, 2023^342^ | China | Mixed methods study | 40 | AFEQT [Chinese] | / | HADS [Cantonese] | / | / | / | / |
|  |  |  | 72.10±4.88 years |  |  |  |  |  |  |  |
|  |  |  | 34% |  |  |  |  |  |  |  |
|  |  |  | NR |  |  |  |  |  |  |  |
|  |  |  | NR |  |  |  |  |  |  |  |
| Li et al, 2024^343^ | China | Methodological study | 16 with AF (total n=444) | AFEQT [Chinese] & EQ-5D-5L [Chinese] | / | / | / | / | / | / |
|  |  |  | 65.6 ±11.3 years |  |  |  |  |  |  |  |
|  |  |  | 39.9% |  |  |  |  |  |  |  |
|  |  |  | NR |  |  |  |  |  |  |  |
|  |  |  | NR |  |  |  |  |  |  |  |
| Li et al 2024 [a] ^344^ | China | RCT (protocol) | 146 participants to be enrolled | AFEQT | / | / | / | / | / | / |
|  |  |  | Participants aged ≥18 years to be enrolled |  |  |  |  |  |  |  |
|  |  |  | NR |  |  |  |  |  |  |  |
|  |  |  | NR |  |  |  |  |  |  |  |
|  |  |  | Paroxysmal or persistent AF participants to be enrolled |  |  |  |  |  |  |  |
| Li YM et al, 2019^345^ | China | Cohort study (registry) | 14,723 | AFEQT | / | / | / | / | / | / |
|  |  |  | Women group (n=5,645): 67.5±10.6 years & men group (n=9,078): 62.2±12.2 years |  |  |  |  |  |  |  |
|  |  |  | 38.3% |  |  |  |  |  |  |  |
|  |  |  | NR |  |  |  |  |  |  |  |
|  |  |  | Age <75 (n=11,515) - Female group (n=4,053): newly diagnosed (5.7%), paroxysmal (65.3%) & persistent (29%) & male group (n=7,462): newly diagnosed (3.8%), paroxysmal (55.8%) & persistent (40.3%)  Age ≥75 (n=3,208) - Female (n=1,592): newly diagnosed (9.5%), paroxysmal (48.7%) & persistent (41.9%) & male group (n=1,616): newly diagnosed (9.3%), paroxysmal (46.8%) & persistent (43.8%) |  |  |  |  |  |  |  |
| Lim et al, 2007^346^ | Australia | RCT | 66 | AQoL, CAST-QoL & SIP | / | / | / | / | / | / |
|  |  |  | Pharmacologic therapy group (n=15): 73±6.8 years, atrioventricular junction ablation and pacing group (n=18): 75±8 years & remaining population (n=33): 74±7.5 years |  |  |  |  |  |  |  |
|  |  |  | Pharmacologic therapy group: 28%, atrioventricular junction ablation and pacing group: 20% & remaining population: 24% |  |  |  |  |  |  |  |
|  |  |  | NR |  |  |  |  |  |  |  |
|  |  |  | Permanent (all participants) |  |  |  |  |  |  |  |
| Lin et al, 2020^347^ | Taiwan | Cohort study | 824 | AFEQT [Chinese] | / | / | / | / | / | / |
|  |  |  | 75.3±7.2 years |  |  |  |  |  |  |  |
|  |  |  | 51.7% |  |  |  |  |  |  |  |
|  |  |  | NR |  |  |  |  |  |  |  |
|  |  |  | Paroxysmal (87%), persistent (5%) & missing (8%) |  |  |  |  |  |  |  |
| Lin et al, 2021^348^ | China | Pilot RCT (protocol) | 60 participants to be enrolled | AFEQT | / | / | / | / | / | / |
|  |  |  | Participants aged 18-75 years to be enrolled |  |  |  |  |  |  |  |
|  |  |  | NR |  |  |  |  |  |  |  |
|  |  |  | NR |  |  |  |  |  |  |  |
|  |  |  | Persistent AF participants to be enrolled |  |  |  |  |  |  |  |
| Lin et al, 2022^349^ | China | Cohort study | 185 | AFEQT | / | / | / | / | / | / |
|  |  |  | 70.52±4.51 years |  |  |  |  |  |  |  |
|  |  |  | 47% |  |  |  |  |  |  |  |
|  |  |  | NR |  |  |  |  |  |  |  |
|  |  |  | Persistent or long-lasting persistent (all participants, n or % not specified) |  |  |  |  |  |  |  |
| Liu et al, 2022^350^ | Taiwan | Cohort study | 142 | EQ-5D | BADL & IADL | / | / | / | / | / |
|  |  |  | Median age reported: 66 (IQR range 34-98) years |  |  |  |  |  |  |  |
|  |  |  | 43.66% |  |  |  |  |  |  |  |
|  |  |  | NR |  |  |  |  |  |  |  |
|  |  |  | NR |  |  |  |  |  |  |  |
| Liu et al, 2023^351^ | China | RCT | 97 | EQ-5D-5L [Chinese] | / | GAD-7 & PHQ-9 | / | / | IPAQ | / |
|  |  |  | Digital therapeutics home-based rehabilitation group (n=49): 57±9 years & conventional home-based rehabilitation group (n=48): 57±11 years |  |  |  |  |  |  |  |
|  |  |  | Digital therapeutics home-based rehabilitation group: 36.7% & conventional home-based rehabilitation group: 33.3% |  |  |  |  |  |  |  |
|  |  |  | NR |  |  |  |  |  |  |  |
|  |  |  | Digital therapeutics home-based rehabilitation group: paroxysmal (69.4%) & persistent (30.6%) Conventional home-based rehabilitation group: paroxysmal (56.3%) & persistent (43.7%) |  |  |  |  |  |  |  |
| Linde et al, 2024^352^ | Latvia | Cohort study | 97 | SF-36 | / | / | / | / | / | / |
|  |  |  | 60.06±11.61 years |  |  |  |  |  |  |  |
|  |  |  | 39.2% |  |  |  |  |  |  |  |
|  |  |  | NR |  |  |  |  |  |  |  |
|  |  |  | Paroxysmal (67%) & persistent (33%) |  |  |  |  |  |  |  |
| Lo et al, 2023^353^ | NR | Cohort study | 223 | AFEQT & EQ-5D-5L | / | / | / | / | / | / |
|  |  |  | 65.4±10.1 years |  |  |  |  |  |  |  |
|  |  |  | 30.9% |  |  |  |  |  |  |  |
|  |  |  | NR |  |  |  |  |  |  |  |
|  |  |  | Persistent (all participants) |  |  |  |  |  |  |  |
| Lomper et al, 2019^354^ | Poland | Methodological study | 244 | ASTA HRQoL scale [Polish] | / | / | / | ASTA symptom scale [Polish] | / | / |
|  |  |  | 70.7±10.7 years |  |  |  |  |  |  |  |
|  |  |  | 56.6% |  |  |  |  |  |  |  |
|  |  |  | NR |  |  |  |  |  |  |  |
|  |  |  | Paroxysmal (47.5%) & permanent or persistent (52.5%) |  |  |  |  |  |  |  |
| Lonnerholm et al, 2009^355^ | Sweden | Cohort study | 61 | SF-36 [Swedish] | / | / | / | / | / | / |
|  |  |  | 55±13 years |  |  |  |  |  |  |  |
|  |  |  | 21.3% |  |  |  |  |  |  |  |
|  |  |  | NR |  |  |  |  |  |  |  |
|  |  |  | Paroxysmal or persistent (56%) & permanent (44%) |  |  |  |  |  |  |  |
| Lönnerholm et al, 2000^356^ | Sweden | Cohort study | 49 | SF-36 [Swedish] | / | / | / | / | / | / |
|  |  |  | 52 years |  |  |  |  |  |  |  |
|  |  |  | 20% |  |  |  |  |  |  |  |
|  |  |  | NR |  |  |  |  |  |  |  |
|  |  |  | Paroxysmal (37%), persistent (22%) & permanent (41%) |  |  |  |  |  |  |  |
| Looi et al, 2013^357^ | UK | Cohort study | 203 | AFEQT | / | / | / | / | / | / |
|  |  |  | 57.7±11 years |  |  |  |  |  |  |  |
|  |  |  | 23% |  |  |  |  |  |  |  |
|  |  |  | NR |  |  |  |  |  |  |  |
|  |  |  | Paroxysmal (all participants) |  |  |  |  |  |  |  |
| Lowres et al, 2016^358^ | Australia | Pilot cohort study | 19 | AF-QoL & SF-12 | / | HADS | / | / | GPAQ | / |
|  |  |  | 65±13 years |  |  |  |  |  |  |  |
|  |  |  | 44% |  |  |  |  |  |  |  |
|  |  |  | NR |  |  |  |  |  |  |  |
|  |  |  | NR |  |  |  |  |  |  |  |
| Lu et al, 2020^359^ | China | Cohort study | 436 | / | / | / | / | / | IPAQ-SF [Chinese] | / |
|  |  |  | Age range reported: Low physical activity group (n=150): 65 years (n=104), 65-75 years (n=32) & ≥75 years (n=14) Moderate physical activity group (n=207): 65 years (n=141), 65-75 years (n=45) & ≥75 years (n=21)  High physical activity group (n=79): 65 years (n=54), 65-75 years (n=22) & ≥75 years (n=3 |  |  |  |  |  |  |  |
|  |  |  | Low physical activity group: 36.7% Moderate physical activity group: 30.9% High physical activity group: 34.2% |  |  |  |  |  |  |  |
|  |  |  | NR |  |  |  |  |  |  |  |
|  |  |  | Low physical activity group: paroxysmal (60.7%) & persistent (39.3%)  Moderate physical activity group: paroxysmal (58.9%) & persistent (41.1%) High physical activity group: paroxysmal (58.2%) & persistent (41.8%) |  |  |  |  |  |  |  |
| Lundberg et al, 2008^360^ | Sweden | Cohort study | 34 | SF-36 [Swedish] | / | / | / | / | / | / |
|  |  |  | 57±10 years |  |  |  |  |  |  |  |
|  |  |  | 26% |  |  |  |  |  |  |  |
|  |  |  | NR |  |  |  |  |  |  |  |
|  |  |  | Paroxysmal (26%), persistent (44%) & permanent (29%) |  |  |  |  |  |  |  |
| Lurz et al, 2024^361^ | NR | RCT | 151 | AFEQT & PROMIS global health | / | / | / | AF6 | / | / |
|  |  |  | 61±8.7 years |  |  |  |  |  |  |  |
|  |  |  | 41% |  |  |  |  |  |  |  |
|  |  |  | NR |  |  |  |  |  |  |  |
|  |  |  | Paroxysmal (55%) & persistent (45%) |  |  |  |  |  |  |  |
| MacDonald et al, 2011^362^ | UK | RCT | 41 | SF-36 | / | / | / | / | / | / |
|  |  |  | Medical treatment group (n=19): 64.4±8.3 years & ablation group (n=22): 62.3±6.7 years |  |  |  |  |  |  |  |
|  |  |  | Medical treatment group: 21% & ablation group: 23% |  |  |  |  |  |  |  |
|  |  |  | NR |  |  |  |  |  |  |  |
|  |  |  | Persistent (all participants) |  |  |  |  |  |  |  |
| Magnani et al, 2022^363^ | USA | RCT | 270 | AFEQT & PROMIS-29 | / | PHQ-8 | T-MoCA | / | / | / |
|  |  |  | Median age reported: 73.1 (IQR range 40.8-92.2) years |  |  |  |  |  |  |  |
|  |  |  | 60.4% |  |  |  |  |  |  |  |
|  |  |  | American Indian or Alaska Native (1.5%), Asian (0.4%), Black (2.6%), White (95.2%) & Unknown (1.6%) |  |  |  |  |  |  |  |
|  |  |  | Persistent, paroxysmal & permanent (n or % not specified) |  |  |  |  |  |  |  |
| Magnussen et al, 2018^364^ | Germany | Cross-sectional study | 466 with AF (total n =14,796) | / | / | GAD-7 & PHQ-9 | / | / | / | / |
|  |  |  | Median age reported: Women group (n=142): 66 (IQR range 60-71) years & men group (n=324): 67 (IQR range 61-71) years |  |  |  |  |  |  |  |
|  |  |  | 30.5% |  |  |  |  |  |  |  |
|  |  |  | NR |  |  |  |  |  |  |  |
|  |  |  | NR |  |  |  |  |  |  |  |
| Mahmoud et al, 2013^365^ | Iran | Case-control study | 30 with AF (total n=60) | / | / | / | MMSE | / | / | / |
|  |  |  | 56.60±16.34 years |  |  |  |  |  |  |  |
|  |  |  | NR |  |  |  |  |  |  |  |
|  |  |  | NR |  |  |  |  |  |  |  |
|  |  |  | Persistent (50%) & permanent (50%) |  |  |  |  |  |  |  |
| Malm et al, 2018^366^ | Sweden | RCT | 104 | EQ-5D [Swedish] | / | HADS [Swedish] | / | / | / | / |
|  |  |  | 66.7±9.4 years |  |  |  |  |  |  |  |
|  |  |  | 54.8% |  |  |  |  |  |  |  |
|  |  |  | NR |  |  |  |  |  |  |  |
|  |  |  | Treatment as usual group: first episode (13.5%), paroxysmal (51.4%), persistent (21.6%), permanent (13.5%)  Cognitive behavioural therapy group: first episode (26.8%), paroxysmal (39%), persistent (31.7%), permanent (2.4%) |  |  |  |  |  |  |  |
| Malmborg et al, 2013^367^ | Sweden | RCT | 110 | SF-36 [Swedish] | / | / | / | SCL | / | / |
|  |  |  | Cryoballoon group (n=54): 59±9 years & pulmonary vein ablation catheter group (n=56): 62±7 years |  |  |  |  |  |  |  |
|  |  |  | 24.5% |  |  |  |  |  |  |  |
|  |  |  | NR |  |  |  |  |  |  |  |
|  |  |  | Cryoballoon group: paroxysmal (39%) & persistent (15%)  Pulmonary vein ablation catheter group: paroxysmal (37%) & persistent (19%) |  |  |  |  |  |  |  |
| Malmo et al, 2016^368^ | Norway | RCT | 51 | SF-36 [Norwegian] | / | / | / | SCL [Norwegian] | / | / |
|  |  |  | Control group (n=25): 62±9 years & exercise group: (n=26): 56±8 years |  |  |  |  |  |  |  |
|  |  |  | Control group: 12% & exercise group: 23% |  |  |  |  |  |  |  |
|  |  |  | NR |  |  |  |  |  |  |  |
|  |  |  | Control group: paroxysmal (56%) & persistent (44%) Exervice group: paroxysmal (58%) & persistent (42%) |  |  |  |  |  |  |  |
| Mant et al, 2003^369^ | UK | RCT (protocol) | 1,240 patients to be enrolled | EQ-5D & SF-12 | / | / | MMSE | / | / | / |
|  |  |  | Participants aged ≥75 years to be enrolled |  |  |  |  |  |  |  |
|  |  |  | NR |  |  |  |  |  |  |  |
|  |  |  | NR |  |  |  |  |  |  |  |
|  |  |  | NR |  |  |  |  |  |  |  |
| Mantovan et al, 2013^370^ | NR | RCT | 100 | SF-36 | / | / | / | / | / | / |
|  |  |  | 57±10 years |  |  |  |  |  |  |  |
|  |  |  | 26% |  |  |  |  |  |  |  |
|  |  |  | NR |  |  |  |  |  |  |  |
|  |  |  | Paroxysmal (64%) & persistent (36%) |  |  |  |  |  |  |  |
| Mar et al, 2013^371^ | Spain | Cohort study (protocol) | 320 participants to be enrolled | EQ-5D [Spanish] | BADL | / | / | / | / | / |
|  |  |  | Participants aged >18 years to be enrolled |  |  |  |  |  |  |  |
|  |  |  | NR |  |  |  |  |  |  |  |
|  |  |  | NR |  |  |  |  |  |  |  |
|  |  |  | Non-valvular AF participants to be enrolled |  |  |  |  |  |  |  |
| Marino et al, 2019^372^ | USA | Cohort study | 1,185 | AFEQT | / | GAD-7 & PHQ-9 | MoCA | / | / | / |
|  |  |  | 75.3±7.0 years |  |  |  |  |  |  |  |
|  |  |  | 48% |  |  |  |  |  |  |  |
|  |  |  | Non-Hispanic white (85.5%) |  |  |  |  |  |  |  |
|  |  |  | Paroxysmal (59.6%), persistent (25%) & permanent (5.7%) |  |  |  |  |  |  |  |
| Mark et al, 2019^373^ | 10 countries (not specified) | RCT | 2,204 | AFEQT, EQ-5D-3L & SF-36 | DASI | / | / | AFSS & MAFSI | / | / |
|  |  |  | Median age reported: 68 (IQR range 62-72) years |  |  |  |  |  |  |  |
|  |  |  | 37.2% |  |  |  |  |  |  |  |
|  |  |  | White (91.9%), Black or African American (3.5%) & Other (4.4%) |  |  |  |  |  |  |  |
|  |  |  | Paroxysmal (42.9%), persistent (47.3%) & long-standing persistent (9.8%) |  |  |  |  |  |  |  |
| Marrouche et al, 2009^374^ | NR | RCT (protocol) | 420 participants to be enrolled | EQ-5D | / | / | / | / | / | / |
|  |  |  | Participants aged ≥18years to be enrolled |  |  |  |  |  |  |  |
|  |  |  | NR |  |  |  |  |  |  |  |
|  |  |  | NR |  |  |  |  |  |  |  |
|  |  |  | Paroxysmal or persistent AF participants to be enrolled |  |  |  |  |  |  |  |
| Marrouche et al, 2021^375^ | USA | RCT (protocol) | 330 participants to be enrolled | AFEQT | / | / | / | AFSS | / | / |
|  |  |  | Participants aged ≥18years to be enrolled |  |  |  |  |  |  |  |
|  |  |  | NR |  |  |  |  |  |  |  |
|  |  |  | NR |  |  |  |  |  |  |  |
|  |  |  | Persistent or paroxysmal AF participants to be enrolled |  |  |  |  |  |  |  |
| Marshall et al, 1998^376^ | UK | Cohort study | 18 | MHIQ | / | PGWB | / | / | / | / |
|  |  |  | 63 years |  |  |  |  |  |  |  |
|  |  |  | 33.3% |  |  |  |  |  |  |  |
|  |  |  | NR |  |  |  |  |  |  |  |
|  |  |  | Paroxysmal (all participants) |  |  |  |  |  |  |  |
| Marshall et al, 1999^377^ | UK | RCT | 56 | MHIQ | / | PGWB | / | / | / | / |
|  |  |  | Medical group (n=19): 60.3±9.8 years & ablation group (n=37): 65.2±7.5 years |  |  |  |  |  |  |  |
|  |  |  | Medical group: 36.8% & ablation group: 51.4% |  |  |  |  |  |  |  |
|  |  |  | NR |  |  |  |  |  |  |  |
|  |  |  | Paroxysmal (all participants) |  |  |  |  |  |  |  |
| Marvig et al, 2015^378^ | Greece, Netherlands, Sweden & UK | RCT | 781 with AF (total n=1,003) | EQ-5D & EQ-VAS | / | / | / | / | / | / |
|  |  |  | Completed data for EQ-5D group (n=660): 72±9.7 years & completed data for EQ-VAS group (n=644): 72±9.8 years |  |  |  |  |  |  |  |
|  |  |  | Completed data for EQ-5D group: 37.6% & completed data for EQ-VAS group: 37.7% |  |  |  |  |  |  |  |
|  |  |  | NR |  |  |  |  |  |  |  |
|  |  |  | NR |  |  |  |  |  |  |  |
| Maryniak et al, 2006^379^ | Poland | Cohort study | 76 | SF-36v2 [Polish] | / | / | / | / | / | / |
|  |  |  | 53.2 years |  |  |  |  |  |  |  |
|  |  |  | 28.9% |  |  |  |  |  |  |  |
|  |  |  | NR |  |  |  |  |  |  |  |
|  |  |  | Paroxysmal (all participants) |  |  |  |  |  |  |  |
| Marzona et al, 2012^380^ | Austria, Belgium, Czech Republic, Germany, Greece, Netherlands, Finland, Norway, Sweden, South Africa & South Korea | RCT | 3,068 with AF (total n=31,506) | / | / | / | MMSE | / | / | / |
|  |  |  | AF at baseline group (n=1,016): 70.3±6.9 years & AF at follow-up group (n=2,052): 69.0±7.1 years |  |  |  |  |  |  |  |
|  |  |  | AF at baseline group: 27.3% & AF at follow up group: 25.6% |  |  |  |  |  |  |  |
|  |  |  | NR |  |  |  |  |  |  |  |
|  |  |  | NR |  |  |  |  |  |  |  |
| Masterson Creber et al, 2022^381^ | USA | RCT | 105 | AFEQT | / | / | / | AFSS | / | / |
|  |  |  | 62 years |  |  |  |  |  |  |  |
|  |  |  | 25% |  |  |  |  |  |  |  |
|  |  |  | White (76%), Black or African American (2%), Asian (1%) & unknown (21%) |  |  |  |  |  |  |  |
|  |  |  | Paroxysmal (67%), persistent (30%) & unknown or not reported (3%) |  |  |  |  |  |  |  |
| Mavaddat et al, 2014^382^ | UK | RCT | 973 | / | / | / | MMSE | / | / | / |
|  |  |  | 81.5±4.3 years |  |  |  |  |  |  |  |
|  |  |  | 45.4% |  |  |  |  |  |  |  |
|  |  |  | NR |  |  |  |  |  |  |  |
|  |  |  | NR |  |  |  |  |  |  |  |
| McCabe et al, 2012^383^ | USA | Cross-sectional study | 207 | / | / | POMS | / | SCL | / | / |
|  |  |  | 64.2±12.3 years |  |  |  |  |  |  |  |
|  |  |  | 44% |  |  |  |  |  |  |  |
|  |  |  | NR |  |  |  |  |  |  |  |
|  |  |  | Paroxysmal (64%) & persistent (36%) |  |  |  |  |  |  |  |
| McManus et al, 2019^384^ | USA | Cohort study | 1,064 | AFEQT | / | PHQ-9 | MoCA | / | LTPA | / |
|  |  |  | 75.7±7.1 years |  |  |  |  |  |  |  |
|  |  |  | 49% |  |  |  |  |  |  |  |
|  |  |  | White (85%) & non-White (15%) |  |  |  |  |  |  |  |
|  |  |  | Direct oral anticoagulant group (n=466): paroxysmal (64%), persistent (25.3%), permanent (4.1%) & unknown (6.7%)  Vitamin K antagonist group (n=598): paroxysmal (50.5%), persistent (29.9%), permanent (8.4%) & unknown (11.2%) |  |  |  |  |  |  |  |
| Medi et al, 2013^385^ | Australia | Cohort study | 150 | / | / | / | MMSE | / | / | / |
|  |  |  | Paroxysmal AF group (n=60): 57±9 years, persistent AF group (n=30): 53±10 years, supraventricular tachycardia group (n=30): 56±11 years & control group (n=30): 53±9 years |  |  |  |  |  |  |  |
|  |  |  | Paroxysmal AF group: 25%, persistent AF group: 7%, supraventricular tachycardia group: 57% & control group: 23% |  |  |  |  |  |  |  |
|  |  |  | NR |  |  |  |  |  |  |  |
|  |  |  | Paroxysmal (40%), persistent (20%) & not specified (40%) |  |  |  |  |  |  |  |
| Medin et al, 2014^386^ | France, Germany, Japan, UK & USA | Methodological study | 313 | AFImpact & SF-36 | / | / | / | AFSS & AFSymp | / | / |
|  |  |  | 65.0±11.3 years |  |  |  |  |  |  |  |
|  |  |  | 40% |  |  |  |  |  |  |  |
|  |  |  | Caucasian (88%), African American (6%), Hispanic American (3%), Asian or Oriental or Pacific Islander (1%) & Other (2%) |  |  |  |  |  |  |  |
|  |  |  | Paroxysmal (32.9%), persistent (31.9%) & permanent (35.1%) |  |  |  |  |  |  |  |
| Mehawej et al, 2021^387^ | USA | Cohort study | 807 | AFEQT | / | GAD-7 & PHQ-9 | MoCA | / | / | / |
|  |  |  | Patient engagement yes group (n=494): 74±6 years & patient engagement no group (n=313): 76±7 years |  |  |  |  |  |  |  |
|  |  |  | Patient engagement yes group: 48% & patient engagement no group: 47% |  |  |  |  |  |  |  |
|  |  |  | NR |  |  |  |  |  |  |  |
|  |  |  | Patient engagement yes group: paroxysmal (61%) & persistent or permanent (31%) Patient engagement no group: paroxysmal (54%) & persistent or permanent (34%) |  |  |  |  |  |  |  |
| Mehra et al, 2004^388^ | NR | Cohort study | 643 | SF-36 | / | / | / | SCL | / | / |
|  |  |  | 69.9±11.6 years |  |  |  |  |  |  |  |
|  |  |  | 43% |  |  |  |  |  |  |  |
|  |  |  | NR |  |  |  |  |  |  |  |
|  |  |  | NR |  |  |  |  |  |  |  |
| Mills et al, 2020^389^ | UK | Cohort study | 33 | / | / | GAD-7 & PHQ-9 | / | / | / | / |
|  |  |  | 69.9±1.7 years |  |  |  |  |  |  |  |
|  |  |  | 58% |  |  |  |  |  |  |  |
|  |  |  | NR |  |  |  |  |  |  |  |
|  |  |  | Paroxysmal (45.5%), persistent (48.5%) & permanent (6%) |  |  |  |  |  |  |  |
| Minjie et al, 2023^390^ | China | Pseudo-RCT | 90 | SF-12 | / | GAD-7 & PHQ-9 [Chinese] | / | AFSS | / | / |
|  |  |  | Cognitive behavioural therapy group (n=45): 60.78±7.50 years & treatment as usual group (n=45): 61.67±5.78 years |  |  |  |  |  |  |  |
|  |  |  | Cognitive behavioural therapy group: 33.3% & treatment as usual group: 51.1% |  |  |  |  |  |  |  |
|  |  |  | NR |  |  |  |  |  |  |  |
|  |  |  | Paroxysmal (all participants) |  |  |  |  |  |  |  |
| Minjie et al, 2024^391^ | China | Cross-sectional study | 187 | SF-12 | / | GAD-7 & PHQ-9 [Chinese] | / | AFSS | / | / |
|  |  |  | 66±7 years |  |  |  |  |  |  |  |
|  |  |  | 54.5% |  |  |  |  |  |  |  |
|  |  |  | NR |  |  |  |  |  |  |  |
|  |  |  | Paroxysmal (50.6%), persistent (23%) & permanent (26.4%) |  |  |  |  |  |  |  |
| Mitchell et al, 2003^392^ | UK | Cohort study | 18 | / | / | HADS | / | / | / | / |
|  |  |  | 63.1 years |  |  |  |  |  |  |  |
|  |  |  | 22% |  |  |  |  |  |  |  |
|  |  |  | NR |  |  |  |  |  |  |  |
|  |  |  | Persistent (all participants) |  |  |  |  |  |  |  |
| Miura et al, 2020^393^ | Japan | Cohort study (registry) | 733 | AFEQT | / | / | / | / | / | / |
|  |  |  | Median age reported: rate control group (n=412): 78 (IQR range 74-82 years) & rhythm control group (n=321): 74 (IQR range 72-77.5) years |  |  |  |  |  |  |  |
|  |  |  | Rate control group: 45.4% & rhythm control group: 40.5% |  |  |  |  |  |  |  |
|  |  |  | NR |  |  |  |  |  |  |  |
|  |  |  | Rate control group: paroxysmal (56.6%) & persistent (43.4%) Rhythm control group: paroxysmal (71.7%) & persistent (28.3%) |  |  |  |  |  |  |  |
| Miyama et al, 2022^394^ | Japan | Cohort study (registry) | 2,233 | AFEQT | / | / | / | / | / | / |
|  |  |  | Median age reported: 69 (IQR range 60-76) years |  |  |  |  |  |  |  |
|  |  |  | 31.7% |  |  |  |  |  |  |  |
|  |  |  | NR |  |  |  |  |  |  |  |
|  |  |  | Narrow QRS group (n=2,967): first detected (6.2%), paroxysmal (48.8%), persistent (27.9%) & permanent (15.8%)  Wide QRS group (n=302): first detected (6%), paroxysmal (48.2%), persistent (22.9%) & permanent (20.6%) |  |  |  |  |  |  |  |
| Miyama et al 2024^395^ | Japan | Cohort study (registry) | 1,960 | AFEQT | / | / | / | / | / | / |
|  |  |  | Median age reported: 69 (IQR range 60-76) years |  |  |  |  |  |  |  |
|  |  |  | 32.1% |  |  |  |  |  |  |  |
|  |  |  | NR |  |  |  |  |  |  |  |
|  |  |  | Rate control group (n=804): paroxysmal (56.7%) & persistent (43.3%)  Antiarrhythmic drug group (n=243): paroxysmal (76.1%) & persistent (23.9%)  Catheter ablation group (n=913): paroxysmal (66.4%) & persistent (33.6%) |  |  |  |  |  |  |  |
| Miyamoto et al, 2022^396^ | Japan | RCT (protocol) | 500 participants to be enrolled | AFEQT | / | / | / | / | / | / |
|  |  |  | Participants aged 20-85 years to be enrolled |  |  |  |  |  |  |  |
|  |  |  | NR |  |  |  |  |  |  |  |
|  |  |  | NR |  |  |  |  |  |  |  |
|  |  |  | Persistent AF participants to be enrolled |  |  |  |  |  |  |  |
| Miyazaki et al, 2008^397^ | Japan | Cohort study | 86 | AFQLQ | / | / | / | / | / | / |
|  |  |  | Paroxysmal AF group (n=61): 59.5±8.5 years & chronic AF group (n=25): 58.6±10.2 years |  |  |  |  |  |  |  |
|  |  |  | 20.9% |  |  |  |  |  |  |  |
|  |  |  | NR |  |  |  |  |  |  |  |
|  |  |  | Paroxysmal (70.9%) & chronic (29.1%) |  |  |  |  |  |  |  |
| Mizrahi et al, 2014^398^ | Israel | Case-control study | 180 with AF (total n=919) | / | / | / | MMSE | / | / | / |
|  |  |  | 78.07±7.67 years |  |  |  |  |  |  |  |
|  |  |  | 61.1% |  |  |  |  |  |  |  |
|  |  |  | NR |  |  |  |  |  |  |  |
|  |  |  | Chronic, paroxysmal & persistent (n or % not specified) |  |  |  |  |  |  |  |
| Mohanty et al, 2011^399^ | USA | Cohort study | 660 | SF-36 | / | BDI, HADS & STAI | / | / | / | / |
|  |  |  | 62±10 years |  |  |  |  |  |  |  |
|  |  |  | 31% |  |  |  |  |  |  |  |
|  |  |  | NR |  |  |  |  |  |  |  |
|  |  |  | Paroxysmal (27%), persistent (31%) & long-standing persistent (42%) |  |  |  |  |  |  |  |
| Mohanty et al, 2012^400^ | USA | Cohort study | 1,496 | SF-36 | / | / | / | / | / | / |
|  |  |  | Group 1 with metabolic syndrome (n=485): 64±8 years & group 2 without metabolic syndrome (n=1,011): 62±10 years |  |  |  |  |  |  |  |
|  |  |  | Group 1 with metabolic syndrome: 23% & group 2 without metabolic syndrome: 28% |  |  |  |  |  |  |  |
|  |  |  | NR |  |  |  |  |  |  |  |
|  |  |  | Paroxysmal (29%), persistent (26%) & long-standing persistent (45%) |  |  |  |  |  |  |  |
| Mohanty et al, 2013^401^ | Italy & USA | RCT | 360 | SF-36 | / | BDI, HADS & STAI | / | / | / | / |
|  |  |  | Group 1 (n=182): 61±10 years & group 2 (n=178): 62±9 years |  |  |  |  |  |  |  |
|  |  |  | Group 1: 24% & group 2: 24% |  |  |  |  |  |  |  |
|  |  |  | NR |  |  |  |  |  |  |  |
|  |  |  | Paroxysmal (all participants) |  |  |  |  |  |  |  |
| Mohanty et al, 2018^402^ | USA | Cohort study | 90 | SF-36 | / | / | / | AFSS | / | / |
|  |  |  | Group 1 (n=58): 62±9 years & group 2 (n=32): 64±10 years |  |  |  |  |  |  |  |
|  |  |  | Group 1: 29% & group 2: 25% |  |  |  |  |  |  |  |
|  |  |  | NR |  |  |  |  |  |  |  |
|  |  |  | Long-standing persistent (all participants) |  |  |  |  |  |  |  |
| Mohanty et al, 2015^403^ | USA | Cohort study | 125 | SF-36 | / | / | / | / | / | / |
|  |  |  | 63±11 years |  |  |  |  |  |  |  |
|  |  |  | 26% |  |  |  |  |  |  |  |
|  |  |  | NR |  |  |  |  |  |  |  |
|  |  |  | Paroxysmal (32%), persistent (30%) & long-standing persistent (38%) |  |  |  |  |  |  |  |
| Mohanty et al, 2021^404^ | USA | Cohort study | 98 | AFEQT | / | / | MoCA | / | / | / |
|  |  |  | Left atrial appendage occlusion group (n=50): 69.6±7.8 years & oral anticoagulation group (n=48): 69.1±5.7 years |  |  |  |  |  |  |  |
|  |  |  | Left atrial appendage occlusion group: 26% & oral anticoagulation group: 29.2% |  |  |  |  |  |  |  |
|  |  |  | NR |  |  |  |  |  |  |  |
|  |  |  | Left atrial appendage occlusion group: non-paroxysmal (76%)  Oral anticoagulation group: non-paroxysmal (60.4%) |  |  |  |  |  |  |  |
| Mohanty et al, 2014^405^ | USA | Cohort study | 61 | SF-36 | / | / | / | / | / | / |
|  |  |  | 62±13 years |  |  |  |  |  |  |  |
|  |  |  | 29% |  |  |  |  |  |  |  |
|  |  |  | NR |  |  |  |  |  |  |  |
|  |  |  | Long-standing persistent (all participants) |  |  |  |  |  |  |  |
| Monahan et al, 2022^406^ | NR | RCT | 2,203 | AFEQT | / | / | / | MAFSI | / | / |
|  |  |  | Median age reported: paroxysmal AF – drug group (n=476): 68 (IQR range 63-73) years & ablation group (n=470): 67 (IQR range 62-72) years  Persistent AF – drug group (n=518): 68 (IQR range 62-73) years & ablation group (n=524): 69 (IQR range 63-73) years Long-standing persistent AF – drug group (n=101): 65 (IQR range 61-70) years & ablation group (n=114): 65 (IQR range 60-70) years |  |  |  |  |  |  |  |
|  |  |  | Paroxysmal AF – drug group: 44.5% & ablation group: 41.3%  Persistent AF – drug group: 32% & ablation group: 35.5% Long-standing persistent AF – drug group: 26.7% & ablation group: 28.9% |  |  |  |  |  |  |  |
|  |  |  | Paroxysmal AF – drug group: White (91.6%), Black or African American (3.6%) & Other (4.8%) ablation group: White (92.1%), Black or African American (2.6%) & Other (4.8%)  Persistent AF – drug group: White (92.8%), Black or African American (3.5%) & other (3.7%) ablation group: White (91.8%), Black or African American (4.4%) & Other (3.8%)  Long-standing persistent AF – drug group: White (91.1%), Black or African American (3%) & Other (5.9%)  ablation group: White (92.1%), Black or African American (3.5%) & Other (4.4%) |  |  |  |  |  |  |  |
|  |  |  | Paroxysmal (42.9%), persistent (47.3%) & long-standing persistent (9.8%) |  |  |  |  |  |  |  |
| Montbleau et al, 2017^407^ | USA | Cohort study | 40 | SF-36 [English & Spanish] | / | / | / | / | / | / |
|  |  |  | 78±8.0 years |  |  |  |  |  |  |  |
|  |  |  | 45% |  |  |  |  |  |  |  |
|  |  |  | White (45%), Black (27.5%), Hispanic (22.5%) & Other (5%) |  |  |  |  |  |  |  |
|  |  |  | NR |  |  |  |  |  |  |  |
| Monz et al, 2013^408^ | Canada, USA & countries not specified in Latin America and Asia | RCT | 1,435 | EQ-5D | / | / | / | / | / | / |
|  |  |  | 71.9±8.15 years |  |  |  |  |  |  |  |
|  |  |  | 40.1% |  |  |  |  |  |  |  |
|  |  |  | White (70%) & not specified (30%) |  |  |  |  |  |  |  |
|  |  |  | NR |  |  |  |  |  |  |  |
| Moreira et al, 2016^409^ | Brazil | Methodological study | 40 | QLAF, QLAFv2 & SF-36 [Brazilian Portuguese] | / | / | / | / | / | / |
|  |  |  | 61.2±9.6 years |  |  |  |  |  |  |  |
|  |  |  | 35% |  |  |  |  |  |  |  |
|  |  |  | NR |  |  |  |  |  |  |  |
|  |  |  | Persistent (47.5%) & permanent (52.5%) |  |  |  |  |  |  |  |
| Morillo et al, 2014^410^ | Canada, Czech Republic Germany, Italy & USA | RCT | 127 | EQ-5D | / | / | / | / | / | / |
|  |  |  | Catheter ablation group (n=66): 56.3±9.3 years & antiarrhythmic drug group (n=61): 53.4±11.7 years |  |  |  |  |  |  |  |
|  |  |  | Catheter ablation group: 22.7% & antiarrhythmic drug group: 26.2% |  |  |  |  |  |  |  |
|  |  |  | NR |  |  |  |  |  |  |  |
|  |  |  | Paroxysmal (all participants) |  |  |  |  |  |  |  |
| Mortsell et al, 2019^411^ | Sweden | RCT | 139 | EQ-5D-5L | / | / | / | / | / | / |
|  |  |  | Persistent AF group (n=77): 62.0±9.1 years & paroxysmal AF group (n=62): 61.0±10.0 years |  |  |  |  |  |  |  |
|  |  |  | Persistent AF group: 23.4% & paroxysmal AF group: 30.6% |  |  |  |  |  |  |  |
|  |  |  | NR |  |  |  |  |  |  |  |
|  |  |  | Persistent (55.4%) & paroxysmal (44.6%) |  |  |  |  |  |  |  |
| Mörtsell et al, 2018^412^ | Sweden | RCT | 139 | EQ-5D-5L | / | / | / | / | / | / |
|  |  |  | Single cryoablation group (n=69): 61.9±9.08 years & routine group (n=60): 68.3±10.0 years |  |  |  |  |  |  |  |
|  |  |  | Single cryoablation group: 30.4% & routine group: 22.9% |  |  |  |  |  |  |  |
|  |  |  | NR |  |  |  |  |  |  |  |
|  |  |  | Single cryoablation group: paroxysmal (49.3%) & persistent (50.7%)  Routine group: paroxysmal (40%) & persistent (60%) |  |  |  |  |  |  |  |
| Mszar et al, 2023^413^ | USA | Cohort study (registry) | 58,960 | AFEQT | / | / | / | / | / | / |
|  |  |  | 65.3±10.3 years |  |  |  |  |  |  |  |
|  |  |  | 34.6% |  |  |  |  |  |  |  |
|  |  |  | White (95.5%), Black (3.1%), Asian (1.2%) & Other (0.3%) |  |  |  |  |  |  |  |
|  |  |  | Paroxysmal (56.9%), persistent (40.7%), long-standing persistent (2.3%) & permanent (0.2%) |  |  |  |  |  |  |  |
| Mujović et al, 2021^414^ | Serbia | Cohort study | 110 | SF-36 | / | / | / | / | / | / |
|  |  |  | 57.5±10.6 years |  |  |  |  |  |  |  |
|  |  |  | 22.8% |  |  |  |  |  |  |  |
|  |  |  | NR |  |  |  |  |  |  |  |
|  |  |  | Paroxysmal (60%) & non-paroxysmal (40%) |  |  |  |  |  |  |  |
| Mulder et al, 2013^415^ | Netherlands | RCT | 287 | SF-36 [Dutch] | / | / | / | / | / | / |
|  |  |  | Lenient rate control group (n=138) 69±8 years & strict rate control group (n=149): 68±9 years |  |  |  |  |  |  |  |
|  |  |  | Lenient rate control group: 37% & strict rate control group: 38.9% |  |  |  |  |  |  |  |
|  |  |  | NR |  |  |  |  |  |  |  |
|  |  |  | Permanent (all participants) |  |  |  |  |  |  |  |
| Murray et al, 2000^416^ | USA | Pilot RCT (protocol) | 200 participants to be enrolled | SF-36 | DASI | / | / | / | / | / |
|  |  |  | NR |  |  |  |  |  |  |  |
|  |  |  | NR |  |  |  |  |  |  |  |
|  |  |  | NR |  |  |  |  |  |  |  |
|  |  |  | Persistent or paroxysmal AF participants to be enrolled |  |  |  |  |  |  |  |
| Nagata et al, 2023^417^ | Japan | Cohort study (registry) | 2,963 | / | / | / | MMSE | / | / | / |
|  |  |  | 81.4±4.8 years |  |  |  |  |  |  |  |
|  |  |  | 44.1% |  |  |  |  |  |  |  |
|  |  |  | NR |  |  |  |  |  |  |  |
|  |  |  | Paroxysmal (39.8%), persistent (33.6%) & permanent (26.6%) |  |  |  |  |  |  |  |
| Nair et al, 2022^418^ | Canada | RCT (protocol) | 396 participants to be enrolled | EQ-5D | / | / | / | / | / | / |
|  |  |  | Participants aged ≥18 years to be enrolled |  |  |  |  |  |  |  |
|  |  |  | NR |  |  |  |  |  |  |  |
|  |  |  | NR |  |  |  |  |  |  |  |
|  |  |  | Paroxysmal AF participants to be enrolled |  |  |  |  |  |  |  |
| Nakamaru et al, 2022^419^ | Japan | Cohort study (registry) | 2,070 | AFEQT | / | / | / | / | / | / |
|  |  |  | Early AF group (n=1,644): 68.5±11.1 years & late AF group (n=426): 64.4±10.6 years |  |  |  |  |  |  |  |
|  |  |  | Early AF group: 35% & late AF group: 26.1% |  |  |  |  |  |  |  |
|  |  |  | NR |  |  |  |  |  |  |  |
|  |  |  | Early AF group: first detected (8.5%), paroxysmal (50.7%), persistent (29.1%), permanent (9.9%) & unknown (1.8%) Late AF group: first detected (0%), paroxysmal (51.9%), persistent (28.6%), permanent (18.3%) & unknown (1.2%) |  |  |  |  |  |  |  |
| Nakase et al, 2023^420^ | Japan | Cohort study | 14 with AF (total n=170) | / | / | / | MMSE [Japanese] | / | / | / |
|  |  |  | 81.6±4.1 years |  |  |  |  |  |  |  |
|  |  |  | 64.3% |  |  |  |  |  |  |  |
|  |  |  | NR |  |  |  |  |  |  |  |
|  |  |  | NR |  |  |  |  |  |  |  |
| Nasso et al, 2011^421^ | NR | Cohort study | 104 | SF-36 | / | / | / | / | / | / |
|  |  |  | 63.9±8 years |  |  |  |  |  |  |  |
|  |  |  | 62.5% |  |  |  |  |  |  |  |
|  |  |  | NR |  |  |  |  |  |  |  |
|  |  |  | Paroxysmal & persistent (n or % not specified) |  |  |  |  |  |  |  |
| Natale et al, 2014^422^ | USA | RCT | 283 | SF-36v2 | / | / | / | SCL | / | / |
|  |  |  | Effectiveness cohort (n=122): 58.3±10.93 years & safety cohort (n=161): 58.7±10.83 years |  |  |  |  |  |  |  |
|  |  |  | Effectiveness cohort: 28.7% & safety cohort: 28% |  |  |  |  |  |  |  |
|  |  |  | NR |  |  |  |  |  |  |  |
|  |  |  | Paroxysmal (all participants) |  |  |  |  |  |  |  |
| Natale et al, 2021^423^ | USA | Cohort study | 381 | AFEQT | / | / | / | / | / | / |
|  |  |  | 65.6±8.7 years |  |  |  |  |  |  |  |
|  |  |  | 28.9% |  |  |  |  |  |  |  |
|  |  |  | NR |  |  |  |  |  |  |  |
|  |  |  | Persistent (all participants) |  |  |  |  |  |  |  |
| Natale et al, 1999^424^ | NR | Cohort study | 75 | Q-LES-Q | / | / | / | / | / | / |
|  |  |  | Group 1 (n=29): 68.4±7.0 years, group 2 (n=24): 69.2±10.1 years & group 3 (n=22): 69.8±11.3 years |  |  |  |  |  |  |  |
|  |  |  | 32% |  |  |  |  |  |  |  |
|  |  |  | NR |  |  |  |  |  |  |  |
|  |  |  | Chronic (all participants) |  |  |  |  |  |  |  |
| Nazlı et al, 2016^425^ | Turkey | Cross-sectional study | 135 | SF-36 | / | / | / | / | / | / |
|  |  |  | Median age reported: intermittent AF group (n=52): 63 (IQR range 55-69) years & chronic AF group (n=83): 64 (IQR range 56-72) years |  |  |  |  |  |  |  |
|  |  |  | Intermittent AF group: 44% & chronic AF group: 41% |  |  |  |  |  |  |  |
|  |  |  | NR |  |  |  |  |  |  |  |
|  |  |  | Intermittent (39%) & chronic (61%) |  |  |  |  |  |  |  |
| Nery et al, 2022^426^ | Canada | RCT (protocol) | 502 participants to be enrolled | AFEQT & EQ-5D | / | / | / | / | / | / |
|  |  |  | Participants aged ≥18 years to be enrolled |  |  |  |  |  |  |  |
|  |  |  | NR |  |  |  |  |  |  |  |
|  |  |  | NR |  |  |  |  |  |  |  |
|  |  |  | Persistent AF participants to be enrolled |  |  |  |  |  |  |  |
| Newman et al, 2003^427^ | 45 centres in USA, Canada and Europe (countries not specified) | Cohort study | 267 | SF-36 | / | / | / | SCL | / | / |
|  |  |  | 62±12 years |  |  |  |  |  |  |  |
|  |  |  | 27% |  |  |  |  |  |  |  |
|  |  |  | NR |  |  |  |  |  |  |  |
|  |  |  | Paroxysmal or persistent (n or % not specified) |  |  |  |  |  |  |  |
| Ng et al, 2019^428^ | Malaysia | Cross-sectional study | 208 | SF-12v2 [English, Chinese & Malay] | / | / | / | / | / | / |
|  |  |  | Warfarin group (n=109): 61.3±15.9 years & oral anticoagulant group: 69.1±12.1 years |  |  |  |  |  |  |  |
|  |  |  | Warfarin group: 49.5% & oral anticoagulant group: 47.5% |  |  |  |  |  |  |  |
|  |  |  | Warfarin group: Malay (35.8%), Chinese (57.8%), Indian (4.6%) & Other (1.8%)  Oral anticoagulant group: Malay (37.4%), Chinese (45.5%), Indian (13.1%) & Other (4%) |  |  |  |  |  |  |  |
|  |  |  | NR |  |  |  |  |  |  |  |
| Ng et al, 2020^429^ | Malaysia | Cross-sectional study | 300 | SF-12v2 [English, Chinese & Malay] | / | / | / | / | / | / |
|  |  |  | 59.7±15.6 years |  |  |  |  |  |  |  |
|  |  |  | 42.7% |  |  |  |  |  |  |  |
|  |  |  | Malay (22.3%), Chinese (22.7%), 53% Native (53%) & Other (6%) |  |  |  |  |  |  |  |
|  |  |  | NR |  |  |  |  |  |  |  |
| Nielsen et al, 2017^430^ | NR | RCT | 245 | ASTA HRQoL scale & SF-36 | / | / | / | ASTA symptom scale | / | / |
|  |  |  | 55±9 years |  |  |  |  |  |  |  |
|  |  |  | 40% |  |  |  |  |  |  |  |
|  |  |  | NR |  |  |  |  |  |  |  |
|  |  |  | Paroxysmal (all participants) |  |  |  |  |  |  |  |
| Nørgaard et al, 2018^431^ | Denmark | Mixed methods study | 147 | / | / | SCL-92 [Danish] | MMSE | / | / | / |
|  |  |  | Control group (n=71): 59.5±9.8 years & intervention group (n=76): 59.9±8.1 years |  |  |  |  |  |  |  |
|  |  |  | Control group: 34% & intervention group: 29% |  |  |  |  |  |  |  |
|  |  |  | NR |  |  |  |  |  |  |  |
|  |  |  | Control group: paroxysmal (52%), persistent (13%) & long-standing persistent (35%) Intervention group: paroxysmal (74%), persistent (17%) & long-standing persistent (9%) |  |  |  |  |  |  |  |
| Noubiap et al, 2024^432^ | Australia | Cohort study | 355 | / | / | / | / | AFSS | / | / |
|  |  |  | Male group (n=234): 62.5±10.6 years & female group (n=121): 65.5±10.4 years |  |  |  |  |  |  |  |
|  |  |  | 34.1% |  |  |  |  |  |  |  |
|  |  |  | NR |  |  |  |  |  |  |  |
|  |  |  | Paroxysmal (54.9%) & non-paroxysmal (45.1%) |  |  |  |  |  |  |  |
| Nourmohammadi et al, 2019^433^ | Iran | RCT | 50 | SF-36 [Persian] | / | / | / | / | / | / |
|  |  |  | Experimental group (n=25): 57.2±7.4 years & control group (n=25): 59.9±7.5 years |  |  |  |  |  |  |  |
|  |  |  | Experimental group: 60% & control group: 48% |  |  |  |  |  |  |  |
|  |  |  | NR |  |  |  |  |  |  |  |
|  |  |  | Chronic (all participants) |  |  |  |  |  |  |  |
| O’Connell et al, 1998^434^ | UK | Case-control study | 27 with AF (total n=81) | / | / | / | MMSE | / | / | / |
|  |  |  | AF group (n=27):71.96±1.45 years & control group (n=27): 72±1.02 years |  |  |  |  |  |  |  |
|  |  |  | 40.7% |  |  |  |  |  |  |  |
|  |  |  | NR |  |  |  |  |  |  |  |
|  |  |  | Non-valvular (all participants) |  |  |  |  |  |  |  |
| O’Neill et al, 2023^435^ | NR | Cohort study | 60 | SF-36 | / | / | / | / | / | / |
|  |  |  | 66±9 years |  |  |  |  |  |  |  |
|  |  |  | 30% |  |  |  |  |  |  |  |
|  |  |  | NR |  |  |  |  |  |  |  |
|  |  |  | Persistent (all participants) |  |  |  |  |  |  |  |
| Okumura et al, 2023^436^ | Japan | Cohort study (protocol) | 660 participants to be enrolled | EQ-5D-5L | / | / | MMSE | / | / | / |
|  |  |  | Participants aged ≥80 years to be enrolled |  |  |  |  |  |  |  |
|  |  |  | NR |  |  |  |  |  |  |  |
|  |  |  | NR |  |  |  |  |  |  |  |
|  |  |  | Non-valvular AF participants to be enrolled |  |  |  |  |  |  |  |
| Ogawa et al, 2009^437^ | Japan | RCT | 823 | AFQLQ | / | / | / | / | / | / |
|  |  |  | 64.7±11.3 years |  |  |  |  |  |  |  |
|  |  |  | 30.7% |  |  |  |  |  |  |  |
|  |  |  | NR |  |  |  |  |  |  |  |
|  |  |  | Paroxysmal (all participants) |  |  |  |  |  |  |  |
| Ong et al, 2006^438^ | Canada | Cross-sectional study | 93 | SF-36 | / | ASI & HADS | / | AFSS | / | / |
|  |  |  | 61.88±12.04 years |  |  |  |  |  |  |  |
|  |  |  | 34% |  |  |  |  |  |  |  |
|  |  |  | NR |  |  |  |  |  |  |  |
|  |  |  | Paroxysmal (59%) & permanent or persistent (41%) |  |  |  |  |  |  |  |
| Ong et al, 2006 [a] ^439^ | Canada | Cross-sectional study | 93 | SF-36 | / | HADS | / | AFSS | / | / |
|  |  |  | 61.88±12.04 years |  |  |  |  |  |  |  |
|  |  |  | 34% |  |  |  |  |  |  |  |
|  |  |  | NR |  |  |  |  |  |  |  |
|  |  |  | Female group (n=32): paroxysmal (71.88%) & permanent or persistent (28.12%)  Male group (n=61): paroxysmal (52.46%) & permanent or persistent (47.54%) |  |  |  |  |  |  |  |
| Oser et al, 2019^440^ | USA | RCT | 8 | AFEQT | / | ASI | / | / | / | / |
|  |  |  | 61.50±13.02 years |  |  |  |  |  |  |  |
|  |  |  | 50% |  |  |  |  |  |  |  |
|  |  |  | White (87.5%) & Latino (12.5%) |  |  |  |  |  |  |  |
|  |  |  | Persistent (all participants) |  |  |  |  |  |  |  |
| Osbak et al, 2012^441^ | Denmark | RCT | 47 | SF-36 | / | / | / | / | / | / |
|  |  |  | Training group (n=24): 69.5±7.3 years & control group (n=23): 70.9±8.3 years |  |  |  |  |  |  |  |
|  |  |  | Training group: 25% & control group: 26% |  |  |  |  |  |  |  |
|  |  |  | NR |  |  |  |  |  |  |  |
|  |  |  | Permanent (all participants) |  |  |  |  |  |  |  |
| Osmancik et al, 2020^442^ | NR | Cohort study | 75 | AFEQT & EQ-5D | / | / | / | / | / | / |
|  |  |  | 62.9±8.45 years |  |  |  |  |  |  |  |
|  |  |  | 34.7% |  |  |  |  |  |  |  |
|  |  |  | NR |  |  |  |  |  |  |  |
|  |  |  | Persistent (36%) & long-standing persistent (64%) |  |  |  |  |  |  |  |
| Osmancik et al, 2022^443^ | Czech Republic | RCT (protocol) | 212 participants to be enrolled | AFEQT | / | / | / | / | / | / |
|  |  |  | Participants aged <75 years to be enrolled |  |  |  |  |  |  |  |
|  |  |  | NR |  |  |  |  |  |  |  |
|  |  |  | NR |  |  |  |  |  |  |  |
|  |  |  | Paroxysmal, persistent or long-standing persistent AF participants to be enrolled |  |  |  |  |  |  |  |
| Ozel et al, 2025^444^ | Turkey | Cross-sectional study | 45 | AFImpact (Turkish) |  |  |  |  |  |  |
|  |  |  | 66.6±9.82 years |  |  |  |  |  |  |  |
|  |  |  | 51.1% |  |  |  |  |  |  |  |
|  |  |  | NR |  |  |  |  |  |  |  |
|  |  |  | Paroxysmal (20%), persistent (20%) & permanent (60%) |  |  |  |  |  |  |  |
| Özin et al, 2018^445^ | Turkey | Cohort study | 210 | EQ-5D | / | / | / | / | / | / |
|  |  |  | 64.86±12.87 years |  |  |  |  |  |  |  |
|  |  |  | 42.9% |  |  |  |  |  |  |  |
|  |  |  | NR |  |  |  |  |  |  |  |
|  |  |  | Paroxysmal (32.9%), persistent (23.3%) & permanent (43.8%) |  |  |  |  |  |  |  |
| Packer et al, 2018^446^ | Australia, Canada, China, Czech Republic, Germany, Italy, Russia, South Korea, UK & USA | RCT (protocol) | 2,200 participants to be enrolled | AFEQT, EQ-5D-3L & SF-36 | DASI | / | / | AFSS & MAFSI | / | SPS & WPAI |
|  |  |  | Participants aged ≥18 years to be enrolled |  |  |  |  |  |  |  |
|  |  |  | NR |  |  |  |  |  |  |  |
|  |  |  | NR |  |  |  |  |  |  |  |
|  |  |  | Paroxysmal, persistent or long-standing persistent AF participants to be enrolled |  |  |  |  |  |  |  |
| Packer et al, 2021^447^ | Australia, Canada, China, Czech Republic, Germany, Italy, Russia, South Korea, UK & USA | RCT | 778 | AFEQT | / | / | / | MAFSI | / | / |
|  |  |  | Median age reported: 68 (IQR range 62-73) years |  |  |  |  |  |  |  |
|  |  |  | 44.3% |  |  |  |  |  |  |  |
|  |  |  | Hispanic or non-White (7.8%) |  |  |  |  |  |  |  |
|  |  |  | Paroxysmal (31.6%), persistent (55.3%) & long-standing persistent (13.1%) |  |  |  |  |  |  |  |
| Pappone et al, 2003^448^ | Italy | Cohort study | 1,171 | SF-36 | / | / | / | / | / | / |
|  |  |  | Ablation group (n=589): 65±9 years & medical group (n=582): 65±10 years |  |  |  |  |  |  |  |
|  |  |  | Ablation group: 42% & medical group: 41% |  |  |  |  |  |  |  |
|  |  |  | NR |  |  |  |  |  |  |  |
|  |  |  | Ablation group: paroxysmal (69%) & chronic (31%) Medical group: paroxysmal (71%) & chronic (29%). |  |  |  |  |  |  |  |
| Pappone et al, 2011^449^ | Italy | RCT | 198 | SF-36 | / | / | / | / | / | / |
|  |  |  | 56±10 years |  |  |  |  |  |  |  |
|  |  |  | Total gender frequencies not reported |  |  |  |  |  |  |  |
|  |  |  | NR |  |  |  |  |  |  |  |
|  |  |  | Paroxysmal (all participants) |  |  |  |  |  |  |  |
| Parimbelli et al, 2015^450^ | Italy | Methodological study | 20 | AFEQT & EQ-5D | / | / | / | / | / | / |
|  |  |  | 66.2 years |  |  |  |  |  |  |  |
|  |  |  | 50% |  |  |  |  |  |  |  |
|  |  |  | Caucasian (95%) & North African (5%) |  |  |  |  |  |  |  |
|  |  |  | Paroxysmal (20%), persistent (50%), permanent (25%) & AF type not specified (5%) |  |  |  |  |  |  |  |
| Paquette et al, 2000^451^ | Canada | RCT | 170 | SF-36 | DASI |  |  | AFSS & SCL |  |  |
|  |  |  | Women group (n=62): 68±9 years & men group (n=108): 62±11 years |  |  |  |  |  |  |  |
|  |  |  | 36.5% |  |  |  |  |  |  |  |
|  |  |  | NR |  |  |  |  |  |  |  |
|  |  |  | Women group: paroxysmal (40%) & men group: paroxysmal (44%) |  |  |  |  |  |  |  |
| Park et al, 2007^452^ | UK | Cohort study | 160 | SF-36 | / | / | MMSE | / | / | / |
|  |  |  | 75.6 years |  |  |  |  |  |  |  |
|  |  |  | Case group (n=74): 57% & control group (n=86): 39% |  |  |  |  |  |  |  |
|  |  |  | NR |  |  |  |  |  |  |  |
|  |  |  | Non-valvular (all participants) |  |  |  |  |  |  |  |
| Park et al, 2019^453^ | South Korea | Cohort study | 1,829 | EQ-5D | / | / | / | / | / | / |
|  |  |  | 59±11 years |  |  |  |  |  |  |  |
|  |  |  | 27.6% |  |  |  |  |  |  |  |
|  |  |  | NR |  |  |  |  |  |  |  |
|  |  |  | Paroxysmal (72.8%) |  |  |  |  |  |  |  |
| Parkash et al, 2022^454^ | Brazil, Canada, Sweden & Taiwan | RCT | 411 | AFEQT & EQ-5D-3L | / | / | / | / | / | / |
|  |  |  | Rate control group (n=197): 67.5±8.0 years & ablation-based rhythm control group (n=214): 65.9±8.6 years |  |  |  |  |  |  |  |
|  |  |  | Rate control group: 24.9% & ablation-based rhythm control group: 26.6% |  |  |  |  |  |  |  |
|  |  |  | Rate control group: Asian (1.5%), Black (0%), White (98%) & Other (0.5%)  Ablation-based rhythm control group: Asian (2.8%), Black (0.9%), White (95.3%) & Other (0.9%) |  |  |  |  |  |  |  |
|  |  |  | Rate control group: high-burden paroxysmal (5.6%), persistent type 1 (4.6%), persistent type 2 (65.5%) & long-lasting persistent (24.4%)  Ablation-based rhythm control group: high-burden paroxysmal (8.9%), persistent type 1 (3.3%), persistent type 2 (65.4%) & long-lasting persistent (22.4% |  |  |  |  |  |  |  |
| Parks et al, 2021^455^ | USA | Cohort study | 3,530 | / | ADL & IADL | / | / | / | / | / |
|  |  |  | Median age reported: 79 years (IQR range 72-85) years |  |  |  |  |  |  |  |
|  |  |  | 53% |  |  |  |  |  |  |  |
|  |  |  | White (85%), Black (9%) & Other (2%) |  |  |  |  |  |  |  |
|  |  |  | NR |  |  |  |  |  |  |  |
| Patel et al, 2014^456^ | USA | Cohort study | 286 | / | / | / | / | AFSS | / | / |
|  |  |  | 61.8±13.3 years |  |  |  |  |  |  |  |
|  |  |  | 35% |  |  |  |  |  |  |  |
|  |  |  | NR |  |  |  |  |  |  |  |
|  |  |  | Paroxysmal (59.6%) & persistent (40.4%) |  |  |  |  |  |  |  |
| Pavelková et al, 2014^457^ | Czech Republic | Cohort study | 264 | SF-36 | / | / | / | / | / | / |
|  |  |  | 63.6±9.6 years |  |  |  |  |  |  |  |
|  |  |  | 40.2% |  |  |  |  |  |  |  |
|  |  |  | NR |  |  |  |  |  |  |  |
|  |  |  | Paroxysmal (52.7%) & persistent (47.4%) |  |  |  |  |  |  |  |
| Pavlovic et al, 2021^458^ | Australia, Argentina & European countries (not specified) | RCT | 218 | AFEQT & SF-36v2 | / | / | / | / | / | / |
|  |  |  | Cryoballoon ablation group (n=107): 50.5±13.1 years & antiarrhythmic drug group (n=111): 54.1±13.4 years |  |  |  |  |  |  |  |
|  |  |  | Cryoballoon ablation group: 29% & antiarrhythmic drug group: 35.1% |  |  |  |  |  |  |  |
|  |  |  | NR |  |  |  |  |  |  |  |
|  |  |  | Paroxysmal (all participants) |  |  |  |  |  |  |  |
| Peinado et al, 2010^459^ | Spain | Cohort study | 341 | AF-QoL & SF-36 | / | / | / | / | / | / |
|  |  |  | Paroxysmal AF group (n=126): 57.3±12.5 years, persistent AF group (n=144): 62.2±11.5 years & permanent AF group (n=66): 63.6±12.8 years |  |  |  |  |  |  |  |
|  |  |  | Paroxysmal AF group: 38.1%, persistent AF group: 29.9% & permanent AF group: 37.9% |  |  |  |  |  |  |  |
|  |  |  | NR |  |  |  |  |  |  |  |
|  |  |  | Paroxysmal (37.5%) persistent (42.9%), & permanent (19.6%) |  |  |  |  |  |  |  |
| Peng et al, 2023^460^ | China | Cohort study | 31 | AFEQT | / | / | / | / | / | / |
|  |  |  | 74.6±7.1 years |  |  |  |  |  |  |  |
|  |  |  | 71% |  |  |  |  |  |  |  |
|  |  |  | NR |  |  |  |  |  |  |  |
|  |  |  | Persistent (all participants) |  |  |  |  |  |  |  |
| Perret-Guillaume et al, 2010^461^ | France | Cross-sectional study | 41 with AF (total n=164) | DUKE [French] & SF-36 [French] | / | / | / | / | / | / |
|  |  |  | 72.3±3.9 years |  |  |  |  |  |  |  |
|  |  |  | 34.1% |  |  |  |  |  |  |  |
|  |  |  | NR |  |  |  |  |  |  |  |
|  |  |  | First discovered (9.8%), paroxysmal (17.1%) & permanent (73.2%) |  |  |  |  |  |  |  |
| Pezawas et al, 2016^462^ | Austria | Cohort study | 229 | SF-12v2 | / | / | / | / | / | / |
|  |  |  | 58.1±11 years |  |  |  |  |  |  |  |
|  |  |  | 27% |  |  |  |  |  |  |  |
|  |  |  | NR |  |  |  |  |  |  |  |
|  |  |  | Paroxysmal (48%), persistent (37%) & long-standing persistent (15%) |  |  |  |  |  |  |  |
| Piccini et al, 2011^463^ | USA | Cohort study (registry protocol) | 1,500 participants to be enrolled for this sub study | AFEQT | / | / | / | / | / | / |
|  |  |  | Participants aged ≥18 years to be enrolled |  |  |  |  |  |  |  |
|  |  |  | NR |  |  |  |  |  |  |  |
|  |  |  | NR |  |  |  |  |  |  |  |
|  |  |  | Paroxysmal, persistent or permanent AF participants to be enrolled |  |  |  |  |  |  |  |
| Piccini et al, 2016^464^ | USA | Cohort study (registry) | 10,135 | AFEQT | / | / | / | / | / | / |
|  |  |  | Median age reported: women group (n=4,293): 77 (IQR range 69-83) years & men group (n=5,842): 73 (IQR range 65-80) years |  |  |  |  |  |  |  |
|  |  |  | 42% |  |  |  |  |  |  |  |
|  |  |  | Women group: White (88.1%), Black (5.6%), Hispanic (4.7%) & Other (1.4%)  Men group: White (90%), Black (4.6%), Hispanic (3.8%) & Other (1.4%) |  |  |  |  |  |  |  |
|  |  |  | Women group: first detected (4.8%), paroxysmal (53.7%), persistent (16.7%) & permanent (25.8%) Men group: first detected (4.7%), paroxysmal (48.2%), persistent (17.7%) & permanent (29.5%) |  |  |  |  |  |  |  |
| Piccini et al, 2020^465^ | 8 European countries (not specified) & USA | RCT | 518 | EQ-5D & SF-12 | KPS | / | MoCA | / | / | / |
|  |  |  | Median age reported: 64 (IQR range 58-70) years |  |  |  |  |  |  |  |
|  |  |  | 33.6% |  |  |  |  |  |  |  |
|  |  |  | NR |  |  |  |  |  |  |  |
|  |  |  | Paroxysmal (57.7%) & persistent or long-standing persistent (42.3%) |  |  |  |  |  |  |  |
| Pierre-Louis et al, 2023^466^ | USA | Cohort study | 1,244 | AFEQT | / | / | / | / | / | / |
|  |  |  | 75.5±7.1 years |  |  |  |  |  |  |  |
|  |  |  | 49% |  |  |  |  |  |  |  |
|  |  |  | Non-Hispanic White (87%) & not specified (13%) |  |  |  |  |  |  |  |
|  |  |  | Paroxysmal (59%), persistent or long-standing persistent (25%), permanent (6%) & unknown (10%) |  |  |  |  |  |  |  |
| Pilotto et al, 2016^467^ | Italy | Cohort study | 1,827 | / | BADL | / | SPMSQ | / | / | / |
|  |  |  | 84.4±7.1 years |  |  |  |  |  |  |  |
|  |  |  | 64.3% |  |  |  |  |  |  |  |
|  |  |  | NR |  |  |  |  |  |  |  |
|  |  |  | NR |  |  |  |  |  |  |  |
| Pilotto et al, 2022^468^ | Austria, Belgium, Czech Republic,  Finland, France, Germany, Italy, Netherlands, Poland, Portugal,  Slovakia & Spain | Cohort study | 2,012 | / | ADL & IADL | / | SPMSQ | / | / | / |
|  |  |  | 83.3±7.5 years |  |  |  |  |  |  |  |
|  |  |  | 57% |  |  |  |  |  |  |  |
|  |  |  | NR |  |  |  |  |  |  |  |
|  |  |  | Non-valvular (all participants) |  |  |  |  |  |  |  |
| Plisiene et al, 2008^469^ | Germany | Pilot cohort study | 10 | SF-36 | / | HAM-D | / | / | / | / |
|  |  |  | 59±10 years |  |  |  |  |  |  |  |
|  |  |  | 30% |  |  |  |  |  |  |  |
|  |  |  | NR |  |  |  |  |  |  |  |
|  |  |  | Permanent (all participants) |  |  |  |  |  |  |  |
| Poggesi et al, 2019^470^ | Italy | Cohort study | 194 | EQ-5D | ADL & IADL | GDS | MoCA | / | / | / |
|  |  |  | 78.1±6.7 years |  |  |  |  |  |  |  |
|  |  |  | 39% |  |  |  |  |  |  |  |
|  |  |  | NR |  |  |  |  |  |  |  |
|  |  |  | Paroxysmal (48%) & persistent or permanent (52%) |  |  |  |  |  |  |  |
| Pokorney et al, 2017^471^ | USA | Cohort study (registry) | 9,642 | AFEQT | / | / | / | / | / | / |
|  |  |  | Non-cardioverted group (n=8,825): 74±11 years & cardioverted group (n=817): 69±11 years |  |  |  |  |  |  |  |
|  |  |  | Non-cardioverted group: 43% & cardioverted group: 39% |  |  |  |  |  |  |  |
|  |  |  | Non-cardioverted group: White (89%) & not specified (11%) Cardioverted group: White (94%) & not specified (6%) |  |  |  |  |  |  |  |
|  |  |  | Paroxysmal (all participants) |  |  |  |  |  |  |  |
| Pokorney et al, 2025^472^ | USA | RCT (protocol) | 3,000 participants to be enrolled | AFEQT | / | / | / | MAFSI | / | / |
|  |  |  | Participants aged ≥21 years to be enrolled |  |  |  |  |  |  |  |
|  |  |  | NR |  |  |  |  |  |  |  |
|  |  |  | NR |  |  |  |  |  |  |  |
|  |  |  | Paroxysmal or persistent AF participants to be enrolled |  |  |  |  |  |  |  |
| Pontoppidan et al, 2009^473^ | Denmark | Cohort study | 149 | SF-36 | / | / | / | / | / | / |
|  |  |  | 56±8 years |  |  |  |  |  |  |  |
|  |  |  | 29% |  |  |  |  |  |  |  |
|  |  |  | NR |  |  |  |  |  |  |  |
|  |  |  | Paroxysmal (53.7%) & persistent (46.3%) |  |  |  |  |  |  |  |
| Potpara et al, 2020^474^ | Serbia | Cohort study | 331 with AF (total n=514) | EQ-5D | / | / | / | / | / | / |
|  |  |  | 65.4±10.32 years |  |  |  |  |  |  |  |
|  |  |  | 38.4% |  |  |  |  |  |  |  |
|  |  |  | NR |  |  |  |  |  |  |  |
|  |  |  | Permanent (29.3%) |  |  |  |  |  |  |  |
| Potter et al, 2019^475^ | Australia, Canada & European countries (not specified) | Cohort study | 292 | AFEQT | / | / | / | / | / | / |
|  |  |  | Safety cohort (n=150): 61.6±9.4 years & evaluable cohort (n=142): 61.6±9.6 years |  |  |  |  |  |  |  |
|  |  |  | Safety cohort: 24% & evaluable cohort: 24.6% |  |  |  |  |  |  |  |
|  |  |  | Safety cohort: Black or African American (0.7%), White or Caucasian (90.7%) & not reported (8.7%)  Evaluable cohort: Black or African American (0.7%), White or Caucasian (90.8%) & not reported (8.5%) |  |  |  |  |  |  |  |
|  |  |  | Persistent (all participants) |  |  |  |  |  |  |  |
| Prabhu et al, 2017^476^ | Australia | RCT | 66 | SF-36 |  |  |  |  |  |  |
|  |  |  | Catheter ablation group (n=33): 59±11 years & medical rate control group (n=33): 62±9.4 years |  |  |  |  |  |  |  |
|  |  |  | Catheter ablation group: 6% & medical rate control group: 12% |  |  |  |  |  |  |  |
|  |  |  | NR |  |  |  |  |  |  |  |
|  |  |  | Persistent (all participants) |  |  |  |  |  |  |  |
| Praus et al, 2021^477^ | USA | Cohort study | 43 | / | / | HADS | / | / | / | / |
|  |  |  | 66.79±9.78 years |  |  |  |  |  |  |  |
|  |  |  | 47% |  |  |  |  |  |  |  |
|  |  |  | Caucasian (77%), African American (10%), Hispanic (8%) & Asian (5%) |  |  |  |  |  |  |  |
|  |  |  | NR |  |  |  |  |  |  |  |
| Proietti et al, 2022^478^ | 27 countries (not specified) | Cohort (registry) | 3,774 | EQ-5D-5L | / | / | / | / | / | / |
|  |  |  | 69.8±10.4 years |  |  |  |  |  |  |  |
|  |  |  | 46.3% |  |  |  |  |  |  |  |
|  |  |  | NR |  |  |  |  |  |  |  |
|  |  |  | First detected (27.3%), paroxysmal (40.8%) & persistent (31.9%) |  |  |  |  |  |  |  |
| Punjani et al, 2011^479^ | USA | Cohort study | 50 | VR-36 | / | / | / | / | / | / |
|  |  |  | 0% |  |  |  |  |  |  |  |
|  |  |  | 73.1±8.8 years |  |  |  |  |  |  |  |
|  |  |  | NR |  |  |  |  |  |  |  |
|  |  |  | Persistent or permanent (n or % not specified) |  |  |  |  |  |  |  |
| Purerfellner et al, 2004^480^ | NR | Cohort study | 89 | SF-36 | / | / | / | SCL | / | / |
|  |  |  | 52.6±10.8 years |  |  |  |  |  |  |  |
|  |  |  | 17% |  |  |  |  |  |  |  |
|  |  |  | NR |  |  |  |  |  |  |  |
|  |  |  | Paroxysmal (all participants) |  |  |  |  |  |  |  |
| Qing et al, 2023^481^ | China | Cross-sectional study | 3,562 | SF-36 [Chinese] | / | / | / | / | / | / |
|  |  |  | 69±11.2 years |  |  |  |  |  |  |  |
|  |  |  | 43.3% |  |  |  |  |  |  |  |
|  |  |  | NR |  |  |  |  |  |  |  |
|  |  |  | New onset (13.2%), paroxysmal (35.5%), persistent (30.2%) & permanent (21.2%) |  |  |  |  |  |  |  |
| Raatikainen et al, 2015^482^ | Denmark, Finland, Germany & Sweden | RCT | 286 | SF-36 | / | / | / | / | / | / |
|  |  |  | Radiofrequency ablation group (n=110): 56±10 years, antiarrhythmic drug group (n=92): 56±10 years & crossover group (n=84): 54±9 years |  |  |  |  |  |  |  |
|  |  |  | Radiofrequency ablation group: 29%, antiarrhythmic drug group: 32% & crossover group: 30% |  |  |  |  |  |  |  |
|  |  |  | NR |  |  |  |  |  |  |  |
|  |  |  | Paroxysmal (all participants) |  |  |  |  |  |  |  |
| Raine et al, 2015^483^ | UK | Cohort study | 80 | AFEQT & SF-36v2 | / | / | / | / | / | / |
|  |  |  | 57±10 years |  |  |  |  |  |  |  |
|  |  |  | 27% |  |  |  |  |  |  |  |
|  |  |  | NR |  |  |  |  |  |  |  |
|  |  |  | Paroxysmal (55%) & persistent (45%) |  |  |  |  |  |  |  |
| Raileanu et al, 2024^484^ | Netherlands | Cohort study | 295 | AFEQT | / | HADS | / | / | / | / |
|  |  |  | 64.9±8.1 years |  |  |  |  |  |  |  |
|  |  |  | 34.2% |  |  |  |  |  |  |  |
|  |  |  | NR |  |  |  |  |  |  |  |
|  |  |  | Paroxysmal (65.1%) & persistent (34.9%) |  |  |  |  |  |  |  |
| Ramos Tuarez et al, 2019^485^ | USA | Cohort study | 151 | SF-12v2 | / | / | / | / | / | / |
|  |  |  | 80±7.7 years |  |  |  |  |  |  |  |
|  |  |  | 32% |  |  |  |  |  |  |  |
|  |  |  | NR |  |  |  |  |  |  |  |
|  |  |  | Non-valvular (all participants) |  |  |  |  |  |  |  |
| Randolph et al, 2016^486^ | USA | Cohort study (registry) | 2,007 | AFEQT | / | / | / | / | / | / |
|  |  |  | Median age reported: 76 (IQR range 67-82) years |  |  |  |  |  |  |  |
|  |  |  | 43.2% |  |  |  |  |  |  |  |
|  |  |  | White (89.9%), Black (5.8%), Hispanic (2.6%) & Other (1.4%) |  |  |  |  |  |  |  |
|  |  |  | First detected (9.8%), paroxysmal (47.6%), persistent (15%) & permanent (27.7%) |  |  |  |  |  |  |  |
| Reddy et al, 2024^487^ | USA | RCT | 593 | AFEQT | / | / | / | / | / | / |
|  |  |  | 62.4±8.6 years |  |  |  |  |  |  |  |
|  |  |  | 34.4% |  |  |  |  |  |  |  |
|  |  |  | NR |  |  |  |  |  |  |  |
|  |  |  | Paroxysmal (all participants) |  |  |  |  |  |  |  |
| Reed et al, 2018^488^ | Canada | Case-control study | 47 with AF (total n=94) | SF-36 | / | HADS | / | / | / | / |
|  |  |  | 69.9±7.7 years |  |  |  |  |  |  |  |
|  |  |  | 23.4% |  |  |  |  |  |  |  |
|  |  |  | White (85.1%) |  |  |  |  |  |  |  |
|  |  |  | Persistent (42.6%) & permanent (57.4%) |  |  |  |  |  |  |  |
| Reynolds et al, 2006^489^ | Canada & USA | Cohort study (registry) | 963 | SF-12 | / | / | / | AFSS & SCL | / | / |
|  |  |  | Women group (n=382): 69±13 years & men group (n=581): 64±15 years |  |  |  |  |  |  |  |
|  |  |  | 39.7% |  |  |  |  |  |  |  |
|  |  |  | NR |  |  |  |  |  |  |  |
|  |  |  | Permanent or persistent (n or % not specified) |  |  |  |  |  |  |  |
| Reynolds et al, 2010^490^ | Canada & USA | Cohort study (registry) | 933 | SF-12 | / | / | / | SCL | / | / |
|  |  |  | 66±14 years |  |  |  |  |  |  |  |
|  |  |  | 40% |  |  |  |  |  |  |  |
|  |  |  | White (93%) & Non-White (7%) |  |  |  |  |  |  |  |
|  |  |  | Permanent or persistent (n or % not specified) |  |  |  |  |  |  |  |
| Reynolds et al, 2010 [a] ^491^ | 19 global centres (countries not specified) | RCT | 159 | SF-36 | / | / | / | SCL | / | / |
|  |  |  | Catheter ablation group (n=103): 55.5±9.4 years & antiarrhythmic drug group (n=56): 55.8±13.1 years |  |  |  |  |  |  |  |
|  |  |  | Catheter ablation group: 31% & antiarrhythmic drug group: 37% |  |  |  |  |  |  |  |
|  |  |  | NR |  |  |  |  |  |  |  |
|  |  |  | Paroxysmal (all participants) |  |  |  |  |  |  |  |
| Ricci et al, 2004^492^ | Italy & Spain | Cohort study | 40 | SF-36 | / | / | / | SCL | / | / |
|  |  |  | 64±10 years |  |  |  |  |  |  |  |
|  |  |  | 30% |  |  |  |  |  |  |  |
|  |  |  | NR |  |  |  |  |  |  |  |
|  |  |  | Paroxysmal (40%) & persistent (60%) |  |  |  |  |  |  |  |
| Richard et al, 2022^493^ | Ireland | Case-control study | 49 with AF (total n=169) | / | / | CES-D | MMSE | / | / | / |
|  |  |  | 68.1±7.5 years |  |  |  |  |  |  |  |
|  |  |  | 22.4% |  |  |  |  |  |  |  |
|  |  |  | NR |  |  |  |  |  |  |  |
|  |  |  | NR |  |  |  |  |  |  |  |
| Rienstra et al, 2004^494^ | Netherlands | RCT | 522 | SF-36 [Dutch] | / | / | / | / | / | / |
|  |  |  | Lone AF group (n=89): 65±10 years & AF with underlying diseases group (n=433): 69±8 years |  |  |  |  |  |  |  |
|  |  |  | Lone AF group: 16% & AF with underlying diseases group: 41% |  |  |  |  |  |  |  |
|  |  |  | NR |  |  |  |  |  |  |  |
|  |  |  | Persistent (all participants) |  |  |  |  |  |  |  |
| Rienstra et al, 2013^495^ | USA | Cohort study | 3,609 | / | Rosow-Breslau Functional Health Scale | / | / | / | / | / |
|  |  |  | 73±8 years |  |  |  |  |  |  |  |
|  |  |  | 59% |  |  |  |  |  |  |  |
|  |  |  | NR |  |  |  |  |  |  |  |
|  |  |  | NR |  |  |  |  |  |  |  |
| Rienstra et al, 2005^496^ | Netherlands | RCT | 522 | SF-36 [Dutch] | / | / | / | / | / | / |
|  |  |  | Female patients group (n=192): 71±8 years & male patients group (n=330): 67±9 years |  |  |  |  |  |  |  |
|  |  |  | 36.8% |  |  |  |  |  |  |  |
|  |  |  | NR |  |  |  |  |  |  |  |
|  |  |  | Persistent (all participants) |  |  |  |  |  |  |  |
| Rienstra et al, 2014^497^ | Netherlands | RCT | 522 | SF-36 [Dutch] | / | / | / | / | / | / |
|  |  |  | Asymptomatic AF group (n=157): 67±9 years & symptomatic AF group (n=365): 69±9 years |  |  |  |  |  |  |  |
|  |  |  | Asymptomatic AF group: 28% & symptomatic AF group: 40% |  |  |  |  |  |  |  |
|  |  |  | NR |  |  |  |  |  |  |  |
|  |  |  | Persistent (all participants) |  |  |  |  |  |  |  |
| Rillig et al, 2018^498^ | Germany | Pilot cohort study | 23 | / | / | / | MoCA | / | / | / |
|  |  |  | 74.1±10.5 years |  |  |  |  |  |  |  |
|  |  |  | 30% |  |  |  |  |  |  |  |
|  |  |  | NR |  |  |  |  |  |  |  |
|  |  |  | Paroxysmal (39.1%), persistent (39.1%) & permanent (21.7%) |  |  |  |  |  |  |  |
| Rinaldi et al, 2024^499^ | Italy | Cohort study | 20 | SF-36 | / | / | / | / | / | / |
|  |  |  | Participants aged 18-75 years enrolled |  |  |  |  |  |  |  |
|  |  |  | NR |  |  |  |  |  |  |  |
|  |  |  | NR |  |  |  |  |  |  |  |
|  |  |  | Paroxysmal (all participants) |  |  |  |  |  |  |  |
| Risom et al, 2018^500^ | Denmark | RCT | 210 | / | / | HADS | / | / | / | / |
|  |  |  | Intervention group: 60±9.7 years & control group: 59±12.3 years |  |  |  |  |  |  |  |
|  |  |  | Intervention group: 29.5% & control group: 26.7% |  |  |  |  |  |  |  |
|  |  |  | NR |  |  |  |  |  |  |  |
|  |  |  | Paroxysmal (72.4%) & persistent (27.6%) |  |  |  |  |  |  |  |
| Risom et al, 2019^501^ | Denmark | Mixed methods study | 10 | SF-36 | / | HADS | / | / | / | / |
|  |  |  | 54.6±11.6 years |  |  |  |  |  |  |  |
|  |  |  | 40% |  |  |  |  |  |  |  |
|  |  |  | NR |  |  |  |  |  |  |  |
|  |  |  | Paroxysmal (60%) |  |  |  |  |  |  |  |
| Risom et al, 2023^502^ | Denmark | Cross-sectional study | 929 | / | / | HADS | / | / | / | / |
|  |  |  | 60.0±9.8 years |  |  |  |  |  |  |  |
|  |  |  | 26% |  |  |  |  |  |  |  |
|  |  |  | NR |  |  |  |  |  |  |  |
|  |  |  | NR |  |  |  |  |  |  |  |
| Risom et al, 2016^503^ | Denmark | RCT | 210 | SF-36 | / | / | / | / | IPAQ | / |
|  |  |  | Cardiac rehabilitation group (n=105): 60±9 years & usual care group (n=105): 59±12.25 years |  |  |  |  |  |  |  |
|  |  |  | Cardiac rehabilitation group: 30% & usual care group: 27% |  |  |  |  |  |  |  |
|  |  |  | NR |  |  |  |  |  |  |  |
|  |  |  | Paroxysmal (72%) & persistent (28%) |  |  |  |  |  |  |  |
| Risom et al, 2020^504^ | Denmark | RCT | 210 | AFEQT & SF-36 | / | HADS | / | / | / | / |
|  |  |  | 59 years |  |  |  |  |  |  |  |
|  |  |  | 26% |  |  |  |  |  |  |  |
|  |  |  | NR |  |  |  |  |  |  |  |
|  |  |  | Paroxysmal (72%) |  |  |  |  |  |  |  |
| Risom et al, 2019 [a] ^505^ | Denmark | Cross-sectional study | 276 with AF (total n=627) | SF-36 | / | HADS | / | / | / | / |
|  |  |  | 60.1±9.5 years |  |  |  |  |  |  |  |
|  |  |  | 29% |  |  |  |  |  |  |  |
|  |  |  | NR |  |  |  |  |  |  |  |
|  |  |  | NR |  |  |  |  |  |  |  |
| Ritchie et al, 2023^506^ | UK | Pilot RCT | 21 | AFEQT & EQ-5D-3L | BADL | / | / | / | / | / |
|  |  |  | 82.9±6.9 years |  |  |  |  |  |  |  |
|  |  |  | 61.9% |  |  |  |  |  |  |  |
|  |  |  | White (100%) |  |  |  |  |  |  |  |
|  |  |  | Paroxysmal (42.9%), permanent (9.5%) & unclassified (47.6%) |  |  |  |  |  |  |  |
| Roalfe et al, 2012^507^ | UK | Cross-sectional study | 1,666 | EQ-5D & SF-12 | / | / | / | / | / | / |
|  |  |  | 82 years |  |  |  |  |  |  |  |
|  |  |  | 46.7% |  |  |  |  |  |  |  |
|  |  |  | NR |  |  |  |  |  |  |  |
|  |  |  | NR |  |  |  |  |  |  |  |
| Rosenstrøm et al, 2023^508^ | Denmark | RCT | 68 | AFEQT | / | HADS | / | / | / | / |
|  |  |  | 68 years |  |  |  |  |  |  |  |
|  |  |  | Intervention group (n=33): 33% & control group (n=35): 37% |  |  |  |  |  |  |  |
|  |  |  | NR |  |  |  |  |  |  |  |
|  |  |  | Intervention group: paroxysmal (39%), persistent (9%), permanent (18%) & unknown (33%)  Control group: paroxysmal (37%), persistent (29%), permanent (9%) & unknown (26%) |  |  |  |  |  |  |  |
| Rosman et al, 2021^509^ | USA | Cohort study | 68 | AFEQT & PROMIS-29 | / | / | / | / | / | / |
|  |  |  | 73.4±10.0 years |  |  |  |  |  |  |  |
|  |  |  | 42.6% |  |  |  |  |  |  |  |
|  |  |  | White (97.1%), African American (1.5%) & Other (1.5%) |  |  |  |  |  |  |  |
|  |  |  | Paroxysmal (76.5%) & permanent or persistent (23.5%) |  |  |  |  |  |  |  |
| Rowin et al, 2024^510^ | USA | Cohort study | 50 | AFEQT | / | / | / | / | / | / |
|  |  |  | 62±9 years |  |  |  |  |  |  |  |
|  |  |  | AFEQT ≥90 group (n=26): 31% AFEQT 70-89 group (n=11): 18% & AFEQT <70 group (n=13): 31% |  |  |  |  |  |  |  |
|  |  |  | NR |  |  |  |  |  |  |  |
|  |  |  | Paroxysmal (82%), persistent (10%) & permanent (8%) |  |  |  |  |  |  |  |
| Rush et al, 2023^511^ | Canada | Cross-sectional study | 196 | AFEQT & SRH | / | PSS-10 | / | / | Phone-FITT | / |
|  |  |  | 65.3±10.3 years |  |  |  |  |  |  |  |
|  |  |  | 37% |  |  |  |  |  |  |  |
|  |  |  | Caucasian (90%) |  |  |  |  |  |  |  |
|  |  |  | Paroxysmal (69.1%), persistent (26.8%), permanent (2.1%) & other (2.1%) |  |  |  |  |  |  |  |
| Sabatini et al, 2000^512^ | Italy | Cohort study | 42 with AF (total n=255) | / | BADL & IADL | GDS | MMSE | / | / | / |
|  |  |  | 81.5±4.8 years |  |  |  |  |  |  |  |
|  |  |  | 36% |  |  |  |  |  |  |  |
|  |  |  | NR |  |  |  |  |  |  |  |
|  |  |  | Chronic non-rheumatic (all participants) |  |  |  |  |  |  |  |
| Salvadori et al, 2023^513^ | Italy | Cohort study | 182 | / | BADL & IADL | / | MoCA | / | / | / |
|  |  |  | 77.7±6.7 years |  |  |  |  |  |  |  |
|  |  |  | 37% |  |  |  |  |  |  |  |
|  |  |  | NR |  |  |  |  |  |  |  |
|  |  |  | Paroxysmal (48%) |  |  |  |  |  |  |  |
| Samuel et al, 2020^514^ | Canada | RCT | 346 | AFEQT | / | / | / | / | / | / |
|  |  |  | Median age reported: 60 (IQR range 52-66) years |  |  |  |  |  |  |  |
|  |  |  | 33.3% |  |  |  |  |  |  |  |
|  |  |  | NR |  |  |  |  |  |  |  |
|  |  |  | Low-burden paroxysmal (29.5%), high-burden paroxysmal (65.3%) & persistent (5.2%) |  |  |  |  |  |  |  |
| Sandhu et al, 2017^515^ | Canada | Cohort study | 49 with AF (total n=100) | AFEQT & SF-36 | / | / | / | / | / | / |
|  |  |  | Median age reported: 60 (IQR range 56-68) years |  |  |  |  |  |  |  |
|  |  |  | 16.3% |  |  |  |  |  |  |  |
|  |  |  | NR |  |  |  |  |  |  |  |
|  |  |  | Non-paroxysmal (91.8%) |  |  |  |  |  |  |  |
| Sang et al, 2013^516^ | China | Cohort study | 166 | SF-36 [Chinese] | / | SAS [Chinese] & SDS [Chinese] | / | / | / | / |
|  |  |  | Ablation group (n=82): 55.9±6.1 years & antiarrhythmic drug group (n=84): 57.2±5.4 years |  |  |  |  |  |  |  |
|  |  |  | Ablation group: 32.9% & antiarrhythmic drug group: 30.9% |  |  |  |  |  |  |  |
|  |  |  | NR |  |  |  |  |  |  |  |
|  |  |  | Paroxysmal (all participants) |  |  |  |  |  |  |  |
| Sanghai et al, 2020^517^ | USA | Cohort study | 460 | / | / | PHQ-9 | MoCA | / | / | / |
|  |  |  | 74±7 years |  |  |  |  |  |  |  |
|  |  |  | 49% |  |  |  |  |  |  |  |
|  |  |  | Non-Hispanic White (82%) |  |  |  |  |  |  |  |
|  |  |  | Paroxysmal (64.1%), persistent or long-standing persistent (25.2%) & permanent (3.9%) |  |  |  |  |  |  |  |
| Sarnholm et al, 2023^518^ | Sweden | RCT | 127 | AFEQT | WHODAS 2.0 | CAQ | / | AFSS & SCL | / | / |
|  |  |  | 65.4±8.3 years |  |  |  |  |  |  |  |
|  |  |  | 58% |  |  |  |  |  |  |  |
|  |  |  | NR |  |  |  |  |  |  |  |
|  |  |  | Paroxysmal (all participants) |  |  |  |  |  |  |  |
| Särnholm et al, 2017^519^ | Sweden | Pilot cohort study | 19 | AFEQT & SF-36 | / | GAD-7, PHQ-9, PSS-10 & PHQ-15 | / | / | / | / |
|  |  |  | 60.5±9.5 years |  |  |  |  |  |  |  |
|  |  |  | 37% |  |  |  |  |  |  |  |
|  |  |  | NR |  |  |  |  |  |  |  |
|  |  |  | Paroxysmal (all participants) |  |  |  |  |  |  |  |
| Scheuermeyer et al, 2019^520^ | Canada | RCT | 84 | SF-8 | / | / | / | / | / | / |
|  |  |  | Median age reported: Chemical-first group (n=41):58 (IQR range 50-66) years & electrical-first group (n=43): 60 (IQR range 53-66) years |  |  |  |  |  |  |  |
|  |  |  | Chemical-first group: 36.6% & electrical-first group: 39.5% |  |  |  |  |  |  |  |
|  |  |  | NR |  |  |  |  |  |  |  |
|  |  |  | NR |  |  |  |  |  |  |  |
| Schilling et al, 2021^521^ | 4 European countries (not specified) | Cohort study | 87 | AFEQT | / | / | / | / | / | / |
|  |  |  | 60.2±10.05 years |  |  |  |  |  |  |  |
|  |  |  | 35.6% |  |  |  |  |  |  |  |
|  |  |  | NR |  |  |  |  |  |  |  |
|  |  |  | Paroxysmal (all participants) |  |  |  |  |  |  |  |
| Schmidt et al, 2019^522^ | NR | Cohort study | 104 | / | / | STAI | MoCA | / | / | / |
|  |  |  | 61.5±9.7 years |  |  |  |  |  |  |  |
|  |  |  | 30.8% |  |  |  |  |  |  |  |
|  |  |  | NR |  |  |  |  |  |  |  |
|  |  |  | Paroxysmal (all participants) |  |  |  |  |  |  |  |
| Schnabel et al, 2013^523^ | Germany | Cross-sectional study | 309 with AF (total n=9,989) | / | / | PHQ-9 | / | / | / | / |
|  |  |  | 64.8±8.2 years |  |  |  |  |  |  |  |
|  |  |  | 30.1% |  |  |  |  |  |  |  |
|  |  |  | NR |  |  |  |  |  |  |  |
|  |  |  | NR |  |  |  |  |  |  |  |
| Schnabel et al, 2018^524^ | France, Germany, Italy, Switzerland, Spain & UK | Cohort study | 6,196 | EQ-5D-5L | / | / | / | / | / | / |
|  |  |  | 71.8±10.4 years |  |  |  |  |  |  |  |
|  |  |  | 39.7% |  |  |  |  |  |  |  |
|  |  |  | NR |  |  |  |  |  |  |  |
|  |  |  | NR |  |  |  |  |  |  |  |
| Schron et al, 2014^525^ | Canada & USA | RCT | 693 | SF-36 & QLI-CV | / | / | / | / | / | / |
|  |  |  | 69.8±8.2 years |  |  |  |  |  |  |  |
|  |  |  | 37.8% |  |  |  |  |  |  |  |
|  |  |  | White (93%) & Minority (7%) |  |  |  |  |  |  |  |
|  |  |  | NR |  |  |  |  |  |  |  |
| Seki et al, 2022^526^ | Japan | Cohort study (registry) | 1,646 | AFEQT | / | / | / | / | / | / |
|  |  |  | Median age reported: 67 (IQR range 59-73) years |  |  |  |  |  |  |  |
|  |  |  | After propensity score matching: catheter ablation group (n=823): 25.4% & medical therapy group (n=823): 27.9% |  |  |  |  |  |  |  |
|  |  |  | NR |  |  |  |  |  |  |  |
|  |  |  | Catheter ablation group: paroxysmal (54.7%)  Medical therapy group: paroxysmal (51.2%) |  |  |  |  |  |  |  |
| Segan et al, 2024^527^ | 3 countries (not specified) | RCT | 338 | AFEQT | / | HADS | / | / | / | / |
|  |  |  | Median age reported: women group (n=79): 70.4 (IQR range 64.8-74.6) years & men group (n=259): 64.0 (IQR range 56.7-69.7) years |  |  |  |  |  |  |  |
|  |  |  | 23.4% |  |  |  |  |  |  |  |
|  |  |  | NR |  |  |  |  |  |  |  |
|  |  |  | Persistent (all participants) |  |  |  |  |  |  |  |
| Seligman et al, 2020^528^ | 11 countries in Europe, North America, Latin America, Middle East & Asia-Pacific (not specified) | Mixed methods study | 30 | AFEQT, SF-12 & PROMIS global health | / | / | PROMIS Cognitive function | AFSS | / | WPAI |
|  |  |  | NR |  |  |  |  |  |  |  |
|  |  |  | NR |  |  |  |  |  |  |  |
|  |  |  | NR |  |  |  |  |  |  |  |
|  |  |  | NR |  |  |  |  |  |  |  |
| Seliutskiiet al, 2020^529^ | Russia | Cohort study | 130 | SF-36 | / | / | / | / | / | / |
|  |  |  | 62.8±11.8 years |  |  |  |  |  |  |  |
|  |  |  | 35% |  |  |  |  |  |  |  |
|  |  |  | NR |  |  |  |  |  |  |  |
|  |  |  | Paroxysmal (46%) & persistent (54%) |  |  |  |  |  |  |  |
| Semaan et al, 2020^530^ | USA | Cohort study | 234 with AF (total n=3,333) | AFEQT | / | / | / | / | / | / |
|  |  |  | 62.2±12.1 years |  |  |  |  |  |  |  |
|  |  |  | 45% |  |  |  |  |  |  |  |
|  |  |  | White (92%), African American (1%), Asian or Pacific Islander (2%), Hispanic (4%) & Other (1%) |  |  |  |  |  |  |  |
|  |  |  | NR |  |  |  |  |  |  |  |
| Senoo et al, 2022^531^ | Japan | Cross-sectional study | 1,041 | SF-12 [Japanese] | / | GDS-15 [Japanese] | / | / | / | / |
|  |  |  | 72±5.6 years |  |  |  |  |  |  |  |
|  |  |  | 46.6% |  |  |  |  |  |  |  |
|  |  |  | NR |  |  |  |  |  |  |  |
|  |  |  | NR |  |  |  |  |  |  |  |
| Serber et al, 2023^532^ | USA | Cohort study | 96 | IIRS | / | CES-D & STAI | / | / | / | / |
|  |  |  | 62.2±11.8 years |  |  |  |  |  |  |  |
|  |  |  | 28% |  |  |  |  |  |  |  |
|  |  |  | NR |  |  |  |  |  |  |  |
|  |  |  | NR |  |  |  |  |  |  |  |
| Serpytis et al, 2018^533^ | Lithuania | Case-control study | 30 with AF (total n=60) | / | / | HADS | MMSE | / | / | / |
|  |  |  | 63.9±6.4 years |  |  |  |  |  |  |  |
|  |  |  | 37% |  |  |  |  |  |  |  |
|  |  |  | NR |  |  |  |  |  |  |  |
|  |  |  | Paroxysmal (26.7%), persistent (63.3%) & permanent (10%) |  |  |  |  |  |  |  |
| Shah et al, 2021^534^ | USA | Cross-sectional study | 779 | / | ADL & IADL | / | / | / | / | / |
|  |  |  | Median age reported: 80 (IQR range 75-86) years |  |  |  |  |  |  |  |
|  |  |  | 50% |  |  |  |  |  |  |  |
|  |  |  | White (93%), Black (4%) & Other (4%) |  |  |  |  |  |  |  |
|  |  |  | NR |  |  |  |  |  |  |  |
| Shamoto et al, 2012^535^ | Japan | Cohort study | 62 | AFQLQ | / | / | / | / | / | / |
|  |  |  | Retrospective study (n=62): 68±12 years & follow up study (n=28): 67±10 years |  |  |  |  |  |  |  |
|  |  |  | Retrospective study: 19.4% & follow up study: 17.9% |  |  |  |  |  |  |  |
|  |  |  | NR |  |  |  |  |  |  |  |
|  |  |  | Paroxysmal (all participants) |  |  |  |  |  |  |  |
| Shan et al, 2022^536^ | China | RCT | 90 | SF-12 | / | HAM-D & PHQ-9 | / | / | / | / |
|  |  |  | 62.81±9.0 years |  |  |  |  |  |  |  |
|  |  |  | 63.5% |  |  |  |  |  |  |  |
|  |  |  | NR |  |  |  |  |  |  |  |
|  |  |  | Cognitive behavioural therapy group (n=45): paroxysmal (23%), persistent (16%) & permanent (6%)  Treatment as usual group (n=45): paroxysmal (21%), persistent (15%) & permanent (9%) |  |  |  |  |  |  |  |
| Shantsila et al, 2020^537^ | UK | RCT | 250 | EQ-5D-5L | / | / | / | / | / | / |
|  |  |  | 72.3±7.4 years |  |  |  |  |  |  |  |
|  |  |  | Spironolactone group (n=125): 22% & placebo group (n=125): 25% |  |  |  |  |  |  |  |
|  |  |  | Spironolactone group: White (94%), Black (2%), Asian (2%) & Other (1%)  Placebo group: White (94%), Black (2%), Asian (2%) & Other (2%) |  |  |  |  |  |  |  |
|  |  |  | Permanent (all participants) |  |  |  |  |  |  |  |
| Sharples et al, 2018^538^ | UK | RCT | 352 | EQ-5D-3L & SF-36 | / | / | / | / | / | / |
|  |  |  | 71.9±7.67 years |  |  |  |  |  |  |  |
|  |  |  | 34.1% |  |  |  |  |  |  |  |
|  |  |  | NR |  |  |  |  |  |  |  |
|  |  |  | Paroxysmal (26.1%), persistent (13.9%) & chronic (59.9%) |  |  |  |  |  |  |  |
| She et al, 2019^539^ | China | Cross-sectional study | 143 | SF-36 | / | / | / | / | / | / |
|  |  |  | 67.0±9.0 years |  |  |  |  |  |  |  |
|  |  |  | 30% |  |  |  |  |  |  |  |
|  |  |  | NR |  |  |  |  |  |  |  |
|  |  |  | Permanent (all participants) |  |  |  |  |  |  |  |
| Shelton et al, 2009^540^ | UK | RCT | 61 | SF-36v2 | / | / | / | / | / | / |
|  |  |  | Rate group (n=31): 72.4±7.1 years & rhythm group (n=30): 72.7±8.3 years |  |  |  |  |  |  |  |
|  |  |  | Rate group: 16% & rhythm group: 19% |  |  |  |  |  |  |  |
|  |  |  | NR |  |  |  |  |  |  |  |
|  |  |  | Persistent (all participants) |  |  |  |  |  |  |  |
| Shiraishi et al, 2023^541^ | Japan | Cohort study (registry) | 530 | AFEQT | / | / | / | / | / |  |
|  |  |  | Median age reported: ablation group (n=75): 65 (IQR range 59-73) years & non-ablation group (n=455): 76 (IQR range 67-81) years |  |  |  |  |  |  |  |
|  |  |  | Ablation group: 23% & non-ablation group: 41% |  |  |  |  |  |  |  |
|  |  |  | NR |  |  |  |  |  |  |  |
|  |  |  | Ablation group: paroxysmal (31%), persistent (47%) & Other not specified (22%)  Non-ablation group: paroxysmal (23%), persistent (35%) & Other not specified (42%) |  |  |  |  |  |  |  |
| Si et al, 2019^542^ | China | Cohort study | 107 | SF-36 | / | / | / | / | / | / |
|  |  |  | Minimally invasive radiofrequency ablation group (n=42): 56.8±11.29 years & open surgery radiofrequency ablation group (n=65): 59.5±10.04 years |  |  |  |  |  |  |  |
|  |  |  | Minimally invasive radiofrequency ablation group: 57.1% & open surgery radiofrequency ablation group: 70.8% |  |  |  |  |  |  |  |
|  |  |  | NR |  |  |  |  |  |  |  |
|  |  |  | Minimally invasive radiofrequency ablation group: paroxysmal (11.9%) & persistent (88.1%)  Open surgery radiofrequency ablation group: paroxysmal (10.8%) & persistent (89.2%) |  |  |  |  |  |  |  |
| Simpson et al, 2001^543^ | NR | Pilot RCT | 14 | SF-36 | / | / | / | / | / | / |
|  |  |  | 62.9±8.5 years |  |  |  |  |  |  |  |
|  |  |  | 14.3% |  |  |  |  |  |  |  |
|  |  |  | NR |  |  |  |  |  |  |  |
|  |  |  | Paroxysmal (64.3%) & chronic (35.7%) |  |  |  |  |  |  |  |
| Singh et al, 2005^544^ | USA | RCT | 665 | SF-36 | / | / | / | / | / | / |
|  |  |  | 67.1±9.3 years |  |  |  |  |  |  |  |
|  |  |  | 1.1% |  |  |  |  |  |  |  |
|  |  |  | White (89.3%) & Black, Hispanic or Other (10.7%) |  |  |  |  |  |  |  |
|  |  |  | Persistent (all participants) |  |  |  |  |  |  |  |
| Singh et al, 2006^545^ | USA | RCT | 319 with AF (total n=577) | SF-36 | / | / | / | AFSS & SCL | Specific activity scale | / |
|  |  |  | 66.4±9.5 years |  |  |  |  |  |  |  |
|  |  |  | 1% |  |  |  |  |  |  |  |
|  |  |  | NR |  |  |  |  |  |  |  |
|  |  |  | Persistent (all participants) |  |  |  |  |  |  |  |
| Skuladottir et al, 2024^546^ | Sweden | RCT | 127 | AFEQT | / | CAQ | / | / | IPAQ | / |
|  |  |  | 65.4±8.3 years |  |  |  |  |  |  |  |
|  |  |  | 58% |  |  |  |  |  |  |  |
|  |  |  | NR |  |  |  |  |  |  |  |
|  |  |  | Paroxysmal (all participants) |  |  |  |  |  |  |  |
| Slawuta et al, 2020^547^ | Poland | Cohort study | 158 | ASTA HRQoL scale [Polish] | / | / | MMSE | ASTA symptom scale [Polish] | / | / |
|  |  |  | 70.4±7.6 years |  |  |  |  |  |  |  |
|  |  |  | 49.4% |  |  |  |  |  |  |  |
|  |  |  | NR |  |  |  |  |  |  |  |
|  |  |  | Permanent, paroxysmal & persistent (n or % not specified) |  |  |  |  |  |  |  |
| Smigorowsky et al, 2017^548^ | Canada | RCT (protocol) | 140 participants to be enrolled | AFEQT & EQ-5D | / | / | / | / | / | / |
|  |  |  | Participants aged ≥18 years to be enrolled |  |  |  |  |  |  |  |
|  |  |  | NR |  |  |  |  |  |  |  |
|  |  |  | NR |  |  |  |  |  |  |  |
|  |  |  | Non-valvular AF participants to be enrolled |  |  |  |  |  |  |  |
| Smith et al, 2010^549^ | UK | RCT (protocol) | 154 participants to be enrolled | AF-QoL | / | HADS | / | / | / | / |
|  |  |  | NR |  |  |  |  |  |  |  |
|  |  |  | NR |  |  |  |  |  |  |  |
|  |  |  | NR |  |  |  |  |  |  |  |
|  |  |  | NR |  |  |  |  |  |  |  |
| Son et al, 2018^550^ | South Korea | Cross-sectional study | 205 | AF-QoL [Korean] | / | / | / | / | / | / |
|  |  |  | 74.24±4.61 years |  |  |  |  |  |  |  |
|  |  |  | 43.9% |  |  |  |  |  |  |  |
|  |  |  | NR |  |  |  |  |  |  |  |
|  |  |  | NR |  |  |  |  |  |  |  |
| Son et al, 2012^551^ | South Korea | Cross-sectional study | 114 | SF-36 [Korean] | / | HADS | / | / | / | / |
|  |  |  | 67±12 years |  |  |  |  |  |  |  |
|  |  |  | 44.7% |  |  |  |  |  |  |  |
|  |  |  | NR |  |  |  |  |  |  |  |
|  |  |  | Chronic (all participants) |  |  |  |  |  |  |  |
| Spertus et al, 2011^552^ | Canada & USA | Methodological study | 214 | AFEQT, EQ-5D & SF-36 | / | GAD-7 | / | AFSS & SCL | / | / |
|  |  |  | 62.1±11.9 years |  |  |  |  |  |  |  |
|  |  |  | 42.5% |  |  |  |  |  |  |  |
|  |  |  | White (96.7%), African American (1.9%), Asian (0.5%) & Other (0.9%) |  |  |  |  |  |  |  |
|  |  |  | Paroxysmal (65.9%), persistent (23.9%), long-standing persistent (5.1%) & permanent (5.1%) |  |  |  |  |  |  |  |
| Springer et al, 2020^553^ | Switzerland | Cohort study | 2,415 | / | / | / | MoCA [German, French & Italian] | / | / | / |
|  |  |  | 73.24±8.4 years |  |  |  |  |  |  |  |
|  |  |  | 27.4% |  |  |  |  |  |  |  |
|  |  |  | NR |  |  |  |  |  |  |  |
|  |  |  | Persistent, paroxysmal & permanent (n or % not specified) |  |  |  |  |  |  |  |
| Spurrell et al, 2003^554^ | NR | Cohort study | 15 | SF-36 | / | HADS | / | SCL | / | / |
|  |  |  | 63±14 years |  |  |  |  |  |  |  |
|  |  |  | 20% |  |  |  |  |  |  |  |
|  |  |  | NR |  |  |  |  |  |  |  |
|  |  |  | Persistent (all participants) |  |  |  |  |  |  |  |
| Srichawla et al, 2024^555^ | USA | Cohort study | 887 | / | / | / | MoCA | / | / | / |
|  |  |  | 75.2±6.7 years |  |  |  |  |  |  |  |
|  |  |  | 48.6% |  |  |  |  |  |  |  |
|  |  |  | White non-Hispanic (87.4%) |  |  |  |  |  |  |  |
|  |  |  | Catheter ablation at baseline yes group (n=193) paroxysmal (61.1%), persistent (31.1%), permanent (2.1%) & Other (5.7%)  Catheter ablation at baseline no group (n=646) paroxysmal (59.8%), persistent (22.5%), permanent (5.9%) & Other (11.8%) |  |  |  |  |  |  |  |
| Srinonprasert et al, 2019^556^ | Thailand | Cross-sectional study | 3,218 | EQ-5D-3L [Thai] | / | / | / | / | / | / |
|  |  |  | 67.3±11.3 years |  |  |  |  |  |  |  |
|  |  |  | Age <65 group (n=1,255): 34.7% & age ≥65 group (n=1,963): 54.1% |  |  |  |  |  |  |  |
|  |  |  | NR |  |  |  |  |  |  |  |
|  |  |  | Age <65 group: paroxysmal (36.3%), persistent (19.7%) & permanent (44%)  Age ≥65 group: paroxysmal (31.5%), persistent (19.2%) & permanent (49.3%) |  |  |  |  |  |  |  |
| Stambler et al, 2003^557^ | USA | RCT | 103 | SF-36 | / | / | / | / | / | / |
|  |  |  | 69.5±12.4 years |  |  |  |  |  |  |  |
|  |  |  | 25% |  |  |  |  |  |  |  |
|  |  |  | NR |  |  |  |  |  |  |  |
|  |  |  | Chronic (all participants) |  |  |  |  |  |  |  |
| Stanley et al, 2024^558^ | South Africa | Cohort study (registry) | 81 | EQ-5D-3L | / | / | / | / | / | / |
|  |  |  | 60±12 years |  |  |  |  |  |  |  |
|  |  |  | 23.5% |  |  |  |  |  |  |  |
|  |  |  | NR |  |  |  |  |  |  |  |
|  |  |  | Paroxysmal (59.3%) & persistent (40.7%) |  |  |  |  |  |  |  |
| Stefansdottir et al, 2013^559^ | Iceland | Cross-sectional study | 330 with AF (total n=4251) | / | / | GDS | MMSE | / | / | / |
|  |  |  | 78.0±5.4 years |  |  |  |  |  |  |  |
|  |  |  | 37.6% |  |  |  |  |  |  |  |
|  |  |  | NR |  |  |  |  |  |  |  |
|  |  |  | Paroxysmal (41.8%) & permanent or persistent (58.2%) |  |  |  |  |  |  |  |
| Steg et al, 2012^560^ | Algeria, Azerbaijan, Belgium, Bulgaria, Czech Republic, Egypt, Germany, Hungary, India, Ireland, Italy, Lebanon, Lithuania, Mexico, Morocco, Portugal, Russia, Slovakia, Spain, Sweden, Switzerland, Taiwan, Tunisia, Turkey, Ukraine & Venezuela | Cross-sectional study | 10,523 | EQ-5D | / | / | / | / | / | / |
|  |  |  | 66.6±12.2 years |  |  |  |  |  |  |  |
|  |  |  | 43.6% |  |  |  |  |  |  |  |
|  |  |  | NR |  |  |  |  |  |  |  |
|  |  |  | Paroxysmal (24.8%), persistent (22.3%), permanent (46.4%) & first episode (6.4%) |  |  |  |  |  |  |  |
| Steinberg et al, 2019^561^ | NR | Analysis study | Sample size range reported: <100 participants (32.7%), 100-499 participants (41.8%), 500-999 participants (7.6%) & ≥1000 participants (17.9%) | AFEQT, AF-QoL-18, ASTA HRQoL scale, EQ-5D, SF-12 & SF-36 | / | / | / | AFSS, ASTA symptom scale, MAFSI & SCL | / | / |
|  |  |  | NR |  |  |  |  |  |  |  |
|  |  |  | NR |  |  |  |  |  |  |  |
|  |  |  | NR |  |  |  |  |  |  |  |
|  |  |  | Only paroxysmal (6.5%), paroxysmal (7.1%), persistent (4.7%), permanent (0.6%) & not specified (88.4%) |  |  |  |  |  |  |  |
| Steinberg et al, 2020^562^ | USA | Cohort study (registry) | 636 | AFEQT | / | / | / | / | / | / |
|  |  |  | Median age reported: 74 (IQR range 67-81) years |  |  |  |  |  |  |  |
|  |  |  | 47.2% |  |  |  |  |  |  |  |
|  |  |  | White (90.7%), Black or African American (4.1%), Hispanic (3.1%) & Other (1.9%) |  |  |  |  |  |  |  |
|  |  |  | First detected (10.8%), paroxysmal (46.5%) & persistent or permanent (42.6%) |  |  |  |  |  |  |  |
| Steinberg et al, 2020 [a] ^563^ | USA | Cohort study | 1,534 | / | PROMIS-Bank Physical Function | PROMIS-Bank Depression | / | AFSS | / | / |
|  |  |  | 68±12 years |  |  |  |  |  |  |  |
|  |  |  | 38.4% |  |  |  |  |  |  |  |
|  |  |  | American Indian and Alaska Native (0.5%), Asian (1.4%), Black or African American (.05%), Native Hawaiian or Pacific Islander (0.6%) & White or Caucasian (92.7%) |  |  |  |  |  |  |  |
|  |  |  | NR |  |  |  |  |  |  |  |
| Stewart et al, 2015^564^ | Australia | RCT | 335 | EQ-5D & SF-12 | / | CES-D | MoCA | / | / | / |
|  |  |  | 72±11 years |  |  |  |  |  |  |  |
|  |  |  | 48% |  |  |  |  |  |  |  |
|  |  |  | NR |  |  |  |  |  |  |  |
|  |  |  | Chronic non-valvular (all participants) |  |  |  |  |  |  |  |
| Stridsman et al, 2019^565^ | Sweden | Mixed methods study | 19 | ASTA HRQoL scale & SF-36 | / | HADS | / | ASTA symptom scale | / | / |
|  |  |  | Median age reported: 61 (IQR range 54-69) years |  |  |  |  |  |  |  |
|  |  |  | 37% |  |  |  |  |  |  |  |
|  |  |  | NR |  |  |  |  |  |  |  |
|  |  |  | Paroxysmal (74%) & persistent (26%) |  |  |  |  |  |  |  |
| Su et al, 2020^566^ | Canada, Japan & USA | Cohort study | 165 | AFEQT & SF-12 | / | / | / | / | / | / |
|  |  |  | 65±9 years |  |  |  |  |  |  |  |
|  |  |  | 29.7% |  |  |  |  |  |  |  |
|  |  |  | NR |  |  |  |  |  |  |  |
|  |  |  | Persistent (all participants) |  |  |  |  |  |  |  |
| Suárez et al, 2016^567^ | Spain | Methodological study | 1,337 | EQ-5D [Spanish] | / | / | / | / | / | / |
|  |  |  | 75.63±8.94 years |  |  |  |  |  |  |  |
|  |  |  | 44.2% |  |  |  |  |  |  |  |
|  |  |  | NR |  |  |  |  |  |  |  |
|  |  |  | Non-valvular (all participants) |  |  |  |  |  |  |  |
| Suleiman et al, 2012^568^ | USA | RCT | 125 | / | / | / | / | SCL | / | / |
|  |  |  | 57±10 years |  |  |  |  |  |  |  |
|  |  |  | 21.6% |  |  |  |  |  |  |  |
|  |  |  | NR |  |  |  |  |  |  |  |
|  |  |  | Paroxysmal (72%) & persistent (28%) |  |  |  |  |  |  |  |
| Sulke et al, 2007^569^ | UK | RCT | 182 | SF-36 | / | / | / | / | / | / |
|  |  |  | 72.6±9.4 years |  |  |  |  |  |  |  |
|  |  |  | 47.3% |  |  |  |  |  |  |  |
|  |  |  | NR |  |  |  |  |  |  |  |
|  |  |  | Paroxysmal (all participants) |  |  |  |  |  |  |  |
| Suman-Horduna et al, 2013^570^ | USA | RCT | 749 | SF-36 [Danish, English, French, Spanish & Portuguese] | / | / | / | / | / | / |
|  |  |  | 66±11 years |  |  |  |  |  |  |  |
|  |  |  | 17.1% |  |  |  |  |  |  |  |
|  |  |  | NR |  |  |  |  |  |  |  |
|  |  |  | Paroxysmal (31.1%) & not specified (68.9%) |  |  |  |  |  |  |  |
| Sun et al, 2021^571^ | China | Cross-sectional study | 254 | / | / | / | MoCA [Chinese] | / | / | / |
|  |  |  | 59.71±11.14 years |  |  |  |  |  |  |  |
|  |  |  | 44.5% |  |  |  |  |  |  |  |
|  |  |  | NR |  |  |  |  |  |  |  |
|  |  |  | Paroxysmal (29.9%), persistent (48.8%), long-term persistent (9.5%) & permanent (11.8%) |  |  |  |  |  |  |  |
| Sun et al, 2019^572^ | China | Cross-sectional study (registry) | 4,161 | SF-36 | / | / | / | / | / | / |
|  |  |  | 68.3±11.9 years |  |  |  |  |  |  |  |
|  |  |  | 46.9% |  |  |  |  |  |  |  |
|  |  |  | NR |  |  |  |  |  |  |  |
|  |  |  | New onset (12.3%), paroxysmal (32.4%) persistent (31.1%) & permanent (23.9%) |  |  |  |  |  |  |  |
| Szili-Torok et al, 2005^573^ | Netherlands | Cohort study | 25 | NHP [Dutch] | / | / | / | / | / | / |
|  |  |  | 63±11.4 years |  |  |  |  |  |  |  |
|  |  |  | 36% |  |  |  |  |  |  |  |
|  |  |  | NR |  |  |  |  |  |  |  |
|  |  |  | Permanent (all participants) |  |  |  |  |  |  |  |
| Tabaja et al, 2023^574^ | USA | Cohort study (registry) | 5,841 | / | / | / | / | AFSS | / | / |
|  |  |  | 64±10 years |  |  |  |  |  |  |  |
|  |  |  | 34% |  |  |  |  |  |  |  |
|  |  |  | Normal weight group (n=986): Black (1%) & not specified (99%) Overweight group (n=1,997): Black (2%) & not specified (98%) Class 1 obesity group (n=1,596): Black (3%) & not specified (97%) Class 2 obesity group (n=758): Black (4%) & not specified (96%)  Class 3 obesity group (n=504): Black (5%) & not specified (95%) |  |  |  |  |  |  |  |
|  |  |  | Paroxysmal (47%) & persistent (53%) |  |  |  |  |  |  |  |
| Tabaja et al, 2024^575^ | USA | Cohort study (registry) | 7,020 | / | / | / | / | AFSS | / | / |
|  |  |  | 68±4years |  |  |  |  |  |  |  |
|  |  |  | 30% |  |  |  |  |  |  |  |
|  |  |  | Non-elderly group (n=2,953): Black (4%) & not specified (97%) Elderly group (n=2,978): Black (2%) & not specified (98%) Very elderly group (n=1,089): Black (2%) & not specified (98%) |  |  |  |  |  |  |  |
|  |  |  | Non-elderly group: paroxysmal (54%) & persistent (46%) Elderly group: paroxysmal (45%) & persistent (55%) Very elderly group: paroxysmal (39%) & persistent (61%) |  |  |  |  |  |  |  |
| Tada et al, 2003^576^ | Japan | Cohort study | 50 | SF-36 [Japanese] | / | / | / | / | / | / |
|  |  |  | 58±7 years |  |  |  |  |  |  |  |
|  |  |  | 32% |  |  |  |  |  |  |  |
|  |  |  | NR |  |  |  |  |  |  |  |
|  |  |  | Paroxysmal (all participants) |  |  |  |  |  |  |  |
| Tailachidis et al, 2016^577^ | Greece | Methodological study | 102 | AFEQT [Greek] & SF-12 [Greek] | / | / | / | / | / | / |
|  |  |  | 70±9.2 years |  |  |  |  |  |  |  |
|  |  |  | 46.1% |  |  |  |  |  |  |  |
|  |  |  | NR |  |  |  |  |  |  |  |
|  |  |  | Paroxysmal (8.8%), persistent (31.4%) & permanent (59.8%) |  |  |  |  |  |  |  |
| Taishi et al, 2022^578^ | Japan | Cohort study (registry) | 2,211 | AFEQT | / | / | / | / | / | / |
|  |  |  | No tricuspid regurgitation group (n=1,104): 64.2±11.6 years Mild tricuspid regurgitation group (n=935): 68.7±10.3 years  Moderate tricuspid regurgitation group (n=159): 73.8±8.5 years  severe tricuspid regurgitation group (n=13): 76.1+7.9 years |  |  |  |  |  |  |  |
|  |  |  | No tricuspid regurgitation group: 21.3%,  Mild tricuspid regurgitation group: 37.9%  Moderate tricuspid regurgitation group: 44.7%  Severe tricuspid regurgitation group: 76.9% |  |  |  |  |  |  |  |
|  |  |  | NR |  |  |  |  |  |  |  |
|  |  |  | No tricuspid regurgitation group: first detected (6.6%), paroxysmal (61.8%) & non-paroxysmal (31.6%)  Mild tricuspid regurgitation group: first detected (5.9%), paroxysmal (48.4%) & non-paroxysmal (45.6%)  Moderate tricuspid regurgitation group: first detected (4.6%), paroxysmal (31.4%) & non-paroxysmal (64.1%)  Severe tricuspid regurgitation group: first detected (0%), paroxysmal (30.8%) & non-paroxysmal (69.2%) |  |  |  |  |  |  |  |
| Takahashi et al, 2003^579^ | Japan | Cohort study | 38 | WHOQoL-BREF | / | / | / | SCL | / | / |
|  |  |  | 69±12 years |  |  |  |  |  |  |  |
|  |  |  | 50% |  |  |  |  |  |  |  |
|  |  |  | NR |  |  |  |  |  |  |  |
|  |  |  | Paroxysmal (73.7%) & persistent (26.3%) |  |  |  |  |  |  |  |
| Tan et al, 2008^580^ | Netherlands | Cohort study | 121 | SF-36 [Dutch] | / | / | / | / | / | / |
|  |  |  | 65±11 years |  |  |  |  |  |  |  |
|  |  |  | 51% |  |  |  |  |  |  |  |
|  |  |  | NR |  |  |  |  |  |  |  |
|  |  |  | Paroxysmal (34%) & permanent (66%) |  |  |  |  |  |  |  |
| Tan et al, 2018^581^ | Singapore | Cross-sectional study | 116 | AFEQT & SF-12v2 [Chinese] | / | PHQ-9 [Chinese] | / | AFSS [Chinese] | / | / |
|  |  |  | 67.4±10.9 years |  |  |  |  |  |  |  |
|  |  |  | 31.9% |  |  |  |  |  |  |  |
|  |  |  | Chinese (73.3%), Malay (19%), Indian (1.7%) & Other (6%) |  |  |  |  |  |  |  |
|  |  |  | NR |  |  |  |  |  |  |  |
| Tang et al, 2023^582^ | Taiwan | Cohort study | 253 | SF-36 | / | CES-D | MoCA [Mandarin] | / | / | / |
|  |  |  | AF burden below median group (n=127):61.0±10.0 years & AF burden above median group (n=126): 64.1±10.7 years |  |  |  |  |  |  |  |
|  |  |  | AF burden below median group: 33.1% & AF burden above median group: 22.2% |  |  |  |  |  |  |  |
|  |  |  | NR |  |  |  |  |  |  |  |
|  |  |  | NR |  |  |  |  |  |  |  |
| Tankut et al, 2023^583^ | USA | RCT (protocol) | 120 participants to be enrolled | AFEQT | / | / | / | / | / | / |
|  |  |  | Participants aged ≥50 years to be enrolled |  |  |  |  |  |  |  |
|  |  |  | NR |  |  |  |  |  |  |  |
|  |  |  | NR |  |  |  |  |  |  |  |
|  |  |  | Paroxysmal or persistent AF participants to be enrolled |  |  |  |  |  |  |  |
| Tao et al, 2023^584^ | China | Cohort study | 158 | / | / | / | MMSE & MoCA [Chinese] | / | / | / |
|  |  |  | 63.6±9.6 years |  |  |  |  |  |  |  |
|  |  |  | 39.9% |  |  |  |  |  |  |  |
|  |  |  | NR |  |  |  |  |  |  |  |
|  |  |  | Persistent or permanent (44.9%) & paroxysmal (55.1%) |  |  |  |  |  |  |  |
| Tatewaki et al, 2022^585^ | Japan | Pilot study | 8 | / | / | CES-D & STAI | MMSE [Japanese] | / | / | / |
|  |  |  | 63.4±7.3 years |  |  |  |  |  |  |  |
|  |  |  | 25% |  |  |  |  |  |  |  |
|  |  |  | NR |  |  |  |  |  |  |  |
|  |  |  | Persistent (all participants) |  |  |  |  |  |  |  |
| Tavassoli et al, 2013^586^ | France | Cross-sectional study | 66 with AF (total n=686) | / | ADL & IADL | / | MMSE | / | / | / |
|  |  |  | 80.5±5.8 years |  |  |  |  |  |  |  |
|  |  |  | 63.6% |  |  |  |  |  |  |  |
|  |  |  | NR |  |  |  |  |  |  |  |
|  |  |  | NR |  |  |  |  |  |  |  |
| Taylor et al, 2020^587^ | UK | Cross-sectional study | 118 | / | / | PHQ-8 | / | / | / | / |
|  |  |  | 63.52±8.82 years |  |  |  |  |  |  |  |
|  |  |  | 23.7% |  |  |  |  |  |  |  |
|  |  |  | White (99.2%) & Other (0.9%) |  |  |  |  |  |  |  |
|  |  |  | Persistent (all participants) |  |  |  |  |  |  |  |
| Taylor et al, 2022^588^ | UK | Cross-sectional study | 198 | AFEQT | / | GAD-7 & PHQ-8 | / | / | / | / |
|  |  |  | 64±9.0 years |  |  |  |  |  |  |  |
|  |  |  | 23% |  |  |  |  |  |  |  |
|  |  |  | White (97%) & Other (3%) |  |  |  |  |  |  |  |
|  |  |  | Persistent (all participants) |  |  |  |  |  |  |  |
| Tendolkar et al, 2012^589^ | Netherlands | RCT | 34 | / | / | / | MMSE | / | / | / |
|  |  |  | Placebo group (n=17): 73.5±4.0 years & treatment group (n=17): 74.5±4.2 years |  |  |  |  |  |  |  |
|  |  |  | 20.6% |  |  |  |  |  |  |  |
|  |  |  | NR |  |  |  |  |  |  |  |
|  |  |  | Chronic (58.8%) & paroxysmal (41.2%) |  |  |  |  |  |  |  |
| Terricabras et al, 2020^590^ | Australia, Canada, China, Korea & European countries (not specified) | RCT | 549 | SF-36 & EQ-5D-3L | / | / | / | / | / | / |
|  |  |  | 60±9 years |  |  |  |  |  |  |  |
|  |  |  | 21% |  |  |  |  |  |  |  |
|  |  |  | NR |  |  |  |  |  |  |  |
|  |  |  | Persistent (all participants) |  |  |  |  |  |  |  |
| Thind et al, 2018^591^ | USA | Cohort study (registry) | 9,319 | / | / | / | / | SCL | / | / |
|  |  |  | Median age reported: 75 (IQR range 67-82) years |  |  |  |  |  |  |  |
|  |  |  | 42.3% |  |  |  |  |  |  |  |
|  |  |  | White (89.5%), Black (4.8%), Hispanic (4%) & Other (1.4%) |  |  |  |  |  |  |  |
|  |  |  | New onset (4.6%), paroxysmal (51.3%) & persistent or permanent (44.2%) |  |  |  |  |  |  |  |
| Thompson et al, 2014^592^ | USA | Cohort study | 378 | / | / | HADS-A & PHQ-9 | / | AFSS | / | / |
|  |  |  | 61.6±13.3 years |  |  |  |  |  |  |  |
|  |  |  | 33.8% |  |  |  |  |  |  |  |
|  |  |  | NR |  |  |  |  |  |  |  |
|  |  |  | Paroxysmal (42.4%) & persistent (57.6%) |  |  |  |  |  |  |  |
| Thomson et al, 2007^593^ | UK | RCT | 109 | / | / | STAI | / | / | / | / |
|  |  |  | Guidelines group (n=56): 73.7±6.2 years & decision aid group (n=53): 73.1±6.7 years |  |  |  |  |  |  |  |
|  |  |  | Guidelines group: 44.6% & decision aid group: 43.4% |  |  |  |  |  |  |  |
|  |  |  | NR |  |  |  |  |  |  |  |
|  |  |  | Chronic non-valvular & paroxysmal (n or % not specified) |  |  |  |  |  |  |  |
| Thrall et al, 2007^594^ | UK | Cross-sectional study | 101 with AF (total n=197) | Dartmouth COOP chart | / | BDI & STAI | / | / | / | / |
|  |  |  | 66.3±11.0 years |  |  |  |  |  |  |  |
|  |  |  | 38.6% |  |  |  |  |  |  |  |
|  |  |  | White (95%), Afro-Caribbean (3%) & South Asian (2%) |  |  |  |  |  |  |  |
|  |  |  | Paroxysmal (52.5%), persistent (5.9%) & permanent (41.6%) |  |  |  |  |  |  |  |
| Tian et al, 2023^595^ | China | Cross sectional study | 223 | AFEQT [Chinese] | / | / | / | / | / | / |
|  |  |  | Well-functioning family group (n=64): 64.86±9.16 years & family dysfunction group (n=159): 62.15±11.28 years |  |  |  |  |  |  |  |
|  |  |  | Well-functioning family group: 57.8% & family dysfunction group: 39.2% |  |  |  |  |  |  |  |
|  |  |  | NR |  |  |  |  |  |  |  |
|  |  |  | Paroxysmal & persistent (n or % not specified) |  |  |  |  |  |  |  |
| Tischer et al, 2019^596^ | NR | RCT | 45 | / | / | / | MMSE & MoCA | / | / | / |
|  |  |  | AF group (n=21): 68.8±7.0 years & AF or HF group (n=24): 67.3±7.8 years |  |  |  |  |  |  |  |
|  |  |  | 26.7% |  |  |  |  |  |  |  |
|  |  |  | NR |  |  |  |  |  |  |  |
|  |  |  | NR |  |  |  |  |  |  |  |
| Tondo et al, 2006^597^ | NR | Cohort study | 105 | SF-36 | / | / | / | / | / | / |
|  |  |  | Patients with congestive HF group (n=40): 57±10 years & patients without congestive HF group (n=65): 56±8 years |  |  |  |  |  |  |  |
|  |  |  | Patients with congestive HF group: 30% & patients without congestive HF group: 11% |  |  |  |  |  |  |  |
|  |  |  | NR |  |  |  |  |  |  |  |
|  |  |  | Patients with congestive HF group: paroxysmal (25%) & persistent (75%) Patients without congestive HF group: paroxysmal (23%) & persistent (76%) |  |  |  |  |  |  |  |
| Tondo et al, 2014^598^ | Italy | Cohort study | 143 | SF-12 | / | / | / | / | / | / |
|  |  |  | 59±9 years |  |  |  |  |  |  |  |
|  |  |  | 15% |  |  |  |  |  |  |  |
|  |  |  | NR |  |  |  |  |  |  |  |
|  |  |  | Paroxysmal (55%) & persistent (45%) |  |  |  |  |  |  |  |
| Toso et al, 2022^599^ | Italy | Cohort study | 133 | SF-36 | / | / | / | / | / | / |
|  |  |  | 48±9 years |  |  |  |  |  |  |  |
|  |  |  | 4% |  |  |  |  |  |  |  |
|  |  |  | NR |  |  |  |  |  |  |  |
|  |  |  | Paroxysmal (60%), long-standing persistent (7%) & persistent (33%) |  |  |  |  |  |  |  |
| Tripathi et al, 2023^600^ | India | Cohort study | 25 | AFEQT | / | / | / | / | / | / |
|  |  |  | 34.43±12.3 years |  |  |  |  |  |  |  |
|  |  |  | 48% |  |  |  |  |  |  |  |
|  |  |  | NR |  |  |  |  |  |  |  |
|  |  |  | Rheumatic (all participants) |  |  |  |  |  |  |  |
| Trymbulak et al, 2020^601^ | USA | Cohort study | 40 | / | / | PHQ-9 | / | / | / | / |
|  |  |  | 70.9±4.8 years |  |  |  |  |  |  |  |
|  |  |  | 37.5% |  |  |  |  |  |  |  |
|  |  |  | White (95%) & not specified (5%) |  |  |  |  |  |  |  |
|  |  |  | Paroxysmal (67.5%) |  |  |  |  |  |  |  |
| Tse et al, 2001^602^ | China | RCT | 16 | SF-36 | / | / | / | SCL | / | / |
|  |  |  | 63±9 years |  |  |  |  |  |  |  |
|  |  |  | Amiodarone group (n=9): 22% & digoxin group (n=7): 14% |  |  |  |  |  |  |  |
|  |  |  | NR |  |  |  |  |  |  |  |
|  |  |  | Chronic (all participants) |  |  |  |  |  |  |  |
| Tse et al, 2004^603^ | Canada, China, Germany Netherlands, UK & USA | RCT | 90 | SF-36 | DASI | / | / | SCL | / | / |
|  |  |  | Paroxysmal AF group (n=45): 74±9 years & persistent AF group (n=45): 70±11 years |  |  |  |  |  |  |  |
|  |  |  | Paroxysmal AF group: 56% & persistent AF group: 44% |  |  |  |  |  |  |  |
|  |  |  | NR |  |  |  |  |  |  |  |
|  |  |  | Paroxysmal (50%) & persistent (50%) |  |  |  |  |  |  |  |
| Tse et al, 2005^604^ | NR | Cohort study | 46 | SF-36 [Chinese] | / | / | / | SCL | / | / |
|  |  |  | 51±14 years |  |  |  |  |  |  |  |
|  |  |  | 26.1% |  |  |  |  |  |  |  |
|  |  |  | NR |  |  |  |  |  |  |  |
|  |  |  | Paroxysmal (all participants) |  |  |  |  |  |  |  |
| Tsounis et al, 2014^605^ | Greece | Cohort study | 108 | EQ-5D [Greek] & SF-36 [Greek] | / | / | / | / | / | / |
|  |  |  | 65.4±12.6 years |  |  |  |  |  |  |  |
|  |  |  | 36% |  |  |  |  |  |  |  |
|  |  |  | NR |  |  |  |  |  |  |  |
|  |  |  | First diagnosed (16.7%), paroxysmal or persistent (43.5%) & permanent (39.8%) |  |  |  |  |  |  |  |
| Tsuneda et al, 2006^606^ | Japan | Cohort study | 29 | AFQLQ & SF-36 [Japanese] | / | / | / | / | / | / |
|  |  |  | 67±8 years |  |  |  |  |  |  |  |
|  |  |  | 13.8% |  |  |  |  |  |  |  |
|  |  |  | NR |  |  |  |  |  |  |  |
|  |  |  | Permanent (all participants) |  |  |  |  |  |  |  |
| Turchioe et al, 2023^607^ | USA | Mixed methods study | 15 | / | / | / | / | AFSS | / | / |
|  |  |  | 71.1±8.6 years |  |  |  |  |  |  |  |
|  |  |  | 27% |  |  |  |  |  |  |  |
|  |  |  | White (67%) & not specified (33%) |  |  |  |  |  |  |  |
|  |  |  | NR |  |  |  |  |  |  |  |
| Turker et al, 2017^608^ | Turkey | Cohort study | 50 | / | / | BDI & HAM-A | / | / | / | / |
|  |  |  | 74.6±8.7 years |  |  |  |  |  |  |  |
|  |  |  | 44% |  |  |  |  |  |  |  |
|  |  |  | NR |  |  |  |  |  |  |  |
|  |  |  | Non-valvular (all participants) |  |  |  |  |  |  |  |
| Ueda et al, 2024^609^ | Japan | Cohort study (registry) | 2,636 | AFEQT |  |  |  |  |  |  |
|  |  |  | No oral anticoagulation concern group (n=1,864): 69.2±11.0 years & oral anticoagulation concern group (n=722): 68.1±10.8 years |  |  |  |  |  |  |  |
|  |  |  | No oral anticoagulation concern group: 29.8% & oral anticoagulation concern group: 36.9% |  |  |  |  |  |  |  |
|  |  |  | NR |  |  |  |  |  |  |  |
|  |  |  | No oral anticoagulation concern group: new onset (5.86%), paroxysmal (45.1%), persistent (29.1%) & long-standing persistent (18.5%)  Oral anticoagulation group: new onset (4.42%), paroxysmal (48.5%), persistent (28.7%) & long-standing persistent (17.3%) |  |  |  |  |  |  |  |
| Uhe et al, 2022^610^ | Germany | RCT | 196 | AFEQT | / | / | / | / | / | / |
|  |  |  | 64±11 years |  |  |  |  |  |  |  |
|  |  |  | First pulmonary vein isolation group (n=144): 37% & redo- pulmonary vein isolation group (n=52): 36% |  |  |  |  |  |  |  |
|  |  |  | NR |  |  |  |  |  |  |  |
|  |  |  | First pulmonary vein isolation group: paroxysmal (47%) & persistent (53%) Redo-pulmonary vein isolation group: paroxysmal (37%) & persistent (63%) |  |  |  |  |  |  |  |
| Ulla et al, 2015^611^ | Sweden | Methodological study | 147 with complete data (total n=270) | ASTA HRQoL scale & SF-36 | / | / | / | / | / | / |
|  |  |  | 58.9±12 years |  |  |  |  |  |  |  |
|  |  |  | 33% |  |  |  |  |  |  |  |
|  |  |  | NR |  |  |  |  |  |  |  |
|  |  |  | NR |  |  |  |  |  |  |  |
| Valderrábano et al, 2019^612^ | USA | RCT (protocol) | 405 participants to be enrolled | AFEQT | / | / | / | / | / | / |
|  |  |  | Participants aged 21-85 years |  |  |  |  |  |  |  |
|  |  |  | NR |  |  |  |  |  |  |  |
|  |  |  | NR |  |  |  |  |  |  |  |
|  |  |  | Persistent AF participants to be enrolled |  |  |  |  |  |  |  |
| van Breugel et al, 2016^613^ | NR | RCT | 125 | EQ-5D & SF-36 | / | / | / | / | / | / |
|  |  |  | 67.9±8.9 years |  |  |  |  |  |  |  |
|  |  |  | 36.8% |  |  |  |  |  |  |  |
|  |  |  | NR |  |  |  |  |  |  |  |
|  |  |  | Paroxysmal (43.2%), persistent (22.4%) & permanent (32.8%) |  |  |  |  |  |  |  |
| van Breugel et al, 2010^614^ | Netherlands | RCT | 132 | EQ-5D, EQ-VAS & SF-36 | / | / | / | / | / | / |
|  |  |  | 68.2±9.1 years |  |  |  |  |  |  |  |
|  |  |  | 35.6% |  |  |  |  |  |  |  |
|  |  |  | NR |  |  |  |  |  |  |  |
|  |  |  | Paroxysmal (43.2%), persistent (22.7%) & permanent (32.6%) |  |  |  |  |  |  |  |
| van Deelen et al, 2005^615^ | Netherlands | Cohort study | 152 | / | / | / | MMSE | / | / | / |
|  |  |  | International normalised ratio 2.0-3.4 >70% of treatment time group (n=106): 78.8±5.3 years & international normalised ratio 2.0-3.4 ≤70% of treatment time group (n=46): 79.5±5.3 years |  |  |  |  |  |  |  |
|  |  |  | International normalised ratio 2.0-3.4 >70% of treatment time group: 41.5% & international normalised ratio 2.0-3.4 ≤70% of treatment time group: 50% |  |  |  |  |  |  |  |
|  |  |  | NR |  |  |  |  |  |  |  |
|  |  |  | NR |  |  |  |  |  |  |  |
| van den Berg et al, 2001^616^ | Netherlands | Cohort study | 73 | SF-36 | / | / | / | / | / | / |
|  |  |  | 54.1±13.4 years |  |  |  |  |  |  |  |
|  |  |  | 30% |  |  |  |  |  |  |  |
|  |  |  | NR |  |  |  |  |  |  |  |
|  |  |  | Paroxysmal (all participants) |  |  |  |  |  |  |  |
| van den Berg et al, 2005^617^ | Netherlands | Cohort study | 73 | SF-36 | / | / | / | / | / | / |
|  |  |  | 55.5±13.3 years |  |  |  |  |  |  |  |
|  |  |  | 32% |  |  |  |  |  |  |  |
|  |  |  | NR |  |  |  |  |  |  |  |
|  |  |  | Paroxysmal (all participants) |  |  |  |  |  |  |  |
| van den Dries et al, 2017^618^ | Netherlands | RCT (protocol) | 1,000 participants to be enrolled | EQ-5D-5L & SF-12 | / | / | / | / | / | / |
|  |  |  | Participants aged ≥65 years to be enrolled |  |  |  |  |  |  |  |
|  |  |  | NR |  |  |  |  |  |  |  |
|  |  |  | NR |  |  |  |  |  |  |  |
|  |  |  | Paroxysmal AF participants to be enrolled |  |  |  |  |  |  |  |
| van der Heijden et al, 2023^619^ | Netherlands | RCT | 41 | EQ-5D-5L |  |  |  |  |  |  |
|  |  |  | Hybrid ablation group (n=19): 64±9 years & catheter ablation group (n=22): 64±8 years |  |  |  |  |  |  |  |
|  |  |  | Hybrid ablation group: 5% & catheter ablation group: 18% |  |  |  |  |  |  |  |
|  |  |  | NR |  |  |  |  |  |  |  |
|  |  |  | Hybrid ablation group: persistent (90%) & long-standing persistent (11%) Catheter ablation group: persistent (91%) & long-standing persistent (9%) |  |  |  |  |  |  |  |
| Van Gelder et al, 2006^620^ | Netherlands | RCT (protocol) | 500 participants to be enrolled | SF-36 | / | / | / | AFSS | / | / |
|  |  |  | Participants aged ≤80 years to be enrolled |  |  |  |  |  |  |  |
|  |  |  | NR |  |  |  |  |  |  |  |
|  |  |  | NR |  |  |  |  |  |  |  |
|  |  |  | Permanent AF participants to be enrolled |  |  |  |  |  |  |  |
| Veasey et al, 2010^621^ | UK | Cohort study | 25 | SF-36 | / | / | / | / | / | / |
|  |  |  | 63.7±9.4 years |  |  |  |  |  |  |  |
|  |  |  | 20% |  |  |  |  |  |  |  |
|  |  |  | NR |  |  |  |  |  |  |  |
|  |  |  | Paroxysmal (72%) & persistent (28%) |  |  |  |  |  |  |  |
| Verdicchio et al, 2023^622^ | Australia | RCT | 120 | / | / | / | / | AFSS | / | / |
|  |  |  | Men group (n=69): 63±11 years & women group (n=51): 68±7 years |  |  |  |  |  |  |  |
|  |  |  | 42.5% |  |  |  |  |  |  |  |
|  |  |  | NR |  |  |  |  |  |  |  |
|  |  |  | Men group: paroxysmal (52%) & persistent (48%)  Women group: paroxysmal (75%) & persistent (25%) |  |  |  |  |  |  |  |
| Verma et al, 2018^623^ | Australia, Belgium, Canada, China, Germany & UK | RCT (protocol) | 1,572 to be enrolled | EQ-5D-5L | / | / | / | / | / | / |
|  |  |  | Participants aged >18 years to be enrolled |  |  |  |  |  |  |  |
|  |  |  | NR |  |  |  |  |  |  |  |
|  |  |  | NR |  |  |  |  |  |  |  |
|  |  |  | Non-valvular AF participants to be enrolled |  |  |  |  |  |  |  |
| Verma et al, 2023^624^ | 9 countries (not specified) | Cohort study | 300 | AFEQT | / | / | / | / | / | / |
|  |  |  | Paroxysmal AF group: zero atrial arrhythmia burden group (n=102): 62.6±10.2 years & non-zero atrial arrhythmia burden group (n=45) 65.1±8.8 years  Persistent AF group: zero atrial arrhythmia burden group (n=89): 64.8±9.2 years & non-zero atrial arrhythmia burden group (n=54): 67.0±8.2 years |  |  |  |  |  |  |  |
|  |  |  | Paroxysmal AF group: zero atrial arrhythmia burden group: 35.8% & non-zero atrial arrhythmia burden group: 37.8%  Persistent AF group: zero atrial arrhythmia burden group: 22.5% & non-zero atrial arrhythmia burden group: 24.1% |  |  |  |  |  |  |  |
|  |  |  | NR |  |  |  |  |  |  |  |
|  |  |  | Paroxysmal (50%) & persistent (50%) |  |  |  |  |  |  |  |
| Vermeer et al, 2023^625^ | Netherlands | RCT (protocol) | 150 participants to be enrolled | AFEQT [Dutch] & EQ-5D [Dutch] | / | / | / | / | / | / |
|  |  |  | Participants aged 18-75 years to be enrolled |  |  |  |  |  |  |  |
|  |  |  | NR |  |  |  |  |  |  |  |
|  |  |  | NR |  |  |  |  |  |  |  |
|  |  |  | Paroxysmal or persistent AF participants to be enrolled |  |  |  |  |  |  |  |
| Vermeer et al, 2024^626^ | Netherlands | Cohort study | 20,725 | AFEQT | / | / | / | / | / | / |
|  |  |  | Median age reported: normal weight group (n=6,272): 64 (IQR 56-70) years, overweight group (n=9,658): 63 (IQR range 56-69) years & obese group (n=4,795): 62 (IQR 55-68) years |  |  |  |  |  |  |  |
|  |  |  | 33% |  |  |  |  |  |  |  |
|  |  |  | NR |  |  |  |  |  |  |  |
|  |  |  | Normal weight group: paroxysmal (77.2%), persistent (21.6%) & long-standing persistent (1.2%)  Overweight group: paroxysmal (71.7%), persistent (26.8%) & long-standing persistent (1.5%) Obese group: paroxysmal (64%), persistent (33.5%) & long-standing persistent (2.5%) |  |  |  |  |  |  |  |
| von Eisenhart Rothe et al, 2013^627^ | Germany | RCT | 334 | SF-12 & SF-8 | / | MDI | / | SCL | / | / |
|  |  |  | Age range reported: 18-55 years (n=79), 55-63 years (n=80), 63-69 years (n=86) & ≥69 years (n=89) |  |  |  |  |  |  |  |
|  |  |  | 41% |  |  |  |  |  |  |  |
|  |  |  | NR |  |  |  |  |  |  |  |
|  |  |  | Paroxysmal (all participants) |  |  |  |  |  |  |  |
| von Eisenhart Rothe et al, 2014^628^ | Germany | RCT | 702 | / | / | MDI | / | / | / | / |
|  |  |  | Median age reported: persistent AF group (n=392): 63.05 (IQR range 14) & paroxysmal group 65.26 (IQR range 12.9) |  |  |  |  |  |  |  |
|  |  |  | Persistent AF group: 29.6% & paroxysmal AF group: 39.4% |  |  |  |  |  |  |  |
|  |  |  | NR |  |  |  |  |  |  |  |
|  |  |  | Persistent (55.8%) & paroxysmal (44.2%) |  |  |  |  |  |  |  |
| Vroomen et al, 2019^629^ | Netherlands | RCT (protocol) | 40 participants to be enrolled | EQ-5D-5L | / | / | / | / | / | / |
|  |  |  | Participants aged >18 years to be enrolled |  |  |  |  |  |  |  |
|  |  |  | NR |  |  |  |  |  |  |  |
|  |  |  | NR |  |  |  |  |  |  |  |
|  |  |  | Persistent or long-standing persistent AF participants to be enrolled |  |  |  |  |  |  |  |
| von Oppell et al, 2009^630^ | UK | RCT | 49 | SF-36 | / | / | / | / | / | / |
|  |  |  | Cardioblate group (n=24): 66±8 years & control group (n=25): 68±9 years |  |  |  |  |  |  |  |
|  |  |  | 55.1% |  |  |  |  |  |  |  |
|  |  |  | NR |  |  |  |  |  |  |  |
|  |  |  | Permanent (89.8%) & persistent (10.2%) |  |  |  |  |  |  |  |
| Wagner et al, 2018^631^ | Denmark | RCT | 210 | AFEQT & SF-36 | / | HADS | / | / | / | / |
|  |  |  | Median age reported: men (n=151): 62.5 years & women (n=59): 59.25 years |  |  |  |  |  |  |  |
|  |  |  | 28% |  |  |  |  |  |  |  |
|  |  |  | NR |  |  |  |  |  |  |  |
|  |  |  | Men group: rehabilitation group (n=74): paroxysmal (70%) & persistent (30%), usual care group (n=77): paroxysmal (70%) & persistent (30%)  Women group: rehabilitation group (n=31): paroxysmal (77%) & persistent (23%), usual care group (n=28): paroxysmal (79%) & persistent (21%) |  |  |  |  |  |  |  |
| Wahlstrom et al, 2020^632^ | Sweden | RCT | 108 | ASTA HRQoL scale & SF-36 [Swedish] | / | / | / | ASTA symptom scale | / | / |
|  |  |  | Yoga group (n=38): 65±9 years, control group (n=41): 63±10 years & relaxation group (n=29): 64±15 years |  |  |  |  |  |  |  |
|  |  |  | Yoga group: 53%, control group: 51% & relaxation group: 48% |  |  |  |  |  |  |  |
|  |  |  | NR |  |  |  |  |  |  |  |
|  |  |  | Paroxysmal (all participants) |  |  |  |  |  |  |  |
| Wahlstrom et al, 2017^633^ | Sweden | Pilot RCT | 69 | SF-36 [Swedish] & EQ-5D | / | / | / | / | / | / |
|  |  |  | Yoga group (n=33): 64±7 years & control group (n=36): 63±8 years |  |  |  |  |  |  |  |
|  |  |  | Yoga group: 52% & control group: 28% |  |  |  |  |  |  |  |
|  |  |  | NR |  |  |  |  |  |  |  |
|  |  |  | Paroxysmal (all participants) |  |  |  |  |  |  |  |
| Walfridsson et al, 2015^634^ | Sweden | RCT | 294 | SF-36 & EQ-5D | / | / | / | ASTA symptom scale | / | / |
|  |  |  | 55±10 years |  |  |  |  |  |  |  |
|  |  |  | 30% |  |  |  |  |  |  |  |
|  |  |  | NR |  |  |  |  |  |  |  |
|  |  |  | Paroxysmal (all participants) |  |  |  |  |  |  |  |
| Walfridsson et al, 2020^635^ | Sweden | Pilot RCT | 15 | ASTA HRQoL scale, EQ-5D-5L & SF-36 | / | HADS | / | ASTA symptom scale | / | / |
|  |  |  | 64.7 years |  |  |  |  |  |  |  |
|  |  |  | 26.7% |  |  |  |  |  |  |  |
|  |  |  | NR |  |  |  |  |  |  |  |
|  |  |  | Paroxysmal (60%), persistent (26.7%) & permanent (13.3%) |  |  |  |  |  |  |  |
| Walfridsson et al, 2022^636^ | Sweden | Cohort study | 1,521 | ASTA HRQoL scale | / | / | / | ASTA symptom scale | / | / |
|  |  |  | 62±10.18 years |  |  |  |  |  |  |  |
|  |  |  | 31% |  |  |  |  |  |  |  |
|  |  |  | NR |  |  |  |  |  |  |  |
|  |  |  | Paroxysmal (39%), persistent (51%) & long-standing persistent (2%) |  |  |  |  |  |  |  |
| Walfridsson et al, 2019^637^ | Denmark & Sweden | Cross-sectional study | 2,493 | ASTA HRQoL scale | / | / | / | ASTA symptom scale | / | / |
|  |  |  | Female group (n=698): 64.5±8.6 years, male group (n=1,795): 59.6±9.7 years |  |  |  |  |  |  |  |
|  |  |  | 28% |  |  |  |  |  |  |  |
|  |  |  | NR |  |  |  |  |  |  |  |
|  |  |  | Female group: paroxysmal (49%), persistent (49%) & long-standing persistent (2%) Male group: paroxysmal (48%), persistent (49%) & long-standing persistent (3%) |  |  |  |  |  |  |  |
| Walfridsson et al, 2022 [a] ^638^ | Australia | Methodological study | 179 with AF (total n=202) | ASTA HRQoL scale | / | / | / | ASTA symptom scale | / | / |
|  |  |  | 67.9±12.2 years |  |  |  |  |  |  |  |
|  |  |  | 30.2% |  |  |  |  |  |  |  |
|  |  |  | NR |  |  |  |  |  |  |  |
|  |  |  | NR |  |  |  |  |  |  |  |
| Walfridsson et al, 2012^639^ | Sweden | Methodological study | 126 with AF (total n=270) | SF-36 | / | / | / | ASTA symptom scale & SCL | / | / |
|  |  |  | 60.3±9.3 years |  |  |  |  |  |  |  |
|  |  |  | 26% |  |  |  |  |  |  |  |
|  |  |  | NR |  |  |  |  |  |  |  |
|  |  |  | NR |  |  |  |  |  |  |  |
| Walters et al, 2018^640^ | Australia | Cohort study | 78 | SF-36 | / | HADS | / | AFSS | / | / |
|  |  |  | 59±10 years |  |  |  |  |  |  |  |
|  |  |  | 26% |  |  |  |  |  |  |  |
|  |  |  | NR |  |  |  |  |  |  |  |
|  |  |  | Paroxysmal (74%) & persistent (25%) |  |  |  |  |  |  |  |
| Walters et al, 2019^641^ | Australia | Cohort study | 78 | SF-36 | / | PSS-10 | / | AFSS | / | / |
|  |  |  | 60±9 years |  |  |  |  |  |  |  |
|  |  |  | 23% |  |  |  |  |  |  |  |
|  |  |  | NR |  |  |  |  |  |  |  |
|  |  |  | Paroxysmal (74%) |  |  |  |  |  |  |  |
| Wang et al, 2016^642^ | China | Cohort study | 99 | SF-36 | / | / | / | / | / | / |
|  |  |  | Atrial tachycardia recurrence group (n=49): 63.9±9.6 years & AF recurrence group (n=50): 60.9±9.3 years |  |  |  |  |  |  |  |
|  |  |  | Atrial tachycardia recurrence group: 32.7% & AF recurrence group: 34% |  |  |  |  |  |  |  |
|  |  |  | NR |  |  |  |  |  |  |  |
|  |  |  | Long-standing persistent (all participants) |  |  |  |  |  |  |  |
| Wang et al, 2020^643^ | USA | Cohort study | 1,244 | / | / | PHQ-9 | MoCA | / | / | / |
|  |  |  | 75.8±6.9 years |  |  |  |  |  |  |  |
|  |  |  | 49% |  |  |  |  |  |  |  |
|  |  |  | Non-Hispanic White (84.9%) |  |  |  |  |  |  |  |
|  |  |  | NR |  |  |  |  |  |  |  |
| Wang et al, 2020 [a] ^644^ | USA | Cohort study | 1,244 | / | / | / | MoCA | / | / | / |
|  |  |  | 75.5±7.1 years |  |  |  |  |  |  |  |
|  |  |  | 48.8% |  |  |  |  |  |  |  |
|  |  |  | White (84.9%) |  |  |  |  |  |  |  |
|  |  |  | NR |  |  |  |  |  |  |  |
| Wang et al, 2021^645^ | USA | Cohort study | 1,137 | / | / | PHQ-9 | MoCA | / | / | / |
|  |  |  | 75.3±7 years |  |  |  |  |  |  |  |
|  |  |  | 47.9% |  |  |  |  |  |  |  |
|  |  |  | Non-Hispanic White (85.9%) |  |  |  |  |  |  |  |
|  |  |  | NR |  |  |  |  |  |  |  |
| Wang X et al, 2021^646^ | China | Cohort study | 139 | / | / | / | TICS-m | / | / | / |
|  |  |  | 60.90±8.51 years |  |  |  |  |  |  |  |
|  |  |  | 30.2% |  |  |  |  |  |  |  |
|  |  |  | NR |  |  |  |  |  |  |  |
|  |  |  | Non-valvular (all participants) |  |  |  |  |  |  |  |
| Wang et al, 2022^647^ | China | Cohort study | 112 | AFEQT | / | / | / | / | / | / |
|  |  |  | Age range reported: After propensity score matching – dronedarone group (n=56): >65 years (27%) & metoprolol and dronedarone group (n=56) >65 years (42%) |  |  |  |  |  |  |  |
|  |  |  | Dronedarone group: 41% & metoprolol and dronedarone group: 36% |  |  |  |  |  |  |  |
|  |  |  | NR |  |  |  |  |  |  |  |
|  |  |  | Paroxysmal (all participants) |  |  |  |  |  |  |  |
| Wang et al, 2023^648^ | USA | Cohort study | 1,244 | / | / | / | MoCA | / | / | / |
|  |  |  | 75.5±7.1 years |  |  |  |  |  |  |  |
|  |  |  | 49% |  |  |  |  |  |  |  |
|  |  |  | Non-Hispanic White (84.9%) |  |  |  |  |  |  |  |
|  |  |  | Anticoagulant use no group (n=180): paroxysmal (78%), persistent or long-standing persistent (7%) & permanent (2%) Anticoagulation use yes group (1,064): paroxysmal (56%), persistent or long-standing persistent (28%) & permanent (7%) |  |  |  |  |  |  |  |
| Wang et al, 2024^649^ | China | Cross-sectional study | 202 | AFEQT | / | / | / | / | / | / |
|  |  |  | 58.28±12.70 years |  |  |  |  |  |  |  |
|  |  |  | 45% |  |  |  |  |  |  |  |
|  |  |  | NR |  |  |  |  |  |  |  |
|  |  |  | Paroxysmal (52.48%), persistent (33.17%) & long-standing persistent (33.17%) |  |  |  |  |  |  |  |
| Ward et al, 2001^650^ | UK | RCT | 18 | / | / | / | / | / | Specific activity scale | / |
|  |  |  | 62.7 years |  |  |  |  |  |  |  |
|  |  |  | 33.3% |  |  |  |  |  |  |  |
|  |  |  | NR |  |  |  |  |  |  |  |
|  |  |  | Paroxysmal (all participants) |  |  |  |  |  |  |  |
| Washida et al, 2017^651^ | Japan | Cohort study | 62 | / | / | / | MMSE [Japanese] & MoCA [Japanese] | / | / | / |
|  |  |  | 76.3±9.0 years |  |  |  |  |  |  |  |
|  |  |  | 32.3% |  |  |  |  |  |  |  |
|  |  |  | NR |  |  |  |  |  |  |  |
|  |  |  | Non-valvular (all participants) |  |  |  |  |  |  |  |
| Wazni et al, 2005^652^ | USA Italy & Germany | Pilot RCT | 70 | SF-36 | / | / | / | / | / | / |
|  |  |  | Pulmonary vein isolation group (n=33): 55±8 years & antiarrhythmic drug group (n=37): 54±8 years |  |  |  |  |  |  |  |
|  |  |  | NR |  |  |  |  |  |  |  |
|  |  |  | NR |  |  |  |  |  |  |  |
|  |  |  | Pulmonary vein isolation group: paroxysmal (97%) & persistent (3%) Antiarrhythmic drug group: paroxysmal (95%) & persistent (5%) |  |  |  |  |  |  |  |
| Wazni et al, 2022^653^ | USA, | RCT | 203 | AFEQT & EQ-5D | / | / | / | / | / | / |
|  |  |  | Cryoballoon catheter ablation group (n=104): 60.4±11.2 years & antiarrhythmic drug group (n=99): 61.6±11.2 years |  |  |  |  |  |  |  |
|  |  |  | Cryoballoon catheter ablation group: 39.4% & antiarrhythmic drug group: 42.4% |  |  |  |  |  |  |  |
|  |  |  | NR |  |  |  |  |  |  |  |
|  |  |  | Paroxysmal (all participants) |  |  |  |  |  |  |  |
| Weerasooriya et al, 2003^654^ | Australia | RCT | 99 | AQoL & CAST-QoL | / | / | / | / | / | / |
|  |  |  | 68±8.7 years |  |  |  |  |  |  |  |
|  |  |  | 29% |  |  |  |  |  |  |  |
|  |  |  | NR |  |  |  |  |  |  |  |
|  |  |  | Permanent (all participants) |  |  |  |  |  |  |  |
| Weerasooriya et al, 2005^655^ | Australia | Cohort study | 63 | SF-36 | / | / | / | SCL | / | / |
|  |  |  | 56±7 years |  |  |  |  |  |  |  |
|  |  |  | 22.2% |  |  |  |  |  |  |  |
|  |  |  | NR |  |  |  |  |  |  |  |
|  |  |  | Paroxysmal (all participants) |  |  |  |  |  |  |  |
| Wei et al, 2024^656^ | NR | RCT | 156 | SF-36 | / | SAS & SDS | / | / | / | / |
|  |  |  | Control group (n=78): 57.50±7.66 years & study group (n=78): 57.56±7.71 years |  |  |  |  |  |  |  |
|  |  |  | Control group: 41% & study group: 43.6% |  |  |  |  |  |  |  |
|  |  |  | NR |  |  |  |  |  |  |  |
|  |  |  | NR |  |  |  |  |  |  |  |
| Wettervik et al, 2025^657^ | Finland & Sweden | RCT | 149 | EQ-5D-3L & SF-36 | / | / | / | / | / | / |
|  |  |  | Median age reported: 58 (IQR range 50-64) years |  |  |  |  |  |  |  |
|  |  |  | 22.8% |  |  |  |  |  |  |  |
|  |  |  | NR |  |  |  |  |  |  |  |
|  |  |  | Paroxysmal (70.5%) |  |  |  |  |  |  |  |
| Wieczorek et al, 2016^658^ | Poland | Cohort study | 74 | / | / | / | MMSE | / | / | / |
|  |  |  | Median age reported: 59 (IQR range 55-63) years |  |  |  |  |  |  |  |
|  |  |  | 39.2% |  |  |  |  |  |  |  |
|  |  |  | NR |  |  |  |  |  |  |  |
|  |  |  | Paroxysmal (all participants) |  |  |  |  |  |  |  |
| Wijtvliet et al, 2020^659^ | Netherlands | RCT | 1,354 | / | / | / | / | AFSS | / | / |
|  |  |  | 64±10 years |  |  |  |  |  |  |  |
|  |  |  | 34% |  |  |  |  |  |  |  |
|  |  |  | NR |  |  |  |  |  |  |  |
|  |  |  | Nurse-led care group (n=671): paroxysmal (61%) & non-paroxysmal (25%) Usual-care group (n=683): paroxysmal (63%) & non-paroxysmal (20%) |  |  |  |  |  |  |  |
| Willits et al, 2021^660^ | UK | Cohort study (registry) | 537 | EQ-5D-5L | / | / | / | / | / | / |
|  |  |  | Median age reported: 75 (IQR range 70-80) years |  |  |  |  |  |  |  |
|  |  |  | 31.2% |  |  |  |  |  |  |  |
|  |  |  | NR |  |  |  |  |  |  |  |
|  |  |  | Paroxysmal (27.7%), persistent (16.6%) & permanent (54.4%) |  |  |  |  |  |  |  |
| William et al, 2025^661^ | Australia, Canada & UK | RCT | 338 | AFEQT | / | / | / | / | / | / |
|  |  |  | 64.3±9.4 years |  |  |  |  |  |  |  |
|  |  |  | 23% |  |  |  |  |  |  |  |
|  |  |  | NR |  |  |  |  |  |  |  |
|  |  |  | Persistent (all participants) |  |  |  |  |  |  |  |
| Wilson et al, 2021^662^ | NR | Cohort study | 26 | / | / | / | / | SCL | / | / |
|  |  |  | 67.1 years |  |  |  |  |  |  |  |
|  |  |  | 50% |  |  |  |  |  |  |  |
|  |  |  | White (100%) |  |  |  |  |  |  |  |
|  |  |  | NR |  |  |  |  |  |  |  |
| Wilton et al, 2011^663^ | NR | RCT | 19 with AF (total n=86) | SF-36 | / | / | / | / | Specific activity scale | / |
|  |  |  | 68±7 years |  |  |  |  |  |  |  |
|  |  |  | 11% |  |  |  |  |  |  |  |
|  |  |  | NR |  |  |  |  |  |  |  |
|  |  |  | Persistent (all participants) |  |  |  |  |  |  |  |
| Wokhlu et al, 2010^664^ | USA | Cohort study | 502 | SF-36 | / | / | / | MAFSI | / | / |
|  |  |  | 55.9±10.3 years |  |  |  |  |  |  |  |
|  |  |  | 18% |  |  |  |  |  |  |  |
|  |  |  | NR |  |  |  |  |  |  |  |
|  |  |  | Paroxysmal (51%), persistent (35%) & long-standing persistent (13%) |  |  |  |  |  |  |  |
| Wood et al, 2024^665^ | USA | Cohort study | 446 | SF-12 | / | CES-D | / | / | / | / |
|  |  |  | 78.1±5.2 years |  |  |  |  |  |  |  |
|  |  |  | 44% |  |  |  |  |  |  |  |
|  |  |  | White (88%) & Black (12%) |  |  |  |  |  |  |  |
|  |  |  | NR |  |  |  |  |  |  |  |
| Wood et al, 2020^666^ | USA | Pilot cohort study | 20 | PPAQ | / | POMS | / | / | / | / |
|  |  |  | 65±7 years |  |  |  |  |  |  |  |
|  |  |  | 55% |  |  |  |  |  |  |  |
|  |  |  | Caucasian (90%) & Asian (10%) |  |  |  |  |  |  |  |
|  |  |  | Paroxysmal (35%) & persistent (65%) |  |  |  |  |  |  |  |
| Wood et al, 2017^667^ | USA | Pilot cohort study | 20 | PPAQ | / | POMS & PHQ-9 | / | / | / | / |
|  |  |  | 65±7 years |  |  |  |  |  |  |  |
|  |  |  | 55% |  |  |  |  |  |  |  |
|  |  |  | Caucasian (90%) & Asian (10%) |  |  |  |  |  |  |  |
|  |  |  | Paroxysmal (35%) & persistent (65%) |  |  |  |  |  |  |  |
| Wood et al, 2009^668^ | USA | Methodological study | 103 | PPAQ | / | / | / | SCL | / | / |
|  |  |  | 49+18 years |  |  |  |  |  |  |  |
|  |  |  | 53% |  |  |  |  |  |  |  |
|  |  |  | NR |  |  |  |  |  |  |  |
|  |  |  | NR |  |  |  |  |  |  |  |
| Woo et al, 2022^669^ | Singapore | Cohort study | 43 | AFEQT [Simplified Chinese & English] | / | PHQ-9 [Simplified Chinese & English] | / | / | / | / |
|  |  |  | Median age reported: 69 (IQR range 64–74) years |  |  |  |  |  |  |  |
|  |  |  | 33% |  |  |  |  |  |  |  |
|  |  |  | Chinese (88%) & Malay (12%) |  |  |  |  |  |  |  |
|  |  |  | Chronic (all participants) |  |  |  |  |  |  |  |
| Worck et al, 2023^670^ | Denmark | Cohort study | 24 | AFEQT | / | / | / | / | / | / |
|  |  |  | Median age reported: 64 (IQR range 50-75) years |  |  |  |  |  |  |  |
|  |  |  | 17% |  |  |  |  |  |  |  |
|  |  |  | NR |  |  |  |  |  |  |  |
|  |  |  | Persistent (all participants) |  |  |  |  |  |  |  |
| Wozniak-Skowerska et al, 2016^671^ | Poland | Cohort study | 33 | SF-36 | / | / | / | / | / | / |
|  |  |  | 54.2±9 years |  |  |  |  |  |  |  |
|  |  |  | 21.2% |  |  |  |  |  |  |  |
|  |  |  | NR |  |  |  |  |  |  |  |
|  |  |  | Paroxysmal (all participants) |  |  |  |  |  |  |  |
| Wu et al, 2021^672^ | China | RCT | 648 | SF-36 | / | / | / | / | / | / |
|  |  |  | 64.1±11.3 years |  |  |  |  |  |  |  |
|  |  |  | 35% |  |  |  |  |  |  |  |
|  |  |  | NR |  |  |  |  |  |  |  |
|  |  |  | Persistent (41.2%) & long-standing persistent (58.8%) |  |  |  |  |  |  |  |
| Wu et al, 2016^673^ | NR | Cohort study | 198 | SF-36 [Dutch] | / | / | / | / | / | / |
|  |  |  | Asymptomatic group (n=66): 52.0±9.0 years & symptomatic group (n=132): 53.1±8.6 years |  |  |  |  |  |  |  |
|  |  |  | Asymptomatic group: 10.6% & symptomatic group: 15.9% |  |  |  |  |  |  |  |
|  |  |  | NR |  |  |  |  |  |  |  |
|  |  |  | Persistent (all participants) |  |  |  |  |  |  |  |
| Wu et al, 2022^674^ | China | RCT | 63 | SF-36 | / | / | / | / | IPAQ | / |
|  |  |  | Usual care group (n=33): 64±7 years & cardiac rehabilitation group (n=30): 66±9 years |  |  |  |  |  |  |  |
|  |  |  | Usual care group: 30% & cardiac rehabilitation group: 37% |  |  |  |  |  |  |  |
|  |  |  | NR |  |  |  |  |  |  |  |
|  |  |  | Usual care group: paroxysmal (9%) & persistent (91%) Cardiac rehabilitation group: paroxysmal (7%) & persistent (93%) |  |  |  |  |  |  |  |
| Wynn et al, 2015^675^ | NR | RCT | 146 | AF-QoL | / | / | / | / | / | / |
|  |  |  | 55±9 years |  |  |  |  |  |  |  |
|  |  |  | Ablation group (n=98): 22.5% & drug therapy group (n=48): 23% |  |  |  |  |  |  |  |
|  |  |  | NR |  |  |  |  |  |  |  |
|  |  |  | Persistent (all participants) |  |  |  |  |  |  |  |
| Wynn et al, 2014^676^ | UK | Methodological study | 362 | AFEQT & EQ-5D-3L | / | / | / | / | / | / |
|  |  |  | 60±12 years |  |  |  |  |  |  |  |
|  |  |  | 33.1% |  |  |  |  |  |  |  |
|  |  |  | NR |  |  |  |  |  |  |  |
|  |  |  | Paroxysmal (56.4%) & non-paroxysmal (43.6%) |  |  |  |  |  |  |  |
| Wynn et al, 2016^677^ | UK | RCT | 130 | AFEQT & SF-36 | / | / | / | / | / | / |
|  |  |  | 61.9±10.5 years |  |  |  |  |  |  |  |
|  |  |  | 32% |  |  |  |  |  |  |  |
|  |  |  | NR |  |  |  |  |  |  |  |
|  |  |  | Persistent (61%) & paroxysmal (39%) |  |  |  |  |  |  |  |
| Xiao et al, 2021^678^ | China | RCT (protocol) | 76 participants to be enrolled | SF-36 | / | HAM-A & HAM-D | / | / | / | / |
|  |  |  | Participants aged 18-85 years to be enrolled |  |  |  |  |  |  |  |
|  |  |  | NR |  |  |  |  |  |  |  |
|  |  |  | NR |  |  |  |  |  |  |  |
|  |  |  | NR |  |  |  |  |  |  |  |
| Yagishita et al, 2017^679^ | Japan | Cohort study | 34 | SF-36 & AFQLQ | / | / | / | / | / | / |
|  |  |  | 60.4±9.2 years |  |  |  |  |  |  |  |
|  |  |  | 21% |  |  |  |  |  |  |  |
|  |  |  | NR |  |  |  |  |  |  |  |
|  |  |  | Persistent (all participants) |  |  |  |  |  |  |  |
| Yamamoto et al, 2014^680^ | Japan | RCT | 233 | AFQLQ | / | / | / | / | / | / |
|  |  |  | 64.9±9.7 years |  |  |  |  |  |  |  |
|  |  |  | 29% |  |  |  |  |  |  |  |
|  |  |  | NR |  |  |  |  |  |  |  |
|  |  |  | Paroxysmal (all participants) |  |  |  |  |  |  |  |
| Yamashita et al, 2003^681^ | Japan | Methodological study | 205 | AFQLQ | / | / | / | / | / | / |
|  |  |  | NR |  |  |  |  |  |  |  |
|  |  |  | NR |  |  |  |  |  |  |  |
|  |  |  | NR |  |  |  |  |  |  |  |
|  |  |  | Paroxysmal (50.2%) & chronic (49.8%) |  |  |  |  |  |  |  |
| Yamashita et al, 2005^682^ | Japan | Methodological study | 240 | AFQLQ | / | / | / | / | / | / |
|  |  |  | Paroxysmal (n=133): 64±9 years & chronic AF group (n=107): 67±9 years |  |  |  |  |  |  |  |
|  |  |  | 27.5% |  |  |  |  |  |  |  |
|  |  |  | NR |  |  |  |  |  |  |  |
|  |  |  | Paroxysmal (55.4%) & chronic (44.6%) |  |  |  |  |  |  |  |
| Yamashita et al, 2011^683^ | Japan | RCT | 318 | AFQLQ | / | / | / | / | / | / |
|  |  |  | Candesartan group (n=158): 66±9.7 years & amlodipine group (n=160): 65.1±9.3 years |  |  |  |  |  |  |  |
|  |  |  | 31% |  |  |  |  |  |  |  |
|  |  |  | NR |  |  |  |  |  |  |  |
|  |  |  | Paroxysmal (all participants) |  |  |  |  |  |  |  |
| Yamashita et al, 2023^684^ | Japan | Cohort study (registry) | 3,318 | AFEQT | / | / | / | / | / | / |
|  |  |  | 67.8±11.6 years |  |  |  |  |  |  |  |
|  |  |  | 31.6% |  |  |  |  |  |  |  |
|  |  |  | NR |  |  |  |  |  |  |  |
|  |  |  | Persistent (44.4%) |  |  |  |  |  |  |  |
| Yamin et al, 2023^685^ | Indonesia | Methodological study | 60 | SF-36 [Indonesian] | / | / | / | AFSS [Indonesian] | / | / |
|  |  |  | 58.8 years |  |  |  |  |  |  |  |
|  |  |  | 46.7% |  |  |  |  |  |  |  |
|  |  |  | NR |  |  |  |  |  |  |  |
|  |  |  | Paroxysmal (18.3%), persistent (15%), long-standing persistent (3.3%) & permanent (63.3%) |  |  |  |  |  |  |  |
| Yamin et al, 2023 [a] ^686^ | Indonesia | Methodological study | 60 | ASTA [Indonesian] & SF-36 [Indonesian] | / | / | / | / | / | / |
|  |  |  | Age range reported: ≤40 years (n=5), 41-50 years (n=8), 51-60 years (n=17), 61-70 years (n=19) & >70 years (n=11) |  |  |  |  |  |  |  |
|  |  |  | 46.67% |  |  |  |  |  |  |  |
|  |  |  | NR |  |  |  |  |  |  |  |
|  |  |  | Paroxysmal (18.3%), persistent (15%), long-standing persistent (3.3%) & permanent (60%) |  |  |  |  |  |  |  |
| Yan et al, 2022^687^ | China | RCT | 235 | SF-36 [Chinese] | / | / | / | / | / | / |
|  |  |  | 65.2±10.9 years |  |  |  |  |  |  |  |
|  |  |  | 38.7% |  |  |  |  |  |  |  |
|  |  |  | NR |  |  |  |  |  |  |  |
|  |  |  | NR |  |  |  |  |  |  |  |
| Yan et al, 2024^688^ | China | Cross-sectional study | 531 | AFEQT | / | GAD-7 & PHQ-9 | / | / | / | / |
|  |  |  | 67.12±10.93 years |  |  |  |  |  |  |  |
|  |  |  | 49.7% |  |  |  |  |  |  |  |
|  |  |  | NR |  |  |  |  |  |  |  |
|  |  |  | Paroxysmal (67.23%) & persistent (32.77%) |  |  |  |  |  |  |  |
| Yanagawa et al, 2013^689^ | NR | Cohort study | 250 | SF-12 | / | / | / | SCL | / | / |
|  |  |  | 64.3±12.1 years |  |  |  |  |  |  |  |
|  |  |  | 41.2% |  |  |  |  |  |  |  |
|  |  |  | NR |  |  |  |  |  |  |  |
|  |  |  | Paroxysmal (14.4%), long-standing persistent (42%) & persistent (43.6%) |  |  |  |  |  |  |  |
| Yang et al, 2023^690^ | China | RCT | 23 | SF-36 | / | / | / | / | / | / |
|  |  |  | 68.5±7.7 years |  |  |  |  |  |  |  |
|  |  |  | 39% |  |  |  |  |  |  |  |
|  |  |  | NR |  |  |  |  |  |  |  |
|  |  |  | Permanent (all participants) |  |  |  |  |  |  |  |
| Yao et al, 2020^691^ | Canada | RCT | 346 | AFEQT & EQ-5D | / | / | / | / | / | / |
|  |  |  | Male group (n=231): 57.7±10.3 years & female group (n=115): 60.9±9.1 years |  |  |  |  |  |  |  |
|  |  |  | 33% |  |  |  |  |  |  |  |
|  |  |  | NR |  |  |  |  |  |  |  |
|  |  |  | Paroxysmal (all participants) |  |  |  |  |  |  |  |
| Yoshihisa et al, 2022^692^ | Japan | Case-control study | 32 with AF (total n=61) | / | / | CES-D | MMSE | / | / | / |
|  |  |  | 68.5±12.3 years |  |  |  |  |  |  |  |
|  |  |  | 34.4% |  |  |  |  |  |  |  |
|  |  |  | NR |  |  |  |  |  |  |  |
|  |  |  | Paroxysmal (56.2%) & persistent (43.8%) |  |  |  |  |  |  |  |
| Younes et al, 2024^693^ | NR | RCT | 815 | SF-36 |  |  |  |  |  |  |
|  |  |  | Median age reported: 62 (IQR range 9.1) |  |  |  |  |  |  |  |
|  |  |  | 21% |  |  |  |  |  |  |  |
|  |  |  | NR |  |  |  |  |  |  |  |
|  |  |  | Persistent (all participants) |  |  |  |  |  |  |  |
| Yu et al, 2012^694^ | China | Cohort study | 199 | SF-36 | / | SAS & SDS | / | / | / | / |
|  |  |  | Circumferential pulmonary vein ablation group (n=97): 55.17±6.45 years & medicine group (n=102): 54.86±7.02 years |  |  |  |  |  |  |  |
|  |  |  | Circumferential pulmonary vein ablation group: 50% & medicine group: 48% |  |  |  |  |  |  |  |
|  |  |  | NR |  |  |  |  |  |  |  |
|  |  |  | Paroxysmal (all participants) |  |  |  |  |  |  |  |
| Zadeh et al, 2019^695^ | Iran | RCT | 50 | AFEQT | / | / | / | / | / | / |
|  |  |  | Age categories reported: ≤64 years (n=72) & >65 years (n=8) |  |  |  |  |  |  |  |
|  |  |  | 56% |  |  |  |  |  |  |  |
|  |  |  | NR |  |  |  |  |  |  |  |
|  |  |  | NR |  |  |  |  |  |  |  |
| Zeitler et al, 2023^696^ | NR | RCT | 2,204 | AFEQT | / | / | / | MAFSI | / | / |
|  |  |  | Median age reported: men group (n=1,385): 66.5 (IQR range 60.1-70.9) years & women group (n=819): 69.4 (IQR range 65.1-73.7) years |  |  |  |  |  |  |  |
|  |  |  | 37.2% |  |  |  |  |  |  |  |
|  |  |  | Men group: White (91%), Black or African American (4%) & Other (5.1%) Women group: White (93.9%), Black or African American (2.7%) & Other (3.4%) |  |  |  |  |  |  |  |
|  |  |  | Men group: paroxysmal (39%), persistent (49.8%), long-standing persistent (11.2%)  Women group: paroxysmal (49.6%), persistent (43%), long-standing persistent (7.3%) |  |  |  |  |  |  |  |
| Zenger et al, 2020^697^ | USA | Cohort study | 1,338 | / | / | / | / | AFSS | / | / |
|  |  |  | 67.3±12.4 years |  |  |  |  |  |  |  |
|  |  |  | 37% |  |  |  |  |  |  |  |
|  |  |  | American Indian and Alaskan Native (1%), Asian (2%), Black or African American (1%), Native Hawaiian or Other Pacific Islander (1%), Other (3%) & White or Caucasian (93%) |  |  |  |  |  |  |  |
|  |  |  | NR |  |  |  |  |  |  |  |
| Zenger et al, 2024^698^ | USA | Cohort study | 308 | / | / | PROMIS - Bank Depression | / | / | / | / |
|  |  |  | Non-same day discharge group (n=150): 66.05±10.34 years & same day discharge group (n=158): 64.83±10.97 years |  |  |  |  |  |  |  |
|  |  |  | Non-same day discharge group: 27.33% & same day discharge group: 25.95% |  |  |  |  |  |  |  |
|  |  |  | Non-same day discharge group: American Indian and Alaska Native (0.67%), Black or African American (0.67%), did not disclose (1.33%), Native Hawaiian and Other Pacific Islander (0.67%), Other (2.67%) & White or Caucasian (94%)  Same day discharge group: American Indian and Alaska Native (0.63%), Black or African American (0%), did not disclose (0.63%), Native Hawaiian and Other Pacific Islander (1.27%), Other (1.27%) & White or Caucasian (96.2%) |  |  |  |  |  |  |  |
|  |  |  | Non-same day discharge group: paroxysmal (35.33%), persistent (60%), permanent (4%) & unable to determine (0.67%) Same day discharge group: paroxysmal (56.96%), persistent (41.77%), permanent (0.63%) & unable to determine (0.63%) |  |  |  |  |  |  |  |
| Zeren et al, 2021^699^ | Turkey | Methodological study | 98 | AFImpact [Turkish] & SF-36 [Turkish] | / | / | / | / | / | / |
|  |  |  | 64.84±6.57 years |  |  |  |  |  |  |  |
|  |  |  | 52% |  |  |  |  |  |  |  |
|  |  |  | NR |  |  |  |  |  |  |  |
|  |  |  | Paroxysmal (19%), persistent (21%) & permanent (60%) |  |  |  |  |  |  |  |
| Zeren et al, 2022^700^ | Turkey | Cross-sectional study | 125 | SF-36 | / | / | / | / | IPAQ-SF | / |
|  |  |  | 65.02±6.84 years |  |  |  |  |  |  |  |
|  |  |  | 49% |  |  |  |  |  |  |  |
|  |  |  | NR |  |  |  |  |  |  |  |
|  |  |  | Paroxysmal (17%), persistent (22%) & permanent (61%) |  |  |  |  |  |  |  |
| Zhang et al, 2014^701^ | China | RCT | 201 | SF-36 | / | / | / | / | / | / |
|  |  |  | 59.9+10.9 years |  |  |  |  |  |  |  |
|  |  |  | 31.3% |  |  |  |  |  |  |  |
|  |  |  | NR |  |  |  |  |  |  |  |
|  |  |  | Persistent (all participants) |  |  |  |  |  |  |  |
| Zhang et al, 2023^702^ | China | Cohort study | 197 | / | / | / | / | AF6 | / | / |
|  |  |  | 74.1±9.0 years |  |  |  |  |  |  |  |
|  |  |  | 53.8% |  |  |  |  |  |  |  |
|  |  |  | NR |  |  |  |  |  |  |  |
|  |  |  | Non-valvular (all participants) |  |  |  |  |  |  |  |
| Zhang et al, 2022^703^ | China | RCT (protocol) | 100 participants to be enrolled | AFEQT [Chinese] | / | / | / | / | / | / |
|  |  |  | Participants aged 18-75 years to be enrolled |  |  |  |  |  |  |  |
|  |  |  | NR |  |  |  |  |  |  |  |
|  |  |  | NR |  |  |  |  |  |  |  |
|  |  |  | Paroxysmal AF participants to be enrolled |  |  |  |  |  |  |  |
| Zito et al, 1996^704^ | Italy | Cohort study | 38 | / | BADL & IADL | GDS | MMSE | / | / | / |
|  |  |  | 80.6±4.5 years |  |  |  |  |  |  |  |
|  |  |  | 60.5% |  |  |  |  |  |  |  |
|  |  |  | NR |  |  |  |  |  |  |  |
|  |  |  | Chronic (all participants) |  |  |  |  |  |  |  |
| Zucchelli et al, 2023^705^ | Argentina,  Austria,  Belgium,  Colombia,  Czech Republic,  Germany,  Germany,  Hungary,  Iceland,  Italy,  Japan,  Kuwait,  Malaysia,  Mexico,  Poland,  Portugal,  Saudi Arabia &  Serbia,  Singapore,  Slovakia,  South Africa  Spain,  Taiwan,  Thailand,  UK, | Cohort study | 1,394 | EQ-5D-5L | / | / | / | / | / | / |
|  |  |  | First-line group (n=433): 60±13 years & antiarrhythmic drug group (n=961): 61±11 years |  |  |  |  |  |  |  |
|  |  |  | First-line group: 35.1% & antiarrhythmic drug group: 36.8% |  |  |  |  |  |  |  |
|  |  |  | NR |  |  |  |  |  |  |  |
|  |  |  | First-line group: paroxysmal (87.3%) antiarrhythmic drug group: paroxysmal (80.2%) |  |  |  |  |  |  |  |
| Zulmiyusrini et al, 2023^706^ | Indonesia | Methodological study | 102 | AFEQT [Indonesian] & SF-36 [Indonesian] | / | / | / | / | / | / |
|  |  |  | 58.98±11.82 years |  |  |  |  |  |  |  |
|  |  |  | 48% |  |  |  |  |  |  |  |
|  |  |  | NR |  |  |  |  |  |  |  |
|  |  |  | Paroxysmal (27.4%), persistent (15.7%), long-standing persistent (5.9%) & permanent (51%) |  |  |  |  |  |  |  |
| Zuk et al, 2024^707^ | NR | Cohort study | 78 | / | / | / | / | AFSS | / | / |
|  |  |  | 58±9 years |  |  |  |  |  |  |  |
|  |  |  | 32.1% |  |  |  |  |  |  |  |
|  |  |  | NR |  |  |  |  |  |  |  |
|  |  |  | Paroxysmal (all participants) |  |  |  |  |  |  |  |
| Zylla et al, 2024^708^ | Germany | Cohort study | 102 | SF-36 | / | / | / | / | / | / |
|  |  |  | Median age reported: Heart failure with preserved ejection fraction group (n=20): 72 (IQR range 66-76.8) years & no heart failure with preserved ejection fraction group (n=22): 68.5 (IQR range 61-73.7) years |  |  |  |  |  |  |  |
|  |  |  | 41.2% |  |  |  |  |  |  |  |
|  |  |  | NR |  |  |  |  |  |  |  |
|  |  |  | Heart failure with preserved ejection fraction group: paroxysmal (75%) & persistent (25%)  No heart failure with preserved ejection fraction group: paroxysmal (77.3%) & persistent (22.7%) |  |  |  |  |  |  |  |
| Zylla et al, 2025^709^ | Germany | Cohort study | 2,769 | EQ-5D-5L | / | / | / | / | / | / |
|  |  |  | 72.5±10.9 years |  |  |  |  |  |  |  |
|  |  |  | 37.4% |  |  |  |  |  |  |  |
|  |  |  | NR |  |  |  |  |  |  |  |
|  |  |  | Paroxysmal (60.6%) & persistent or long-standing persistent (39.4%) |  |  |  |  |  |  |  |

*ADL = Katz index of independence in Activities of Daily Living****,*** *AF = Atrial Fibrillation,* *AF6 = Atrial Fibrillation six-item questionnaire****,*** *AFEQT = Atrial Fibrillation Effect on QualiTy-of-life, AFImpact = Atrial Fibrillation Impact****,*** *AFQLQ = Atrial fibrillation Quality of Life Questionnaire, AF-QoL = Atrial Fibrillation-Quality of Life, AFS/B = Atrial Fibrillation Symptom and Burden, AFSS = Atrial Fibrillation Symptom Severity, AFSymp = Atrial Fibrillation Symptom questionnaire, AQoL = Australian Quality of Life, ASI = Anxiety Sensitivity Index, ASTA = Arrhythmia‐Specific questionnaire in Tachycardia and Arrhythmia, AT = Atrial Tachycardia,* *BADL = Barthel Activities of Daily Living* ***,*** *BDI = Beck Depression Inventory, BDI-II = Beck Depression Inventory version two****,*** *BDI-SF = Beck Depression Inventory Short Form, CAQ = Cardiac Anxiety Questionnaire****,*** *CAST-QoL = Cardiac Arrhythmia Suppression Trial Quality of Life questionnaire****,*** *C-CAP = Cardiff Cardiac Ablation Patient-reported outcome measure, CES-D = Centre for Epidemiologic Studies Depression scale****,*** *CABG = Corpnary Artery Bypass Graft,* *Dartmouth COOP chart = Dartmouth Care Cooperative Information Project chart system****,*** *DASI = Duke Activity Status Index, DUKE = Duke Health Profile****,*** *EQ-5D = EuroQol five dimensions questionnaire****,*** *EQ-5D-3L = EuroQol five dimensions questionnaire (three level)****,*** *EQ-5D-5L = EuroQol five dimensions questionnaire (five level), EQ-VAS* ***=*** *EuroQol Visual Analogue Scale, GAD-7 = General Anxiety Disorder seven-item questionnaire, GDS = Geriatric Depression Scale****,*** *GPAQ = Global Physical Activity Questionnaire****,*** *HADS = Hospital Anxiety and Depression Scale****,*** *HAM-A = Hamilton Anxiety Scale****,*** *HAM-D = Hamilton Depression Scale, HRQoL = Health Related Quality of Life****,*** *HSQ = Health Status Questionnaire****,*** *IADL = Lawton Instrumental Activities of Daily Living****,*** *IIRS = Illness Intrusiveness Ratings Scale****,*** *IPAQ = International Physical Activity Questionnaire****,*** *IPAQ-SF = International Physical Activity Questionnaire-Short Form, IQR = Interquartile range, KPS = Karnofsky Performance Scale****,*** *LARQ = Leuven Arrhythmia Questionnaire****,*** *LTPA = Minnesota Leisure Time Physical Activity questionnaire, MAFSI = Mayo Atrial Fibrillation-Specific Symptom Inventory****,*** *MDI = Major Depression Inventory, MHIQ = McMaster Health index Questionnaire****,*** *MMSE = Mini-Mental State Examination****,*** *MoCA = Montreal Cognitive Assessment****,*** *MOCS-A= Measure of Current Status-Anxiety, MRI = Magnetic Resonance Imaging, NHP = Nottingham Health Profile, NR = data Not Reported****,*** *OARS = Older Americans’ Resources and Services Multidimensional Functional Assessment Questionnaire****,*** *PGWB = Psychological General Well-Being index, Phone-FITT = Physical Activity Frequency, Intensity, Time and Type questionnaire, PHQ-2 = Patient Health Questionnaire two item, PHQ-8 = Patient Health Questionnaire eight item****,*** *PHQ-9 = Patient Health Questionnaire nine item****,*** *POMS = Profile of Mood States questionnaire, PPAQ = Patient Perspective of Arrhythmia questionnaire, PROMIS= Patient-Reported Outcomes Measurement Information System****,*** *PROMIS-29 = Patient-Reported Outcomes Measurement Information System (29 items), PSS-10 = Perceived Stress Scale (10 items)****,*** *QDIS-MCC = Quality of Life Disease Impact Scale – Multiple Chronic Conditions****,*** *QLAF = Quality of Life in AF patients questionnaire, QLAFv2 = Quality of Life in AF patients questionnaire version two****,*** *Q-LES-Q = Quality of Life Enjoyment and Satisfaction Questionnaire****,*** *QLI = Quality of Life Index****,*** *QLI-CV = Quality of Life Index–Cardiac, 36 Item Health Survey, RCT = Randomised Control Trial, SAS = Zung Self-Rating Anxiety Scale****,*** *SCL = Symptom Checklist: Frequency and Severity Scale****,*** *SCL-90 = Symptom Checklist (90 items)****,*** *SCL-92 = Symptom Checklist (92 items), SDS = Zung Self-Rating Depression Scale****,*** *SF-12 = 12-item Short Form Survey****,*** *SF-12v2 = 12-item Short Form Survey version two, SF-36 = 36-item Short Form Survey****,*** *SF-36v2 = 36-item Short Form Survey version two****,*** *SF-6 = 6-item Short Form Survey****,*** *SF-8 = 8-item Short Form Survey****,*** *SGPALS = Saltin-Grimby Physical Activity Level Scale****,*** *SIP = Sickness Impact Profile****,*** *SPMSQ = Short Portable Mental Status Questionnaire****,*** *SPS = Stanford Presenteeism Scale****,*** *SRH = Self-Rated Health measure****,*** *STAI = Spielberger State-Trait Anxiety Inventory****,*** *T-MoCA = Telephone-based Montreal Cognitive Assessment****,*** *TICS-m = Telephone Interview for Cognitive Status-modified, UK = United Kingdom, USA = United States of America,* *VR-36 = Veterans RAND (Research and Development), WHO-5 = World Health Organisation- Five Well-Being Index****,*** *WHODAS 2.0 = World Health Organisation Disability Assessment Schedule version two, WHOQoL-BREF = World Health Organization Quality of Life-Brief Version questionnaire****,*** *WI = Whitely Index****,*** *WPAI = Work Productivity and Activity Impairment Instrument****,*** *YPAS = Yale Physical Activity Survey*

/ = Outcome not assessed using a validated patient-reported outcome measure that fits the inclusion criteria for this study

**References**

1. Abu HO, Saczynski JS, Mehawej J, Tisminetzky M, Kiefe CI, Goldberg RJ, et al. Clinically Meaningful Change in Quality of Life and Associated Factors Among Older Patients With Atrial Fibrillation. J Am Heart Assoc 2020;9:e016651.

2.	Abu HO [a], Saczynski JS, Ware J, Mehawej J, Paul T, Awad H, et al. Impact of comorbid conditions on disease-specific quality of life in older men and women with atrial fibrillation. Qual Life Res 2020;29:3285-96.

3.	Abed HS, Wittert GA, Leong DP, Shirazi MG, Bahrami B, Middeldorp ME, et al. Effect of weight reduction and cardiometabolic risk factor management on symptom burden and severity in patients with atrial fibrillation: a randomized clinical trial. JAMA 2013;310:2050-60.

4.	Accinni T, Maraone A, Bonucci A, D'Amato A, Lavalle C, Bersani FS, et al. Prevalence of demoralization and depressive symptoms in a sample of patients with supraventricular tachyarrhythmias: preliminary results. Front Psychiatry 2024;15:1355031.

5.	Ad N, Henry L, Friehling T, Wish M, Holmes SD. Minimally invasive stand-alone Cox-maze procedure for patients with nonparoxysmal atrial fibrillation. Ann Thorac Surg 2013;96:792-8.

6.	Ad N [a], Henry L, Holmes SD, Stone LE, Hunt S. The association between early atrial arrhythmia and long-term return to sinus rhythm for patients following the Cox maze procedure for atrial fibrillation. Eur J Cardiothorac Surg 2013;44:295-300.

7.	Ad N, Henry L, Hunt S. The concomitant cryosurgical Cox-Maze procedure using Argon based cryoprobes: 12 month results. J Cardiovasc Surg (Torino) 2011;52:593-9.

8.	Ad N [a], Henry L, Hunt S. The impact of surgical ablation in patients with low ejection fraction, heart failure, and atrial fibrillation. Eur J Cardiothorac Surg 2011;40:70-6.

9.	Ad N [b], Henry L, Hunt S, Holmes SD. The outcome of the Cox Maze procedure in patients with previous percutaneous catheter ablation to treat atrial fibrillation. Ann Thorac Surg 2011;91:1371-7.

10.	Ad N, Henry L, Hunt S, Holmes SD. Do we increase the operative risk by adding the Cox Maze III procedure to aortic valve replacement and coronary artery bypass surgery? J Thorac Cardiovasc Surg 2012;143:936-44.

11.	Ad N, Henry L, Hunt S, Holmes SD, Halpin L. Results of the Cox-Maze III/IV procedure in patients over 75 years old who present for cardiac surgery with a history of atrial fibrillation. J Cardiovasc Surg (Torino) 2013;54:281-8.

12.	Ahmed S, Ranchor AV, Crijns HJ, Van Veldhuisen DJ, Van Gelder IC. Effect of continuous versus episodic amiodarone treatment on quality of life in persistent atrial fibrillation. Europace 2010;12:785-91.

13.	Aguilera J, Hutt E, Kaur S, Saliba WI, Tarakji KG, Baranowski B, et al. Outcomes of atrial fibrillation ablation in patients with or without silent pulmonary veins from prior ablation procedure. J Cardiovasc Electrophysiol 2022;33:1994-2000.

14.	Ahn HJ, Lee SR, Choi EK, Han KD, Jung JH, Lim JH, et al. Association between exercise habits and stroke, heart failure, and mortality in Korean patients with incident atrial fibrillation: A nationwide population-based cohort study. PLoS Med 2021;18:e1003659.

15.	Ahn H, Lim HE, On YK, Selma JM, Kueffer FJ, van Bragt KA, et al. Long-term Outcome of Cryoballoon Ablation in Korean Patients With Atrial Fibrillation: Real-World Experience From the Cryo Global Registry. Korean Circ J 2024;54:619-33.

16.	Akintade BF, Chapa D, Friedmann E, Thomas SA. The Influence of Depression and Anxiety Symptoms on Health-Related Quality of Life in Patients With Atrial Fibrillation and Atrial Flutter. J Cardiovasc Nurs 2015;30:66-73.

17.	Al-Bashaireh AM, Alkouri O, Alharbi A, Khader Y, Hammoudeh A, Aljawarneh Y, et al. Factors Associated with Quality of Life among People with Atrial Fibrillation: Jordan Atrial Fibrillation Registry Study. Medicina (Kaunas) 2024;60:1262.

18.	Al-Jazairi MIH, Rienstra M, Klinkenberg TJ, Mariani MA, Van Gelder IC, Blaauw Y. Hybrid atrial fibrillation ablation in patients with persistent atrial fibrillation or failed catheter ablation. Neth Heart J 2019;27:142-51.

19.	Al-Kaisey AM, Parameswaran R, Bryant C, Anderson RD, Hawson J, Chieng D, et al. Atrial Fibrillation Catheter Ablation vs Medical Therapy and Psychological Distress: A Randomized Clinical Trial. JAMA 2023;330:925-33.

20.	Alli O, Doshi S, Kar S, Reddy V, Sievert H, Mullin C, et al. Quality of life assessment in the randomized PROTECT AF (Percutaneous Closure of the Left Atrial Appendage Versus Warfarin Therapy for Prevention of Stroke in Patients With Atrial Fibrillation) trial of patients at risk for stroke with nonvalvular atrial fibrillation. J Am Coll Cardiol 2013;61:1790-8.

21.	Almeida GdQ, Noblat LdA, Passos LCS, do Nascimento HF, Noblat LdACB. Quality of life analysis of patients in chronic use of oral anticoagulant: an observational study. Health Qual Life Outcomes 2011;9:91.

22.	Althouse AD, Abebe KZ, Paasche-Orlow MK, Lalama CM, Ferry D, Lancet M, et al. Design, rationale, and baseline characteristics of a randomized controlled trial evaluating a mobile relational agent to enhance atrial fibrillation self-care. Contemp Clinl Trials 2023;124:107015.

23.	Andrade JG, Champagne J, Deyell MW, Essebag V, Lauck S, Morillo C, et al. A randomized clinical trial of early invasive intervention for atrial fibrillation (EARLY-AF) - methods and rationale. Am Heart J 2018;206:94-104.

24.	Andrade JG, Deyell MW, Macle L, Steinberg JS, Glotzer TV, Hawkins NM, et al. Healthcare utilization and quality of life for atrial fibrillation burden: the CIRCA-DOSE study. Eur Heart J 2023;44:765-76.

25.	Andrade JG [a], Deyell MW, Macle L, Wells GA, Bennett M, Essebag V, et al. Progression of Atrial Fibrillation after Cryoablation or Drug Therapy. N Engl J Med 2023;388:105-16.

26.	Andrade JG, Wells GA, Deyell MW, Bennett M, Essebag V, Champagne J, et al. Cryoablation or Drug Therapy for Initial Treatment of Atrial Fibrillation. N Eng J Med 2021;384:305-15.

27.	Antoun I, Alkhayer A, Aljabal M, Alkhayer A, Simon P, Mahfoud Y, et al. The validity and reliability of the Arabic version of the EQ-5D in atrial fibrillation patients in a conflict country: a study from Syria. BMC Cardiovasc Disord 2024;24:541.

28.	Arbelo E, Aktaa S, Bollmann A, D'Avila A, Drossart I, Dwight J, et al. Quality indicators for the care and outcomes of adults with atrial fibrillation: Task Force for the development of quality indicators in atrial fibrillation of the European Heart Rhythm Association (EHRA) of the European Society of Cardiology (ESC): Developed in collaboration with the Heart Rhythm Society (HRS), the Asia Pacific Heart Rhythm Society (APHRS), and the Latin-American Heart Rhythm Society (LAHRS). Europace 2021;23:494-5.

29.	Ariansen I, Abdelnoor M, Tveit A, Gjesdal K. Guidelines' criteria for rate control in atrial fibrillation: are they useful? Scand Cardiovasc J 2010;44:132-8.

30.	Ariansen I, Dammen T, Abdelnoor M, Tveit A, Gjesdal K. Mental health and sleep in permanent atrial fibrillation patients from the general population. Clin Cardiol 2011;34:327-31.

31.	Armentaro G, D'Arrigo G, Pastori D, Crudo G, Daidone M, Soraci L, et al. Long-term cognitive function changes with non-vitamin K oral anticoagulants in older patients with atrial fibrillation. A multicenter cohort study. Eur J Clin Invest 2025;55:e14321.

32.	Arribas F, Ormaetxe JM, Peinado R, Perulero N, Ramirez P, Badia X. Validation of the AF-QoL, a disease-specific quality of life questionnaire for patients with atrial fibrillation. Europace 2010;12:364-70.

33.	Arvanitis P, Johansson AK, Frick M, Malmborg H, Gerovasileiou S, Larsson EM, et al. Serial Magnetic Resonance Imaging after Electrical Cardioversion of Recent Onset Atrial Fibrillation in Anticoagulant-Naïve Patients - A Prospective Study Exploring Clinically Silent Cerebral Lesions. J Atr Fibrillation 2020;13:2271.

34.	Athreya DS, Saczynski JS, Gurwitz JH, Monahan KM, Bamgbade BA, Paul TJ, et al. Cognitive impairment and treatment strategy for atrial fibrillation in older adults: The SAGE‐AF study. J Am Geriatr Soc 2024;72:2082-90.

35.	Badia X, Arribas F, Ormaetxe JM, Peinado R, de Los Terreros MS. Development of a questionnaire to measure health-related quality of life (HRQoL) in patients with atrial fibrillation (AF-QoL). Health Qual Life Outcomes 2007;5:37.

36.	Bagge L, Blomstrom P, Nilsson L, Einarsson GM, Jideus L, Blomstrom-Lundqvist C. Epicardial off-pump pulmonary vein isolation and vagal denervation improve long-term outcome and quality of life in patients with atrial fibrillation. J Thorac Cardiovasc Surg 2009;137:1265-71.

37.	Bagge L, Probst J, Jensen SM, Blomstrom P, Thelin S, Holmgren A, et al. Quality of life is not improved after mitral valve surgery combined with epicardial left atrial cryoablation as compared with mitral valve surgery alone: a substudy of the double blind randomized SWEDish Multicentre Atrial Fibrillation study (SWEDMAF). Europace 2018;20(FI_3):f343-f50.

38.	Bai Y, Bai R, Wu JH, Zhang T, Liu N, Shi XB, et al. Differences in Quality of Life Between Atrial Fibrillation Patients with Low Stroke Risk Treated With and Without Catheter Ablation. J Am Heart Assoc 2015;4:e002130.

39.	Bakker RC, Akin S, Rizopoulos D, Kik C, Takkenberg JJ, Bogers AJ. Results of clinical application of the modified maze procedure as concomitant surgery. Interact Cardiovasc Thorac Surg 2013;16:151-6.

40.	Baldus S, Beyer-Westendorf J, Möllmann H, Rottbauer W, Beyerlein E, Goette A. Edoxaban in patients with non-valvular atrial fibrillation after percutaneous coronary intervention: ENCOURAGE-AF design. Sci Rep 2023;13:18215.

41.	Ball J, Carrington MJ, Stewart S. Mild cognitive impairment in high-risk patients with chronic atrial fibrillation: a forgotten component of clinical management? Heart 2013;99:542-7.

42.	Ballard DW, Reed ME, Singh N, Rauchwerger AS, Hamity CA, Warton EM, et al. Emergency Department Management of Atrial Fibrillation and Flutter and Patient Quality of Life at One Month Postvisit. Ann Emerg Med 2015;66:646-54.

43.	Bang C, Park S. Symptom Clusters, Psychological Distress, and Quality of Life in Patients with Atrial Fibrillation. Healthcare 2023;11:08.

44.	Bamgbade BA, Sanghai SR, McManus DD, Lessard D, Waring ME, Forrester S, et al. Psychosocial and cognitive multimorbidity and health-related quality of life and symptom burden in older adults with atrial fibrillation: The systematic assessment of geriatric elements in atrial fibrillation (SAGE-AF) cohort study. Arch Gerontol Geriatr 2020;90:104117.

45.	Barber M, Tait RC, Scott J, Rumley A, Lowe GD, Stott DJ. Dementia in subjects with atrial fibrillation: hemostatic function and the role of anticoagulation. J Thromb Haemost 2004;2:1873-8.

46.	Barbagallo M, Springer A, Vanetta C, Allemann M, Lee P, Saeedi S, et al. Coffee Consumption Correlates With Better Cognitive Performance in Patients With a High Incidence for Stroke. J Am Heart Assoc 2025;14:e034365.

47.	Barmano N, Charitakis E, Karlsson JE, Nystrom FH, Walfridsson H, Walfridsson U. Predictors of improvement in arrhythmia-specific symptoms and health-related quality of life after catheter ablation of atrial fibrillation. Clin Cardiol 2019;42:247-55.

48.	Barmano N, Walfridsson U, Walfridsson H, Karlsson JE. Structured care of patients with atrial fibrillation improves guideline adherence. J Atrial Fibrillation 2016;9:1498.

49.	Barrios V, Escobar C, Baron Esquivias G, Gomez Doblas JJ, Recalde Del Vigo E, Segura Martinez L, et al. Quality of life, adherence and satisfaction of patients with auricular fibrillation treated with dabigatran or vitamin K antagonists. Rev Clin Esp 2019;219:285-92.

50.	Bazemore TC, Bolger LE, Sears SF, Sadaf MI, Gehi AK. Gender differences in symptoms and functional status in patients with atrial fibrillation undergoing catheter ablation. Pacing Clin Electrophysiol 2019;42:224-9.

51.	Beaver TM, Hedna VS, Khanna AY, Miles WM, Price CC, Schmalfuss IM, et al. Thoracoscopic Ablation With Appendage Ligation Versus Medical Therapy for Stroke Prevention: A Proof-of-Concept Randomized Trial. Innovations (Phila) 2016;11:99-105.

52.	Benzimra M, Bonnamour B, Duracinsky M, Lalanne C, Aubert JP, Chassany O, et al. Real-life experience of quality of life, treatment satisfaction, and adherence in patients receiving oral anticoagulants for atrial fibrillation. Patient Prefer Adherence 2018;12:79-87.

53.	Berg J, Lindgren P, Nieuwlaat R, Bouin O, Crijns H. Factors determining utility measured with the EQ-5D in patients with atrial fibrillation. Qual Life Res 2010;19:381-90.

54.	Berger WR, Krul SP, van der Pol JA, van Dessel PF, Conrath CE, Wilde AA, et al. Documented atrial fibrillation recurrences after pulmonary vein isolation are associated with diminished quality of life. J Cardiovasc Med 2016;17:201-8.

55.	Berkowitsch A, Neumann T, Kurzidim K, Reiner C, Kuniss M, Siemon G, et al. Comparison of generic health survey SF-36 and arrhythmia related symptom severity check list in relation to post-therapy AF recurrence. Europace 2003;5:351-5.

56.	Berry C, Stewart S, Payne EM, McArthur JD, McMurray JJ. Electrical cardioversion for atrial fibrillation: outcomes in "real-life" clinical practice. Int J Cardiol 2001;81:29-35.

57.	Bittman J, Thomson CJ, Lyall LA, Alexis SL, Lyall ET, Cannatella SL, et al. Effect of an Exercise and Nutrition Program on Quality of Life in Patients With Atrial Fibrillation: The Atrial Fibrillation Lifestyle Project (ALP). CJC Open 2022;4:685-94.

58.	Biviano AB, Hunter TD, Dandamudi G, Fishel RS, Gidney B, Herweg B, et al. Healthcare Utilization and Quality of Life Improvement after Ablation for Paroxysmal AF in Younger and Older Patients. Pacing Clin Electrophysiol 2017;40:391-400.

59.	Björkenheim A, Brandes A, Magnuson A, Chemnitz A, Edvardsson N, Poçi D. Patient-Reported Outcomes in Relation to Continuously Monitored Rhythm Before and During 2 Years After Atrial Fibrillation Ablation Using a Disease-Specific and a Generic Instrument. J Am Heart Assoc 2018;7:e008362.

60.	Björkenheim A, Brandes A, Magnuson A, Chemnitz A, Svedberg L, Edvardsson N, et al. Assessment of Atrial Fibrillation-Specific Symptoms Before and 2 Years After Atrial Fibrillation Ablation: Do Patients and Physicians Differ in Their Perception of Symptom Relief? JACC Clin Electrophysiol 2017;3:1168-76.

61.	Blandino A, Toso E, Scaglione M, Anselmino M, Ferraris F, Sardi D, et al. Long-term efficacy and safety of two different rhythm control strategies in elderly patients with symptomatic persistent atrial fibrillation. J Cardiovasc Electrophysiol 2013;24:731-8.

62.	Blomström-Lundqvist C, Gizurarson S, Schwieler J, Jensen SM, Bergfeldt L, Kennebäck G, et al. Effect of Catheter Ablation vs Antiarrhythmic Medication on Quality of Life in Patients With Atrial Fibrillation: The CAPTAF Randomized Clinical Trial. JAMA 2019;321:1059-68.

63.	Bo M, Sciarrillo I, Li Puma F, Badinella Martini M, Falcone Y, Iacovino M, et al. Effects of Oral Anticoagulant Therapy in Medical Inpatients ≥65 Years With Atrial Fibrillation. Am J Cardiol 2016;117:590-5.

64.	Boehmer AA, Kaess BM, Ruckes C, Meyer C, Metzner A, Rillig A, et al. Pulmonary Vein Isolation or Pace and Ablate in Elderly Patients With Persistent Atrial Fibrillation (ABLATE Versus PACE)-Rationale, Methods, and Design. Can J Cardiol 2024;40:2429-40.

65.	Boersma L, Koźluk E, Maglia G, de Sousa J, Grebe O, Eckardt L, et al. Paroxysmal and persistent atrial fibrillation ablation outcomes with the pulmonary vein ablation catheter GOLD duty-cycled phased radiofrequency ablation catheter: quality of life and 12-month efficacy results from the GOLD Atrial Fibrillation Registry. Europace 2020;22:888-96.

66.	Borland M, Bergfeldt L, Cider A, Rosenkvist A, Jakobsson M, Olsson K, et al. Effects of 3 months of detraining following cardiac rehabilitation in patients with atrial fibrillation. Eur Rev Aging Phy 2022;19:14.

67.	Bostrom JA, Saczynski JS, Hajduk A, Donahue K, Rosenthal LS, Browning C, et al. Burden of Psychosocial and Cognitive Impairment in Patients With Atrial Fibrillation. Crit Pathw Cardiol 2017;16:71-5.

68.	Boveda S, Metzner A, Nguyen DQ, Chun KRJ, Goehl K, Noelker G, et al. Single-Procedure Outcomes and Quality-of-Life Improvement 12 Months Post-Cryoballoon Ablation in Persistent Atrial Fibrillation: Results From the Multicenter CRYO4PERSISTENT AF Trial. JACC Clin Electrophysiol 2018;4:1440-7.

69.	Bowyer JL, Tully PJ, Ganesan AN, Chahadi FK, Singleton CB, McGavigan AD. A Randomised Controlled Trial on the Effect of Nurse-Led Educational Intervention at the Time of Catheter Ablation for Atrial Fibrillation on Quality of Life, Symptom Severity and Rehospitalisation. Heart Lung Circ 2017;26:73-81.

70.	Boyalla V, Haldar S, Khan H, Kralj-Hans I, Banya W, Lord J, et al. Long-term clinical outcomes and cost-effectiveness of catheter vs thoracoscopic surgical ablation in long-standing persistent atrial fibrillation using continuous cardiac monitoring: CASA-AF randomized controlled trial. Heart Rhythm 2024;21:1562‐9.

71.	Braganca EO, Filho BL, Maria VH, Levy D, de Paola AA. Validating a new quality of life questionnaire for atrial fibrillation patients. Int J Cardiol 2010;143:391-8.

72.	Bubien RS, Knotts-Dolson SM, Plumb VJ, Kay GN. Effect of radiofrequency catheter ablation on health-related quality of life and activities of daily living in patients with recurrent arrhythmias. Circulation 1996;94:1585-91.

73.	Buist TJ, Adiyaman A, Beukema RJ, Smit JJJ, Delnoy P, Hemels MEW, et al. Quality of life after catheter and minimally invasive surgical ablation of paroxysmal and early persistent atrial fibrillation: results from the SCALAF trial. Clin Res Cardiol 2020;109:215-24.

74.	Bulková V, Fiala M, Havránek S, Simek J, Skňouřil L, Januška J, et al. Improvement in quality of life after catheter ablation for paroxysmal versus long-standing persistent atrial fibrillation: a prospective study with 3-year follow-up. J Am Heart Assoc 2014;3:e000881.

75.	Bunch TJ, May H, Cutler M, Woller SC, Jacobs V, Stevens SM, et al. Impact of anticoagulation therapy on the cognitive decline and dementia in patients with non-valvular atrial fibrillation (cognitive decline and dementia in patients with non-valvular atrial fibrillation [CAF] trial). J Arrhythm 2022;38:997-1008.

76.	Burns JL, Sears SF, Sotile R, Schwartzman DS, Hoyt RH, Alvarez LG, et al. Do patients accept implantable atrial defibrillation therapy? Results from the Patient Atrial Shock Survey of Acceptance and Tolerance (PASSAT) Study. J Cardiovasc Electrophysiol 2004;15:286-91.

77.	Caramelli B, Yu PC, Cardozo FAM, Magalhaes IR, Spera RR, Amado DK, et al. Effects of dabigatran versus warfarin on 2-year cognitive outcomes in old patients with atrial fibrillation: results from the GIRAF randomized clinical trial. BMC Med 2022;20:374.

78.	Carnlöf C, Malinowsky C, Insulander P, Gadler F, Jensen-Urstad M, M I. The Symptom Checklist: Frequency and Severity Scale – translation and psychometric properties of the Swedish version. Nord J Nurs Res 2020;40:97-104.

79.	Caceres BA, Hickey KT, Bakken SB, Biviano AB, Garan H, Goldenthal IL, et al. Mobile Electrocardiogram Monitoring and Health-Related Quality of Life in Patients With Atrial Fibrillation: Findings From the iPhone Helping Evaluate Atrial Fibrillation Rhythm Through Technology (iHEART) Study. J Cardiovasc Nurs 2020;35:327-36.

80.	Calò L, Lamberti F, Loricchio ML, Castro A, Shpun S, Boggi A, et al. Long-term follow-up of right atrial ablation in patients with atrial fibrillation. J Cardiovasc Electrophysiol 2004;15:37-43.

81.	Cannavan PMS, Cannavan FPS, Walfridsson U, Lopes M. Translation and Validation of the Arrhythmia-Specific Questionnaire in Tachycardia and Arrhythmia (ASTA) to the Brazilian Context: An Instrument Focusing on Arrhythmia Symptoms. Cardiol Res Pract 2020;2020:1402916.

82.	Carlsson J, Miketic S, Windeler J, Cuneo A, Haun S, Micus S, et al. Randomized trial of rate-control versus rhythm-control in persistent atrial fibrillation: the Strategies of Treatment of Atrial Fibrillation (STAF) study. J Am Coll Cardiol 2003;41:1690-6.

83.	Carnlöf C, Insulander P, Pettersson PH, Jensen-Urstad M, Fossum B. Health-related quality of life in patients with atrial fibrillation undergoing pulmonary vein isolation, before and after treatment. Eur J Cardiovasc Nurs 2010;9:45-9.

84.	Carnlöf C, Iwarzon M, Jensen-Urstad M, Gadler F, Insulander P. Women with atrial fibrillation undergoing pulmonary vein isolation are more symptomatic but improve more in health-related quality of life and symptom burden than men. Scand Cardiovasc J 2022;56:316-24.

85.	Castro-Urda V, Segura-Dominguez M, Jiménez-Sánchez D, Aguilera-Agudo C, García-Izquierdo E, De la Rosa Rojas Y, et al. Efficacy and safety of Proglide use and early discharge after atrial fibrillation ablation compared to standard approach. PROFA trial. Pacing Clin Electrophysiol 2023;46:598-606.

86.	Cha YM, Friedman PA, Asirvatham SJ, Shen WK, Munger TM, Rea RF, et al. Catheter ablation for atrial fibrillation in patients with obesity. Circulation 2008;117:2583-90.

87.	Chandan N, Matthews V, He H, Lachlan T, Lim VG, Joshi S, et al. Cardiac prehabilitation, rehabilitation and education in first-time atrial fibrillation (AF) ablation (CREED AF): study protocol for a randomised controlled trial. PLoS One 2024;19:e0310951.

88.	Chapa DW, Akintade B, Schron E, Friedmann E, Thomas SA. Is Health-Related Quality of Life a Predictor of Hospitalization or Mortality Among Women or Men With Atrial Fibrillation? J Cardiovasc Nurs 2014;29:555-64.

89.	Charitakis E, Walfridsson U, Nyström F, Nylander E, Strömberg A, Alehagen U, et al. Symptom burden, Metabolic profile, Ultrasound findings, Rhythm, neurohormonal activation, haemodynamics and health-related quality of life in patients with atrial Fibrillation (SMURF): a protocol for an observational study with a randomised interventional component. BMJ Open 2015;5:e008723.

90.	Chen WJ, Gan CX, Cai YW, Liu YY, Xiao PL, Zou LL, et al. Impact of high-power short-duration atrial fibrillation ablation technique on the incidence of silent cerebral embolism: a prospective randomized controlled study. BMC Med 2023;21:461.

91.	Chen Y, Tertulien T, Althouse AD, Johnson A, Gisi B, Magnani JW. Association of patient-reported outcomes with hospitalization risk in atrial fibrillation. Am Heart J Plus 2021;2:100007.

92.	Cherian TS, Shrader P, Fonarow GC, Allen LA, Piccini JP, Peterson ED, et al. Effect of Atrial Fibrillation on Mortality, Stroke Risk, and Quality-of-Life Scores in Patients With Heart Failure (from the Outcomes Registry for Better Informed Treatment of Atrial Fibrillation [ORBIT-AF]). Am J Cardiol 2017;119:1763-9.

93.	Chernyavskiy A, Kareva Y, Pak I, Rakhmonov S, Pokushalov E, Romanov A. Quality of Life after Surgical Ablation of Persistent Atrial Fibrillation: A Prospective Evaluation. Heart Lung Circ 2016;25:378-83.

94.	Chieng D, Sugumar H, Segan L, Tan C, Vizi D, Nanayakkara S, et al. Atrial Fibrillation Ablation for Heart Failure With Preserved Ejection Fraction: A Randomized Controlled Trial. JACC Heart Fail 2023;11:646-58.

95.	Chung MK, Shemanski L, Sherman DG, Greene HL, Hogan DB, Kellen JC, et al. Functional status in rate- versus rhythm-control strategies for atrial fibrillation: results of the Atrial Fibrillation Follow-Up Investigation of Rhythm Management (AFFIRM) Functional Status Substudy. J Am Coll Cardiol 2005;46:1891-9.

96.	Clarkesmith DE, Pattison HM, Lip GY, Lane DA. Educational intervention improves anticoagulation control in atrial fibrillation patients: the TREAT randomised trial. PLoS One 2013;8:e74037.

97.	Conde D, Costabel JP, Aragon M, Lambardi F, Trivi M. Vernakalant: Perception of state of health in patients with a recent-onset atrial fibrillation. Cardiol J 2014;21:304-8.

98.	Conen D, Rodondi N, Mueller A, Beer J, Auricchio A, Ammann P, et al. Design of the Swiss Atrial Fibrillation Cohort Study (Swiss-AF): structural brain damage and cognitive decline among patients with atrial fibrillation. Swiss Med Wkly 2017;147:w14467.

99.	Conen D, Rodondi N, Müller A, Beer JH, Ammann P, Moschovitis G, et al. Relationships of Overt and Silent Brain Lesions With Cognitive Function in Patients With Atrial Fibrillation. J Am Coll Cardiol 2019;73:989-99.

100.	Contreras Muruaga MDM, Vivancos J, Reig G, Gonzalez A, Cardona P, Ramirez-Moreno JM, et al. Satisfaction, quality of life and perception of patients regarding burdens and benefits of vitamin K antagonists compared with direct oral anticoagulants in patients with nonvalvular atrial fibrillation. J Comp Eff Res 2017;6:303-12.

101.	Cooper HA, Bloomfield DA, Bush DE, Katcher MS, Rawlins M, Sacco JD, et al. Relation between achieved heart rate and outcomes in patients with atrial fibrillation (from the Atrial Fibrillation Follow-up Investigation of Rhythm Management [AFFIRM] Study). Am J Cardiol 2004;93:1247-53.

102.	Corino VD, Platonov PG, Enger S, Tveit A, Ulimoen SR. Circadian variation of variability and irregularity of heart rate in patients with permanent atrial fibrillation: relation to symptoms and rate control drugs. Am J Physiol Heart Circ Physiol 2015;309:H2152-7.

103.	Cosansu K, Ureyen CM, Yılmaz S. Effect of novel oral anticoagulants on Hospital Anxiety and Depression Scale scores. Herz 2019;44:743-9.

104.	Coyne KS, Edvardsson N, Ryden A. Development and Validation of the AFImpact: An Atrial Fibrillation-Specific Measure of Patient-Reported Health-Related Quality of Life. Value Health 2017;20:1355-61.

105.	Crowley R, Chieng D, Sugumar H, Ling LH, Segan L, William J, et al. Catheter ablation for persistent atrial fibrillation: patterns of recurrence and impact on quality of life and health care utilization. Eur Heart J 2024;45:2604‐16.

106.	Dąbrowski R, Smolis-Bąk E, Kowalik I, Kazimierska B, Wójcicka M, Szwed H. Quality of life and depression in patients with different patterns of atrial fibrillation. Kardiol Pol 2010;68:1133-9.

107.	Dagres N, Nieuwlaat R, Vardas PE, Andresen D, Lévy S, Cobbe S, et al. Gender-related differences in presentation, treatment, and outcome of patients with atrial fibrillation in Europe: a report from the Euro Heart Survey on Atrial Fibrillation. J Am Coll Cardiol 2007;49:572-7.

108.	Das M, Wynn GJ, Saeed Y, Gomes S, Morgan M, Ronayne C, et al. Pulmonary Vein Re-Isolation as a Routine Strategy Regardless of Symptoms: The PRESSURE Randomized Controlled Trial. JACC Clin Electrophysiol 2017;3:602-11.

109.	De Caterina R, Brüggenjürgen B, Darius H, Köhler S, Lucerna M, Pecen L, et al. Quality of life and patient satisfaction in patients with atrial fibrillation on stable vitamin K antagonist treatment or switched to a non-vitamin K antagonist oral anticoagulant during a 1-year follow-up: A PREFER in AF Registry substudy. Arch Cardiovasc Dis 2018;111:74-84.

110.	De Potter T, Van Herendael H, Balasubramaniam R, Wright M, Agarwal SC, Sanders P, et al. Safety and long-term effectiveness of paroxysmal atrial fibrillation ablation with a contact force-sensing catheter: real-world experience from a prospective, multicentre observational cohort registry. Europace 2018;20(FI_3):f410-f8.

111.	De With RR, Rienstra M, Smit MD, Weijs B, Zwartkruis VW, Hobbelt AH, et al. Targeted therapy of underlying conditions improves quality of life in patients with persistent atrial fibrillation: results of the RACE 3 study. Europace 2019;21:563-71.

112.	Deftereos S, Giannopoulos G, Efremidis M, Kossyvakis C, Katsivas A, Panagopoulou V, et al. Colchicine for prevention of atrial fibrillation recurrence after pulmonary vein isolation: mid-term efficacy and effect on quality of life. Heart Rhythm 2014;11:620-8.

113.	Delesie M, Knaepen L, Dendale P, Vijgen J, Ector J, Verbeeck J, et al. Effect of targeted education for atrial fibrillation patients: design of the EduCare-AF Study. Eur J Clin Invest 2021;51:e13442.

114.	Demolder A, O'Neill L, El Haddad M, Scherr D, Vijgen J, Wolf M, et al. No Effect of Continued Antiarrhythmic Drug Treatment on Top of Optimized Pulmonary Vein Isolation in Patients With Persistent Atrial Fibrillation: Results From the POWDER-AF2 Trial. Circ Arrhythm Electrophysiol 2023;16:e012043.

115.	Desteghe L, Engelhard L, Vijgen J, Koopman P, Dilling-Boer D, Schurmans J, et al. Effect of reinforced, targeted in-person education using the Jessa Atrial fibrillation Knowledge Questionnaire in patients with atrial fibrillation: A randomized controlled trial. Eur J Cardiovasc Nurs 2019;18:194-203.

116.	Dhruva SS, Ross JS, Akar JG, Caldwell B, Childers K, Chow W, et al. Aggregating multiple real-world data sources using a patient-centered health-data-sharing platform. NPJ Digit Med 2020;3:60.

117.	Dhruva SS, Shah ND, Vemulapalli S, Deshmukh A, Beatty AL, Gamble GM, et al. Heart Watch Study: protocol for a pragmatic randomised controlled trial. BMJ Open 2021;11:e054550.

118.	Dierkes S, Hennersdorf MG, Vester EG, Perings C. [Long-term follow-up of right atrial multilinear high-frequency ablation in the treatment of recurrent paroxysmal atrial fibrillation]. Dtsch Med Wochenschr 2003;128:130-4.

119.	Ding M, Fratiglioni L, Johnell K, Santoni G, Fastbom J, Ljungman P, et al. Atrial fibrillation, antithrombotic treatment, and cognitive aging: A population-based study. Neurology 2018;91:e1732-40.

120.	Dohi T, Nakatani D, Inoue K, Hikoso S, Oka T, Hayashi K, et al. Effect of Extensive Ablation on Recurrence in Patients with Persistent Atrial Fibrillation Treated with Pulmonary Vein Isolation (EARNEST-PVI) trial: Design and rationale. J Cardiol 2019;74:164-8.

121.	Dorian P, Burk C, Mullin CM, Bubien R, Godejohn D, Reynolds MR, et al. Interpreting changes in quality of life in atrial fibrillation: how much change is meaningful? Am Heart J 2013;166:381-7.e8.

122.	Dorian P, Guerra PG, Kerr CR, O'Donnell SS, Crystal E, Gillis AM, et al. Validation of a new simple scale to measure symptoms in atrial fibrillation: the Canadian Cardiovascular Society Severity in Atrial Fibrillation scale. Circ Arrhythm Electrophysiol 2009;2:218-24.

123.	Dorian P, Jung W, Newman D, Paquette M, Wood K, Ayers GM, et al. The impairment of health-related quality of life in patients with intermittent atrial fibrillation: implications for the assessment of investigational therapy. J Am Coll Cardiol 2000;36:1303-9.

124.	Dorian P, Paquette M, Newman D, Green M, Connolly SJ, Talajic M, et al. Quality of life improves with treatment in the Canadian Trial of Atrial Fibrillation. Am Heart J 2002;143:984-90.

125.	Doshi RN, Daoud EG, Fellows C, Turk K, Duran A, Hamdan MH, et al. Left ventricular-based cardiac stimulation post AV nodal ablation evaluation (the PAVE study). J Cardiovasc Electrophysiol 2005;16:1160-5.

126.	Dossett ML, Needles EW, Donahue Z, Gadenne G, Macklin EA, Ruskin JN, et al. A SMART approach to reducing paroxysmal atrial fibrillation symptoms: Results from a pilot randomized controlled trial. Heart Rhythm O2 2021;2:326-32.

127.	Doucet J, Gréboval-Furstenfeld E, Tavildari A, M'Bello L, Delaunay O, Pesqué T, et al. Which parameters differ in very old patients with chronic atrial fibrillation treated by anticoagulant or aspirin? Antithrombotic treatment of atrial fibrillation in the elderly. Fundam Clin Pharmacol 2008;22:569-74.

128.	Driessen AHG, Berger WR, Bierhuizen MFA, Piersma FR, van den Berg NWE, Neefs J, et al. Quality of life improves after thoracoscopic surgical ablation of advanced atrial fibrillation: Results of the Atrial Fibrillation Ablation and Autonomic Modulation via Thoracoscopic Surgery (AFACT) study. J Thorac Cardiovasc Surg 2018;155:972-80.

129.	Du M, Cheng T, Ye Y, Wei Y. Prevalence and Risk Factors of Postprocedure Depression in Patients with Atrial Fibrillation after Radiofrequency Ablation. Cardiol Res Pract 2023:4635336.

130.	Du X, Guo L, He X, Jia Y, Wu J, Long D, et al. A comparison of the real world effectiveness of catheter ablation and drug therapy in atrial fibrillation patients in a Chinese setting. BMC Cardiovasc Disord 2017;17:204.

131.	Duan W, Baojun R. Application Effect of Multi-Dimension Nursing Combined with GRACE Scoring System in Patients with Atrial Fibrillation after Green Precision Catheter Radiofrequency Ablation. Heart Surg Forum 2023;26(5):e600-8.

132.	Dublin S, Anderson ML, Haneuse SJ, Heckbert SR, Crane PK, Breitner JC, et al. Atrial fibrillation and risk of dementia: a prospective cohort study. J Am Geriatr Soc 2011;59:1369-75.

133.	Dudink E, Essers B, Holvoet W, Weijs B, Luermans J, Ramanna H, et al. Acute cardioversion vs a wait-and-see approach for recent-onset symptomatic atrial fibrillation in the emergency department: Rationale and design of the randomized ACWAS trial. Am Heart J 2017;183:49-53.

134.	Duff HJ, Raj SR, Exner DV, Sheldon RS, Roach D, Mitchell LB, et al. Randomized controlled trial of fixed rate versus rate responsive pacing after radiofrequency atrioventricular junction ablation: quality of life, ventricular refractoriness, and paced QT dispersion. J Cardiovasc Electrophysiol 2003;14:1163-70.

135.	Dulai R, Furniss SS, Sulke N, Freemantle N, Lambiase PD, Farwell D, et al. A randomized sham-controlled study of pulmonary vein isolation in symptomatic atrial fibrillation (The SHAM-PVI study): Study design and rationale. Clin Cardiol 2023;46:973-80.

136.	Dulai R, Sulke N, Freemantle N, Lambiase PD, Farwell D, Srinivasan NT, et al. Pulmonary Vein Isolation vs Sham Intervention in Symptomatic Atrial Fibrillation: The SHAM-PVI Randomized Clinical Trial. JAMA 2024;332:1165-73.

137.	Duytschaever M, De Pooter J, Demolder A, El Haddad M, Phlips T, Strisciuglio T, et al. Long-term impact of catheter ablation on arrhythmia burden in low-risk patients with paroxysmal atrial fibrillation: The CLOSE to CURE study. Heart Rhythm 2020;17:535-43.

138.	Duytschaever M, Demolder A, Phlips T, Sarkozy A, El Haddad M, Taghji P, et al. PulmOnary vein isolation With vs. without continued antiarrhythmic Drug trEatment in subjects with Recurrent Atrial Fibrillation (POWDER AF): results from a multicentre randomized trial. Eur Heart J 2018;39:1429-37.

139.	Echouffo-Tcheugui JB, Shrader P, Thomas L, Gersh BJ, Kowey PR, Mahaffey KW, et al. Care Patterns and Outcomes in Atrial Fibrillation Patients With and Without Diabetes: ORBIT-AF Registry. J Am Coll Cardiol 2017;70:1325-35.

140.	Edwards A, Elwyn G, Hood K, Atwell C, Robling M, Houston H, et al. Patient-based outcome results from a cluster randomized trial of shared decision making skill development and use of risk communication aids in general practice. Fam Pract 2004;21:347-54.

141.	Efremidis M, Letsas KP, Lioni L, Giannopoulos G, Korantzopoulos P, Vlachos K, et al. Association of quality of life, anxiety, and depression with left atrial ablation outcomes. Pacing Clin Electrophysiol 2014;37:703-11.

142.	Elliott AD, Verdicchio CV, Gallagher C, Linz D, Mahajan R, Mishima R, et al. Factors Contributing to Exercise Intolerance in Patients With Atrial Fibrillation. Heart Lung Circ 2021;30:947-54.

143.	Elliott AD, Verdicchio CV, Mahajan R, Middeldorp ME, Gallagher C, Mishima RS, et al. An Exercise and Physical Activity Program in Patients With Atrial Fibrillation: The ACTIVE-AF Randomized Controlled Trial. JACC Clin Electrophysiol 2023;9:455-65.

144.	Elliott DJ, Zhao L, Jasper SE, Lieber EA, Klein AL, Weintraub WS. Health status outcomes after cardioversion for atrial fibrillation: results from the Assessment of Cardioversion Using Transesophageal Echocardiography (ACUTE) II Trial. Am Heart J 2008;156:374.e1-6.

145.	Erdogan A, Carlsson J, Neumann T, Berkowitsch A, Neuzner J, Hamm CW, et al. Quality-of-life in patients with paroxysmal atrial fibrillation after catheter ablation: results of long-term follow-up. Pacing Clin Electrophysiol 2003;26:678-84.

146.	Essebag V, Azizi Z, Alipour P, Khaykin Y, Leong-Sit P, Sarrazin JF, et al. Relationship between quality of life and burden of recurrent atrial fibrillation following ablation: CAPCOST multicentre cohort study. Europace 2020;22:1017-25.

147.	Evans JM, Withers KL, Lencioni M, Carolan-Rees G, Wood KA, Patrick H, et al. Quality of life benefits from arrhythmia ablation: A longitudinal study using the C-CAP questionnaire and EQ5D. Pacing Clin Electrophysiol 2019;42:705-11.

148.	Farina E, Magni E, Ambrosini F, Manfredini R, Binda A, Sina C, et al. Neuropsychological deficits in asymptomatic atrial fibrillation. Acta Neurol Scand 1997;96:310-6.

149.	Fareau S, Baumstarck K, Farcet A, Molines C, Auquier P, Retornaz F. [Quality of life of elderly people on oral anticoagulant for atrial fibrillation: VKA versus direct oral anticoagulants]. Geriatr Psychol Neuropsychiatr Vieil 2015;13:45-54.

150.	Farwati M, Amin M, Saliba WI, Nakagawa H, Tarakji KG, Diab M, et al. Impact of redo ablation for atrial fibrillation on patient-reported outcomes and quality of life. J Cardiovasc Electrophysiol 2023;34:54-61.

151.	Farwati M, Wazni OM, Tarakji KG, Diab M, Scandinaro A, Amin M, et al. Super and Nonresponders to Catheter Ablation for Atrial Fibrillation: A Quality-of-Life Assessment Using Patient Reported Outcomes. Circ Arrhythm Electrophysiol 2021;14:e009938.

152.	Feinberg JB, Olsen MH, Brandes A, Raymond L, Nielsen WB, Nielsen EE, et al. Lenient rate control versus strict rate control for atrial fibrillation: a protocol for the Danish Atrial Fibrillation (DanAF) randomised clinical trial. BMJ Open 2021;11:e044744.

153.	Fengsrud E, Wickbom A, Almroth H, Englund A, Ahlsson A. Total endoscopic ablation of patients with long-standing persistent atrial fibrillation: a randomized controlled study. Interact Cardiovasc Thorac Surg 2016;23:292-8.

154.	Ferguson C, Allida S, William S, Downie A, Wilson T, John R, et al. INFORM-AF II-Phase 1 pilot randomised controlled trial evaluating the effect of an atrial fibrillation digital education programme: study protocol. J Clin Nurs 2024;33:4138‐47.

155.	Ferreira M, Cunha PS, Felix AC, Fonseca H, Oliveira M, Laranjo S, et al. AFTER-CA: Autonomic Function Transformation and Evaluation Following Catheter Ablation in Atrial Fibrillation. J Clin Med 2024;13:5796.

156.	Feyz L, Theuns DA, Bhagwandien R, Strachinaru M, Kardys I, Van Mieghem NM, et al. Atrial fibrillation reduction by renal sympathetic denervation: 12 months' results of the AFFORD study. Clin Res Cardiol 2019;108:634-42.

157.	Fiala M, Bulkova V, Sknouril L, Nevralova R, Toman O, Januska J, et al. Functional improvement after successful catheter ablation for long-standing persistent atrial fibrillation. Europace 2017;19:1781-9.

158.	Fiala M, Wichterle D, Bulková V, Sknouril L, Nevralová R, Toman O, et al. A prospective evaluation of haemodynamics, functional status, and quality of life after radiofrequency catheter ablation of long-standing persistent atrial fibrillation. Europace 2014;16:15-25.

159.	Fichtner S, Deisenhofer I, Kindsmuller S, Dzijan-Horn M, Tzeis S, Reents T, et al. Prospective assessment of short- and long-term quality of life after ablation for atrial fibrillation. J Cardiovasc Electrophysiol 2012;23:121-7.

160.	Flaker GC, Pogue J, Yusuf S, Pfeffer MA, Goldhaber SZ, Granger CB, et al. Cognitive function and anticoagulation control in patients with atrial fibrillation. Circ Cardiovasc Qual Outcomes 2010;3:277-83.

161.	Fleischmann KE, Orav EJ, Lamas GA, Mangione CM, Schron EB, Lee KL, et al. Atrial fibrillation and quality of life after pacemaker implantation for sick sinus syndrome: data from the Mode Selection Trial (MOST). Am Heart J 2009;158:78-83.e2.

162.	Forlani S, De Paulis R, Guerrieri Wolf L, Greco R, Polisca P, Moscarelli M, et al. Conversion to sinus rhythm by ablation improves quality of life in patients submitted to mitral valve surgery. Ann Thorac Surg 2006;81:863-7.

163.	Forleo GB, Mantica M, De Luca L, Leo R, Santini L, Panigada S, et al. Catheter ablation of atrial fibrillation in patients with diabetes mellitus type 2: results from a randomized study comparing pulmonary vein isolation versus antiarrhythmic drug therapy. J Cardiovasc Electrophysiol 2009;20:22-8.

164.	Forleo GB, Tondo C, De Luca L, Dello Russo A, Casella M, De Sanctis V, et al. Gender-related differences in catheter ablation of atrial fibrillation. Europace 2007;9:613-20.

165.	Foster-Witassek F, Aebersold H, Aeschbacher S, Ammann P, Beer JH, Blozik E, et al. Longitudinal Changes in Health-Related Quality of Life in Patients With Atrial Fibrillation. J Am Heart Assoc 2023;12:e031872.

166.	Frasure-Smith N, Lespérance F, Habra M, Talajic M, Khairy P, Dorian P, et al. Elevated depression symptoms predict long-term cardiovascular mortality in patients with atrial fibrillation and heart failure. Circulation 2009;120:134-40.

167.	Frasure-Smith N, Lesperance F, Talajic M, Khairy P, Dorian P, O'Meara E, et al. Anxiety sensitivity moderates prognostic importance of rhythm-control versus rate-control strategies in patients with atrial fibrillation and congestive heart failure: insights from the Atrial Fibrillation and Congestive Heart Failure Trial. Circ Heart Fail 2012;5:322-30.

168.	Frausing M, Van De Lande M, Linz D, Crijns H, Tieleman RG, Hemels MEW, et al. Healthcare utilisation and quality of life according to atrial fibrillation burden, episode frequency and duration. Heart 2024;110:1030-9.

169.	Fujisawa T, Kimura T, Kohsaka S, Ikemura N, Katsumata Y, Miyama H, et al. Symptom burden and treatment perception in patients with atrial fibrillation, with and without a family history of atrial fibrillation. Heart Vessels 2021;36:267-76.

170.	Fumagalli S, Cardini F, Roberts A, Boni S, Gabbai D, Calvani S, et al. Psychological effects of treatment with new oral anticoagulants in elderly patients with atrial fibrillation: a preliminary report. Aging Clin Exp Res 2015;27:99-102.

171.	Fumagalli S, Pelagalli G, Franci Montorzi R, Li K-M, Chang M-S, Chuang S-C, et al. Atrial fibrillation in older patients and artificial intelligence: a quantitative demonstration of a link with some of the geriatric multidimensional assessment tools—a preliminary report. Aging Clin Exp Res 2021;33:451-5.

172.	Fumagalli S, Pelagalli G, Montorzi RF, Marozzi I, Migliorini M, D'Andria MF, et al. The CHA(2)DS(2)-VASc score and Geriatric Multidimensional Assessment tools in elderly patients with persistent atrial fibrillation undergoing electrical cardioversion. A link with arrhythmia relapse? Eur J Intern Med 2020;82:56-61.

173.	Fumagalli S, Ricciardi G, Di Serio C, La Marca G, Pieraccini G, Franci Montorzi R, et al. Inflammation, mitochondrial dysfunction and physical performance: a possible association in older patients with persistent atrial fibrillation-the results of a preliminary study. Aging Clin Exp Res 2023;35:2831-7.

174.	Furlanello F, Lupo P, Pittalis M, Foresti S, Vitali-Serdoz L, Francia P, et al. Radiofrequency catheter ablation of atrial fibrillation in athletes referred for disabling symptoms preventing usual training schedule and sport competition. J Cardiovasc Electrophysiol 2008;19:457-62.

175.	Gabilondo M, Loza J, Pereda A, Caballero O, Zamora N, Gorostiza A, et al. Quality of life in patients with nonvalvular atrial fibrillation treated with oral anticoagulants. Hematology 2021;26:277-83.

176.	Gagné M, Legault C, Boulet LP, Charbonneau L, Lemyre M, Giguere AMC, et al. Impact of adding a video to patient education on quality of life among adults with atrial fibrillation: a randomized controlled trial. Patient Educ Couns 2019;102:1490‐8.

177.	Gaita F, Scaglione M, Battaglia A, Matta M, Gallo C, Galatà M, et al. Very long-term outcome following transcatheter ablation of atrial fibrillation. Are results maintained after 10 years of follow up? Europace 2018;20:443-50.

178.	Gallagher C, Orchard J, Nyfort-Hansen K, Sanders P, Neubeck L, Hendriks JM. NursE led Atrial Fibrillation Management: The NEAT Study: A Randomized Controlled Trial. J Cardiovasc Nurs 2020;35:456-67.

179.	Galli F, Borghi L, Faioni E, Cavicchioli M, Ferrari Losi J, Vegni E. Failure of the anticoagulant therapy and psychological distress: Still far from a bridge. Front Psychol 2018;9:1709.

180.	García-Izquierdo E, Fajardo-Simón L, Cruz-Utrilla A, Aguilera-Agudo C, Jiménez-Sánchez D, Sánchez-García M, et al. The Role of Anxiety in Patients With Persistent Atrial Fibrillation Undergoing Elective Cardioversion: An Observational Study. Psychosom Med 2020;82:744-50.

181.	Garimella RS, Chung EH, Mounsey JP, Schwartz JD, Pursell I, Gehi AK. Accuracy of patient perception of their prevailing rhythm: a comparative analysis of monitor data and questionnaire responses in patients with atrial fibrillation. Heart Rhythm 2015;12:658-65.

182.	Garimella RS, Sears SF, Gehi AK. Depression and Physical Inactivity as Confounding the Effect of Obesity on Atrial Fibrillation. Am J Cardiol 2016;117:1760-4.

183.	Gatti C, Parato VM, Guarnieri F, Rocchi R, Traini T, Galli A, et al. Impatto degli eventi avversi correlati alla fibrillazione atriale e proposte di strategie infermieristiche limitanti: studio di una realtà italiana. It J Nurs 2023:28-35.

184.	Gehi AK, Sears S, Goli N, Walker TJ, Chung E, Schwartz J, et al. Psychopathology and symptoms of atrial fibrillation: implications for therapy. J Cardiovasc Electrophysiol 2012;23:473-8.

185.	Genbrugge C, Jorissen E, Eertmans W, Jans F, Boer W, Dens J, et al. Increase in regional cerebral saturation after elective electrical cardioversion of atrial fibrillation is only transient and without beneficial effects on neuropsychological functioning: cerebral saturation during electrical cardioversion. J Clin Monit Comput 2021;35:165-73.

186.	Gerstenfeld EP, Guerra P, Sparks PB, Hattori K, Lesh MD. Clinical outcome after radiofrequency catheter ablation of focal atrial fibrillation triggers. J Cardiovasc Electrophysiol 2001;12:900-8.

187.	Gilbert KA, Hogarth AJ, MacDonald W, Lewis NT, Tan LB, Tayebjee MH. Restoration of sinus rhythm results in early and late improvements in the functional reserve of the heart following direct current cardioversion of persistent AF: FRESH-AF. Int J Cardiol 2015;199:121-5.

188.	Gill J, Crossen KJ, Blauth C, Kerendi F, Oza SR, Magnano AR, et al. Atrial fibrillation symptom reduction and improved quality of life following the hybrid convergent procedure: a CONVERGE trial subanalysis. Ann Cardiothorac Surg  2024;13:155-64.

189.	Gisi B, Althouse AD, Mathier AS, Pusateri A, Rollman BL, LaRosa A, et al. The unmeasured burden: Contribution of depression and psychological stress to patient-reported outcomes in atrial fibrillation. Int J Cardiol 2020;302:75-80.

190.	Gleason KT, Dennison Himmelfarb CR, Ford DE, Lehmann H, Samuel L, Jain S, et al. Association of sex and atrial fibrillation therapies with patient-reported outcomes. Heart 2019;105:1642-8.

191.	Goebel N, Brandel-Ursulescu C, Tanriverdi S, Franke UF. Partial upper sternotomy for concomitant left atrial ablation and aortic valve replacement. J Cardiovasc Surg (Torino) 2021;62:87-94.

192.	Goette A, Benninger G, Pittrow D, Paar WD, von Stritzky B, Bosch RF. One-year safety and quality of life outcomes in patients with atrial fibrillation on dronedarone: prospective, non-interventional study in German ambulatory care. Herzschrittmacherther Elektrophysiol 2015;26:148-54.

193.	Goette A, Borof K, Breithardt G, Camm AJ, Crijns H, Kuck KH, et al. Presenting Pattern of Atrial Fibrillation and Outcomes of Early Rhythm Control Therapy. J Am Coll Cardiol 2022;80:283‐95.

194.	Goette A, Schon N, Kirchhof P, Breithardt G, Fetsch T, Hausler KG, et al. Angiotensin II-antagonist in paroxysmal atrial fibrillation (ANTIPAF) trial. Circ Arrhythm Electrophysiol 2012;5:43-51.

195.	Goldberg A, Menen M, Mickelsen S, MacIndoe C, Binder M, Nawman R, et al. Atrial fibrillation ablation leads to long-term improvement of quality of life and reduced utilization of healthcare resources. J Interv Card Electrophysiol 2003;8:59-64.

196.	Goli NM, Thompson T, Sears SF, Mounsey JP, Chung E, Schwartz J, et al. Educational Attainment Is Associated with Atrial Fibrillation Symptom Severity. Pacing Clin Electrophysiol 2012;35:1090-6.

197.	Golwala H, Jackson LR, 2nd, Simon DN, Piccini JP, Gersh B, Go AS, et al. Racial/ethnic differences in atrial fibrillation symptoms, treatment patterns, and outcomes: Insights from Outcomes Registry for Better Informed Treatment for Atrial Fibrillation Registry. Am Heart J 2016;174:29-36.

198.	Goren A, Liu X, Gupta S, Simon TA, Phatak H. Quality of life, activity impairment, and healthcare resource utilization associated with atrial fibrillation in the US National Health and Wellness Survey. PLoS One 2013;8:e71264.

199.	Gorzelak-Pabiś P, Zyzak S, Krewko Ł, Broncel M. Assessment of the mean time in the therapeutic INR range and the SAME-TT2R2 score in patients with atrial fibrillation and cognitive impairment. Pol Arch Med Wewn 2016;126:494-501.

200.	Groenveld HF, Crijns HJ, Rienstra M, Van den Berg MP, Van Veldhuisen DJ, Van Gelder IC. Does intensity of rate control influence outcome in persistent atrial fibrillation? Data of the RACE study. Am Heart J 2009;158:785-91.

201.	Groenveld HF, Crijns HJ, Van den Berg MP, Van Sonderen E, Alings AM, Tijssen JG, et al. The effect of rate control on quality of life in patients with permanent atrial fibrillation: data from the RACE II (Rate Control Efficacy in Permanent Atrial Fibrillation II) study. J Am Coll Cardiol 2011;58:1795-803.

202.	Grönefeld GC, Lilienthal J, Kuck KH, Hohnloser SH. Impact of rate versus rhythm control on quality of life in patients with persistent atrial fibrillation. Results from a prospective randomized study. Eur Heart J 2003;24:1430-6.

203.	Gu J, Liu X, Tan H, Zhou L, Jiang W, Wang Y, et al. Impact of chronic obstructive pulmonary disease on procedural outcomes and quality of life in patients with atrial fibrillation undergoing catheter ablation. J Cardiovasc Electrophysiol 2013;24:148-54.

204.	Guedon-Moreau L, Capucci A, Denjoy I, Morgan CC, Perier A, Leplege A, et al. Impact of the control of symptomatic paroxysmal atrial fibrillation on health-related quality of life. Europace 2010;12:634-42.

205.	Guhl E, Althouse A, Sharbaugh M, Pusateri AM, Paasche-Orlow M, Magnani JW. Association of income and health-related quality of life in atrial fibrillation. Open Heart 2019;6:e000974.

206.	Guhl EN, Schlusser CL, Henault LE, Bickmore TW, Kimani E, Paasche-Orlow MK, et al. Rationale and design of the Atrial Fibrillation health Literacy Information Technology Trial: (AF-LITT). Contemp Clin Trials 2017;62:153-8.

207.	Gullón A, Formiga F, Díez-Manglano J, Mostaza JM, Cepeda JM, Pose A, et al. Influence of frailty on anticoagulant prescription and clinical outcomes after 1-year follow-up in hospitalised older patients with atrial fibrillation. Intern Emerg Med 2019;14:59-69.

208.	Güneş F, Boyraz S. Effect of atrial fibrillation on quality of life (AFEQT) questionnaire: A Turkish validity and reliability study. Turk Kardiyol Dern Ars 2021;49:223-32.

209.	Guo Y, Chen Y, Lane DA, Liu L, Wang Y, Lip GYH. Mobile Health Technology for Atrial Fibrillation Management Integrating Decision Support, Education, and Patient Involvement: mAF App Trial. Am J Med 2017;130:1388-96.e6.

210.	Gupta D, Earley MJ, Haywood GA, Richmond L, Fitzgerald M, Kojodjojo P, et al. Can atrial fibrillation with a coarse electrocardiographic appearance be treated with catheter ablation of the tricuspid valve-inferior vena cava isthmus? Results of a multicentre randomised controlled trial. Heart 2007;93:688-93.

211.	Gupta D, Vijgen J, Potter T, Scherr D, Van Herendael H, Knecht S, et al. Quality of life and healthcare utilisation improvements after atrial fibrillation ablation. Heart 2021;107:1296-302.

212.	Ha AC, Breithardt G, Camm AJ, Crijns HJ, Fitzmaurice GM, Kowey PR, et al. Health-related quality of life in patients with atrial fibrillation treated with rhythm control versus rate control: insights from a prospective international registry (Registry on Cardiac Rhythm Disorders Assessing the Control of Atrial Fibrillation: RECORD-AF). Circ Cardiovasc Qual Outcomes 2014;7:896-904.

213.	Ha ACT, Stewart J, Klein G, Roy D, Connolly S, Koren A, et al. Impact of electrical cardioversion on quality of life for patients with symptomatic persistent atrial fibrillation: Is there a treatment expectation effect? Am Heart J 2020;226:152-60.

214.	Haeusler KG, Eichner FA, Heuschmann PU, Fiebach JB, Engelhorn T, Blank B, et al. MRI-Detected Brain Lesions and Cognitive Function in Patients With Atrial Fibrillation Undergoing Left Atrial Catheter Ablation in the Randomized AXAFA-AFNET 5 Trial. Circulation 2022;145:906-15.

215.	Hagens VE, Crijns HJ, Van Veldhuisen DJ, Van Den Berg MP, Rienstra M, Ranchor AV, et al. Rate control versus rhythm control for patients with persistent atrial fibrillation with mild to moderate heart failure: results from the RAte Control versus Electrical cardioversion (RACE) study. Am Heart J 2005;149:1106-11.

216.	Hagens VE, Ranchor AV, Van Sonderen E, Bosker HA, Kamp O, Tijssen JG, et al. Effect of rate or rhythm control on quality of life in persistent atrial fibrillation. Results from the Rate Control Versus Electrical Cardioversion (RACE) Study. J Am Coll Cardiol 2004;43:241-7.

217.	Haldar S, Khan HR, Boyalla V, Kralj-Hans I, Jones S, Lord J, et al. Catheter ablation vs. thoracoscopic surgical ablation in long-standing persistent atrial fibrillation: CASA-AF randomized controlled trial. Eur Heart J 2020;41:4471-80.

218.	Hamer ME, Blumenthal JA, McCarthy EA, Phillips BG, Pritchett EL. Quality-of-life assessment in patients with paroxysmal atrial fibrillation or paroxysmal supraventricular tachycardia. Am J Cardiol 1994;74:826-9.

219.	Hämmerle P, Aeschbacher S, Springer A, Eken C, Coslovsky M, Dutilh G, et al. Cardiac autonomic function and cognitive performance in patients with atrial fibrillation. Clin Res Cardiol 2022;111:60-9.

220.	Haman L, Dostalova H, Parizek P. Catheter ablation for atrial fibrillation—Single center experience. Cor Et Vasa 2012;54:e369-74.

221.	Han G, Fu T, Zhang Y. Effects of Moracizine Combined with Metoprolol on Hemodynamic Indices of the Left Atrium and Quality of Life in Patients with Atrial Fibrillation. Heart Surg Forum 2023;26:e463-9.

222.	Hanon O, Chaussade E, Gueranger P, Gruson E, Bonan S, Gay A. Patient-Reported Treatment Satisfaction with Rivaroxaban for Stroke Prevention in Atrial Fibrillation. A French Observational Study, the SAFARI Study. PLoS One 2016;11:e0166218.

223.	Hanon O, Vidal JS, Le Heuzey JY, Kirchhof P, De Caterina R, Schmitt J, et al. Oral anticoagulant use in octogenarian European patients with atrial fibrillation: A subanalysis of PREFER in AF. Int J Cardiol 2017;232:98-104.

224.	Harden M, Nystrom B, Bengtson A, Medin J, Frison L, Edvardsson N. Responsiveness of AF6, a new, short, validated, atrial fibrillation-specific questionnaire--symptomatic benefit of direct current cardioversion. J Interv Card Electrophysiol 2010;28:185-91.

225.	Harden M, Nystrom B, Kulich K, Carlsson J, Bengtson A, Edvardsson N. Validity and reliability of a new, short symptom rating scale in patients with persistent atrial fibrillation. Health Qual Life Outcomes 2009;7:65.

226.	Hasebe H, Shinba T. Decreased anxiety after catheter ablation for paroxysmal atrial fibrillation is associated with augmented parasympathetic reactivity to stress. Heart Rhythm O2 2020;1:189-99.

227.	Hashiba M, Rasmussen TB, Lamberts MK, Nielsen SK, Risom SS. Validation of the Danish translation of the atrial fibrillation severity scale: a study on linguistic and cultural adaptation. Health Qual Life Outcomes. 2025;23:36.

228.	Hashimoto N, Arimoto T, Koyama K, Kutsuzawa D, Watanabe K, Aita S, et al. Improved cerebral blood flow and hippocampal blood flow in stroke‐free patients after catheter ablation of atrial fibrillation. J Cardiovasc Electrophysiol 2023;34:2055-64.

229.	He Y, Tang Z, Sun G, Cai C, Wang Y, Yang G, et al. Effectiveness of a Mindfulness Meditation App Based on an Electroencephalography-Based Brain-Computer Interface in Radiofrequency Catheter Ablation for Patients With Atrial Fibrillation: Pilot Randomized Controlled Trial. JMIR Mhealth Uhealth 2023;11:e44855.

230.	Hedberg B, Malm D, Karlsson JE, Arestedt K, Brostrom A. Factors associated with confidence in decision making and satisfaction with risk communication among patients with atrial fibrillation. Eur J Cardiovasc Nurs 2018;17:446-55.

231.	Hemels ME, Gu YL, Tuinenburg AE, Boonstra PW, Wiesfeld AC, van den Berg MP, et al. Favorable long-term outcome of Maze surgery in patients with lone atrial fibrillation. Ann Thorac Surg 2006;81:1773-9.

232.	Hemels ME, Ruiter JH, Molhoek GP, Veeger NJ, Wiesfeld AC, Ranchor AV, et al. Right atrial preventive and antitachycardia pacing for prevention of paroxysmal atrial fibrillation in patients without bradycardia: a randomized study. Europace 2008;10:306-13.

233.	Hegbom F, Sire S, Heldal M, Orning OM, Stavem K, Gjesdal K. Short-term exercise training in patients with chronic atrial fibrillation: effects on exercise capacity, AV conduction, and quality of life. J Cardiopulm Rehabil 2006;26:24-9.

234.	Hendriks JM, de Wit R, Vrijhoef HJ, Tieleman RG, Crijns HJ. An integrated chronic care program for patients with atrial fibrillation: study protocol and methodology for an ongoing prospective randomised controlled trial. Int J Nurs Stud 2010;47:1310-6.

235.	Hendriks J, Tomini F, van Asselt T, Crijns H, Vrijhoef H. Cost-effectiveness of a specialized atrial fibrillation clinic vs. usual care in patients with atrial fibrillation. Europace 2013;15:1128-35.

236.	Hendriks JM, Vrijhoef HJ, Crijns HJ, Brunner-La Rocca HP. The effect of a nurse-led integrated chronic care approach on quality of life in patients with atrial fibrillation. Europace 2014;16:491-9.

237.	Henry L, Hunt S, Holmes SD, Martin LM, Ad N. Are there gender differences in outcomes after the Cox-Maze procedure for atrial fibrillation? Innovations (Phila) 2013;8:190-8.

238.	Herber E, Aeschbacher S, Coslovsky M, Schwendinger F, Hennings E, Gasser A, et al. Physical activity and brain health in patients with atrial fibrillation. Eur J Neurol 2023;30:567-77.

239.	Hickey KT, Hauser NR, Valente LE, Riga TC, Frulla AP, Masterson Creber R, et al. A single-center randomized, controlled trial investigating the efficacy of a mHealth ECG technology intervention to improve the detection of atrial fibrillation: the iHEART study protocol. BMC Cardiovasc Disord 2016;16:152.

240.	Ho JC, Chang AM, Yan BP, Yu CM, Lam YY, Lee VW. Dabigatran compared with warfarin for stroke prevention with atrial fibrillation: experience in Hong Kong. Clin Cardiol 2012;35:e40-5.

241.	Hoegh V, Lundbye-Christensen S, Delmar C, Frederiksen K, Riahi S, Overvad K. Association between the diagnosis of atrial fibrillation and aspects of health status: a Danish cross-sectional study. Scand J Caring Sci 2016;30:507-17.

242.	Hoglund N, Ronn F, Tollefsen T, Jensen SM, Kesek M. U22 protocol as measure of symptomatic improvement after catheter ablation of atrial fibrillation. Ups J Med Sci 2013;118:240-6.

243.	Holmes DN, Piccini JP, Allen LA, Fonarow GC, Gersh BJ, Kowey PR, et al. Defining Clinically Important Difference in the Atrial Fibrillation Effect on Quality-of-Life Score. Circ Cardiovasc Qual Outcomes 2019;12:e005358.

244.	Holmlund L, Hornsten C, Valham F, Olsson K, Hornsten A, Angerud KH. Illness Perceptions and Health-Related Quality of Life in Women and Men With Atrial Fibrillation. J Cardiovasc Nurs 2024;39:49-57.

245.	Holmlund L [a], Hörnsten C, Hörnsten Å, Olsson K, Valham F, Ängerud KH. More positive patient-reported outcomes in patients newly diagnosed with atrial fibrillation: a comparative longitudinal study. Eur J Cardiovasc Nurs 2024;23:618-26.

246.	Howes CJ, Reid MC, Brandt C, Ruo B, Yerkey MW, Prasad B, et al. Exercise tolerance and quality of life in elderly patients with chronic atrial fibrillation. J Cardiovasc Pharmacol Ther 2001;6:23-9.

247.	Hsieh HL, Kao CW, Cheng SM, Chang YC. A Web-Based Integrated Management Program for Improving Medication Adherence and Quality of Life, and Reducing Readmission in Patients With Atrial Fibrillation: Randomized Controlled Trial. J Med Internet Res 2021;23:e30107.

248.	Hsu NW, Tsao HM, Chen HC, Lo SS, Chen SA, Chou P. Different Impacts of Atrial Fibrillation and Cardiac Premature Contractions on the Health-Related Quality of Life in Elderly People: The Yilan Study. Tohoku J Exp Med 2016;238:75-83.

249.	Hsu LF, Jais P, Sanders P, Garrigue S, Hocini M, Sacher F, et al. Catheter ablation for atrial fibrillation in congestive heart failure. N Eng J Med 2004;351:2373-83.

250.	Humphries B, Cox JL, Parkash R, Thabane L, Foster GA, MacKillop J, et al. Patient-Reported Outcomes and Patient-Reported Experience of Patients With Atrial Fibrillation in the IMPACT-AF Clinical Trial. J Am Heart Assoc 2021;10:e019783.

251.	Hu CL, Jiang H, Tang QZ, Zhang QH, Chen JB, Huang CX, et al. Comparison of rate control and rhythm control in patients with atrial fibrillation after percutaneous mitral balloon valvotomy: a randomised controlled study. Heart 2006;92:1096-101.

252.	Huang H, Liu Y, Shuai W, Jiang C, Zhang M, Qu X, et al. Atrial tachyarrhythmia prevention by Shensong Yangxin after catheter ablation for persistent atrial fibrillation: the SS-AFRF trial. Eur Heart J 2024;45:4305‐14.

253.	Huang T, Xie H, Ma N. Clinical efficacy of radiofrequency ablation guided by high-density mapping on persistent atrial fibrillation. Cir Cir 2024;92:715-23.

254.	Hussein AA, Delaughter MC, Monir G, Natale A, Dukkipati S, Oza S, et al. Paroxysmal atrial fibrillation ablation with a novel temperature-controlled CF-sensing catheter: Q-FFICIENCY clinical and healthcare utilization benefits. J Cardiovasc Electrophysiol 2023;34:2493-503.

255.	Hussein AA, Lindsay B, Madden R, Martin D, Saliba WI, Tarakji KG, et al. New Model of Automated Patient-Reported Outcomes Applied in Atrial Fibrillation. Circ Arrhythm Electrophysiol 2019;12:e006986.

256.	Hutchings HA, Lanyon K, Lister S, Alikhan R, Halcox J, Holland G, et al. Investigating the feasibility of recruitment to an observational, quality-of-life study of patients diagnosed with atrial fibrillation (AF) who have experienced a bleed while anticoagulated: EQUAL-AF feasibility study protocol. Pilot Feasibility Stud 2022;8:180.

257.	Hutchings HA, Lanyon KJ, Holland G, Alikhan R, Jenkins R, Laing H, et al. Can we collect health-related quality of life information from anticoagulated atrial fibrillation participants who have recently experienced a bleed? An observational feasibility study in primary and secondary care in Wales and through a UK online forum. BMJ Open 2023;13:e075335.

258.	Ibáñez JL, Ibáñez A, Barrio T, Ortiz M, Ajo R, Castellanos E, et al. Lessons learned in attempting catheter-based interatrial electrical disconnection for nonpharmacologic rate control of atrial fibrillation or flutter. J Interv Card Electrophysiol 2020;57:333-43.

259.	Ikemura N, Kimura T, Sawano M, Fukuda K, Takatsuki S, Spertus JA, et al. Novel Approach for Visualizing Multiple Domains of Quality of Life Scales. Circ Cardiovasc Qual Outcomes 2019;12:e005573.

260.	Ikemura N [a], Spertus JA, Kimura T, Mahaffey K, Piccini JP, Inohara T, et al. Cohort profile: patient characteristics and quality-of-life measurements for newly-referred patients with atrial fibrillation-Keio interhospital Cardiovascular Studies-atrial fibrillation (KiCS-AF). BMJ Open 2019;9:e032746.

261.	Ikemura N, Kohsaka S, Kimura T, Jones PG, Katsumata Y, Tanimoto K, et al. Physician Estimates and Patient-Reported Health Status in Atrial Fibrillation. JAMA Netw Open 2024;7:e2356693.

262.	Inagaki D, Fukamizu S, Tokioka S, Kawamura I, Kitamura T, Hojo R, et al. Quality of life improvements by durable pulmonary vein isolation in patients with atrial fibrillation. J Cardiovasc Electrophysiol 2020;31:2013-21.

263.	Iroegbu CD, Chen W, Wu X, Cheng L, Zhang H, Wu M, et al. Evaluating the cost-effectiveness of catheter ablation of atrial fibrillation. Cardiovasc Diagn Ther 2020;10:1200-15.

264.	Ishiguro H, Ikeda T, Abe A, Tsukada T, Mera H, Nakamura K, et al. Antiarrhythmic effect of bisoprolol, a highly selective beta1-blocker, in patients with paroxysmal atrial fibrillation. Int Heart J 2008;49:281-93.

265.	Jeong HK, Cho JG, Lee KH, Park HW, Kim MR, Lee KJ, et al. Determinants of quality of life in patients with atrial fibrillation. Int J Cardiol 2014;172:e300-2.

266.	Jackson A, Frobert O, Boye Larsen D, Arendt-Nielsen L, Björkenheim A. Patients with symptomatic permanent atrial fibrillation show quantitative signs of pain sensitisation. Open Heart 2021;8:e001699.

267.	Jain SK, Novak PG, Sangrigoli R, Champagne J, Dubuc M, Adler SW, et al. Sustained quality-of-life improvement post-cryoballoon ablation in patients with paroxysmal atrial fibrillation: Results from the STOP-AF Post-Approval Study. Heart Rhythm 2020;17:485-91.

268.	Jaïs P, Cauchemez B, Macle L, Daoud E, Khairy P, Subbiah R, et al. Catheter ablation versus antiarrhythmic drugs for atrial fibrillation: the A4 study. Circulation 2008;118:2498-505.

269.	Jankowska-Polanska B, Kaczan A, Lomper K, Nowakowski D, Dudek K. Symptoms, acceptance of illness and health-related quality of life in patients with atrial fibrillation. Eur J Cardiovasc Nurs 2018;17:262-72.

270.	Jankowska-Polańska B, Katarzyna L, Lidia A, Joanna J, Dudek K, Izabella U. Cognitive function and adherence to anticoagulation treatment in patients with atrial fibrillation. J Geriatr Cardiol 2016;13:559-65.

271.	Jansson V, Bergfeldt L, Schwieler J, Kenneback G, Rubulis A, Jensen SM, et al. Atrial fibrillation burden, episode duration and frequency in relation to quality of life in patients with implantable cardiac monitor. Int J Cardiol Heart Vasc 2021;34:100791.

272.	Jasper SE, Lieber EA, Murray RD, Apperson-Hansen C, Black IW, Grimm RA, et al. Impact of cardioversion strategy on functional capacity in patients with atrial fibrillation: the Assessment of Cardioversion Using Transesophageal Echocardiography (ACUTE) study. Am Heart J 2005;149:309-15.

273.	Jenkins LS, Brodsky M, Schron E, Chung M, Rocco T, Jr., Lader E, et al. Quality of life in atrial fibrillation: the Atrial Fibrillation Follow-up Investigation of Rhythm Management (AFFIRM) study. Am Heart J 2005;149:112-20.

274.	Jessurun ER, van Hemel NM, Defauw JJ, Brutel De La Riviere A, Stofmeel MA, Kelder JC, et al. A randomized study of combining maze surgery for atrial fibrillation with mitral valve surgery. J Cardiovasc Surg (Torino) 2003;44:9-18.

275.	Ji-Yeong S, Hyeon-Ok L. The Impact of Self-Management Behaviors, Self-Efficacy, and Grit on Health-related Quality of Life in Patients with Atrial Fibrillation: A Cross-Sectional Descriptive Study. Korean J Adult Nurs 2023;35:158-68.

276.	Jia Z, Du X, Du J, Xia S, Guo L, Su X, et al. Prevalence and factors associated with depressive and anxiety symptoms in a Chinese population with and without cardiovascular diseases. J Affect Disord 2021;286:241-7.

277.	Jin MN, Kim TH, Kang KW, Yu HT, Uhm JS, Joung B, et al. Atrial Fibrillation Catheter Ablation Improves 1-Year Follow-Up Cognitive Function, Especially in Patients With Impaired Cognitive Function. Circ Arrhythm Electrophysiol 2019;12:e007197.

278.	Joensen AM, Dinesen PT, Svendsen LT, Hoejbjerg TK, Fjerbaek A, Andreasen J, et al. Effect of patient education and physical training on quality of life and physical exercise capacity in patients with paroxysmal or persistent atrial fibrillation: A randomized study. J Rehabil Med 2019;51:442-50.

279.	Johansson B, Houltz B, Berglin E, Brandrup-Wognsen G, Karlsson T, Edvardsson N. Short-term sinus rhythm predicts long-term sinus rhythm and clinical improvement after intraoperative ablation of atrial fibrillation. Europace 2008;10:610-7.

280.	Johnson BM, Wazni OM, Farwati M, Saliba WI, Santangeli P, Madden R, et al. Atrial Fibrillation Ablation in Young Adults: Measuring Quality of Life Using Patient-Reported Outcomes Over 5 Years. Circ Arrhythm Electrophysiol 2023;16:e011565.

281.	Jones J, Stanbury M, Haynes S, Bunting KV, Lobban T, Camm AJ, et al. Importance and Assessment of Quality of Life in Symptomatic Permanent Atrial Fibrillation: Patient Focus Groups from the RATE-AF Trial. Cardiology 2020;145:666-75.

282.	Jons C, Hansen PS, Johannessen A, Hindricks G, Raatikainen P, Kongstad O, et al. The Medical ANtiarrhythmic Treatment or Radiofrequency Ablation in Paroxysmal Atrial Fibrillation (MANTRA-PAF) trial: clinical rationale, study design, and implementation. Europace 2009;11:917-23.

283.	Joosten LPT, van Doorn S, Hoes AW, Nierman MC, Wiersma NM, Koek HL, et al. Safety of switching from Vitamin K antagonist to non-Vitamin K antagonist oral anticoagulant in frail elderly with atrial fibrillation: rationale and design of the FRAIL-AF randomised controlled trial. BMJ Open 2019;9:e032488.

284.	Joshibayev S, Bolatbekov B. Early and long-term outcomes and quality of life after concomitant mitral valve surgery, left atrial size reduction, and radiofrequency surgical ablation of atrial fibrillation. Anatol J Cardiol 2016;16:797-803.

285.	Ju Youn K, Hyoung-Seob P, Hyung Wook P, Eue-Keun C, Jin-Kyu P, Jin-Bae K, et al. Clinical Outcomes of Rhythm Control Strategies for Asymptomatic Atrial Fibrillation According to the Quality-of-Life Score: The CODE-AF (Comparison Study of Drugs for Symptom Control and Complication Prevention of Atrial Fibrillation) Registry. J Am Heart Assoc 2022;11:e025956.

286.	Kanthasamy V, Schilling R, Zongo O, Khan K, Earley M, Monk V, et al. Feasibility of double-blinded, placebo-controlled interventional study for assessing catheter ablation efficacy in persistent atrial fibrillation: Insights from the ORBITA AF feasibility study. Am Heart J 2024;269:56-71.

287.	Kahya Eren N, Yakar Tuluce S, Kilicaslan B, Nazli C, Ergene O. The validity and reliability of the Turkish version of the University of Toronto Atrial Fibrillation Severity Scale. Turk J Med Sci 2014;44:996-1001.

288.	Kale M, Bennett DH. Atrial septal pacing in the prevention of paroxysmal atrial fibrillation refractory to antiarrhythmic drugs. Int J Cardiol 2002;82:167-75.

289.	Kamalvand K, Tan K, Kotsakis A, Bucknall C, Sulke N. Is mode switching beneficial? A randomized study in patients with paroxysmal atrial tachyarrhythmias. J Am Coll Cardiol 1997;30:496-504.

290.	Kanagaratnam P, McCready J, Tayebjee M, Shepherd E, Sasikaran T, Todd D, et al. Ablation versus anti-arrhythmic therapy for reducing all hospital episodes from recurrent atrial fibrillation: a prospective, randomized, multi-centre, open label trial. Europace 2023;25:863-72.

291.	Kang Y. Effects of uncertainty on perceived health status in patients with atrial fibrillation. Nurs Crit Care 2005;10:184-91.

292.	Kang Y. Gender and culture differences in the quality of life among Americans and Koreans with atrial fibrillation. Nurs Health Sci 2009;11:301-5.

293.	Kang Y. The relationships between uncertainty and its antecedents in Korean patients with atrial fibrillation. J Clin Nurs 2011;20:1880-6.

294.	Kang Y, Bahler R. Health-related quality of life in patients newly diagnosed with atrial fibrillation. Eur J Cardiovasc Nurs 2004;3:71-6.

295.	Kato M, Miake J, Ogura K, Iitsuka K, Okamura A, Tomomori T, et al. Different Effects of Pulmonary Vein Isolation on Quality of Life Between Patients with Persistent and Paroxysmal Atrial Fibrillation. Int Heart J 2019;60:1328-33.

296.	Kato N, Muraga K, Hirata Y, Shindo A, Matsuura K, Ii Y, et al. Brain magnetic resonance imaging and cognitive alterations after ablation in patients with atrial fibrillation. Sci Rep 2021;11:18995.

297.	Katsumata Y, Kimura T, Kohsaka S, Ikemura N, Ueda I, Fujisawa T, et al. Discrepancy in recognition of symptom burden among patients with atrial fibrillation. Am Heart J 2020;226:240-9.

298.	Kay GN, Bubien RS, Epstein AE, Plumb VJ. Effect of catheter ablation of the atrioventricular junction on quality of life and exercise tolerance in paroxysmal atrial fibrillation. Am J Cardiol 1988;62:741-4.

299.	Kay GN, Ellenbogen KA, Giudici M, Redfield MM, Jenkins LS, Mianulli M, et al. The Ablate and Pace Trial: a prospective study of catheter ablation of the AV conduction system and permanent pacemaker implantation for treatment of atrial fibrillation. APT Investigators. J Interv Card Electrophysiol 1998;2:121-35.

300.	Khalifehzadeh-Esfahani A, Amirzadeh A, Golshahi J. Effect of a Care Plan on the Quality of Life of the Patients with Atrial Fibrillation. Iran J Nurs Midwifery Res 2018;23:277-80.

301.	Khan HR, Kralj-Hans I, Haldar S, Bahrami T, Clague J, De Souza A, et al. Catheter Ablation versus Thoracoscopic Surgical Ablation in Long Standing Persistent Atrial Fibrillation (CASA-AF): study protocol for a randomised controlled trial. Trials 2018;19:117.

302.	Khan TI, Kamali F, Kesteven P, Avery P, Wynne H. The value of education and self-monitoring in the management of warfarin therapy in older patients with unstable control of anticoagulation. Br J Haematol 2004;126:557-64.

303.	Kilander L, Andren B, Nyman H, Lind L, Boberg M, Lithell H. Atrial fibrillation is an independent determinant of low cognitive function: a cross-sectional study in elderly men. Stroke 1998;29:1816-20.

304.	Kim D, Shim J, Choi EK, Oh IY, Kim J, Lee YS, et al. The anticoagulation one year after ablation of atrial fibrillation in patients with atrial fibrillation (ALONE-AF) trial: Study protocol. Heliyon 2024;10:e36506.

305.	Kim S, Lee S, Han D, Jeong I, Lee HH, Koh Y, et al. One-year Aerobic Interval Training Improves Endothelial Dysfunction in Patients with Atrial Fibrillation: A Randomized Trial. Intern Med 2023;62:2465-74.

306.	Kimura M, Kobori A, Nitta J, Hirao K, Shizuta S, Kurita T, et al. Cryoballoon ablation for paroxysmal atrial fibrillation in Japan: 2-year safety and efficacy results from the Cryo AF Global Registry. J Interv Card Electrophysiol 2022;64:695-703.

307.	Kirchhof P, Breithardt G, Camm AJ, Crijns HJ, Kuck KH, Vardas P, et al. Improving outcomes in patients with atrial fibrillation: rationale and design of the Early treatment of Atrial fibrillation for Stroke prevention Trial. Am Heart J 2013;166:442-8.

308.	Kirchhof P, Camm AJ, Goette A, Brandes A, Eckardt L, Elvan A, et al. Early Rhythm-Control Therapy in Patients with Atrial Fibrillation. N Engl J Med 2020;383:1305-16.

309.	Kirchhof P, Haeusler KG, Blank B, De Bono J, Callans D, Elvan A, et al. Apixaban in patients at risk of stroke undergoing atrial fibrillation ablation. Eur Heart J 2018;39:2942-55.

310.	Kjolseth AJ, Norekval TM, Brors G, Hendriks JM, Risom SS, Rotevatn S, et al. Modifiable risk factors and self-reported health after percutaneous coronary intervention - with and without a history of atrial fibrillation. Eur J Cardiovasc Nurs 2025;24:58-68.

311.	Kloosterman M, Chua W, Fabritz L, Al-Khalidi HR, Schotten U, Nielsen JC, et al. Sex differences in catheter ablation of atrial fibrillation: results from AXAFA-AFNET 5. Europace 2020;22:1026-35.

312.	Koca M, Yavuz BB, Tuna Dogrul R, Caliskan H, Sengul Aycicek G, Ozsurekci C, et al. Impact of atrial fibrillation on frailty and functionality in older adults. Ir J Med Sci 2020;189:917-24.

313.	Kocheril AG, Calkins H, Sharma AD, Cher D, Stubbs HA, Block JE. Hybrid therapy with right atrial catheter ablation and previously ineffective antiarrhythmic drugs for the management of atrial fibrillation. J Interv Card Electrophysiol 2005;12:189-97.

314.	Kochhäuser S, Joza J, Essebag V, Proietti R, Koehler J, Tsang B, et al. The Impact of Duration of Atrial Fibrillation Recurrences on Measures of Health-Related Quality of Life and Symptoms. Pacing Clin Electrophysiol 2016;39:166-72.

315.	Koci F, Forbes P, Mansour MC, Heist EK, Singh JP, Ellinor PT, et al. New classification scheme for atrial fibrillation symptom severity and burden. Am J Cardiol 2014;114:260-5.

316.	Kooistra HA, Piersma-Wichers M, Kluin-Nelemans HC, Veeger NJ, Meijer K. Impact of Vitamin K Antagonists on Quality of Life in a Prospective Cohort of 807 Atrial Fibrillation Patients. Circ Cardiovasc Qual Outcomes 2016;9:388-94.

317.	Kosior DA, Szulc M, Rosiak M, Rabczenko D, Opolski G, For The Investigators Of The Hot Cafe Polish S. Functional status with rhythm- versus rate-control strategy for persistent atrial fibrillation. Pol Arch Intern Med 2018;128:658-66.

318.	Kosmalska K, Rzyman M, Miękus P, Gilis-Malinowska N, Nowak R, Fijałkowski M. Usefulness of transesophageal echocardiography before cardioversion in atrial arrhythmias. Cardiol J 2021;28:101-9.

319.	Kotalczyk A, Yutao G, Stefil M, Yutang W, Lip GYH, Guo Y, et al. Effects of the Atrial Fibrillation Better Care Pathway on Outcomes Among Clinically Complex Chinese Patients With Atrial Fibrillation With Multimorbidity and Polypharmacy: A Report From the ChiOTEAF Registry. J Am Heart Assoc 2022;11:1-10.

320.	Kotecha D, Bunting KV, Gill SK, Mehta S, Stanbury M, Jones JC, et al. Effect of Digoxin vs Bisoprolol for Heart Rate Control in Atrial Fibrillation on Patient-Reported Quality of Life: The RATE-AF Randomized Clinical Trial. JAMA 2020;324:2497-2508.

321.	Kotecha D, Calvert M, Deeks JJ, Griffith M, Kirchhof P, Lip GY, et al. A review of rate control in atrial fibrillation, and the rationale and protocol for the RATE-AF trial. BMJ Open 2017;7:e015099.

322.	Koutoukidis DA, Jones NR, Taylor CJ, Casadei B, Aveyard P. Obesity, self-reported symptom severity, and quality of life in people with atrial fibrillation: A community-based cross-sectional survey. Nutr Metab Cardiovasc Dis 2020;30:2221-9.

323.	Kowalewski M, Wańha W, Litwinowicz R, Kołodziejczak M, Pasierski M, Januszek R, et al. Stand-Alone Left Atrial appendage occlusion for throMboembolism prevention in nonvalvular Atrial fibrillatioN DiseasE Registry (SALAMANDER): protocol for a prospective observational nationwide study. BMJ Open 2022;12:e063990.

324.	Kristensen MS, Zwisler AD, Berg SK, Zangger G, Gronset CN, Risom SS, et al. Validating the HeartQoL questionnaire in patients with atrial fibrillation. Eur J Prev Cardiol 2016;23:1496-503.

325.	Kuck KH, Furnkranz A, Chun KR, Metzner A, Ouyang F, Schluter M, et al. Cryoballoon or radiofrequency ablation for symptomatic paroxysmal atrial fibrillation: reintervention, rehospitalization, and quality-of-life outcomes in the FIRE AND ICE trial. Eur Heart J 2016;37:2858-65.

326.	Kupper N, van den Broek K, Haagh E, van der Voort P, Widdershoven J, Denollet J. Type D personality affects health-related quality of life in patients with lone atrial fibrillation by increasing symptoms related to sympathetic activation. J Psychosom Res 2018;115:44-52.

327.	Kupper N, van den Broek KC, Widdershoven J, Denollet J. Subjectively reported symptoms in patients with persistent atrial fibrillation and emotional distress. Front Psychol 2013;4:192.

328.	Kwon JY, Sawatzky R, Baumbusch J, Ratner PA. Patient-reported outcomes and the identification of subgroups of atrial fibrillation patients: a retrospective cohort study of linked clinical registry and administrative data. Qual Life Res 2021;30:1547-59.

329.	Lakkireddy D, Atkins D, Pillarisetti J, Ryschon K, Bommana S, Drisko J, et al. Effect of yoga on arrhythmia burden, anxiety, depression, and quality of life in paroxysmal atrial fibrillation: the YOGA My Heart Study. J Am Coll Cardiol 2013;61:1177-82.

330.	Lai Y, Zhao M, Jiang C, Du X, Wang Z, Zhang J, et al. Validation of T-MoCA in the Screening of Mild Cognitive Impairment in Chinese Patients With Atrial Fibrillation. Front Cardiovasc Med 2022;9:896846.

331.	LaMori JC, Mody SH, Gross HJ, DiBonaventura M, Patel A, Schein J, et al. Dyspepsia and disease burden among patients with atrial fibrillation. Crit Pathw Cardiol 2012;11:14-9.

332.	Lane DA, Langman CM, Lip GYH, Nouwen A. Illness perceptions, affective response, and health-related quality of life in patients with atrial fibrillation. J Psychosom Res 2009;66:211-20.

333.	Lau CP, Tse HF, Yu CM, Teo WS, Kam R, Ng KS, et al. Dual-site atrial pacing for atrial fibrillation in patients without bradycardia. Am J Cardiol  2001;88:371‐5.

334.	Lawin D, Lawrenz T, Chun KRJ, Lim HE, Obidigbo V, Selma JM, et al. Cryoballoon ablation of atrial fibrillation in octogenarians: one year outcomes from the cryo global registry. J Interv Card Electrophysiol 2024;67:1341-51.

335.	Lazaridis C, Bakogiannis C, Mouselimis D, Tsarouchas A, Antoniadis AP, Triantafyllou K, et al. The usability and effect of an mHealth disease management platform on the quality of life of patients with paroxysmal atrial fibrillation - The emPOWERD-AF study. Health Informatics J 2022;28:14604582221139053.

336.	Lee K, Lee SK, Lee J, Jeon BK, Kim TH, Yu HT, et al. Protocol of BEYOND trial: clinical BEnefit of sodium-glucose cotransporter-2 (SGLT-2) inhibitors in rhYthm cONtrol of atrial fibrillation in patients with diabetes mellitus. PLoS One 2023;18:e0280359.

337.	Leiner J, Konig S, Mouratis K, Kim I, Schmitz P, Joshi T, et al. A Digital Infrastructure for Cardiovascular Patient Care Based on Mobile Health Data and Patient-Reported Outcomes: Concept Details of the Helios TeleWear Project Including Preliminary Experiences. JMIR Form Res 2023;7:e41115.

338.	Lelakowski J, Malecka B, Bednarek J, Bigaj J. [Left ventricular systolic function, paced QT dispersion, exercise tolerance and quality of life in long term follow up after ventricular pacemaker implantation (VVIR) and radiofrequency atrioventricular junction ablation in drug refractory atrial fibrillation]. Pol Merkur Lekarski 2008;24:190-4.

339.	Levy T, Walker S, Mason M, Spurrell P, Rex S, Brant S, et al. Importance of rate control or rate regulation for improving exercise capacity and quality of life in patients with permanent atrial fibrillation and normal left ventricular function: a randomised controlled study. Heart 2001;85:171-8.

340.	Li PWC, Yu DSF, Yan BBY. A nurse-coordinated integrated care model to support decision-making and self-care in patients with atrial fibrillation: A study protocol. J Adv Nurs 2019;75:3749-57.

341.	Li PWC, Yu DSF, Yan BP. Psychometric Validation of the Chinese Version of Atrial Fibrillation Effect on Quality-of-Life Questionnaire Among Chinese Patients. J Cardiovasc Nurs 2021;36:136-42.

342.	Li PWC, Yu DSF, Yan BP. Nurse-led multi-component behavioural activation programme to improve health outcomes in patients with atrial fibrillation: a mixed-methods study and feasibility analysis. Eur J Cardiovasc Nurs 2023;22:655-63.

343.	Li X, Li R, Xiao F, Zhao K, Zhang X, Wang X, et al. Validation of China Health Related Outcomes Measures-Cardiovascular Disease (CHROME-CVD). Value Health 2024;27:490-499.

344.	Li MT, Shi GX, Wang Y, Wu BQ, Zhang ZH, Zhao QY, et al. Effect of acupuncture on quality of life in atrial fibrillation: study protocol for a randomised controlled trial. BMJ Open 2024;14:e087460.

345.	Li YM, Jiang C, He L, Li XX, Hou XX, Chang SS, et al. Sex Differences in Presentation, Quality of Life, and Treatment in Chinese Atrial Fibrillation Patients: Insights from the China Atrial Fibrillation Registry Study. Med Sci Monit 2019;25:8011-8.

346.	Lim KT, Davis MJ, Powell A, Arnolda L, Moulden K, Bulsara M, et al. Ablate and pace strategy for atrial fibrillation: long-term outcome of AIRCRAFT trial. Europace 2007;9:498-505.

347.	Lin JL, Wu TJ, Chen CP, Hsu JC, Ueng KC, Kuo JY, et al. Observational study of dronedarone in Taiwanese patients with atrial fibrillation. J Formos Med Assoc 2020;119:59-68.

348.	Lin Y, Wang X, Li XB, Wu BQ, Zhang ZH, Guo WH, et al. Acupuncture for persistent atrial fibrillation after catheter ablation: study protocol for a pilot randomized controlled trial. Trials 2021;22:35.

349.	Lin ZQ, Chen XJ, Dai XF, Chen LW, Lin F. Impact of frailty status on clinical and functional outcomes after concomitant valve replacement and bipolar radiofrequency ablation in patients aged 65 years and older. J Cardiothorac Surg 2022;17:295.

350.	Liu SY, Hsu YL, Tu YC, Lin CH, Wang SC, Lee YW, et al. Functional outcome prediction of ischemic stroke patients with atrial fibrillation accepting post-acute care training. Front Neurol 2022;13:954212.

351.	Liu T, Tang Z, Cai C, Wu N, Jia J, Yang G, et al. Cost-effectiveness analysis of digital therapeutics for home-based cardiac rehabilitation for patients with atrial fibrillation after catheter ablation. Digit Health 2023;9:20552076231211548.

352.	Linde M, Jubele K, Kupics K, Nikitina A, Erglis A. Quality of Life in Patients with Atrial Fibrillation Undergoing Pulmonary Vein Isolation: Short-Term Follow-Up Study. Medicina (Kaunas) 2024;60:1594.

353.	Lo M, Nair D, Mansour M, Calkins H, Reddy VY, Colley BJ, 3rd, et al. Contact force catheter ablation for the treatment of persistent atrial fibrillation: Results from the PERSIST-END study. J Cardiovasc Electrophysiol 2023;34:279-90.

354.	Lomper K, Slawuta A, Dudek K, Mazur G, Walfridsson U, Jankowska-Polanska B. Psychometric evaluation of the Polish version of the Arrhythmia-Specific Questionnaire in Tachycardia and Arrhythmia: a new tool for symptom and health-related quality of life assessment. Kardiol Pol 2019;77:541-52.

355.	Lonnerholm S, Blomstrom P, Nilsson L, Blomstrom-Lundqvist C. A high quality of life is maintained late after Maze III surgery for atrial fibrillation. Eur J Cardiothorac Surg 2009;36:558-62.

356.	Lönnerholm S, Blomström P, Nilsson L, Oxelbark S, Jideus L, Blomström-Lundqvist C. Effects of the maze operation on health-related quality of life in patients with atrial fibrillation. Circulation 2000;101:2607-11.

357.	Looi KL, Gajendragadkar P, Taha T, Elsik M, Scully E, Heck P, et al. Long-term outcomes (>2 years) of atrial fibrillation ablation using a multi-electrode ablation catheter in patients with paroxysmal atrial fibrillation. J Interv Card Electrophysiol 2013;36:61-9; discussion 69.

358.	Lowres N, Redfern J, Freedman SB, Orchard J, Bennett AA, Briffa T, et al. Choice of Health Options In prevention of Cardiovascular Events for people with Atrial Fibrillation (CHOICE-AF): A pilot study. Eur J Cardiovasc Nurs 2016;15:39-46.

359.	Lu S, Du X, Yang X, Jia Z, Li J, Xia S, et al. Physical activity and atrial tachyarrhythmia recurrence in atrial fibrillation patients after catheter ablation. Pacing Clin Electrophysiol 2020;43:922-9.

360.	Lundberg C, Albåge A, Carnlöf C, Kennebäck G. Long-term health-related quality of life after maze surgery for atrial fibrillation. Ann Thorac Surg 2008;86:1878-82.

361.	Lurz J, Hengelhaupt L, Unterhuber M, Stenzel L, Hilbert S, Schober AR, et al. App-Based Mental Training to Reduce Atrial Fibrillation-Related Symptoms After Pulmonary Vein Isolation: MENTAL AF Trial. J Am Heart Assoc 2024;13:e033500.

362.	MacDonald MR, Connelly DT, Hawkins NM, Steedman T, Payne J, Shaw M, et al. Radiofrequency ablation for persistent atrial fibrillation in patients with advanced heart failure and severe left ventricular systolic dysfunction: a randomised controlled trial. Heart 2011;97:740-7.

363.	Magnani JW, Ferry D, Swabe G, Martin D, Chen X, Brooks MM, et al. Design and rationale of the mobile health intervention for rural atrial fibrillation. Am Heart J 2022;252:16‐25.

364.	Magnussen C, Ojeda FM, Wild PS, Sörensen N, Rostock T, Hoffmann BA, et al. Atrial Fibrillation Manifestations Risk Factors and Sex Differences in a Population-Based Cohort (From the Gutenberg Health Study). Am J Cardiol 2018;122:76-82.

365.	Mahmoud alilou A, Ahmadi P, Akbarzadeh F. Cognitive Impairment in Patients with Atrial Fibrillation Measured by Mini Mental State Examination (MMSE). Med J Tabriz Univ Med Sci Health Serv 2013;35(3):82-7.

366.	Malm D, Fridlund B, Ekblad H, Karlström P, Hag E, Pakpour AH. Effects of brief mindfulness-based cognitive behavioural therapy on health-related quality of life and sense of coherence in atrial fibrillation patients. Eur J Cardiovasc Nurs 2018;17:589-97.

367.	Malmborg H, Lönnerholm S, Blomström P, Blomström-Lundqvist C. Ablation of atrial fibrillation with cryoballoon or duty-cycled radiofrequency pulmonary vein ablation catheter: a randomized controlled study comparing the clinical outcome and safety; the AF-COR study. Europace 2013;15:1567-73.

368.	Malmo V, Nes BM, Amundsen BH, Tjonna AE, Stoylen A, Rossvoll O, et al. Aerobic Interval Training Reduces the Burden of Atrial Fibrillation in the Short Term: A Randomized Trial. Circulation 2016;133:466-73.

369.	Mant JW, Richards SH, Hobbs FD, Fitzmaurice D, Lip GY, Murray E, et al. Protocol for Birmingham Atrial Fibrillation Treatment of the Aged study (BAFTA): a randomised controlled trial of warfarin versus aspirin for stroke prevention in the management of atrial fibrillation in an elderly primary care population. BMC Cardiovasc Disord 2003;3:9.

370.	Mantovan R, Macle L, De Martino G, Chen J, Morillo CA, Novak P, et al. Relationship of quality of life with procedural success of atrial fibrillation (AF) ablation and postablation AF burden: substudy of the STAR AF randomized trial. Can J Cardiol 2013;29:1211-7.

371.	Mar J, Álvarez-Sabín J, Oliva J, Becerra V, Casado M, Yébenes M, et al. The costs of stroke in Spain by aetiology: the CONOCES study protocol. Neurologia 2013;28:332-9.

372.	Marino FR, Lessard DM, Saczynski JS, McManus DD, Silverman-Lloyd LG, Benson CM, et al. Gait Speed and Mood, Cognition, and Quality of Life in Older Adults With Atrial Fibrillation. J Am Heart Assoc 2019;8:e013212.

373.	Mark DB, Anstrom KJ, Sheng S, Piccini JP, Baloch KN, Monahan KH, et al. Effect of Catheter Ablation vs Medical Therapy on Quality of Life Among Patients With Atrial Fibrillation: The CABANA Randomized Clinical Trial. JAMA 2019;321:1275-85.

374.	Marrouche NF, Brachmann J. Catheter ablation versus standard conventional treatment in patients with left ventricular dysfunction and atrial fibrillation (CASTLE-AF) - study design. Pacing Clin Electrophysiol 2009;32:987-94.

375.	Marrouche NF, Dagher L, Wazni O, Akoum N, Mansour M, El Hajjar AH, et al. Effect of DrOnedarone on atrial fibrosis progression and atrial fibrillation recurrence postablation: Design of the EDORA randomized clinical trial. J Cardiovasc Electrophysiol 2021;32:3203-10.

376.	Marshall HJ, Harris ZI, Griffith MJ, Gammage MD. Atrioventricular nodal ablation and implantation of mode switching dual chamber pacemakers: effective treatment for drug refractory paroxysmal atrial fibrillation. Heart 1998;79:543-7.

377.	Marshall HJ, Harris ZI, Griffith MJ, Holder RL, Gammage MD. Prospective randomized study of ablation and pacing versus medical therapy for paroxysmal atrial fibrillation: effects of pacing mode and mode-switch algorithm. Circulation 1999;99:1587-92.

378.	Marvig CL, Verhoef TI, de Boer A, Kamali F, Redekop K, Pirmohamed M, et al. Quality of life in patients with venous thromboembolism and atrial fibrillation treated with coumarin anticoagulants. Thromb Res 2015;136:69-75.

379.	Maryniak A, Walczak F, Bodalski R, Szumowski L, Derejko P, Urbanek P, et al. Atrial fibrillation onset circumstances and their relation to patients' quality of life. Kardiol Pol 2006;64:1102-8; discussion 1109.

380.	Marzona I, O'Donnell M, Teo K, Gao P, Anderson C, Bosch J, et al. Increased risk of cognitive and functional decline in patients with atrial fibrillation: results of the ONTARGET and TRANSCEND studies. CMAJ 2012;184:e329‐36.

381.	Masterson Creber RM, Reading Turchioe M, Biviano A, Caceres B, Garan H, Goldenthal I, et al. Cardiac symptom burden and arrhythmia recurrence drives digital health use: results from the iHEART randomized controlled trial. Eur J Cardiovasc Nurs 2022;21:107‐15.

382.	Mavaddat N, Roalfe A, Fletcher K, Lip GY, Hobbs FD, Fitzmaurice D, et al. Warfarin versus aspirin for prevention of cognitive decline in atrial fibrillation: randomized controlled trial (Birmingham Atrial Fibrillation Treatment of the Aged Study). Stroke 2014;45:1381-6.

383.	McCabe PJ, Barnason SA. Illness perceptions, coping strategies, and symptoms contribute to psychological distress in patients with recurrent symptomatic atrial fibrillation. J Cardiovasc Nurs 2012;27:431-44.

384.	McManus DD, Kiefe C, Lessard D, Waring ME, Parish D, Awad HH, et al. Geriatric Conditions and Prescription of Vitamin K Antagonists vs. Direct Oral Anticoagulants Among Older Patients With Atrial Fibrillation: SAGE-AF. Front Cardiovasc Med 2019;6:155.

385.	Medi C, Evered L, Silbert B, Teh A, Halloran K, Morton J, et al. Subtle post-procedural cognitive dysfunction after atrial fibrillation ablation. J Am Coll Cardiol 2013;62:531-9.

386.	Medin J, Arbuckle R, Abetz L, Halling K, Kulich K, Edvardsson N, et al. Development and validation of the AFSymp TM: an atrial fibrillation-specific measure of patient-reported symptoms. Patient 2014;7:319-27.

387.	Mehawej J, Saczynski J, Abu HO, Gagnier M, Bamgbade BA, Lessard D, et al. Factors Associated With Patient Engagement in Shared Decision-Making for Stroke Prevention Among Older Adults with Atrial Fibrillation. Can Geriatr J 2021;24:174-83.

388.	Mehra R, Ziegler P, Koehler J. Diagnostic information in implantable devices that pertain to endpoints in atrial fibrillation studies. Heart Rhythm 2004;1:B64-9.

389.	Mills M, Johnson E, Zafar H, Horwood A, Lax N, Charlesworth S, et al. An exercise-based cardiac rehabilitation programme for AF patients in the NHS: a feasibility study. Br J Cardiol 2020;27:20.

390.	Minjie Z, Zhijuan X, Xinxin S, Xinzhu B, Shan Q. The effects of cognitive behavioral therapy on health-related quality of life, anxiety, depression, illness perception, and in atrial fibrillation patients: a six-month longitudinal study. BMC Psychol 2023;11:431.

391.	Minjie Z, Zhijuan X, Xinxin S, Shan Q. Mediating effect of coping strategy and psychological status between illness perception and quality of life among patients with atrial fibrillation: a cross-sectional study. BMC Cardiovasc Disord 2024;24:504.

392.	Mitchell AR, Spurrell PA, Boodhoo L, Sulke N. Psychosocial aspects of patient-activated atrial defibrillation. J Cardiovasc Electrophysiol 2003;14:812-6.

393.	Miura K, Ikemura N, Kimura T, Katsumata Y, Ueda I, Tanimoto K, et al. Treatment strategies and subsequent changes in the patient-reported quality-of-life among elderly patients with atrial fibrillation. Am Heart J 2020;222:83-92.

394.	Miyama H, Ikemura N, Kimura T, Katsumata Y, Fujisawa T, Ueda I, et al. Implications of QRS Prolongation in Patients With Atrial Fibrillation (from a Multicenter Outpatient Registry). Am J Cardiol 2022;178:43-51.

395.	Miyama H, Ikemura N, Kimura T, Katsumata Y, Yamashita S, Yamaoka K, et al. Predictors and incidence of health status deterioration in patients with early atrial fibrillation. Heart Rhythm 2024;21:1469-76.

396.	Miyamoto K, Kanaoka K, Yoh M, Takahashi H, Kishihara J, Ishikura M, et al. Cryoballoon Ablation versus Radiofrequency Ablation in Patients with Persistent Atrial Fibrillation (CRRF-PeAF): Protocol for a Prospective, Multicenter, Randomized, Controlled Study. Int J Cardiol Heart Vasc 2022;41:101074.

397.	Miyazaki S, Kuwahara T, Takahashi A, Kobori A, Takahashi Y, Nozato T, et al. Effect of left atrial ablation on the quality of life in patients with atrial fibrillation. Circ J 2008;72:582-7.

398.	Mizrahi EH, Fleissig Y, Arad M, Adunsky A. Short-term functional outcome of ischemic stroke in the elderly: a comparative study of atrial fibrillation and non-atrial fibrillation patients. Arch Gerontol Geriatr 2014;58:121-4.

399.	Mohanty S, Mohanty P, Di Biase L, Bai R, Dixon A, Burkhardt D, et al. Influence of body mass index on quality of life in atrial fibrillation patients undergoing catheter ablation. Heart Rhythm 2011;8:1847-52.

400.	Mohanty S, Mohanty P, Di Biase L, Bai R, Pump A, Santangeli P, et al. Impact of metabolic syndrome on procedural outcomes in patients with atrial fibrillation undergoing catheter ablation. J Am Coll Cardiol 2012;59:1295-301.

401.	Mohanty S, Mohanty P, Di Biase L, Bai R, Santangeli P, Casella M, et al. Results from a single-blind, randomized study comparing the impact of different ablation approaches on long-term procedure outcome in coexistent atrial fibrillation and flutter (APPROVAL). Circulation 2013;127:1853-60.

402.	Mohanty S, Mohanty P, Natale V, Trivedi C, Gianni C, Burkhardt JD, et al. Impact of weight loss on ablation outcome in obese patients with longstanding persistent atrial fibrillation. J Cardiovasc Electrophysiol 2018;29:246-53.

403.	Mohanty S, Mohanty P, Rutledge JN, Di Biase L, Yan RX, Trivedi C, et al. Effect of catheter ablation and periprocedural anticoagulation regimen on the clinical course of migraine in atrial fibrillation patients with or without pre-existent migraine: results from a prospective study. Circ Arrhythm Electrophysiol 2015;8:279-87.

404.	Mohanty S, Mohanty P, Trivedi C, Assadourian J, Mayedo AQ, MacDonald B, et al. Impact of Oral Anticoagulation Therapy Versus Left Atrial Appendage Occlusion on Cognitive Function and Quality of Life in Patients With Atrial Fibrillation. J Am Heart Assoc 2021;10:e019664.

405.	Mohanty S, Santangeli P, Mohanty P, Biase LD, Holcomb S, Trivedi C, et al. Catheter Ablation of Asymptomatic Longstanding Persistent Atrial Fibrillation: Impact on Quality of Life, Exercise Performance, Arrhythmia Perception, and Arrhythmia-Free Survival. J Cardiovasc Electrophysiol 2014;25:1057-64.

406.	Monahan KH, Bunch TJ, Mark DB, Poole JE, Bahnson TD, Al-Khalidi HR, et al. Influence of atrial fibrillation type on outcomes of ablation vs. drug therapy: results from CABANA. Europace 2022;24:1430-40.

407.	Montbleau KE, King D, Henault L, Magnani JW. Health literacy, health-related quality of life, and atrial fbrillation. Cogent Med 2017;4:1412121.

408.	Monz BU, Connolly SJ, Korhonen M, Noack H, Pooley J. Assessing the impact of dabigatran and warfarin on health-related quality of life: results from an RE-LY sub-study. Int J Cardiol 2013;168:2540-7.

409.	Moreira RS, Bassolli L, Coutinho E, Ferrer P, Braganca EO, Carvalho AC, et al. Reproducibility and Reliability of the Quality of Life Questionnaire in Patients With Atrial Fibrillation. Arq Bras Cardiol 2016;106:171-81.

410.	Morillo CA, Verma A, Connolly SJ, Kuck KH, Nair GM, Champagne J, et al. Radiofrequency ablation vs antiarrhythmic drugs as first-line treatment of paroxysmal atrial fibrillation (RAAFT-2): a randomized trial. JAMA 2014;311:692-700.

411.	Mortsell D, Jansson V, Malmborg H, Lonnerholm S, Blomstrom-Lundqvist C. Clinical outcome of the 2nd generation cryoballoon for pulmonary vein isolation in patients with persistent atrial fibrillation - A sub-study of the randomized trial evaluating single versus dual cryoballoon applications. Int J Cardiol 2019;278:120-5.

412.	Mörtsell D, Malmborg H, Lönnerholm S, Jansson V, Blomström-Lundqvist C. Acute and long-term efficacy and safety with a single cryoballoon application as compared with the standard dual application strategy: a prospective randomized study using the second-generation cryoballoon for pulmonary vein isolation in patients with symptomatic atrial fibrillation. Europace 2018;20:1598-605.

413.	Mszar R, Friedman DJ, Ong E, Du C, Wang Y, Zeitler EP, et al. Sex-based differences in atrial fibrillation ablation adverse events. Heart 2023;109:595-605.

414.	Mujović NM, Marinković MM, Nedeljković I, Marković N, Banović M, Vučićević V, et al. Improvement of Maximal Exercise Performance After Catheter-Ablation of Atrial Fibrillation and Its Prognostic Significance for Long-Term Rhythm Outcome. J Am Heart Assoc 2021;10:e017445.

415.	Mulder BA, Van Veldhuisen DJ, Crijns HJ, Tijssen JG, Hillege HL, Alings M, et al. Lenient vs. strict rate control in patients with atrial fibrillation and heart failure: a post-hoc analysis of the RACE II study. Eur J Heart Fail 2013;15:1311-8.

416.	Murray RD, Shah A, Jasper SE, Goodman A, Deitcher SR, Katz WE, et al. Transesophageal echocardiography guided enoxaparin antithrombotic strategy for cardioversion of atrial fibrillation: the ACUTE II pilot study. Am Heart J 2000;139:e1-7.

417.	Nagata K, Inoue H, Yamashita T, Akao M, Atarashi H, Ikeda T, et al. Impact of cognitive impairment on clinical outcomes in elderly patients with atrial fibrillation: ANAFIE Registry. BMJ Neurol Open 2023;5:e000370.

418.	Nair GM, Birnie DH, Wells GA, Nery PB, Redpath CJ, Sarrazin JF, et al. Augmented wide area circumferential catheter ablation for reduction of atrial fibrillation recurrence (AWARE) trial: Design and rationale. Am Heart J 2022;248:1-12.

419.	Nakamaru R, Ikemura N, Spertus JA, Kimura T, Katsumata Y, Fujisawa T, et al. Rate versus rhythm control in patients with newly diagnosed atrial fibrillation: Effects of the treatment timing on health status outcomes. Am Heart J 2022;254:156-65.

420.	Nakase T, Tatewaki Y, Thyreau B, Odagiri H, Tomita N, Yamamoto S, et al. Impact of atrial fibrillation on the cognitive decline in Alzheimer's disease. Alzheimers Res Ther 2023;15:15.

421.	Nasso G, Bonifazi R, Del Prete A, Del Prete G, Lopriore V, Bartolomucci F, et al. Long-term results of ablation for isolated atrial fibrillation through a right minithoracotomy: toward a rational revision of treatment protocols. J Thorac Cardiovasc Surg 2011;142:e41-6.

422.	Natale A, Reddy VY, Monir G, Wilber DJ, Lindsay BD, McElderry HT, et al. Paroxysmal AF catheter ablation with a contact force sensing catheter: results of the prospective, multicenter SMART-AF trial. J Am Coll Cardiol 2014;64:647-56.

423.	Natale A, Calkins H, Osorio J, Pollak SJ, Melby D, Marchlinski FE, et al. Positive Clinical Benefit on Patient Care, Quality of Life, and Symptoms After Contact Force-Guided Radiofrequency Ablation in Persistent Atrial Fibrillation: Analyses From the PRECEPT Prospective Multicenter Study. Circ Arrhythm Electrophysiol 2021;14:e008867.

424.	Natale A, Zimerman L, Tomassoni G, Newby K, Leonelli F, Fanelli R, et al. AV node ablation and pacemaker implantation after withdrawal of effective rate-control medications for chronic atrial fibrillation: effect on quality of life and exercise performance. Pacing Clin Electrophysiol 1999;22:1634-9.

425.	Nazlı C, Kahya Eren N, Yakar Tülüce S, Koçağra Yağız İ G, Kılıçaslan B, Kocabaş U. Impaired quality of life in patients with intermittent atrial fibrillation. Anatol J Cardiol 2016;16:250-5.

426.	Nery PB, Wells GA, Verma A, Joza J, Nair GM, Veenhuyzen G, et al. Characterization of arrhythmia substrate to ablate persistent atrial fibrillation (COAST-AF): Randomized controlled trial design and rationale. Am Heart J 2022;254:133-40.

427.	Newman DM, Dorian P, Paquette M, Sulke N, Gold MR, Schwartzman DS, et al. Effect of an implantable cardioverter defibrillator with atrial detection and shock therapies on patient-perceived, health-related quality of life. Am Heart J 2003;145:841-6.

428.	Ng DL, Gan GG, Chai CS, Chee KH, Tan KL, Tan SB, et al. Comparing quality of life and treatment satisfaction between patients on warfarin and direct oral anticoagulants: a cross-sectional study. Patient Prefer Adherence 2019;13:1363-73.

429.	Ng DL, Malik N, Chai CS, Goh GM, Tan SB, Bee PC, et al. Time in therapeutic range, quality of life and treatment satisfaction of patients on long-term warfarin for non-valvular atrial fibrillation: a cross-sectional study. Health Qual Life Outcomes 2020;18:347.

430.	Nielsen JC, Johannessen A, Raatikainen P, Hindricks G, Walfridsson H, Pehrson SM, et al. Long-term efficacy of catheter ablation as first-line therapy for paroxysmal atrial fibrillation: 5-year outcome in a randomised clinical trial. Heart 2017;103:368-76.

431.	Nørgaard MW, Pedersen PU, Bjerrum M. Understanding how patients use visualization during ablation of atrial fibrillation in reducing their experience of pain, anxiety, consumption of pain medication and procedure length: Integrating quantitative and qualitative results. Appl Nurs Res 2018;39:229-40.

432.	Noubiap JJ, Pathak RK, Thomas G, Elliott AD, Sanders P, Middeldorp ME. Sex Differences in Outcomes of an Intensive Risk Factor Modification Program in Patients With Atrial Fibrillation. Circ Arrhythm Electrophysiol 2024;17:e012534.

433.	Nourmohammadi Z, Khalifehzadeh-Esfahani A, Eftekhari M, Sanei H. The effect of aerobic physical rehabilitation on the quality of life in patients with chronic atrial fibrillation; A randomized controlled clinical trial study. ARYA Atheroscler 2019;15:1-8.

434.	O'Connell JE, Gray CS, French JM, Robertson IH. Atrial fibrillation and cognitive function: case-control study. J Neurol Neurosurg Psychiatry 1998;65:386-9.

435.	O'Neill L, Almorad A, El Haddad M, Wielandts JY, Gillis K, Hilfiker G, et al. Impact of Catheter Ablation on Arrhythmia Burden in Patients With Shock-Resistant Persistent Atrial Fibrillation. JACC Clin Electrophysiol 2023;9:2071-81.

436.	Okumura Y, Nagashima K, Watanabe R, Yokoyama K, Kato T, Fukaya H, et al. Registry for Evaluating Healthy Life Expectancy and Long-Term Outcomes after Catheter Ablation of Atrial Fibrillation in the Very Elderly (REHEALTH AF) study: rationale and design of a prospective, multicentre, observational, comparative study. BMJ Open 2023;13:e068894.

437.	Ogawa S, Yamashita T, Yamazaki T, Aizawa Y, Atarashi H, Inoue H, et al. Optimal treatment strategy for patients with paroxysmal atrial fibrillation: J-RHYTHM Study. Circ J 2009;73:242-8.

438.	Ong L, Cribbie R, Harris L, Dorian P, Newman D, Mangat I, et al. Psychological correlates of quality of life in atrial fibrillation. Qual Life Res 2006;15:1323-33.

439.	Ong L [a], Irvine J, Nolan R, Cribbie R, Harris L, Newman D, et al. Gender differences and quality of life in atrial fibrillation: the mediating role of depression. J Psychosom Res 2006;61:769-74.

440.	Oser M, Khan A, Kolodziej M, Gruner G, Barsky AJ, Epstein L. Mindfulness and interoceptive exposure therapy for anxiety sensitivity in atrial fibrillation: A pilot study. Behav Modif 2021;45:462-79.

441.	Osbak PS, Mourier M, Henriksen JH, Kofoed KF, Jensen GB. Effect of physical exercise training on muscle strength and body composition, and their association with functional capacity and quality of life in patients with atrial fibrillation: a randomized controlled trial. J Rehabil Med 2012;44:975-9.

442.	Osmancik P, Budera P, Talavera D, Herman D, Vesela J, Prochazkova R, et al. Improvement in the quality of life of patients with persistent or long-standing persistent atrial fibrillation after hybrid ablation. J Interv Card Electrophysiol 2020;57:435-42.

443.	Osmancik P, Havránek Š, Bulková V, Chovančík J, Roubíček T, Heřman D, et al. Catheter ablation versus antiarrhythmic drugs with risk factor modification for treatment of atrial fibrillation: a protocol of a randomised controlled trial (PRAGUE-25 trial). BMJ Open 2022;12:e056522.

444.	Ozel CB, Ozdemir HY, Dural M, Al A, Yalvac HE, Mert GO, et al. The one-minute sit-to-stand test is an alternative to the 6-minute walk test in patients with atrial fibrillation: A cross-sectional study and ROC curve analysis. Int J Cardiol 2025;419:132713.

445.	Özin B, Aytemir K, Aslan Ö, Özcan T, Kanadaşı M, Demir M, et al. Clinical practices of the management of nonvalvular atrial fibrillation and outcome of treatment: A representative prospective survey in tertiary healthcare centers across Turkey. Turk Kardiyol Dern Ars 2018;46:92-102.

446.	Packer DL, Mark DB, Robb RA, Monahan KH, Bahnson TD, Moretz K, et al. Catheter Ablation versus Antiarrhythmic Drug Therapy for Atrial Fibrillation (CABANA) Trial: Study Rationale and Design. Am Heart J 2018;199:192-9.

447.	Packer DL, Piccini JP, Monahan KH, Al-Khalidi HR, Silverstein AP, Noseworthy PA, et al. Ablation Versus Drug Therapy for Atrial Fibrillation in Heart Failure: Results From the CABANA Trial. Circulation 2021;143:1377-90.

448.	Pappone C, Rosanio S, Augello G, Gallus G, Vicedomini G, Mazzone P, et al. Mortality, morbidity, and quality of life after circumferential pulmonary vein ablation for atrial fibrillation: outcomes from a controlled nonrandomized long-term study. J Am Coll Cardiol 2003;42:185-97.

449.	Pappone C, Vicedomini G, Augello G, Manguso F, Saviano M, Baldi M, et al. Radiofrequency catheter ablation and antiarrhythmic drug therapy: a prospective, randomized, 4-year follow-up trial: the APAF study. Circ Arrhythm Electrophysiol 2011;4:808-14.

450.	Parimbelli E, Sacchi L, Rubrichi S, Mazzanti A, Quaglini S. UceWeb: a web-based collaborative tool for collecting and sharing quality of life data. Methods Inf Med 2015;54:156-63.

451.	Paquette M, Roy D, Talajic M, Newman D, Couturier A, Yang C, et al. Role of gender and personality on quality-of-life impairment in intermittent atrial fibrillation. Am J Cardiol 2000;86:764-8.

452.	Park H, Hildreth A, Thomson R, O'Connell J. Non-valvular atrial fibrillation and cognitive decline: a longitudinal cohort study. Age Ageing 2007;36:157-63.

453.	Park JW, Yu HT, Kim TH, Uhm JS, Joung B, Lee MH, et al. Atrial Fibrillation Catheter Ablation Increases the Left Atrial Pressure. Circ Arrhythm Electrophysiol 2019;12:e007073.

454.	Parkash R, Wells GA, Rouleau J, Talajic M, Essebag V, Skanes A, et al. Randomized Ablation-Based Rhythm-Control Versus Rate-Control Trial in Patients With Heart Failure and Atrial Fibrillation: Results from the RAFT-AF trial. Circulation 2022;145:1693-704.

455.	Parks AL, Jeon SY, Boscardin WJ, Steinman MA, Smith AK, Fang MC, et al. Long-term individual and population functional outcomes in older adults with atrial fibrillation. J Am Geriatr Soc 2021;69:1570-8.

456.	Patel N, Chung EH, Mounsey JP, Schwartz JD, Pursell I, Gehi AK. Effectiveness of atrial fibrillation monitor characteristics to predict severity of symptoms of atrial fibrillation. Am J Cardiol 2014;113:1674-8.

457.	Pavelková Z, Bulava A. Nursing and quality of life in patients with atrial fibrillation before and after radiofrequency ablation. Neuro Endocrinol Lett 2014;35 Suppl 1:49-53.

458.	Pavlovic N, Chierchia GB, Velagic V, Hermida JS, Healey S, Arena G, et al. Initial rhythm control with cryoballoon ablation vs drug therapy: Impact on quality of life and symptoms. Am Heart J 2021;242:103-14.

459.	Peinado R, Arribas F, Ormaetxe JM, Badía X. Variation in quality of life with type of atrial fibrillation. Rev Esp Cardiol 2010;63:1402-9.

460.	Peng QI, Yi-Zhen Y, Liang SHI, Yan-Jiang W, Ying T, Ke-Xin Y, et al. His-Purkinje conduction system pacing combined with atrioventricular node ablation improves quality of life in older patients with persistent atrial fibrillation refractory to multiple ablation procedures. J Geriatr Cardiol 2023;20:130-8.

461.	Perret-Guillaume C, Briancon S, Wahl D, Guillemin F, Empereur F. Quality of Life in elderly inpatients with atrial fibrillation as compared with controlled subjects. J Nutr Health Aging 2010;14:161-6.

462.	Pezawas T, Ristl R, Schukro C, Schmidinger H. Health-related quality of life changes in patients undergoing repeated catheter ablation for atrial fibrillation. Clin Res Cardiol 2016;105:1-9.

463.	Piccini JP, Fraulo ES, Ansell JE, Fonarow GC, Gersh BJ, Go AS, et al. Outcomes registry for better informed treatment of atrial fibrillation: rationale and design of ORBIT-AF. Am Heart J 2011;162:606-12.e1.

464.	Piccini JP, Simon DN, Steinberg BA, Thomas L, Allen LA, Fonarow GC, et al. Differences in Clinical and Functional Outcomes of Atrial Fibrillation in Women and Men: Two-Year Results From the ORBIT-AF Registry. JAMA Cardiol 2016;1:282-91.

465.	Piccini JP, Todd DM, Massaro T, Lougee A, Haeusler KG, Blank B, et al. Changes in quality of life, cognition and functional status following catheter ablation of atrial fibrillation. Heart 2020;106:1919-26.

466.	Pierre-Louis IC, Saczynski JS, Lopez-Pintado S, Waring ME, Abu HO, Goldberg RJ, et al. Characteristics associated with poor atrial fibrillation-related quality of life in adults with atrial fibrillation. J Cardiovasc Med 2023;24:422-9.

467.	Pilotto A, Gallina P, Copetti M, Pilotto A, Marcato F, Mello AM, et al. Warfarin Treatment and All-Cause Mortality in Community-Dwelling Older Adults with Atrial Fibrillation: A Retrospective Observational Study. J Am Geriatr Soc 2016;64:1416-24.

468.	Pilotto A, Veronese N, Polidori MC, Strandberg T, Topinkova E, Cruz-Jentoft AJ, et al. The role of prognostic stratification on prescription of anticoagulants in older patients with atrial fibrillation: a multicenter, observational, prospective European study (EUROSAF). Ann Med 2022;54:2411-9.

469.	Plisiene J, Blumberg A, Haager G, Knackstedt C, Latsch J, Norra C, et al. Moderate physical exercise: a simplified approach for ventricular rate control in older patients with atrial fibrillation. Clin Res Cardiol 2008;97:820-6.

470.	Poggesi A, Barbato C, Galmozzi F, Camilleri E, Cesari F, Chiti S, et al. Role of Biological Markers for Cerebral Bleeding Risk STRATification in Patients with Atrial Fibrillation on Oral Anticoagulants for Primary or Secondary Prevention of Ischemic Stroke (Strat-AF Study): Study Design and Methodology. Medicina (Kaunas) 2019;55:626.

471.	Pokorney SD, Kim S, Thomas L, Fonarow GC, Kowey PR, Gersh BJ, et al. Cardioversion and subsequent quality of life and natural history of atrial fibrillation. Am Heart J 2017;185:59-66.

472.	Pokorney SD, Nemeth H, Chiswell K, Albert C, Allyn N, Blanco R, et al. Design and rationale of a pragmatic randomized clinical trial of early dronedarone versus usual care to change and improve outcomes in persons with first-detected atrial fibrillation - the CHANGE AFIB study. Am Heart J 2025;279:66-75.

473.	Pontoppidan J, Nielsen JC, Poulsen SH, Hansen PS. Symptomatic and asymptomatic atrial fibrillation after pulmonary vein ablation and the impact on quality of life. Pacing Clin Electrophysiol 2009;32:717-26.

474.	Potpara TS, Mihajlovic M, Zec N, Marinkovic M, Kovacevic V, Simic J, et al. Self-reported treatment burden in patients with atrial fibrillation: quantification, major determinants, and implications for integrated holistic management of the arrhythmia. Europace 2020;22:1788-97.

475.	Potter T, Herendael HV, Balasubramaniam R, Wright M, Laţcu DG, Agarwal SC, et al. Safety and Long-Term Success of Persistent Atrial Fibrillation Ablation Using THERMOCOOL SMARTTOUCH® Catheter: Real-World Experience. J Atr Fibrillation 2019;12:2126.

476.	Prabhu S, Taylor AJ, Costello BT, Kaye DM, McLellan AJA, Voskoboinik A, et al. Catheter Ablation Versus Medical Rate Control in Atrial Fibrillation and Systolic Dysfunction: the CAMERA-MRI Study. J Am Coll Cardiol 2017;70:1949‐61.

477.	Praus T, Li J, Barbarash S, Proenza M, Bondmass MD. Improving care for patients with atrial fibrillation through the use of a personal electrocardiogram. J Am Assoc Nurse Pract 2021;33:1307-13.

478.	Proietti M, Vitolo M, Harrison SL, Lane DA, Fauchier L, Marin F, et al. Real-world applicability and impact of early rhythm control for European patients with atrial fibrillation: a report from the ESC-EHRA EORP-AF Long-Term General Registry. Clin Res Cardiol 2022;111:70-84.

479.	Punjani S, Wu WC, Cohen S, Sharma SC, Choudhary G, Punjani S, et al. Echocardiographic indices of diastolic function relate to functional capacity and quality of life in ambulatory men with atrial fibrillation. J Am Soc Echocardiogr 2011;24:533-40.e3.

480.	Purerfellner H, Martinek M, Aichinger J, Nesser HJ, Kempen K, Janssen JP. Quality of life restored to normal in patients with atrial fibrillation after pulmonary vein ostial isolation. Am Heart J 2004;148:318-25.

481.	Qing L, Yinong C, Luyao Y, Longyang Z, Zhe W, Siqi J, et al. Sex Differences in Quality of Life and their Explanatory Variables in Patients with Non-Valvular Atrial Fibrillation. CVIA 2023;8:1-10.

482.	Raatikainen MJ, Hakalahti A, Uusimaa P, Nielsen JC, Johannessen A, Hindricks G, et al. Radiofrequency catheter ablation maintains its efficacy better than antiarrhythmic medication in patients with paroxysmal atrial fibrillation: On-treatment analysis of the randomized controlled MANTRA-PAF trial. Int J Cardiol 2015;198:108-14.

483.	Raine D, Langley P, Shepherd E, Lord S, Murray S, Murray A, et al. Effect of catheter ablation on quality of life in patients with atrial fibrillation and its correlation with arrhythmia outcome. Open Heart 2015;2:e000302.

484.	Raileanu G, Jawid N, Bohte E, Hof IE, Khan M, de Ruiter GS, et al. Do depressive and anxiety symptoms influence the quality of life of patients with atrial fibrillation after cryoballoon ablation: a comparison study. J Interv Card Electrophysiol 2024;67:797-805.

485.	Ramos Tuarez FJ, Pino JE, Alrifai A, Kabach M, Donath E, Saona J, et al. Long-term quality of life in octogenarians and nonagenarians with nonvalvular atrial fibrillation following WATCHMAN™ device implantation. Catheter Cardiovasc Interv 2019;93:1138-45.

486.	Randolph TC, Simon DN, Thomas L, Allen LA, Fonarow GC, Gersh BJ, et al. Patient factors associated with quality of life in atrial fibrillation. Am Heart J 2016;182:135-43.

487.	Reddy VY, Mansour M, Calkins H, d'Avila A, Chinitz L, Woods C, et al. Pulsed Field vs Conventional Thermal Ablation for Paroxysmal Atrial Fibrillation: recurrent Atrial Arrhythmia Burden. J Am Coll Cardiol 2024;84:61‐74.

488.	Reed JL, Clarke AE, Faraz AM, Birnie DH, Tulloch HE, Reid RD, et al. The Impact of Cardiac Rehabilitation on Mental and Physical Health in Patients With Atrial Fibrillation: A Matched Case-Control Study. Can J Cardiol 2018;34:1512-21.

489.	Reynolds MR, Lavelle T, Essebag V, Cohen DJ, Zimetbaum P. Influence of age, sex, and atrial fibrillation recurrence on quality of life outcomes in a population of patients with new-onset atrial fibrillation: the Fibrillation Registry Assessing Costs, Therapies, Adverse events and Lifestyle (FRACTAL) study. Am Heart J 2006;152:1097-103.

490.	Reynolds MR, Morais E, Zimetbaum P. Impact of hospitalization on health-related quality of life in atrial fibrillation patients in Canada and the United States: results from an observational registry. Am Heart J 2010;160:752-8.

491.	Reynolds MR [a], Walczak J, White SA, Cohen DJ, Wilber DJ. Improvements in symptoms and quality of life in patients with paroxysmal atrial fibrillation treated with radiofrequency catheter ablation versus antiarrhythmic drugs. Circ Cardiovasc Qual Outcomes 2010;3:615-23.

492.	Ricci R, Quesada A, Pignalberi C, Roda J, Disertori M, Capucci A, et al. Dual defibrillator improves quality of life and decreases hospitalizations in patients with drug refractory atrial fibrillation. J Interv Card Electrophysiol 2004;10:85-92.

493.	Richard G, O'Halloran AM, Doody P, Harbison J, Kenny RA, Romero-Ortuno R. Atrial fibrillation and acceleration of frailty: findings from the Irish Longitudinal Study on Ageing. Age Ageing 2022;51:afab273.

494.	Rienstra M, Hagens VE, Van Veldhuisen DJ, Bosker HA, Tijssen JG, Kamp O, et al. Clinical characteristics of persistent lone atrial fibrillation in the RACE study. Am J Cardiol 2004;94:1486-90.

495.	Rienstra M, Lyass A, Murabito JM, Magnani JW, Lubitz SA, Massaro JM, et al. Reciprocal relations between physical disability, subjective health, and atrial fibrillation: the Framingham Heart Study. Am Heart J 2013;166:171-8.

496.	Rienstra M, Van Veldhuisen DJ, Hagens VE, Ranchor AV, Veeger NJ, Crijns HJ, et al. Gender-related differences in rhythm control treatment in persistent atrial fibrillation: data of the Rate Control Versus Electrical Cardioversion (RACE) study. J Am Coll Cardiol 2005;46:1298-306.

497.	Rienstra M, Vermond RA, Crijns HJ, Tijssen JG, Van Gelder IC. Asymptomatic persistent atrial fibrillation and outcome: results of the RACE study. Heart Rhythm 2014;11:939-45.

498.	Rillig A, Bellmann B, Skurk C, Leistner DM, Haeusler KG, Lin T, et al. Left atrial appendage angiography is associated with the incidence and number of magnetic resonance imaging-detected brain lesions after percutaneous catheter-based left atrial appendage closure. Heart Rhythm 2018;15:3-8.

499.	Rinaldi S, Fontani V. The Efficacy and Safety of Radio Electric Asymmetric Conveyer (REAC) External Radio Electric Reprogramming for Atrial Fibrillation (EX-RER AF) Treatment: Results From a Post-market Clinical Follow-Up. Cureus 2024;16:e76057.

500.	Risom SS, Cromhout PF, Overgaard D, Svendsen JH, Berg SK. Effect of Rehabilitation on Sleep Quality After Ablation for Atrial Fibrillation: Data From a Randomized Trial. J Cardiovasc Nurs 2018;33:261-8.

501.	Risom SS, Lind J, Dickson VV, Berg SK. Exploring the mechanism of effectiveness of a psychoeducational intervention in a rehabilitation program (CopenHeartRFA) for patients treated with ablation for atrial fibrillation: A mixed methods study. J Cardiovasc Nurs 2019;34:336-43.

502.	Risom SS, Thygesen LC, Rasmussen TB, Borregaard B, Nørgaard MW, Mols R, et al. Association Between Risk Factors and Readmission for Patients With Atrial Fibrillation Treated With Catheter Ablation: Results From the Nationwide DenHeart Study. J Cardiovasc Nurs 2023;38:e31-9.

503.	Risom SS, Zwisler AD, Rasmussen TB, Sibilitz KL, Madsen TL, Svendsen JH, et al. Cardiac rehabilitation versus usual care for patients treated with catheter ablation for atrial fibrillation: results of the randomized CopenHeartRFA trial. Am Heart J 2016;181:120‐9.

504.	Risom SS, Zwisler AD, Sibilitz KL, Rasmussen TB, Taylor RS, Thygesen LC, et al. Cardiac Rehabilitation for Patients Treated for Atrial Fibrillation With Ablation Has Long-Term Effects: 12-and 24-Month Follow-up Results From the Randomized CopenHeart(RFA) Trial. Arch Phys Med Rehabil 2020;101:1877-86.

505.	Risom SS, Zwisler AD, Thygesen LC, Svendsen JH, Berg SK. High Readmission Rates and Mental Distress 1 yr After Ablation for Atrial Fibrillation or Atrial Flutter: A NATIONWIDE SURVEY. J Cardiopulm Rehabil Prev 2019;39:33-8.

506.	Ritchie LA, Penson PE, Akpan A, Lip GYH, Lane DA. Pharmacist-led intervention for older people with atrial fibrillation in long-term care (PIVOTALL study): a randomised pilot and feasibility study. BMC Geriatr 2024;24:64.

507.	Roalfe AK, Bryant TL, Davies MH, Hackett TG, Saba S, Fletcher K, et al. A cross-sectional study of quality of life in an elderly population (75 years and over) with atrial fibrillation: secondary analysis of data from the Birmingham Atrial Fibrillation Treatment of the Aged study. Europace 2012;14:1420-7.

508.	Rosenstrøm S, Risom SS, Kallemose T, Dixen U, Hove JD, Brødsgaard A. Clinical outcomes of a short-term family-focused intervention for patients with atrial fibrillation-A randomised clinical trial. PLoS One 2023;18:e0282639.

509.	Rosman L, Armbruster T, Kyazimzade S, Tugaoen Z, Mazzella AJ, Deyo Z, et al. Effect of a virtual self-management intervention for atrial fibrillation during the outbreak of COVID-19. Pacing Clin Electrophysiol 2021;44:451-61.

510.	Rowin EJ, Wadid M, Maron MS, White M, Hook BG, Harnish P, et al. Assessing the impact of atrial fibrillation on symptoms and quality of life in hypertrophic cardiomyopathy. J Cardiovasc Electrophysiol 2024;35:654-63.

511.	Rush KL, Seaton CL, O'Connor BP, Andrade JG, Loewen P, Corman K, et al. Managing With Atrial Fibrillation: An Exploratory Model-Based Cluster Analysis of Clinical and Personal Patient Characteristics. CJC Open 2023;5:833-45.

512.	Sabatini T, Frisoni GB, Barbisoni P, Bellelli G, Rozzini R, Trabucchi M. Atrial fibrillation and cognitive disorders in older people. J Am Geriatr Soc 2000;48:387-90.

513.	Salvadori E, Barucci E, Barbato C, Formelli B, Cesari F, Chiti S, et al. Cognitive phenotypes and factors associated with cognitive decline in a cohort of older patients with atrial fibrillation: The Strat-AF study. Eur J Neurol 2023;30:849-60.

514.	Samuel M, Khairy P, Champagne J, Deyell MW, Macle L, Leong-Sit P, et al. Association of Atrial Fibrillation Burden With Health-Related Quality of Life After Atrial Fibrillation Ablation: Substudy of the Cryoballoon vs Contact-Force Atrial Fibrillation Ablation (CIRCA-DOSE) Randomized Clinical Trial. JAMA Cardiol 2021;6:1324-8.

515.	Sandhu RK, Smigorowsky M, Lockwood E, Savu A, Kaul P, McAlister FA. Impact of Electrical Cardioversion on Quality of Life for the Treatment of Atrial Fibrillation. Can J Cardiol 2017;33:450-5.

516.	Sang CH, Chen K, Pang XF, Dong JZ, Du X, Ma H, et al. Depression, anxiety, and quality of life after catheter ablation in patients with paroxysmal atrial fibrillation. Clin Cardiol 2013;36:40-5.

517.	Sanghai S, Wong C, Wang Z, Clive P, Tran W, Waring M, et al. Rates of Potentially Inappropriate Dosing of Direct-Acting Oral Anticoagulants and Associations With Geriatric Conditions Among Older Patients With Atrial Fibrillation: The SAGE-AF Study. J Am Heart Assoc 2020;9:e014108.

518.	Sarnholm J, Skuladottir H, Ruck C, Axelsson E, Bonnert M, Bragesjo M, et al. Cognitive Behavioral Therapy Improves Quality of Life in Patients With Symptomatic Paroxysmal Atrial Fibrillation. J Am Coll Cardiol 2023;82:46‐56.

519.	Särnholm J, Skúladóttir H, Rück C, Pedersen SS, Braunschweig F, Ljótsson B. Exposure-Based Therapy for Symptom Preoccupation in Atrial Fibrillation: An Uncontrolled Pilot Study. Behav Ther 2017;48:808-19.

520.	Scheuermeyer FX, Andolfatto G, Christenson J, Villa-Roel C, Rowe B. A Multicenter Randomized Trial to Evaluate a Chemical-first or Electrical-first Cardioversion Strategy for Patients With Uncomplicated Acute Atrial Fibrillation. Acad Emerg Med 2019;26:969-81.

521.	Schilling R, Dhillon GS, Tondo C, Riva S, Grimaldi M, Quadrini F, et al. Safety, effectiveness, and quality of life following pulmonary vein isolation with a multi-electrode radiofrequency balloon catheter in paroxysmal atrial fibrillation: 1-year outcomes from SHINE. Europace 2021;23:851-60.

522.	Schmidt B, Széplaki G, Merkely B, Kautzner J, van Driel V, Bourier F, et al. Silent cerebral lesions and cognitive function after pulmonary vein isolation with an irrigated gold-tip catheter: REDUCE-TE Pilot study. J Cardiovasc Electrophysiol 2019;30:877-85.

523.	Schnabel RB, Michal M, Wilde S, Wiltink J, Wild PS, Sinning CR, et al. Depression in atrial fibrillation in the general population. PLoS One 2013;8:e79109.

524.	Schnabel RB, Pecen L, Rzayeva N, Lucerna M, Purmah Y, Ojeda FM, et al. Symptom Burden of Atrial Fibrillation and Its Relation to Interventions and Outcome in Europe. J Am Heart Assoc 2018;7:e007559.

525.	Schron E, Friedmann E, Thomas SA. Does health-related quality of life predict hospitalization or mortality in patients with atrial fibrillation? J Cardiovasc Electrophysiol 2014;25:23-8.

526.	Seki Y, Fujisawa T, Ikemura N, Ibe S, Tsuzuki I, Hashimoto K, et al. Catheter ablation improves outcomes and quality of life in Japanese patients with early-stage atrial fibrillation: A retrospective cohort study. Heart Rhythm 2022;19:1076-83.

527.	Segan L, Chieng D, Crowley R, William J, Sugumar H, Ling LH, et al. Sex-specific outcomes after catheter ablation for persistent AF. Heart Rhythm 2024;21:762‐70.

528.	Seligman WH, Das-Gupta Z, Jobi-Odeneye AO, Arbelo E, Banerjee A, Bollmann A, et al. Development of an international standard set of outcome measures for patients with atrial fibrillation: a report of the International Consortium for Health Outcomes Measurement (ICHOM) atrial fibrillation working group. Eur Heart J 2020;41:1132-40.

529.	Seliutskii SI, Savina NM, Chapurnykh AV. [The effectiveness of radiofrequency ablation and repeated cardioversion in combination with antiarrhythmic drug therapy in maintaining stable sinus rhythm in patients with atrial fibrillation and heart failure]. Kardiologiia 2020;60:90-7.

530.	Semaan S, Dewland TA, Tison GH, Nah G, Vittinghoff E, Pletcher MJ, et al. Physical activity and atrial fibrillation: Data from wearable fitness trackers. Heart Rhythm 2020;17:842-6.

531.	Senoo K, Yukawa A, Ohkura T, Iwakoshi H, Nishimura T, Teramukai S, et al. Depression and quality of life in older adults with atrial fibrillation: A cross-sectional community-based study. Geriatr Gerontol Int 2022;22:505-10.

532.	Serber ER, Sears MF, Sotile RO, Burns JL, Schwartzman DS, Hoyt RH, et al. Sleep quality among patients treated with implantable atrial defibrillation therapy: effect of nocturnal shock delivery and psychological distress. J Cardiovasc Electrophysiol 2003;14:960-4.

533.	Serpytis R, Navickaite A, Serpytiene E, Barysiene J, Marinskis G, Jatuzis D, et al. Impact of Atrial Fibrillation on Cognitive Function, Psychological Distress, Quality of Life, and Impulsiveness. Am J Med 2018;131:703.e1-.e5.

534.	Shah SJ, Fang MC, Jeon SY, Gregorich SE, Covinsky KE. Geriatric Syndromes and Atrial Fibrillation: Prevalence and Association with Anticoagulant Use in a National Cohort of Older Americans. J Am Geriatr Soc 2021;69:349-56.

535.	Shamoto A, Chishaki A, Tsuchihashi-Makaya M, Chishaki H, Takemoto M, Mukai Y, et al. Bepridil is effective and improves QOL in multidrug-resistant paroxysmal atrial fibrillation. J Cardiovasc Med (Hagerstown) 2012;13:747-54.

536.	Shan Q, Xinxin S, Zhijuan X, Rongjing D, Minjie Z. Effects of Cognitive Behavior Therapy on Depression, Illness Perception, and Quality of Life in Atrial Fibrillation Patients. Front Psychiatry 2022;13:830363.

537.	Shantsila E, Shahid F, Sun Y, Deeks J, Calvert M, Fisher JP, et al. Spironolactone in Atrial Fibrillation With Preserved Cardiac Fraction: The IMPRESS-AF Trial. J Am Heart Assoc 2020;9:e016239.

538.	Sharples L, Everett C, Singh J, Mills C, Spyt T, Abu-Omar Y, et al. Amaze: a double-blind, multicentre randomised controlled trial to investigate the clinical effectiveness and cost-effectiveness of adding an ablation device-based maze procedure as an adjunct to routine cardiac surgery for patients with pre-existing atrial fibrillation. Health Technol Assess 2018;22:1-132.

539.	She F, Ma Y, Li Y, Li L, Xu W, Wang H, et al. Influence of heart rate control on exercise capacity and quality of life in patients with permanent atrial fibrillation. BMC Cardiovasc Disord 2019;19:308.

540.	Shelton RJ, Clark AL, Goode K, Rigby AS, Houghton T, Kaye GC, et al. A randomised, controlled study of rate versus rhythm control in patients with chronic atrial fibrillation and heart failure: (CAFE-II Study). Heart 2009;95:924-30.

541.	Shiraishi Y, Kohsaka S, Ikemura N, Kimura T, Katsumata Y, Tanimoto K, et al. Catheter ablation for patients with atrial fibrillation and heart failure with reduced and preserved ejection fraction: insights from the KiCS-AF multicentre cohort study. Europace 2023;25:83-91.

542.	Si W, Yang S, Pan L, Li C, Ma L. Comparison of modified MAZE with minimally invasive monopolar ablation and traditional bipolar radiofrequency ablation in the treatment of atrial fibrillation. J Cardiothorac Surg 2019;14:198.

543.	Simpson CS, Yee R, Lee JK, Braney M, Klein GJ, Krahn AD, et al. Safety and feasibility of a novel rate-smoothed ventricular pacing algorithm for atrial fibrillation. Am Heart J 2001;142:294-300.

544.	Singh BN, Singh SN, Reda DJ, Tang XC, Lopez B, Harris CL, et al. Amiodarone versus sotalol for atrial fibrillation. N Engl J Med 2005;352:1861-72.

545.	Singh SN, Tang XC, Singh BN, Dorian P, Reda DJ, Harris CL, et al. Quality of life and exercise performance in patients in sinus rhythm versus persistent atrial fibrillation: a Veterans Affairs Cooperative Studies Program Substudy. J Am Coll Cardiol 2006;48:721-30.

546.	Skuladottir H, Sarnholm J, Olafsdottir E, Arnardottir ES, Hoppe K, Bottai M, et al. Cognitive Behavioral Therapy for Paroxysmal Atrial Fibrillation: Heart Rate Variability, Physical Activity, and Sleep. JACC Adv 2024;3:101289.

547.	Slawuta A, Jacek P, Mazur G, Jankowska-Polanska B. Quality of Life and Frailty Syndrome in Patients with Atrial Fibrillation. Clin Interv Aging 2020;15:783-95.

548.	Smigorowsky MJ, Norris CM, McMurtry MS, Tsuyuki RT. Measuring the effect of nurse practitioner (NP)-led care on health-related quality of life in adult patients with atrial fibrillation: study protocol for a randomized controlled trial. Trials 2017;18:364.

549.	Smith DE, Xuereb CB, Pattison HM, Lip GY, Lane DA. TRial of an Educational intervention on patients' knowledge of Atrial fibrillation and anticoagulant therapy, INR control, and outcome of Treatment with warfarin (TREAT). BMC Cardiovasc Disord 2010;10:21.

550.	Son YJ, Seo EJ. Impact of Sociodemographic and Clinical Factors on Health-Related Quality of Life in Older Adults With Atrial Fibrillation. Res Gerontol Nurs 2018;11:207-15.

551.	Son YJ, Song EK. The impact of type D personality and high-sensitivity C-reactive protein on health-related quality of life in patients with atrial fibrillation. Eur J Cardiovasc Nurs 2012;11:304-12.

552.	Spertus J, Dorian P, Bubien R, Lewis S, Godejohn D, Reynolds MR, et al. Development and validation of the Atrial Fibrillation Effect on QualiTy-of-Life (AFEQT) Questionnaire in patients with atrial fibrillation. Circ Arrhythm Electrophysiol 2011;4:15-25.

553.	Springer A, Monsch AU, Dutilh G, Coslovsky M, Kievit RA, Bonati LH, et al. A factor score reflecting cognitive functioning in patients from the Swiss Atrial Fibrillation Cohort Study (Swiss-AF). PLoS One 2020;15:e0240167.

554.	Spurrell P, Mitchell A, Kamalvand K, Sulke N. Quality of life after use of the patient activated atrial defibrillator. Int J Clin Pract 2003;57:30-4.

555.	Srichawla BS, Hamel AP, Cook P, Aleyadeh R, Lessard D, Otabil EM, et al. Is catheter ablation associated with preservation of cognitive function? An analysis from the SAGE-AF observational cohort study. Front Neurol 2024;14:1302020.

556.	Srinonprasert V, Ratanasumawong K, Thongsri T, Dutsadeevettakul S, Jittham P, Wiwatworapan W, et al. Factors associated with low health-related quality of life among younger and older Thai patients with non-valvular atrial fibrillation. Qual Life Res 2019;28:2091-8.

557.	Stambler BS, Ellenbogen K, Zhang X, Porter TR, Xie F, Malik R, et al. Right ventricular outflow versus apical pacing in pacemaker patients with congestive heart failure and atrial fibrillation. J Cardiovasc Electrophysiol 2003;14:1180-6.

558.	Stanley A, Van Bragt KA, Obidigbo V, Vezi B. Cryoballoon ablation for atrial fibrillation in South Africa: One-year outcome from the Cryo Global Registry. S Afr Med J 2024;114:e1338.

559.	Stefansdottir H, Arnar DO, Aspelund T, Sigurdsson S, Jonsdottir MK, Hjaltason H, et al. Atrial Fibrillation is Associated With Reduced Brain Volume and Cognitive Function Independent of Cerebral Infarcts. Stroke 2013;44:1020-5.

560.	Steg PG, Alam S, Chiang CE, Gamra H, Goethals M, Inoue H, et al. Symptoms, functional status and quality of life in patients with controlled and uncontrolled atrial fibrillation: data from the RealiseAF cross-sectional international registry. Heart 2012;98:195-201.

561.	Steinberg BA, Dorian P, Anstrom KJ, Hess R, Mark DB, Noseworthy PA, et al. Patient-Reported Outcomes in Atrial Fibrillation Research: Results of a Clinicaltrials.gov Analysis. JACC Clin Electrophysiol 2019;5:599-605.

562.	Steinberg BA, Holmes DN, Pieper K, Allen LA, Chan PS, Ezekowitz MD, et al. Factors Associated With Large Improvements in Health-Related Quality of Life in Patients With Atrial Fibrillation: Results From ORBIT-AF. Circ Arrhythm Electrophysiol 2020;13:e007775.

563.	Steinberg BA [a], Turner J, Lyons A, Biber J, Chelu MG, Fang JC, et al. Systematic collection of patient-reported outcomes in atrial fibrillation: feasibility and initial results of the Utah mEVAL AF programme. Europace 2020;22:368-74.

564.	Stewart S, Ball J, Horowitz JD, Marwick TH, Mahadevan G, Wong C, et al. Standard versus atrial fibrillation-specific management strategy (SAFETY) to reduce recurrent admission and prolong survival: pragmatic, multicentre, randomised controlled trial. Lancet 2015;385:775-84.

565.	Stridsman M, Strömberg A, Hendriks J, Walfridsson U. Patients' Experiences of Living with Atrial Fibrillation: A Mixed Methods Study. Cardiol Res Pract 2019;2019:6590358.

566.	Su WW, Reddy VY, Bhasin K, Champagne J, Sangrigoli RM, Braegelmann KM, et al. Cryoballoon ablation of pulmonary veins for persistent atrial fibrillation: Results from the multicenter STOP Persistent AF trial. Heart Rhythm 2020;17:1841-7.

567.	Suarez C, Pose A, Montero-Perez-Barquero M, Roquer J, Gallego J, Rafols C, et al. [Validation of satisfaction questionnaire ACTS in outpatients with atrial fibrillation treated with oral anticoagulants in Spain. ALADIN Study]. Med Clin (Barc) 2016;147:192-8.

568.	Suleiman M, Koestler C, Lerman A, Lopez-Jimenez F, Herges R, Hodge D, et al. Atorvastatin for prevention of atrial fibrillation recurrence following pulmonary vein isolation: a double-blind, placebo-controlled, randomized trial. Heart Rhythm 2012;9:172-8.

569.	Sulke N, Silberbauer J, Boodhoo L, Freemantle N, Kamalvand K, O'Nunain S, et al. The use of atrial overdrive and ventricular rate stabilization pacing algorithms for the prevention and treatment of paroxysmal atrial fibrillation: the Pacemaker Atrial Fibrillation Suppression (PAFS) study. Europace 2007;9:790-7.

570.	Suman-Horduna I, Roy D, Frasure-Smith N, Talajic M, Lespérance F, Blondeau L, et al. Quality of life and functional capacity in patients with atrial fibrillation and congestive heart failure. J Am Coll Cardiol 2013;61:455‐60.

571.	Sun D, Wang Q, Kang J, Zhou J, Qian R, Wang W, et al. Correlation between Serum Platelet Count and Cognitive Function in Patients with Atrial Fibrillation: A Cross-Sectional Study. Cardiol Res Pract 2021:9039610.

572.	Sun Y, Zhu J, Ma C, Liu S, Yang Y, Hu D. Stroke Risk Status, Anticoagulation Treatment, and Quality-of-Life in Chinese Patients with Atrial Fibrillation: China Registry of Atrial Fibrillation (CRAF). Cardiovasc Ther 2019;2019:7372129.

573.	Szili-Torok T, Bountioukos M, Muskens AJ, Theuns DA, Poldermans D, Roelandt JR, et al. The presence of contractile reserve has no predictive value for the evolution of left ventricular function following atrio-ventricular node ablation in patients with permanent atrial fibrillation. Eur J Echocardiogr 2005;6:344-50.

574.	Tabaja C, Younis A, Santageli P, Farwati M, Braghieri L, Nakagawa H, et al. Impact of obesity on catheter ablation of atrial fibrillation: Patient characteristics, procedural complications, outcomes, and quality of life. J Cardiovasc Electrophysiol 2023;34:1648-57.

575.	Tabaja C, Younis A, Santangeli P, Madden R, Taigen T, Farwati M, et al. Catheter ablation of atrial fibrillation in elderly and very elderly patients: safety, outcomes, and quality of life. J Interv Card Electrophysiol 2024;67:1083-92.

576.	Tada H, Naito S, Kurosaki K, Ueda M, Ito S, Shinbo G, et al. Segmental pulmonary vein isolation for paroxysmal atrial fibrillation improves quality of life and clinical outcomes. Circ J 2003;67:861-5.

577.	Tailachidis P, Tsimtsiou Z, Galanis P, Theodorou M, Kouvelas D, Athanasakis K. The Atrial Fibrillation Effect on QualiTy-of-Life (AFEQT) questionnaire: cultural adaptation and validation of the Greek version. Hippokratia 2016;20:264-7.

578.	Taishi F, Takehiro K, Nobuhiro I, Hiroshi M, Yoshinori K, Ikuko U, et al. Effect of Tricuspid Regurgitation on the Reported Quality of Life and Subsequent Outcomes in Patients With Atrial Fibrillation. J Am Heart Assoc 2022;11:e022713.

579.	Takahashi Y, Yoshito I, Takahashi A, Harada T, Mitsuhashi T, Shirota K, et al. AV nodal ablation and pacemaker implantation improves hemodynamic function in atrial fibrillation. Pacing Clin Electrophysiol 2003;26:1212-7.

580.	Tan ES, Rienstra M, Wiesfeld AC, Schoonderwoerd BA, Hobbel HH, Van Gelder IC. Long-term outcome of the atrioventricular node ablation and pacemaker implantation for symptomatic refractory atrial fibrillation. Europace 2008;10:412-8.

581.	Tan HC, Koh KWL, Wu VX, Lim TW, Wang W. Health-related quality of life, psychological distress, and symptom burden in an Asian population of outpatients with atrial fibrillation. Heart Lung 2018;47:322-8.

582.	Tang SC, Liu YB, Lin LY, Huang HC, Ho LT, Lai LP, et al. Association between atrial fibrillation burden and cognitive function in patients with atrial fibrillation. Int J Cardiol 2023;377:73-8.

583.	Tankut SS, Huang DT, Zareba W, Aktas MK, Rosero SZ, Steinberg J, et al. Insertable cardiac monitor-guided early intervention to reduce atrial fibrillation burden following catheter ablation: Study design and clinical protocol (ICM-REDUCE-AF trial). Ann Noninvasive Electrocardiol 2023;28:e13043.

584.	Tao W, Liu J, Ye C, Kwapong WR, Wang A, Wang Z, et al. Relationships between cerebral small vessel diseases markers and cognitive performance in stroke-free patients with atrial fibrillation. Front Aging Neurosci 2023;14:1045910.

585.	Tatewaki Y, Mutoh T, Sato H, Kobayashi A, Totsune T, Thyreau B, et al. Impact of Catheter Ablation on Brain Microstructure and Blood Flow Alterations for Cognitive Improvements in Patients with Atrial Fibrillation: A Pilot Longitudinal Study. J Clin Med 2022;11:4346.

586.	Tavassoli N, Perrin A, Bérard E, Gillette S, Vellas B, Rolland Y. Factors associated with undertreatment of atrial fibrillation in geriatric outpatients with Alzheimer disease. Am J Cardiovasc Drugs 2013;13:425-33.

587.	Taylor EC, O'Neill M, Hughes LD, Bennett P, Moss-Morris R. Examining Adherence to Medication in Patients With Atrial Fibrillation: The Role of Medication Beliefs, Attitudes, and Depression. J Cardiovasc Nurs 2020;35:337-46.

588.	Taylor EC, O'Neill M, Hughes LD, Moss-Morris R. Atrial fibrillation, quality of life and distress: A cluster analysis of cognitive and behavioural responses. Qual Life Res 2022;31:1415-25.

589.	Tendolkar I, Enajat M, Zwiers MP, van Wingen G, de Leeuw FE, van Kuilenburg J, et al. One-year cholesterol lowering treatment reduces medial temporal lobe atrophy and memory decline in stroke-free elderly with atrial fibrillation: evidence from a parallel group randomized trial. Int J Geriatr Psychiatry 2012;27:49-58.

590.	Terricabras M, Mantovan R, Jiang CY, Betts TR, Chen J, Deisenhofer I, et al. Association Between Quality of Life and Procedural Outcome After Catheter Ablation for Atrial Fibrillation: a Secondary Analysis of a Randomized Clinical Trial. JAMA Netw Open 2020;3:e2025473.

591.	Thind M, Holmes DN, Badri M, Pieper KS, Singh A, Blanco RG, et al. Embolic and Other Adverse Outcomes in Symptomatic Versus Asymptomatic Patients With Atrial Fibrillation (from the ORBIT-AF Registry). Am J Cardiol 2018;122:1677‐83.

592.	Thompson TS, Barksdale DJ, Sears SF, Mounsey JP, Pursell I, Gehi AK. The effect of anxiety and depression on symptoms attributed to atrial fibrillation. Pacing Clin Electrophysiol 2014;37:439-46.

593.	Thomson RG, Eccles MP, Steen IN, Greenaway J, Stobbart L, Murtagh MJ, et al. A patient decision aid to support shared decision-making on anti-thrombotic treatment of patients with atrial fibrillation: randomised controlled trial. Qual Saf Health Care 2007;16:216-23.

594.	Thrall G, Lip GY, Carroll D, Lane D. Depression, anxiety, and quality of life in patients with atrial fibrillation. Chest 2007;132:1259-64.

595.	Tian M, Kang J, Huan X, Yin J, Zhang Z. Correlation between family function and quality of life in patients with atrial fibrillation. Zhong Nan da Xue Xue Bao Yi Xue Ban 2023;48:1234-42.

596.	Tischer TS, Nitschke D, Krause I, Kundt G, Öner A, D'Ancona G, et al. Prevalence and Progression of Cognitive Impairment in Atrial Fibrillation Patients after Treatment with Catheter Ablation or Drug Therapy. Cardiol Res Pract 2019;2019:7216598.

597.	Tondo C, Mantica M, Russo G, Avella A, De Luca L, Pappalardo A, et al. Pulmonary vein vestibule ablation for the control of atrial fibrillation in patients with impaired left ventricular function. Pacing Clin Electrophysiol 2006;29:962-70.

598.	Tondo C, Tritto M, Landolina M, Girolamo P, Bencardino G, Moltrasio M, et al. Rhythm-Symptom Correlation in Patients on Continuous Monitoring After Catheter Ablation of Atrial Fibrillation. J Cardiovasc Electrophysiol 2014;25:154-60.

599.	Toso E, Gagliardi M, Peyracchia M, Angelini F, Anselmino M, Ferraris F, et al. Long-term efficacy and impact on quality of life of atrial fibrillation catheter ablation in competitive athletes. J Sports Med Phys Fitness 2022;62:1266-71.

600.	Tripathi U, Kapoor A, Kumar Agarwal S, Tewari P, Pande S, Chandra B, et al. Flecainide for conversion and maintenance of sinus rhythm after mitral valve replacement in rheumatic atrial fibrillation. Indian Heart J 2023;75:352-6.

601.	Trymbulak K, Ding E, Marino F, Wang Z, Saczynski JS. Mobile health assessments of geriatric elements in older patients with atrial fibrillation: The Mobile SAGE-AF Study (M-SAGE). Cardiovasc Digit Health J 2020;1:123-9.

602.	Tse HF, Lam YM, Lau CP, Cheung BM, Kumana CR. Comparison of digoxin versus low-dose amiodarone for ventricular rate control in patients with chronic atrial fibrillation. Clin Exp Pharmacol Physiol 2001;28:446-50.

603.	Tse HF, Newman D, Ellenbogen KA, Buhr T, Markowitz T, Lau CP. Effects of ventricular rate regularization pacing on quality of life and symptoms in patients with atrial fibrillation (Atrial fibrillation symptoms mediated by pacing to mean rates [AF SYMPTOMS study]). Am J Cardiol 2004;94:938-41.

604.	Tse HF, Sin PY, Siu CW, Tsang V, Lam CL, Lau CP. Successful pulmonary vein isolation using transvenous catheter cryoablation improves quality-of-life in patients with atrial fibrillation. Pacing Clin Electrophysiol 2005;28:421-4.

605.	Tsounis D, Ioannidis A, Bouras G, Raikou M, Giannopoulos G, Deftereos S, et al. Assessment of health-related quality of life in a greek symptomatic population with atrial fibrillation: correlation with functional status and echocardiographic indices. Hellenic J Cardiol 2014;55:475-85.

606.	Tsuneda T, Yamashita T, Fukunami M, Kumagai K, Niwano S, Okumura K, et al. Rate control and quality of life in patients with permanent atrial fibrillation: the Quality of Life and Atrial Fibrillation (QOLAF) Study. Circ J 2006;70:965-70.

607.	Turchioe MR, Mangal S, Ancker JS, Gwyn J, Varosy P, Slotwiner D. 'Replace uncertainty with information': shared decision-making and decision quality surrounding catheter ablation for atrial fibrillation. Eur J Cardiovasc Nurs 2023;22:430-40.

608.	Turker Y, Ekinozu I, Aytekin S, Turker Y, Basar C, Baltaci D, et al. Comparison of Changes in Anxiety and Depression Level Between Dabigatran and Warfarin Use in Patients With Atrial Fibrillation. Clin Appl Thromb Hemost 2017;23:164-7.

609.	Ueda I, Kohsaka S, Ikemura N, Kimura T, Katsumata Y, Takemura R, et al. Patient concern regarding bleeding side effects from oral anticoagulation therapy for atrial fibrillation: an analysis from the multicentre KiCS-AF registry. Eur J Cardiovasc Nurs 2024;23:358-66.

610.	Uhe T, Beimel S, Langhammer R, Stegmann T, Hindricks G, Laufs U, et al. Patients' attitude towards a sham-controlled trial on pulmonary vein isolation in atrial fibrillation. Clin Res Cardiol 2022;111:114-23.

611.	Ulla W, Anna S, Arestedt K. Development and validation of an arrhythmia-specific scale in tachycardia and arrhythmia with focus on health-related quality of life. J Cardiovasc Nurs 2015;30:98-108.

612.	Valderrábano M, Peterson LE, Bunge R, Prystash M, Dave AS, Nagueh S, et al. Vein of Marshall ethanol infusion for persistent atrial fibrillation: VENUS and MARS clinical trial design. Am Heart J 2019;215:52-61.

613.	van Breugel H, Parise O, Nieman FHM, Accord RE, Lucà F, Lozekoot P, et al. Does sinus rhythm conversion after cardiac surgery affect postoperative health- related quality of life? J Cardiothorac Surg 2016;11:75.

614.	van Breugel HN, Nieman FH, Accord RE, Van Mastrigt GA, Nijs JF, Severens JL, et al. A prospective randomized multicenter comparison on health-related quality of life: the value of add-on arrhythmia surgery in patients with paroxysmal, permanent or persistent atrial fibrillation undergoing valvular and/or coronary bypass surgery. J Cardiovasc Electrophysiol 2010;21:511-20.

615.	van Deelen BA, van den Bemt PM, Egberts TC, van 't Hoff A, Maas HA. Cognitive impairment as determinant for sub-optimal control of oral anticoagulation treatment in elderly patients with atrial fibrillation. Drugs Aging 2005;22:353-60.

616.	van den Berg MP, Hassink RJ, Tuinenburg AE, van Sonderen EF, Lefrandt JD, de Kam PJ, et al. Quality of life in patients with paroxysmal atrial fibrillation and its predictors: importance of the autonomic nervous system. Eur Heart J 2001;22:247-53.

617.	van den Berg MP, Ranchor AV, van Sonderen FL, van Gelder IC, van Veldhuisen DJ. Paroxysmal atrial fibrillation, quality of life and neuroticism. Neth J Med 2005;63:170-4.

618.	van den Dries CJ, Oudega R, Elvan A, Rutten FH, van de Leur S, Bilo HJG, et al. Integrated management of atrial fibrillation including tailoring of anticoagulation in primary care: study design of the ALL-IN cluster randomised trial. BMJ Open 2017;7:e015510.

619.	van der Heijden CAJ, Weberndorfer V, Vroomen M, Luermans JG, Chaldoupi SM, Bidar E, et al. Hybrid Ablation Versus Repeated Catheter Ablation in Persistent Atrial Fibrillation: A Randomized Controlled Trial. JACC Clin Electrophysiol 2023;9:1013-23.

620.	Van Gelder IC, Van Veldhuisen DJ, Crijns HJ, Tuininga YS, Tijssen JG, Alings AM, et al. RAte Control Efficacy in permanent atrial fibrillation: a comparison between lenient versus strict rate control in patients with and without heart failure. Background, aims, and design of RACE II. Am Heart J 2006;152:420-6.

621.	Veasey RA, Silberbauer J, Schilling RJ, Morgan JM, Paul V, Furniss SS, et al. The evaluation of pulmonary vein isolation and wide-area left atrial ablation to treat atrial fibrillation in patients with implanted permanent pacemakers: the Previously Paced Pulmonary Vein Isolation Study. Heart 2010;96:1037-42.

622.	Verdicchio CV, Mahajan R, Middeldorp ME, Gallagher C, Mishima RS, Lau DH, et al. Influence of sex on efficacy of exercise training for patients with symptomatic atrial fibrillation: insights from the ACTIVE-AF randomized controlled trial. Eur J Prev Cardiol 2023;30:2006-14.

623.	Verma A, Ha ACT, Kirchhof P, Hindricks G, Healey JS, Hill MD, et al. The Optimal Anti-Coagulation for Enhanced-Risk Patients Post-Catheter Ablation for Atrial Fibrillation (OCEAN) trial. Am Heart J 2018;197:124-32.

624.	Verma A, Haines DE, Boersma LV, Sood N, Natale A, Marchlinski FE, et al. Influence of monitoring and atrial arrhythmia burden on quality of life and health care utilization in patients undergoing pulsed field ablation: A secondary analysis of the PULSED AF trial. Heart Rhythm 2023;20:1238-45.

625.	Vermeer J, Vinck T, de Louw B, Slingerland S, van 't Veer M, Regis M, et al. Improving outcomes of AF ablation by integrated personalized lifestyle interventions: rationale and design of the prevention to improve outcomes of PVI (POP) trial. Clin Res Cardiol 2023;112:716-23.

626.	Vermeer J, Houterman S, Medendorp N, van der Voort P, Dekker L. Body mass index and pulmonary vein isolation: real-world data on outcomes and quality of life. Europace 2024;26:euae157.

627.	von Eisenhart Rothe A, Bielitzer M, Meinertz T, Limbourg T, Ladwig KH, Goette A. Predictors of discordance between physicians' and patients' appraisals of health-related quality of life in atrial fibrillation patients: findings from the Angiotensin II Antagonist in Paroxysmal Atrial Fibrillation Trial. Am Heart J 2013;166:589-96.

628.	von Eisenhart Rothe AF, Goette A, Kirchhof P, Breithardt G, Limbourg T, Calvert M, et al. Depression in paroxysmal and persistent atrial fibrillation patients: a cross-sectional comparison of patients enroled in two large clinical trials. Europace 2014;16:812-9.

629.	Vroomen M, La Meir M, Maesen B, Luermans JGL, Vernooy K, Essers B, et al. Hybrid thoracoscopic surgical and transvenous catheter ablation versus transvenous catheter ablation in persistent and longstanding persistent atrial fibrillation (HARTCAP-AF): study protocol for a randomized trial. Trials 2019;20:370.

630.	von Oppell UO, Masani N, O'Callaghan P, Wheeler R, Dimitrakakis G, Schiffelers S. Mitral valve surgery plus concomitant atrial fibrillation ablation is superior to mitral valve surgery alone with an intensive rhythm control strategy. Eur J Cardiothorac Surg 2009;35:641-50.

631.	Wagner MK, Zwisler AO, Risom SS, Svendsen JH, Christensen AV, Berg SK. Sex differences in health status and rehabilitation outcomes in patients with atrial fibrillation treated with ablation: Results from the CopenHeartRFA trial. Eur J Cardiovasc Nurs 2018;17:123-35.

632.	Wahlstrom M, Rosenqvist M, Medin J, Walfridsson U, Rydell-Karlsson M. MediYoga as a part of a self-management programme among patients with paroxysmal atrial fibrillation - a randomised study. Eur J Cardiovasc Nurs 2020;19:74-82.

633.	Wahlstrom M, Rydell Karlsson M, Medin J, Frykman V. Effects of yoga in patients with paroxysmal atrial fibrillation - a randomized controlled study. Eur J Cardiovasc Nurs 2017;16:57-63.

634.	Walfridsson H, Walfridsson U, Nielsen JC, Johannessen A, Raatikainen P, Janzon M, et al. Radiofrequency ablation as initial therapy in paroxysmal atrial fibrillation: results on health-related quality of life and symptom burden. The MANTRA-PAF trial. Europace 2015;17:215-21.

635.	Walfridsson U, Brohede D, Walfridsson H. ASK FOR IT: An Internet-based educational program for patients with atrial fibrillation - Results from a pilot study and design of the randomized, controlled, multicenter ASK FOR IT study. Cardiovasc Digit Health J 2020;1:160-8.

636.	Walfridsson U, Hassel Jonsson A, Karlsson LO, Liuba I, Almroth H, Sandgren E, et al. Symptoms and health-related quality of life 5 years after catheter ablation of atrial fibrillation. Clin Cardiol 2022;45:42-50.

637.	Walfridsson U, Steen Hansen P, Charitakis E, Almroth H, Jönsson A, Karlsson LO, et al. Gender and age differences in symptoms and health-related quality of life in patients with atrial fibrillation referred for catheter ablation. Pacing Clin Electrophysiol 2019;42:1431-9.

638.	Walfridsson U [a], Walfridsson H, Middeldorp ME, Sanders P, Årestedt K. Validation of the English version of the arrhythmia-specific questionnaire in tachycardia and arrhythmia (ASTA): a Rasch evaluation study. J Patient Rep Outcomes 2022;6:90.

639.	Walfridsson U, Arestedt K, Stromberg A. Development and validation of a new Arrhythmia-Specific questionnaire in Tachycardia and Arrhythmia (ASTA) with focus on symptom burden. Health Qual Life Outcomes 2012;10:44.

640.	Walters TE, Wick K, Tan G, Mearns M, Joseph SA, Morton JB, et al. Psychological Distress and Suicidal Ideation in Patients With Atrial Fibrillation: Prevalence and Response to Management Strategy. J Am Heart Assoc 2018;7:e005502.

641.	Walters TE, Wick K, Tan G, Mearns M, Joseph SA, Morton JB, et al. Symptom severity and quality of life in patients with atrial fibrillation: Psychological function outweighs clinical predictors. Int J Cardiol 2019;279:84-9.

642.	Wang Q, Jiang SL, Liu X, Yang YQ. Repeat Catheter Ablation of Long-standing Persistent Atrial Fibrillation in Patients with a Total Atrial Fibrillation Duration of More Than 2 Years: Effects of the CHA2DS2-VASc Score and Estimated Glomerular Filtration Rate on the Outcomes. Intern Med 2016;55:2537-47.

643.	Wang W, Darleen L, Abu H, McManus DD, Mailhot T, Gurwitz JH, et al. Hearing loss and cognitive decline among older adults with atrial fibrillation: the SAGE-AF study. J Geriatr Cardiol 2020;17:177-83.

644.	Wang W [a], Lessard D, Saczynski J, Goldberg RJ, Go AS, Paul T, et al. Incident frailty and cognitive impairment by heart failure status in older patients with atrial fibrillation: the SAGE-AF study. J Geriatr Cardiol 2020;17:653-8.

645.	Wang W, Lessard D, Saczynski JS, Goldberg RJ, Mehawej J, Gracia E, et al. Prognostic value of geriatric conditions for death and bleeding in older patients with atrial fibrillation. Int J Cardiol Heart Vasc 2021;33:100739.

646.	Wang X, Wang Z, Yan X, Huang M, Wu Y. Radiofrequency and cryoballoon ablation improve cognitive function in patients with atrial fibrillation. Medicine (Baltimore) 2021;100:e26914.

647.	Wang P, Yan D, Peng Y, Zhang Z. Efficacy and Safety of Sustained-Release Metoprolol With Dronedarone After Radiofrequency Ablation of Paroxysmal Atrial Fibrillation: A Propensity Matched Analysis. Am J Cardiol 2022;185:37-45.

648.	Wang W, Lessard D, Kiefe CI, Goldberg RJ, Parish D, Helm R, et al. Differential effect of anticoagulation according to cognitive function and frailty in older patients with atrial fibrillation. J Am Geriatr Soc 2023;71:394-403.

649.	Wang FJ, Zhang C, Cai MM, Zhang JQ, Wang HX. Personal mastery and quality of life in patients with atrial fibrillation after radiofrequency ablation: The mediating role of health promoting behavior. Heart Lung 2024;67:46-52.

650.	Ward KJ, Willett JE, Bucknall C, Gill JS, Kamalvand K. Atrial arrhythmia suppression by atrial overdrive pacing: pacemaker Holter assessment. Europace 2001;3:108-14.

651.	Washida K, Kowa H, Hamaguchi H, Kanda F, Toda T. Validation of the R(2)CHADS(2) and CHADS(2) Scores for Predicting Post-stroke Cognitive Impairment. Intern Med 2017;56:2719-25.

652.	Wazni OM, Marrouche NF, Martin DO, Verma A, Bhargava M, Saliba W, et al. Radiofrequency ablation vs antiarrhythmic drugs as first-line treatment of symptomatic atrial fibrillation: a randomized trial. JAMA 2005;293:2634-40.

653.	Wazni O, Dandamudi G, Sood N, Hoyt R, Tyler J, Durrani S, et al. Quality of life after the initial treatment of atrial fibrillation with cryoablation versus drug therapy. Heart Rhythm 2022;19:197-205.

654.	Weerasooriya R, Davis M, Powell A, Szili-Torok T, Shah C, Whalley D, et al. The Australian Intervention Randomized Control of Rate in Atrial Fibrillation Trial (AIRCRAFT). J Am Coll Cardiol 2003;41:1697-702.

655.	Weerasooriya R, Jais P, Hocini M, Scavee C, MacLe L, Hsu LF, et al. Effect of catheter ablation on quality of life of patients with paroxysmal atrial fibrillation. Heart Rhythm 2005;2:619-23.

656.	Wei W, Ma C, Tian Z, Bu M, Liu W, Song M. Impact of Early Rehabilitation Nursing on Postoperative Cardiac Function and Quality of Life in Patients with Atrial Fibrillation. Altern Ther Health Med 2024:AT10252.

657.	Wettervik V, Schwieler J, Bergfeldt L, Kenneback G, Jensen S, Rubulis A, et al. Long-term health-related quality of life and rhythm outcomes of catheter ablation versus antiarrhythmic drugs in patients with atrial fibrillation. Heart Rhythm 2025;22:944-51.

658.	Wieczorek J, Mizia-Stec K, Lasek-Bal A, Wieczorek P, Hoffmann A, Nowak S, et al. CHA2DS2-Vasc score, age and body mass index as the main risk factors of hyperintense brain lesions in asymptomatic patients with paroxysmal non-valvular atrial fibrillation. Int J Cardiol 2016;215:476-81.

659.	Wijtvliet E, Tieleman RG, van Gelder IC, Pluymaekers N, Rienstra M, Folkeringa RJ, et al. Nurse-led vs. usual-care for atrial fibrillation. Eur Heart J 2020;41:634-41.

660.	Willits I, Keltie K, Linker N, de Belder M, Henderson R, Patrick H, et al. Left atrial appendage occlusion in the UK: prospective registry and data linkage to Hospital Episode Statistics. Eur Heart J Qual Care Clin Outcomes 2021;7:468-75.

661.	William J, Chieng D, Curtin AG, Sugumar H, Ling LH, Segan L, et al. Radiofrequency catheter ablation of persistent atrial fibrillation by pulmonary vein isolation with or without left atrial posterior wall isolation: long-term outcomes of the CAPLA trial. Eur Heart J 2025;46:132-43.

662.	Wilson RE, Rush KL, Reid RC, Laberge CG. The symptom experience of early and late treatment seekers before an atrial fibrillation diagnosis. Eur J Cardiovasc Nurs 2021;20:231-42.

663.	Wilton SB, Kavanagh KM, Aggarwal SG, Philippon F, Yee R, Cowan K, et al. Association of rate-controlled persistent atrial fibrillation with clinical outcome and ventricular remodelling in recipients of cardiac resynchronization therapy. Can J Cardiol 2011;27:787-93.

664.	Wokhlu A, Monahan KH, Hodge DO, Asirvatham SJ, Friedman PA, Munger TM, et al. Long-term quality of life after ablation of atrial fibrillation the impact of recurrence, symptom relief, and placebo effect. J Am Coll Cardiol 2010;55:2308-16.

665.	Wood KA, Alam AB, Chen LY, Soliman EZ, Quyyumi AA, Alonso A. Factors Associated With Fatigue in Persons With Atrial Fibrillation in the Atherosclerosis Risk in Communities (ARIC) Study. Biol Res Nurs 2024;26:350-60.

666.	Wood KA, Barnes AH. Variations in follow-up after atrial fibrillation ablation. J Am Assoc Nurse Pract 2020;33:602-10.

667.	Wood KA, Barnes AH, Paul S, Hines KA, Jackson KP. Symptom challenges after atrial fibrillation ablation. Heart Lung 2017;46:425-31.

668.	Wood KA, Stewart AL, Drew BJ, Scheinman MM, Frolicher ES. Development and initial psychometric evaluation of the Patient Perspective of Arrhythmia Questionnaire. Res Nurs Health 2009;32:504-16.

669.	Woo BFY, Tam WWS, Rangpa T, Liau WF, Nathania J, Lim TW. A Nurse-Led Integrated Chronic Care E-Enhanced Atrial Fibrillation (NICE-AF) Clinic in the Community: A Preliminary Evaluation. Int J Environ Res Public Health 2022;19:4467.

670.	Worck R, Sørensen SK, Johannessen A, Ruwald MH, Hansen ML, Haugdal M, et al. Posterior wall isolation in persistent atrial fibrillation. Long-term outcomes of a repeat procedure strategy. J Interv Card Electrophysiol 2023;66:971-9.

671.	Wozniak-Skowerska IM, Skowerski MJ, Hoffmann A, Nowak S, Faryan M, Kolasa J, et al. Quality of life in patients with paroxysmal atrial fibrillation after circumferential pulmonary vein ablation. Kardiol Pol 2016;74:244-50.

672.	Wu G, Huang H, Cai L, Yang Y, Liu X, Yu B, et al. Long-term observation of catheter ablation vs. pharmacotherapy in the management of persistent and long-standing persistent atrial fibrillation (CAPA study). Europace 2021;23:731-9.

673.	Wu L, Lu Y, Zheng L, Qiao YU, Chen G, Ding L, et al. Comparison of Radiofrequency Catheter Ablation Between Asymptomatic and Symptomatic Persistent Atrial Fibrillation: A Propensity Score Matched Analysis. J Cardiovasc Electrophysiol 2016;27:531-5.

674.	Wu Y, Lin J, Gong B, Wang L, Ruan Z, Xu K. Cardiac Rehabilitation in Atrial Fibrillation Patients With Left Atrial Appendage Occlusion: A RANDOMIZED TRIAL. J Mol Signal 2022;42:266-71.

675.	Wynn GJ, Das M, Bonnett LJ, Gupta D. Quality-of-life benefits of catheter ablation of persistent atrial fibrillation: a reanalysis of data from the SARA study. Europace 2015;17:222-4.

676.	Wynn GJ, Todd DM, Webber M, Bonnett L, McShane J, Kirchhof P, et al. The European Heart Rhythm Association symptom classification for atrial fibrillation: validation and improvement through a simple modification. Europace 2014;16:965-72.

677.	Wynn GJ, Panikker S, Morgan M, Hall M, Waktare J, Markides V, et al. Biatrial linear ablation in sustained nonpermanent AF: Results of the substrate modification with ablation and antiarrhythmic drugs in nonpermanent atrial fibrillation (SMAN-PAF) trial. Heart Rhythm 2016;13:399-406.

678.	Xiao Y, Wang X, Yang J, Liu K, Li M, Ma Z, et al. Huatan Dingji Decoction intervening in atrial fibrillation: protocol for a randomized double-blind single-simulated placebo-controlled clinical trial. Trials 2021;22:693.

679.	Yagishita A, Yamauchi Y, Sato H, Yamashita S, Hirao T, Miyamoto T, et al. Improvement in the Quality of Life and Exercise Performance in Relation to the Plasma B-Type Natriuretic Peptide Level After Catheter Ablation in Patients With Asymptomatic Persistent Atrial Fibrillation. Circ J 2017;81:444-9.

680.	Yamamoto M, Watanabe E, Suzuki T, Yamazaki T, Ohtsu H, Ozaki Y, et al. Association between the quality of life and asymptomatic episodes of paroxysmal atrial fibrillation in the J-RHYTHM II study. J Cardiol 2014;64:64-9.

681.	Yamashita T, Kumagai K, Koretsune Y, Mitamura H, Okumura K, Ogawa S, et al. A new method for evaluating quality of life specific to patients with atrial fibrillation: Atrial fibrillation quality of life questionnaire (AFQLQ). Jpn J Electrocardiol 2003;23:332-43.

682.	Yamashita T, Komatsu T, Kumagai K, Uno K, Niwano S, Fujiki A, et al. Internal consistency and reproducibility of atrial fibrillation specific quality of life questionnaire (AFQLQ). Jpn J Electrocardiol 2005;25:488-94.

683.	Yamashita T, Inoue H, Okumura K, Kodama I, Aizawa Y, Atarashi H, et al. Randomized trial of angiotensin II-receptor blocker vs. dihydropiridine calcium channel blocker in the treatment of paroxysmal atrial fibrillation with hypertension (J-RHYTHM II study). Europace 2011;13:473-9.

684.	Yamashita T, Kimura T, Ikemura N, Niimi N, Tsuzuki I, Seki Y, et al. Characteristics and health-status outcomes in patients with atrial fibrillation detected via health screening. Clin Cardiol 2023;46:32-40.

685.	Yamin M, Salim S, Setiati S, Pudianto AP, Zulmiyusrini P, Nasution SA, et al. Validity and reliability studies of the Indonesian version of Atrial Fibrillation Severity Scale (AFSS). BMC Cardiovasc Disord 2023;23:216.

686.	Yamin M [a], Salim S, Setiati S, Pudianto AP, Zulmiyusrini P, Tondas AE, et al. Validity and Reliability Studies of the Indonesian Version of Arrhythmia-Specific Questionnaire in Tachycardia and Arrhythmia (ASTA). Acta Med Indones 2023;55:165-71.

687.	Yan H, Du Y-X, Wu F-Q, Lu X-Y, Chen R-M, Zhang Y. Effects of nurse-led multidisciplinary team management on cardiovascular hospitalization and quality of life in patients with atrial fibrillation: Randomized controlled trial. Int J Nurs Stud 2022;127:104159.

688.	Yan Q, Liang J, Yuan Y, Li Y, Fan J, Wu W, et al. Association between personality type and patient-reported outcomes (PRO) in patients with atrial fibrillation. BMC Cardiovasc Disord 2024;24:437.

689.	Yanagawa B, Holmes SD, Henry L, Hunt S, Ad N. Outcome of concomitantCox-maze III procedure using an argon-based cryosurgical system: a single-center experience with 250 patients. Ann Thorac Surg 2013;95:1633-9.

690.	Yang YE, Bo GAO, Yuan LV, Tian-Tian XU, Si-Si Z, Xiao-Li LU, et al. His bundle pacing versus left bundle branch pacing on ventricular function in atrial fibrillation patients referred for pacing: a prospective crossover comparison. J Geriatr Cardiol 2023;20:51-60.

691.	Yao RJR, Macle L, Deyell MW, Tang L, Hawkins NM, Sedlak T, et al. Impact of Female Sex on Clinical Presentation and Ablation Outcomes in the CIRCA-DOSE Study. JACC Clin Electrophysiol 2020;6:945-54.

692.	Yoshihisa A, Kono S, Kaneshiro T, Ichijo Y, Misaka T, Yamada S, et al. Impaired brain activity in patients with persistent atrial fibrillation assessed by near-infrared spectroscopy and its changes after catheter ablation. Sci Rep 2022;12:7866.

693.	Younes H, Sohns C, Akoum N, Feng H, Tsakiris E, Hajjar AHE, et al. Sex-specific outcomes and left atrial remodeling following catheter ablation of persistent atrial fibrillation: results from the DECAAF II trial. J Interv Card Electrophysiol 2024;67:1843-50.

694.	Yu S, Zhao Q, Wu P, Qin M, Huang H, Cui H, et al. Effect of anxiety and depression on the recurrence of paroxysmal atrial fibrillation after circumferential pulmonary vein ablation. J Cardiovasc Electrophysiol 2012;23 Suppl 1:S17-23.

695.	Zadeh FY, Moeini M, Shafie D. Evaluation of the effect of adherence to treatment regimen program on quality of life in atrial fibrillation patients hospitalized in Shahid Chamran Hospital in Isfahan in 2017. Revista Latinoam Hipertens 2019;14:297‐304.

696.	Zeitler EP, Li Y, Silverstein AP, Russo AM, Poole JE, Daniels MR, et al. Effects of Ablation Versus Drug Therapy on Quality of Life by Sex in Atrial Fibrillation: Results From the CABANA Trial. J Am Heart Assoc 2023;12:e027871.

697.	Zenger B, Zhang M, Lyons A, Bunch TJ, Fang JC, Freedman RA, et al. Patient-reported outcomes and subsequent management in atrial fibrillation clinical practice: Results from the Utah mEVAL AF program. J Cardiovasc Electrophysiol 2020;31:3187-95.

698.	Zenger B, Torre M, Zhang Y, Boo L, Jamshidian F, Young J, et al. Comprehensive analysis of same day discharge after atrial fibrillation ablation: Clinical, cost, and patient reported outcomes. J Cardiovasc Electrophysiol 2024;35:1570-8.

699.	Zeren M, Demir R, Karcı M, Yiğit Z, Uzunhasan I, Gürses HN. Atrial Fibrillation Impact Questionnaire (AFImpact): Validity and reliability of the Turkish version. Turk Kardiyol Dern Ars 2021;49:395-403.

700.	Zeren M, Karci M, Demir R, Gurses HN, Oktay V, Uzunhasan I, et al. Cut-off values of 6-min walk test and sit-to-stand test for determining symptom burden in atrial fibrillation. Ir J Med Sci 2022;191:2587-95.

701.	Zhang XD, Gu J, Jiang WF, Zhao L, Zhou L, Wang YL, et al. Optimal rhythm-control strategy for recurrent atrial tachycardia after catheter ablation of persistent atrial fibrillation: a randomized clinical trial. Eur Heart J 2014;35:1327‐34.

702.	Zhang CL, Chen F, Li XM, Li XY. [The status of patient-reported outcomes and their correlation with the number of hospitalizations within 1 year in patients with atrial fibrillation]. Chung Hua Hsin Hsueh Kuan Ping Tsa Chih 2023;51:958-62.

703.	Zhang X, Wang Q, Dong Y, Jia Y, Hou Z, Deng W, et al. Acupuncture-assisted anaesthesia for catheter ablation of atrial fibrillation to reduce the consumption of morphine hydrochloride and postoperative nausea and vomiting (PONV): study protocol for a randomised controlled trial. BMJ Open 2022;12:e068318.

704.	Zito M, Muscari A, Marini E, Di Iorio A, Puddu GM, Abate G. Silent lacunar infarcts in elderly patients with chronic non valvular atrial fibrillation. Aging (Milano). 1996;8(5):341-6.

705.	Zucchelli G, Chun KRJ, Khelae SK, Földesi C, Kueffer FJ, van Bragt KA, et al. Impact of first-line cryoablation for atrial fibrillation on healthcare utilization, arrhythmia disease burden and efficacy outcomes: real-world evidence from the Cryo Global Registry. J Interv Card Electrophysiol 2023;66:711-22.

706.	Zulmiyusrini P, Yamin M, Muhadi M, Kurniawan J, Salim S. The validity and reliability of Indonesian version of atrial fibrillation effect on quality of life (AFEQT) questionnaire for atrial fibrillation patients. J Patient Rep Outcomes 2023;7:1-10.

707.	Zuk A, Piotrowski R, Sikorska A, Kowalik I, Kulakowski P, Baran J. Association between ablation-induced baroreceptor reflex modification and procedure efficacy in patients with atrial fibrillation. Front Cardiovasc Med 2024;11:1474002.

708.	Zylla MM, Leiner J, Rahm AK, Hoffmann T, Lugenbiel P, Schweizer P, et al. Catheter ablation of atrial fibrillation in women with heart failure with preserved ejection fraction. Front Cardiovasc Med 2024;11:1463815.

709.	Zylla MM, Özdemir B, Hochadel M, Zeymer U, Akin I, Grau A, et al. Community-based analysis of stroke prevention and effect of public interventions in atrial fibrillation: results from the ARENA project. Clin Res Cardiol 2025;114:138-149
